# Impact of COVID-19 Pandemic on the Clinical Activities in Obstetrics and Gynecology: A National Survey in China

**DOI:** 10.3389/fmed.2021.633477

**Published:** 2021-07-30

**Authors:** Lei Li, Yang Cao, Junning Fan, Ting Li, Jinghe Lang, Heping Zhang, Jun Lv, Lan Zhu, Chinese Medical Doctor Association

**Affiliations:** ^1^Department of Obstetrics and Gynecology, Peking Union Medical College Hospital, Beijing, China; ^2^Department of Epidemiology and Biostatistics, School of Public Health, Peking University Health Science Center, Beijing, China; ^3^Department of Biostatistics, Yale University School of Public Health, New Haven, CT, United States

**Keywords:** COVID-19 pandemic, Mainland China, city category, hospital levels, obstetrics and gynecology, clinical practice

## Abstract

**Objective:** Few studies have quantified the influence of coronavirus disease 2019 (COVID-19) pandemic on medical providers. This is the first national study to investigate the impact of the pandemic on physicians practicing obstetrics and gynecology in China.

**Methods:** A two-stage, stratified, cluster sampling method was performed based on the city categories (category 1, fewer than 10,000 beds; category 2, 10,000–30,000; and category 3, more than 30,000) and public hospital levels (primary, secondary, and tertiary). Physicians practicing obstetrics and gynecology reported the relevant changes in their general clinical activities and changes in the management of specific diseases or conditions occurring during the periods that they were most strongly affected. These changes were compared by municipal and hospital characteristics.

**Results:** Questionnaires were collected from a representative sample of 11,806 physicians actively practicing obstetrics and gynecology in 779 hospitals from 157 cities of 31 provinces. Except emergency visits and online consultations, category 3 cities, tertiary hospitals and general hospitals had greater reductions in overall clinical activities than category 1 cities, primary hospitals and specialized hospitals (all adjusted *p* < 0.05), respectively. The differences also existed in the management of specific diseases and conditions, especially for less urgent conditions, including cervical cancer screening, instructions regarding contraception and miscarriage, and assisted reproduction (all *p* < 0.05).

**Conclusions:** During the COVID-19 pandemic, the clinical obstetrics and gynecology activities in China markedly decreased, with significant differences across municipal and hospital characteristics.

**Trial Registration:** This study was registered with ClinicalTrials.gov on July 27, 2020 (NCT04491201).

## Introduction

Coronavirus disease 2019 (COVID-19) has caused substantial damage to China since its outbreak and spread in the first half of 2020. As of August 24, 2020, the cumulative number of confirmed cases of COVID-19 reported in China was 84,967, among which 68,139 (80.2%) were from Hubei Province (1). Although there have been numerous studies performed pertaining to this pandemic, only a few studies have quantitatively assessed the impact of this pandemic on medical providers in China (2–4). In a survey of junior doctors in the United Kingdom, most units limited face-to-face antenatal clinics and suspended elective gynecology services (5). Other reports showed the impact of COVID-19 lockdowns on the treatment of gynecologic cancer patients (6, 7), admissions to gynecological emergency care (8, 9), emergency surgery (10), and maternal and newborn healthcare (11). However, these studies had limited sample sizes and voluntary response sampling methods, restricting the generalizability of their findings. As well as in other fields, the lack of sufficient health and legal protection for surgeons and patients may result in a special reduction in the volume of surgical interventions during COVID pandemic and the immediately following period, therefore, determining inability to ensure health care to all patients (12–14).

As reported by the WHO, people living with non-communicable diseases (NCDs) are more vulnerable to becoming severely ill with or dying from COVID-19. The more severe the transmission phase of the COVID-19 pandemic, the more NCD services are disrupted (15). Although most physicians in obstetrics and gynecology were not involved in the immediate response to the pandemic, they represent a major force at the crossroads of politics, social justice, and reproductive rights in the fight for the preservation of reproductive healthcare (16). A representative quantitative assessment of the changes in clinical activities of obstetrics and gynecology during the pandemic would not only provide vital and accurate information for developing coping strategies during this time (17, 18), but also offer suggestions for health care reform, leading to the development of more flexible, and effective health care systems (19).

As previous studies were confined to local regions or used convenience sampling methods hence providing limited information, we performed a national survey in China among registered physicians who practice obstetrics and gynecology in public hospitals. To our knowledge, this is the first nationally representative survey of physicians describing the impact of the COVID-19 pandemic on clinical activities. We particularly examined whether such an impact varied between different municipal and hospital characteristics. In this way we were to explore the practical effects of COVID pandemic on clinical practicing in the view of obstetricians and gynecologists.

## Methods

### Sampling Design and Participants

This study used a stratified two-stage random cluster sampling design as to obtain a representative sample and minimize selection bias. Considering the vastness of the territory and large size of the population as well as the unbalanced distribution of healthcare resources across mainland China, all 31 provinces, municipalities and autonomous regions (the latter two have same administrative status as provinces) were included in the study. In the first stage, within each province, three strata of cities were generated according to the total number of hospital beds, namely, category 1 (fewer than 10,000), category 2 (10,000 to 30,000), and category 3 (more than 30,000). Two cities were randomly chosen from each stratum, if applicable. In the second stage, in each selected city, three strata of hospitals were generated according to the hospital levels, namely, primary, secondary, and tertiary. All physicians of obstetrics and gynecology in the chosen hospitals received a link to an electronic questionnaire (https://www.wjx.cn). The data were obtained from completed questionnaires, and were stored in the same online database. A more detailed sampling methods and results were described in Supplementary Materials.

The eligible participants were registered physicians working in the obstetrics and gynecology from public hospitals who agreed to participate in the survey. Participants were excluded if they were registered as assistant physician or midwife, or if they retired from routine medical practice. Participants presented their electronic consents when they submitted their questionnaire. The Institutional Review Board of Peking Union Medical College Hospital approved the study (No. S-K1291). This study was registered with ClinicalTrials.gov on July 27, 2020 (NCT04491201).

### Data Collection

The questionnaire was developed based on the current clinical activities in China, and consisted of 31 items: 10 pertained to the participants' sociodemographics, one pertained to the period that was most strongly affected by the pandemic (January to June as multiple options), 7 pertained to general clinical activities (outpatient visits and appointments, emergency visits, surgical volumes, consultant requests, admission arrangements and online consultations), and 13 pertained to specific diseases or conditions (preconception counseling, prenatal examinations, prenatal diagnoses, instructions regarding contraception and miscarriage, assisted reproduction, outpatient surgeries and procedures, emergency obstetrical and gynecological surgeries, cervical cancer screening, treatment for benign neoplasms, malignancies and pelvic floor dysfunctions, and follow-up for malignancies). For each clinical activity, the responder was asked to select options to describe the changes during the pandemic from January to June 2020 as irrelevant to his/her specialty, complete shutdown, decreased by >50%, decreased by 25–50%, decreased by <25%, no change or increase. For the item “online consultations,” based on the experience gained while constructing the questionnaire, the options consisted of irrelevant, decreased by >50%, decreased by <50%, no change, increased by <50%, and increased by >50%. For each item, the respondent was also asked to evaluate the changes after July 1, 2020, with the following options of the same, less than or more than the level in 2019. A team of 20 physicians from Peking Union Medical College Hospital had validated and modified the questionnaire, and these physicians were excluded in the formal survey.

### Statistical Analysis

Unweighted demographic characteristics of all participants were stratified by the city categories, hospital levels, hospital natures (general vs. specialized hospitals for women health) and various provinces (Hubei Province vs. other provinces). Continuous variables are presented as the means with standard deviations, and categorical variables are presented as percentages. All the calculations were then weighted to represent obstetricians and gynecologists nationwide and analyzed with the “Survey data analysis” module in Stata (version 15.0, StataCorp, TX, USA). The weights incorporated sampling probabilities, non-response adjustments, and poststratification adjustments. The weighted percentages of changes in clinical activities and changes in the management of specific diseases or conditions were compared between various municipal and hospital characteristics mentioned above by χ^2^-test. Multinomial logistic analysis was used to simultaneously examine the associations of city categories, hospital levels, and natures with changes in clinical activities. The results are presented as relative risk ratios (RRRs) and 95% confidence intervals (95% CIs). Unless otherwise stated, all analyses were performed with a two-sided significance level of 0.05 performed by Stata.

## Results

### Sampling Design and Participating Results

Overall, 11,806 physicians from 779 hospitals in 157 cities of 31 provinces completed the questionnaires from August 1 to August 10, 2020, corresponding to 7.6% of the 155,787 registered, actively practicing obstetrics and gynecology physicians in China (20). The response rates of physicians and hospitals were 93.8 and 82.0%, respectively. More than one third (35.9%) physicians had the experiences of frontline working against COVID-19 infection. The 11,806 respondents consisted of 17.8, 51.2, and 20.9% of all physicians from category 1, 2, and 3 cities; consisted of 16.2, 31.1, and 52.7% of all from primary, secondary, and tertiary hospitals; and consisted of 78.3 and 21.7% of all from primary and specialized hospitals, respectively. Besides, 376 (3.2%) physicians were from Hubei Province. Table 1 shows the demographic characteristics of the participants.

**Table 1:** Demographics of the participants.

	Categories of cities^*^			**Levels of hospital**			**Natures of hospital**	
	**Category 1**	**Category 2**	**Category 3**	**Primary**	**Secondary**	**Tertiary**	**General**	**Specialized**
Age, mean (SD)	40.9 (9.2)	40.3 (9.0)	40.3 (8.9)	41.4 (9.1)	40.8 (9.0)	39.9 (9.0)	40.3 (9.0)	40.7 (9.0)
Female, %	92.6	91.7	89.6	93.8	93.2	89.2	91.0	92.1
Han Chinese, %	83.7	91.0	95.7	88.0	90.5	92.5	91.0	91.7
Married, %	86.6	85.6	84.5	87.1	86.5	84.3	85.3	86.1
**Degrees, %**
Master or doctor of medicine	10.4	21.9	43.0	6.5	13.8	39.9	29.4	15.5
Bachelor of medicine	72.7	66.9	51.6	70.9	72.8	55.2	62.2	66.6
Others	17.0	11.2	5.4	22.6	13.4	4.9	8.4	17.8
**Subspecialties, %**
Obstetrics	37.8	38.9	32.3	39.9	36.9	35.6	34.7	43.9
Gynecology	32.1	34.6	39.7	26.3	30.7	41.6	37.0	31.3
Others^†^	30.1	26.5	28.0	33.8	32.4	22.9	28.4	24.8
**Working years, %**
No more than 10 years	41.8	46.0	46.7	38.8	42.3	49.3	46.7	40.7
11–20 years	25.9	26.7	27.3	24.0	28.4	26.6	26.5	27.4
More than 20 years	32.3	27.4	26.0	37.2	29.3	24.1	26.7	31.9
**Professional title, %**
Chief doctor	29.1	32.6	31.0	28.1	30.1	33.4	32.5	27.8
Attending doctor	29.1	30.6	31.4	32.2	32.0	29.2	29.9	33.1
Resident doctor	21.7	21.7	20.8	20.2	20.4	22.4	22.1	19.1
Others^‡^	20.1	15.1	16.8	19.5	17.6	15.0	15.5	20.1

*The percentages were calculated from a sample of 11,806 participants. SD, standard deviation.*

* *The categories of cities were based on the numbers of total hospital beds. In the cities of category 1, 2 and 3, the numbers of total beds were <10,000, 10,000–30,000, and more than 30,000.*

† *Including physicians on reproductive medicine, family planning and no subspecialty*.

‡ *Including post-doctor and physicians refusing to report*.

### Impact of the COVID-19 Pandemic on Clinical Activities

**Figure 1:**
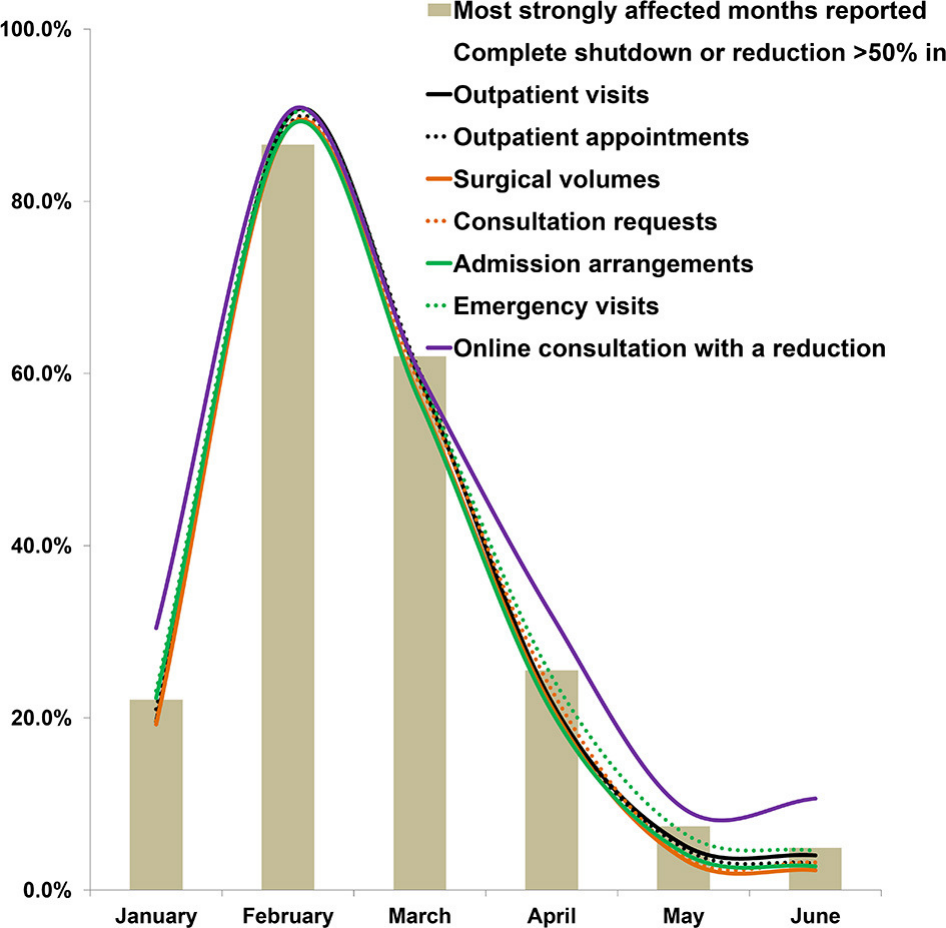
The periods most affected during the COVID-19 pandemic and clinical activities with a >50% reduction or a complete shutdown as reported by physicians. The ratios have been weighted.

With regard to the months during which their activities were the most strongly affected by the pandemic, 21.7, 87.1, 58.8, 21.7, 6.3, and 4.9% of the physicians chose January, February, March, April, May and June, respectively (Figure 1).

#### Changes of General Clinical Activities

As shown in Table 2, from January to June 2020, all clinical activities were reduced. Complete shutdown or a >50% reduction was reported to range from 45.1% (95% CI 43.2–47.0%) for outpatient visits to 20.8% (18.9–22.7%) for emergency visits. With regard to online consultations, 17.7% (95% CI 16.1–19.5%) and 51.6% (48.9–54.3%) of the physicians reported decreased and increased volumes, respectively. Except for emergency visits and online consultations, the proportions of activities with complete shutdowns or >50% reductions differed significantly according to various city categories, hospital levels and hospital natures (all *p* < 0.05, Supplementary Tables 1–3).

**Table 2:** Changes in clinical activities during the pandemic.

	**No. of participants (%)**	**During the imminent months of the pandemics (% [95% CI])**			**After July 1, 2020 (% [95% CI])**		
		**Complete shutdown or >50% reduction**	**Reduction by 25%-50%**	**Reduction <25% or no change**	**Less than 2019**	**Same as 2019**	**More than 2019**
Outpatient visits	9,673 (81.9%)	45.1 (43.2-47.0)	32.6 (31.2–34.1)	22.3 (20.7–24.0)	49.0 (46.6–51.5)	42.6 (40.4–44.9)	8.3 (7.6–9.2)
Outpatient appointments	7,519 (63.7%)	27.4 (25.5–29.5)	26.2 (24.9–27.6)	46.4 (44.3–48.5)	44.5 (42.2–46.8)	47.5 (45.4–49.5)	8.0 (7.3–8.9)
Surgical volumes	9,398 (79.6%)	30.2 (28.2–32.2)	30.5 (28.9–32.2)	39.3 (37.3–41.2)	49.1 (46.7–51.6)	42.9 (40.7–45.3)	7.9 (7.1–8.8)
Consultation requests	7,827 (66.3%)	22.3 (20.6–24.2)	18.3 (17.0–19.6)	59.4 (57.4–61.4)	39.7 (37.0–42.3)	53.7 (51.0–56.5)	6.6 (5.8–7.5)
Admission arrangements	9,180 (77.8%)	27.4 (25.1–29.7)	29.1 (27.3–31.0)	43.5 (41.4–45.7)	48.2 (45.8–50.7)	43.8 (41.6–45.9)	8.0 (7.1–9.0)
Emergency visits	6,763 (57.3%)	20.8 (18.9–22.7)	25.4 (23.3–27.7)	53.8 (51.2–56.3)	42.2 (39.5–45.0)	49.4 (46.7–52.1)	8.4 (7.5–9.4)
Online consultations^*^	5,231 (44.3%)	17.7 (16.1–19.5)	30.7 (28.7–32.7)	51.6 (48.9–54.3)	29.9 (28.1–31.9)	47.9 (45.4–50.4)	22.2 (20.5–24.0)

*95% CI, 95% confidence interval*.

* *The three percentage values during the imminent months of the pandemics denote decreasing, no change and increasing*.

As shown in Table 3, a multivariable regression analysis revealed that, with the exception of emergency visits and online consultations, category 3 cities, tertiary hospitals, and general hospitals experienced more reductions across broad areas of clinical activities compared with category 1 cities, primary hospitals, and specialized hospitals (all adjusted *p* < 0.05), respectively. However, after July 1, 2020, these differences disappeared. With regard to emergency visits and online consultations, differences in reductions only existed in the comparison of various city categories and hospital natures (adjusted *p* < 0.05).

**Table 3:** Relative risk ratios for the changes in clinical activities estimated from a multivariable regression model adjusted by municipal and hospital characteristics.

	**During the imminent months of the pandemics**				**After July 1, 2020**			
	**Complete shutdown or** **>50% reduction**		**Reduction by 25%−50%**		** <2019**		**More than 2019**	
	RRR^*^ (95% CI)	***p*-values**	RRR^*^ (95% CI)	***p*-values**	**RRR^†^ (95% CI)**	***p*-values**	**RRR^†^ (95% CI)**	***p*-values**
**Outpatient visits**								
**Categories of cities**								
Category 1	1.00		1.00		1.00		1.00	
Category 2	1.30 (1.00–1.69)	0.052	1.18 (0.95–1.47)	0.139	1.13 (0.90–1.42)	0.276	0.74 (0.60–0.92)	0.006
Category 3	1.39 (1.05–1.85)	0.024	1.30 (1.01–1.66)	0.038	1.29 (0.96–1.73)	0.093	0.57 (0.42–0.77)	<0.001
**Levels of hospitals**								
Primary	1.00		1.00		1.00		1.00	
Secondary	1.05 (0.81–1.37)	0.706	0.93 (0.74–1.16)	0.493	1.07 (0.84–1.37)	0.572	1.08 (0.80–1.47)	0.598
Tertiary	1.44 (1.04–1.99)	0.030	0.96 (0.75–1.23)	0.743	0.86 (0.62–1.20)	0.368	0.79 (0.57–1.09)	0.145
**Natures of hospitals**								
Specialized	1.00		1.00		1.00		1.00	
General	1.63 (1.16–2.28)	0.005	1.38 (1.11–1.71)	0.004	0.88 (0.69–1.13)	0.315	1.05 (0.78–1.40)	0.754
**Outpatient appointments**								
**Categories of cities**								
Category 1	1.00		1.00		1.00		1.00	
Category 2	1.34 (0.96–1.88)	0.084	1.04 (0.84–1.30)	0.716	1.09 (0.84–1.41)	0.508	0.70 (0.57–0.85)	0.001
Category 3	1.50 (1.04–2.16)	0.028	1.23 (0.98–1.54)	0.073	1.27 (0.93–1.74)	0.126	0.58 (0.45–0.74)	<0.001
**Levels of hospitals**								
Primary	1.00		1.00		1.00		1.00	
Secondary	1.09 (0.81–1.46)	0.565	0.95 (0.76–1.20)	0.686	1.02 (0.80–1.30)	0.864	1.44 (1.04–1.98)	0.029
Tertiary	1.63 (1.19–2.25)	0.003	1.01 (0.81–1.25)	0.957	0.75 (0.55–1.01)	0.061	0.99 (0.72–1.36)	0.953
**Natures of hospitals**								
Specialized	1.00		1.00		1.00		1.00	
General	1.56 (1.12–2.18)	0.01	1.43 (1.18–1.71)	<0.001	1.05 (0.82–1.34)	0.694	1.08 (0.85–1.37)	0.529
**Surgical volumes**								
**Categories of cities**								
Category 1	1.00		1.00		1.00		1.00	
Category 2	1.20 (0.83–1.72)	0.325	1.26 (0.92–1.72)	0.145	1.01 (0.80–1.28)	0.904	0.79 (0.63–0.99)	0.045
Category 3	1.55 (1.06–2.25)	0.023	1.38 (1.02–1.86)	0.037	1.21 (0.89–1.65)	0.22	0.66 (0.46–0.95)	0.025
**Levels of hospitals**								
Primary	1.00		1.00		1.00		1.00	
Secondary	1.06 (0.79–1.40)	0.706	1.02 (0.81–1.29)	0.861	1.03 (0.76–1.41)	0.828	1.46 (1.08–1.97)	0.013
Tertiary	1.40 (1.03–1.92)	0.034	0.94 (0.74–1.19)	0.583	0.78 (0.55–1.12)	0.174	1.01 (0.74–1.37)	0.972
**Natures of hospitals**								
Specialized	1.00		1.00		1.00		1.00	
General	2.18 (1.47–3.23)	<0.001	1.63 (1.33–2.00)	<0.001	0.94 (0.73–1.21)	0.612	0.85 (0.67–1.08)	0.19
**Consultation requests**								
Categories of cities
Category 1	1.00		1.00		1.00		1.00	
Category 2	1.24 (0.90–1.69)	0.183	0.90 (0.68–1.20)	0.466	0.95 (0.73–1.23)	0.687	0.64 (0.51–0.80)	<0.001
Category 3	1.56 (1.12–2.17)	0.01	1.07 (0.80–1.44)	0.648	1.06 (0.77–1.48)	0.709	0.47 (0.31–0.71)	<0.001
**Levels of hospitals**								
Primary	1.00		1.00		1.00		1.00	
Secondary	1.30 (0.98–1.71)	0.068	1.29 (1.02–1.62)	0.032	0.99 (0.75–1.30)	0.93	1.22 (0.88–1.67)	0.227
Tertiary	1.91 (1.44–2.55)	<0.001	1.39 (1.11–1.73)	0.004	0.86 (0.59–1.24)	0.405	1.06 (0.75–1.48)	0.744
**Natures of hospitals**								
Specialized	1.00		1.00		1.00		1.00	
General	1.69 (1.21–2.35)	0.002	1.40 (1.15–1.71)	0.001	0.88 (0.65–1.19)	0.393	0.98 (0.72–1.32)	0.877
**Admission arrangements**								
**Categories of cities**								
Category 1	1.00		1.00		1.00		1.00	
Category 2	1.26 (0.91–1.76)	0.166	1.19 (0.89–1.58)	0.234	1.09 (0.87–1.36)	0.47	0.66 (0.51–0.85)	0.002
Category 3	1.64 (1.15–2.33)	0.006	1.24 (0.90–1.70)	0.186	1.20 (0.89–1.63)	0.232	0.51 (0.36–0.72)	<0.001
**Levels of hospitals**								
Primary	1.00		1.00		1.00		1.00	
Secondary	1.13 (0.81–1.56)	0.475	1.00 (0.77–1.30)	0.998	1.06 (0.77–1.44)	0.729	1.29 (0.95–1.74)	0.099
Tertiary	1.63 (1.14–2.34)	0.008	0.98 (0.75–1.29)	0.901	0.80 (0.57–1.14)	0.218	0.90 (0.64–1.26)	0.524
**Natures of hospitals**								
Specialized	1.00		1.00		1.00		1.00	
General	1.86 (1.22–2.82)	0.004	1.43 (1.16–1.76)	0.001	1.04 (0.80–1.34)	0.776	1.07 (0.80–1.44)	0.636
**Emergency visits**								
**Categories of cities**								
Category 1	1.00		1.00		1.00		1.00	
Category 2	1.12 (0.82–1.53)	0.461	1.03 (0.79–1.34)	0.824	1.02 (0.81–1.28)	0.893	0.85 (0.66–1.08)	0.179
Category 3	1.36 (0.95–1.96)	0.091	1.16 (0.85–1.60)	0.34	1.05 (0.74–1.47)	0.798	0.60 (0.44–0.83)	0.002
**Levels of hospitals**								
Primary	1.00		1.00		1.00		1.00	
Secondary	0.96 (0.69–1.33)	0.814	0.87 (0.68–1.10)	0.243	0.86 (0.66–1.11)	0.232	1.16 (0.81–1.68)	0.416
Tertiary	1.14 (0.87–1.49)	0.342	0.97 (0.77–1.21)	0.769	0.68 (0.51–0.91)	0.011	0.81 (0.56–1.16)	0.249
**Natures of hospitals**								
Specialized	1.00		1.00		1.00		1.00	
General	1.82 (1.36–2.43)	<0.001	1.39 (1.15–1.68)	0.001	1.06 (0.86–1.31)	0.569	1.10 (0.80–1.52)	0.551
**Online consultations** ^**‡**^								
**Categories of cities**								
Category 1	1.00		1.00		1.00		1.00	
Category 2	0.89 (0.67–1.19)	0.425	0.68 (0.54–0.86)	0.001	0.92 (0.71–1.20)	0.552	0.91 (0.71–1.16)	0.445
Category 3	1.06 (0.74–1.50)	0.764	0.59 (0.44–0.79)	<0.001	1.07 (0.79–1.45)	0.68	1.20 (0.90–1.62)	0.215
**Levels of hospitals**								
Primary	1.00		1.00		1.00		1.00	
Secondary	0.87 (0.61–1.24)	0.445	0.95 (0.74–1.22)	0.693	0.78 (0.56–1.08)	0.128	1.37 (0.99–1.90)	0.058
Tertiary	0.81 (0.59–1.12)	0.202	0.83 (0.65–1.06)	0.134	0.67 (0.49–0.91)	0.012	0.94 (0.69–1.29)	0.702
**Natures of hospitals**								
Specialized	1.00		1.00		1.00		1.00	
General	1.38 (0.94–2.03)	0.098	1.10 (0.92–1.33)	0.298	1.08 (0.81–1.44)	0.593	1.06 (0.83–1.36)	0.637

*The categories of cities were based on the total number of hospital beds. In categories 1, 2 and 3 cities, the total numbers of beds were fewer than 10,000; 10,000–30,000; and more than 30,000, respectively. 95% CI, 95% confidence interval;. RRR, relative risk ratio.*

* *With the response of “reduction <25% or no change” as reference.*

† *With the response of “same as 2019” as reference*.

‡ *The two percentage values during the imminent months of the pandemics denote no change and increasing, with the response of “reduction” as reference*.

With the exception of outpatient visits and online consultations, physicians from Hubei Province and physicians from other provinces did not report any significant differences in complete shutdowns or >50% reductions (all *p* > 0.05, Supplementary Table 4). Significantly higher proportions of physicians reported reduction in online consultations (*p* = 0.015) and complete shutdowns or >50% reductions in outpatient visits (*p* = 0.003) from Hubei Province than physicians from other provinces.

#### Changes of Management of Specific Diseases or Conditions

As shown in Table 4, treatments for specific diseases or conditions decreased in parallel with the changes in general clinical activities. From the 11,806 respondents, the proportion of physicians reporting a complete shutdown or a >50% reduction ranged from 38.0% (35.4–40.6%) for assisted reproduction to 15.8% (95% CI 13.9–18.0%) for emergency obstetrical surgeries. However, unlike general clinical activities, disparities existed according to municipal and hospital characteristics (Supplementary Tables 5–7). The treatment and follow-up of malignancies did not significantly differ based on various municipal or hospital characteristics (all *p* > 0.05). Less urgent issues, including assisted reproduction, cervical cancer screening, instructions regarding contraception and miscarriage, and treatment for benign neoplasms or for pelvic floor dysfunctions, differed significantly across municipal or hospital levels and natures (all *p* < 0.05). Compared with other provinces, in Hubei Province, all clinical activities for specific conditions or diseases significantly decreased (all *p* < 0.05, Supplementary Table 8).

**Table 4:** Changes in the management of specific diseases or conditions.

	**No. of participants (%)**	**Complete shutdown or >50% reduction, (% [95% CI])**	**Reduction by 25%−50%, (% [95% CI])**	**Reduction <25% or no change, (% [95% CI])**
Preconception counseling	8,241 (69.8%)	34.3 (32.7–36.0)	31.7 (30.2–33.1)	34.0 (32.4–35.6)
Prenatal examinations	8,218 (69.6%)	26.9 (25.1–28.9)	32.2 (30.5–34.0)	40.8 (39.2–42.5)
Prenatal diagnosis	6,919 (58.6%)	27.6 (25.8–29.4)	28.0 (26.6–29.5)	44.4 (42.5–46.4)
Instructions for contraception and miscarriage	8,419 (71.3%)	29.0 (27.2–30.8)	23.4 (22.1–24.8)	47.6 (45.9–49.3)
Assistant reproduction	3,871 (32.8%)	38.0 (35.4–40.6)	21.5 (19.7–23.5)	40.5 (38.1–42.9)
Outpatient surgeries and procedures	7,196 (61.0%)	35.4 (33.6–37.4)	26.7 (25.1–28.3)	37.9 (36.0–39.8)
Emergent obstetrical surgeries	7,542 (63.9%)	15.8 (13.9–18.0)	20.7 (19.2–22.4)	63.4 (61.1–65.7)
Emergent gynecological surgeries	7,575 (64.2%)	21.0 (18.9–23.3)	20.3 (18.7–21.9)	58.8 (56.7–60.8)
Cervical cancer screening	7,434 (63.0%)	37.3 (35.4–39.2)	22.0 (20.6–23.4)	40.7 (39.0–42.5)
Treatment for benign neoplasm	7,278 (61.6%)	35.7 (33.7–37.7)	24.7 (23.0–26.5)	39.6 (37.9–41.4)
Treatment for malignancies	6,625 (56.1%)	25.8 (23.7–28.1)	20.1 (18.9–21.4)	54.0 (51.7–56.3)
Follow–up for malignancies	6,492 (55.0%)	26.5 (24.6–28.6)	19.6 (18.3–20.9)	53.9 (51.8–55.9)
Treatment for pelvic floor dysfunctions	6,551 (55.5%)	39.5 (37.9–41.1)	21.7 (20.3–23.1)	38.9 (37.0–40.7)

*95% CI, 95% confidence interval*.

## Discussion

This was the first nationally representative survey of physicians describing the impact of the COVID-19 pandemic on clinical activities in China. In this national survey including Chinese obstetricians and gynecologists, all clinical activities except online consultations substantially decreased. Our findings provided a specific description and sceneria of the national reflection toward COVID pandemic in a medical speciality caring the health of the women and children. The data from our survey could offer a substantial basis for the discussion and reformation of health system coping with the global outbreak and persistence of severe pandemic. In our survey, cities with more hospital beds, hospitals with better resources, and general hospitals were more severely affected with regard to most clinical activities. There are several explanations for these differences. Larger, densely populated cities have a greater risk of infection; therefore, the general clinical activities were more severely impacted in these cities due to lockdown. Larger hospitals and general hospitals undertook the more pressing tasks of testing and caring for patients who had contracted COVID-19 than smaller hospitals and specialized hospitals for women health. In such conditions, medical staff and resources were significantly shifted to other priorities as an emergency measure spontaneously or according to the administrative regulations.

However, the need to shift resources and personnel to cope with an emerging crisis does not mean that the shift remains indefinitely sustainable (21). It is important to evaluate whether and how much this shift has exacerbated existing health inequities and to be proactive in creating policies that promote equity (22). A reform to create a more balanced, healthy medical service system may be warranted, and steps need to be taken after the pandemic to minimize the delay in routine care for women. In our study, cervical cancer screening and instructions regarding contraception and miscarriage had more significant reductions in cities with more hospital beds and in higher-level hospitals. These changes should be noted. With regard to cervical cancer screening, health professionals should focus on high-risk women and adhere to cost-effective policies, including self-sampling in the immediate postepidemic phase (23). The reduction in attention paid to contraception and miscarriage in large cities or high-level hospitals may reflect a substantial bias with regard to such topics (24), since the shutdown of or delays in contraception and safe abortion during COVID-19 will disproportionately impact the most vulnerable populations in low-income and middle-income regions and countries and lead to considerable increases in preventable mortality and lifelong disability (25).

Our survey provided insight into the management of specific diseases and conditions, including emergencies and less urgent medical issues. According to the survey, the changes in emergency visits, including changes in emergency gynecological or obstetrical surgeries, differed significantly between general and specialized hospitals. Although numerous reports on COVID-19 exist, only a few discussed the impact of COVID-19 pandemic on clinical activities. We used keywords of “clinical activity,” “COVID-19,” and “impact” had a search in clinical trials and reviews published in English in PubMed (https://pubmed.ncbi.nlm.nih.gov/), only 260 papers were available up to July, 2021. The COVID-19 lockdown substantially reduced admission to gynecological emergency departments, but triage allowed the separation of real emergencies from more deferrable emergencies (8), such as emergency surgeries (26). On the other hand, only less urgent or critical medical issues, including assisted reproduction, differed significantly according to the levels and characteristics of the cities and hospitals. While these services were temporarily disrupted, new strategies are needed to overcome these changes. It is essential for authorities and health care providers to identify patients who should be prioritized for the continuation of fertility care in a safe environment (27). Many guidelines or protocols are available to support prioritization in the field of obstetrics and gynecology (28, 29), and they should be considered on the basis of local resources and planning (30). In our study, we did not find significant differences in the treatment of gynecological malignancies according to city categories, hospital levels or hospital natures, which reflected the attention paid to these critical diseases across the country.

Our survey highlighted feasible innovative treatment strategies during the pandemic. According to the WHO report (15), telemedicine is currently one of the mitigation strategies most often used (27). As previously reported (31), and as expected during the design of the questionnaire, online consultations increased by 51.6% in our survey. The pandemic afforded ambulatory clinicians with the opportunity to expand care to vulnerable populations in ways that were previously underutilized, thus improving health equity (32) by adopting the necessary regulatory framework for the wide application of telemedicine (33). However, telemedicine has its own limitations with regard to examinations and procedures necessary for the diagnosis and treatment of gynecological and obstetrical diseases (34, 35). The quality and trustworthiness of social media are also questionable (36). Legal issues pertaining to telemedicine have yet to be resolved in China (37). Last, in our study, little evidences suggested telemedicine would provide a sufficient and satisfactory solution for the lack of direct clinical interviews during pandemic lockdown. A more exhaustive survey would prudently translate the changes of tendency in medical service into specific, quantified clinical activities, such as outpatient's visits, medication, and examination. However, in our study, in order to quantize the impact, we must include a lot more respondents as to decrease the greater bias caused by epidemiological and personal characteristics. In conclusion, as no study could forward direct evidences discovering and resolving the gaps between telemedicine and face-to-face interviews, we must keep discreet optimism toward the prosperity of telemedicine.

Our survey revealed critical differences in the changes in medical services among various regions of different situations with respect to the pandemic. The comparison between Hubei Province and other provinces in China suggested that general clinical activities did not significantly decrease in Hubei; however, the management of all specific gynecological or obstetrical conditions declined significantly. These differences suggested the shift of medical sources to cope with COVID-19, including new assignments for obstetricians and gynecologists, since 80.2% of confirmed cases in China occurred in Hubei Province.

The large nationally representative sample and a comprehensive assessment of the impact on clinical activities were the strengths of our study. Specifically, our results revealed that COVID-19 pandemic had significantly different impact on the clinical activities across various municipal and hospital characteristics. However, there are several limitations in our study. We did not include private health services in the survey since they account for a very limited proportion of the total volume of the healthcare market in China. This study did not explore the impact of the pandemic on the lives, professional careers, and mental health of obstetricians and gynecologists, although many reports have discussed the stress experienced by these clinicians (4, 38), which may be associated with changes of medical service during the pandemic (39). Physicians' reported qualitative assessment about changes in clinical activities lacks of uniform evaluation, which should be supported by more data from national statistical data. However, although personal feeling had its limitation of clear description, it indeed reflected an invaluable experience in caring their patients.

One of the most important limitations is that we did not consider the national and/or local policies and interventions in this study. The national policies would have played a great role of resumption of medical service and social economics. Just like the situations in other society activities, a temporal trend in the decrease or increase would be most strongly affected by the restrictions, measures of dealing with the pandemics by federal and local governments and organizations (40, 41). A successful control of COVID-19 pandemics depends on the unselfish devotion of the healthcare staffs, comprehensive society movement against pandemics, and national decisions and policies. Although widespread gaps in the quality of primary health care still exist in China (42), a series effective, rapid measure have been implemented to tackle the disease (43). The most serious outbreaks occurred in February and March, 2020, and accordingly, the most rigorous restrictions from personal, organizational and national requirements were performed (44–47). These restrictions, undoubtedly, would cause great changes in clinical activities. It is regret that we couldn't quantitatively take these changes in this analysis. However, since all the provinces and hospitals in China were under a series of relative consistent policies, changes among these administrations and different diseases have their significances in coping with COVID-19 pandemics. Authors' clinical experiences during the COVID-19 pandemics accorded with the trends discovered in this study. In the principle investigational hospital, one of the teaching tertiary hospitals, in February and March of 2020, only less half outpatient and inpatient workload was required for gynecologic services.

## Conclusions

In this national, stratified, two-stage, random cluster sampling survey, clinical activities in obstetrics and gynecology were majorly reduced during the COVID-19 pandemic in China. Cities and hospitals with more resources or general hospitals were more severely affected, resulting in delays or other disparities in the medical care of vulnerable populations, such as women needing cancer screening or assisted reproduction. However, the magnitudes of the decline varied among other specific diseases or conditions.

## Data Availability Statement

The raw data supporting the conclusions of this article will be made available by the authors, without undue reservation.

## Ethics Statement

The studies involving human participants were reviewed and approved by Institutional Review Board of Peking Union Medical College Hospital. Written informed consent for participation was not required for this study in accordance with the national legislation and the institutional requirements.

## Author Contributions

LZ, HZ, and JLv conceived of the original idea for the study, interpreted results, carried out the statistical analysis, edited the paper, and were overall guarantor. LL obtained ethical approval, contributed to the preparation of the data set, interpreted results, and contributed to drafts of the paper. YC, JF, TL, and JLa contributed to the study design, data collection, interpretation of results, and commented on drafts of the paper. All authors contributed to the article and approved the submitted version.

## Conflict of Interest

The authors declare that the research was conducted in the absence of any commercial or financial relationships that could be construed as a potential conflict of interest.

## Publisher's Note

All claims expressed in this article are solely those of the authors and do not necessarily represent those of their affiliated organizations, or those of the publisher, the editors and the reviewers. Any product that may be evaluated in this article, or claim that may be made by its manufacturer, is not guaranteed or endorsed by the publisher.
