# Supplementary material for: Impact of COVID-19 Pandemic on the Clinical Activities in Obstetrics and Gynecology: A National Survey in China
Source: Front Med (Lausanne). 2021 Jul 30;8:633477. doi: 10.3389/fmed.2021.633477 (PMC8360866; doi:10.3389/fmed.2021.633477)
Supplement: Supplementary file 2 [file Data_Sheet_2.DOCX]

**Supplementary Materials**

[Members from Chinese Medical Doctor Association 2](#_Toc56692992)

[Supplement 1: Sampling methods and results. 10](#_Toc56692993)

[Supplement 2: The health economics of all cities included in the random sampling. 16](#_Toc56692994)

[Supplement 3: Cities categorized according to the total bed numbers in each province. 28](#_Toc56692995)

[Supplement 4: Hospitals grouped according to various levels in each city. 29](#_Toc56692996)

[Supplement 5: The response rates in various groups 33](#_Toc56692997)

[Supplement 6: The results of the weighting analysis 64](#_Toc56692998)

[Supplement 7: Questionnaire design 107](#_Toc56692999)

[Supplement 8: Hospital codes in this study 111](#_Toc56693000)

[References 130](#_Toc56693001)

[Legend of supplementary tables 131](#_Toc56693002)

[Table S1 133](#_Toc56693003)

[Table S2 135](#_Toc56693004)

[Table S3 137](#_Toc56693005)

[Table S4 139](#_Toc56693006)

[Table S5 141](#_Toc56693007)

[Table S6 145](#_Toc56693008)

[Table S7 148](#_Toc56693009)

[Table S8 151](#_Toc56693010)

# Members from Chinese Medical Doctor Association

Bianbazhuoma, M.D.

Department of Obstetrics and Gynecology, The Municipal People’s Hospital of Lhasa, Lhasa 850000, China

Email: 18908910688@163.com

Liehong Wang, M.D.

Department of Obstetrics and Gynecology, Qinghai Red Cross Hospital, Xining 810000, China

Email: doctorwlh@126.com

Chunfang Ha, M.D.

Department of Gynecology, General Hospital of Ningxia Medical University, Yinchuan 750004, China

Email: hachunfang@163.com

Fengxia Xue, M.D.

Department of Obstetrics and Gynecology, Tianjin Medical University General Hospital, Tianjin 300052, China

Email: fengxiaxue1962@163.com

Yuan Sun, M.D.

Department of Obstetrics and Gynecology, Maternity and Child Health Hospital of Guizhou Province, Guiyang 550002, China

Email: 2641580736@qq.com

Genhai Zhu, M.D.

Department of Obstetrics and Gynecology, Hainan People’s Hospital, Haikou 570000, China

Email: genhaizhu@163.com

Liqun Wang, M.D.

Department of Obstetrics and Gynecology, Jiangxi Provincial Maternal and Child Health Hospital, Nanchang 330006, China

Email: 1500325038@qq.com

Ruifang An, M.D.

Department of Obstetrics and Gynecology, The First Affiliated Hospital of Xi'an Jiaotong University, Xi'an 710061, China

Email: anruifang@163.com

Wen Di, M.D.

Department of Obstetrics and Gynecology, Renji Hospital, School of Medicine, Shanghai Jiao Tong University, Shanghai 200127, China

Email: diwen163@163.com

Jianliu Wang, M.D.

Department of Obstetrics and Gynecology, Peking University People's Hospital, Beijing 100044, China

Email: wangjianliu@pkuph.edu.cn

Meisong Lu, M.D.

Department of Obstetrics and Gynecology, The First Affiliated Hospital of Harbin Medical University, Harbin 150001, China

Email: lumeisong0417@163.com

Min Hao, M.D.

Department of Obstetrics and Gynecology, The Second Hospital of Shanxi Medical University, Taiyuan 030000, China

Email: 2Yuanhaomin@163.com

Jun Lin, M.D.

Department of Obstetrics and Gynecology, Women's Hospital, School of Medicine, Zhejiang University, Hangzhou 310000, China

Email: linjun@zju.edu.cn

Yan Ding, M.D.

Department of Gynecology, The First Affiliated Hospital of Xinjiang Medical University, Urumchi 830000, China

Email: ddy8930@126.com

Xianghua Huang, M.D.

Department of Obstetrics and Gynecology, The Second Hospital of Hebei Medical University, Shijiazhuang 050000, China

Email: huangxh2003@163.com

Guonan Zhang, M.D.

Department of Gynecologic Oncology, Sichuan Cancer Hospital & Institute, Sichuan Cancer Center, Cancer Hospital Affiliated to School of Medicine, University of Electronic Science and Technology of China, Chengdu 610041, China

Email: zhanggn@hotmail.com

Wenjun Cheng, M.D.

Department of Gynecology, Jiangsu Province Hospital, The First Affiliated Hospital of Nanjing Medical University, Nanjing 210029, China

Email: chengwenjundic@163.com

Yongxiu Yang, M.D.

Department of Obstetrics and Gynecology, The First Hospital of Lanzhou University, Lanzhou 730000, China

Email: yongxiuyang@163.com

Manhua Cui, M.D.

Department of Obstetrics and Gynecology, The Second Hospital of Jilin University, Changchun 130041, China

Email: cuimanhua@126.com

Shijie Yan, M.D.

Department of Obstetrics and Gynecology, The First Affiliated Hospital of Anhui Medical University, Hefei 230022, China

Email: ysj5851@126.com

Renfeng Zhao, M.D.

Department of Obstetrics and Gynecology, People's Hospital of Guangxi Zhuang Autonomous Region, Nanning 530021, China

Email: gxzhaorenfeng@163.com

Min Xue, M.D.

Department of Obstetrics and Gynecology, The Third Xiangya Hospital of Central South University, Changsha 410013, China

Email: xuemin5908@sina.com

Yiling Ding, M.D.

Department of Obstetrics and Gynecology, The Second Xiangya Hospital of Central South University, Changsha 410011, China

Email: dylcsu@sina.cn

Jinghui Song, M.D.

Department of Obstetrics and Gynecology, Affiliated Hospital of Inner Mongolia Medical University, Huhhot 010059, China

Email: songjinghui2002@aliyun.com

Lin Xu, M.D.

Department of Gynecology, Kunming Tongren Hospital, Kunming 650000, China

Email: xulin64@163.com

Zehua Wang, M.D.

Department of Obstetrics and Gynecology, Union Hospital, Tongji Medical College, Huazhong University of Science and Technology, Wuhan 430022, China

Email: zehuawang@163.net

Jie Chen, M.D.

Department of Obstetrics and Gynecology, Fujian Provincial People's Hospital, Fuzhou 350000, China

Email: 1073057290@qq.com

Qing Yang, M.D.

Department of Obstetrics and Gynecology, Shengjing Hospital of China Medical University, Shenyang 110004, China

Email: yangq@sj-hospital.org

Zhiqing Liang, M.D.

Department of Obstetrics and Gynecology, Southwestern Hospital, Third Military Medical University, Chongqing 400038, China

Email: zhi_lzliang@yahoo.com

Dongzi Yang, M.D.

Department of Obstetrics and Gynecology, Sun Yat-sen Memorial Hospital, Sun Yat-sen University, Guangzhou 510120, China

Email: yangdz@mail.sysu.edu.cn

Liuxia Li, M.D.

Department of Obstetrics and Gynecology, The First Affiliated Hospital of Zhengzhou University, Zhengzhou 450052, China

Email: llxia698@163.com

Beihua Kong, M.D.

Department of Obstetrics and Gynecology, Qilu hospital of Shandong University, Jinan 250012, China

Email: kongbeihua@sdu.edu.cn

# Supplement 1: Sampling methods and results.

1. Sampling design

(1) A survey was conducted on 304 prefecture-level cities in 31 provincial administrative regions, consisting of 22 provinces, 5 autonomous regions, and 4 municipalities.

- The 22 provinces were: Hebei Province, Shanxi Province, Liaoning Province, Jilin Province, Heilongjiang Province, Jiangsu Province, Zhejiang Province, Anhui Province, Fujian Province, Jiangxi Province, Shandong Province, Henan Province, Hubei Province, Hunan Province, Guangdong Province, Hainan Province, Sichuan Province, Guizhou Province, Yunnan Province, Shaanxi Province, Gansu Province, and Qinghai Province.
- The five autonomous regions were: Inner Mongolia Autonomous Region, Guangxi Zhuang Autonomous Region, Tibet Autonomous Region, Ningxia Hui Autonomous Region, and Xinjiang Uygur Autonomous Region.
- The four municipalities directly under the Central Government were: Beijing, Tianjin, Shanghai, and Chongqing.

(2) The prefecture-level cities were divided into 3 categories according to the number of hospital beds in the city. Cities with less than 10,000 beds belonged to Category 1, cities with 10,000-29999 beds belonged to Category 2, and cities with ≥30,000 beds belonged to the Category 3 (Supplement 2). Cities were randomly selected from each category using a selection ratio of 1:2 or 1:3, based on the city numbers in each province.

(3) In the selected cities, hospitals were stratified into 3 layers based on a 3-tier system, the primary, secondary, and tertiary hospitals. Two hospitals were randomly selected from each layer, however, there were probably not enough hospitals in some layers to sample in some cities, and only one hospital was surveyed. The Tibet Autonomous Region lacked enough tertiary hospitals for sampling. Qinghai Province lacked enough tertiary hospitals for sampling except for Xining City, so more tertiary hospitals in Xining City were surveyed as supplements to other cities. In the municipalities, twelve hospitals were randomly selected from each layer, since the whole municipality city is regarded as one city.

(4) In the selected hospitals, all obstetrician-gynecologists were to answer the questionnaire.

- Primary hospital: 31 provinces × 6 cities × 2 hospitals × all obstetrician-gynecologists in the hospital
- Secondary hospital: 31 provinces × 6 cities × 2 hospitals × all obstetrician-gynecologists in the hospital
- Tertiary hospital: 31 provinces × 6 cities × 2 hospitals × all obstetrician-gynecologists in the hospital

2. Data preparation

1. Stratification Variables

- Provincial administrative regions: 31
- Prefecture-level cities divided into 3 layers according to the number of hospital beds in the city: 3
- Hospitals stratified based on a 3-tier system (primary, secondary, and tertiary hospitals): 3
- The above three variables were grouped together to generate a stratification variable. Theoretically there should be 279 layers (31×3×3=279), the actual stratifications were 190 layers, and the final stratifications obtained were 183 layers.

(2) Clusters

- Primary sampling units (PSUs): regional variables
- Secondary sampling units (SSUs): hospital variables

(3) FPC correction (finite population correction)

- The FPC variable sampled in the first stage: the total number of cities in each layer stratified by the number of hospital beds in each province (FPC variable 1: the number of cities in the province, Supplement 3)
- The FPC variable of the second stage sampling: the total number of primary, secondary and tertiary hospitals of each selected city (FPC variable 2: the number of hospitals in the city, Supplement 4)

(4) Sample weights:

- - Base weights

$$Sampling probability 1=\frac{the number of cities selected from each layer stratified by the number of hospital beds in each province}{the total number of cities in the corresponding layer}$$

$$Sampling probability 2 =\frac{the number of primary, secondary and tertiary hospitals selected from each selected city}{the total number of hospitals in the corresponding layer}$$

$$The probability of each doctor being selected=the probability of the hospital being selected =sampling probability 1 \times sampling probability 2$$

$$The weight of each doctor=\frac{1}{the probability of each doctor being selected}$$

(*All doctors in each selected hospital were given the same weight)

- Nonresponse adjustments

$$Hospital response rate =\frac{the actual number of responding primary, secondary, and tertiary hospitals in each selected city}{the total number of hospitals selected in the corresponding layer*}$$

(*Among the selected hospitals, some did not exist after restructuring, and some had no obstetrician-gynecologists. These hospitals were excluded from the denominator.)

$$Individual response rate=\frac{the actual number of respondents in each hospital}{the total number of OB/GYNs in each selected hospital}$$

$$Total response rate=hospital response rate\times individual response rate$$

$$Nonresponse adjustments=\frac{1}{total response rate}$$

- Poststratification adjustments

$$poststratification adjustments=\frac{the actual number of OB/GYNs in a certain layer/the total number of OB/GYNs in the country}{the number of respondents in a certain layer/the total number of OB/GYNs surveyed nationwide}$$

Theoretically, the poststratification adjustments should be calculated on the basis of 279 (31×3×3=279) layers. However, if it was impossible to actually know the proportion of each layer, it could only be simplified to, for example, calculating the adjustment factors separately using a combined stratification according to the number of hospital beds in the city and the primary, secondary, and tertiary hospital system (3×3). All physicians in the corresponding layer were assigned the same adjustment factor.

- Total weight

$$The total weight of each doctor surveyed=base weights\times nonresponse adjustments\times poststratification adjustments$$

3. Sampling and responding results

The sampling and responding results were listed in Supplement 5 and Supplement 6, respectively. The detailed responding information was summarized as followings.

- Three hundred sixty-seven primary hospitals were selected and 253 responded. Among the 114 hospitals that did not respond, twenty-two did not exist after the restructuring, twenty-four had no obstetrician-gynecologist, one was repeatedly selected, and 67 hospitals refused to participate. The response rate was 79.1% (367-22-24-1=320; 253/320=79.1%).
- Three hundred sixty-six secondary hospitals were selected and 284 responded. Among the 82 hospitals that did not respond, sixteen did not exist after the restructuring, three had no obstetrician-gynecologists, two were repeatedly selected, and 61 hospitals refused to participate. The response rate was 82.3% (366-16-3-2=345; 284/345=82.3%).
- Two hundred ninety four tertiary hospitals were selected and 242 responded. Among the 52 hospitals that did not respond, five did not exist after the restructuring, one had no obstetrician-gynecologists, four were repeatedly selected, and 42 hospitals refused to participate. The response rate was 85.2% (294-5-1-4=284; 242/284=85.2%).
- Seven hundred seventy-nine hospitals responded to the questionnaire survey, in which 630 hospitals had a response rate of over 80.0 percent.
- There were 155,787 registered obstetrician-gynecologists in China nationwide, in whom 12,592 were sampled and 11,806 completed the questionnaire survey (response rate 93.8%), who accounted for 7.6% of all registered obstetrician-gynecologists.

# Supplement 2: The health economics of all cities included in the random sampling.

| **Provinces** | **City** | **City population (thousand)** | **City GDP (thousand RMB)** | **City hospital numbers** | **City hospital beds** | **City (assistant) physicians** |
| --- | --- | --- | --- | --- | --- | --- |
| Anhui | Anqing | 5300 | 191758500 | 73 | 16773 | 8880 |
| Anhui | Bengbu | 3830 | 171465600 | 81 | 17101 | 7219 |
| Anhui | Bozhou | 6540 | 127719450 | 78 | 14250 | 7198 |
| Anhui | Chizhou | 1620 | 68492530 | 33 | 5913 | 3001 |
| Anhui | Chuzhou | 4540 | 180174960 | 70 | 14419 | 6840 |
| Anhui | Fuyang | 10700 | 175951990 | 120 | 32749 | 15403 |
| Anhui | Hefei | 7500 | 782290610 | 171 | 48042 | 22320 |
| Anhui | Huaibei | 2170 | 98519020 | 70 | 9252 | 4603 |
| Anhui | Huainan | 3900 | 113330640 | 78 | 14470 | 6229 |
| Anhui | Huangshan | 1490 | 67791430 | 32 | 6735 | 3440 |
| Anhui | Lu'an | 5880 | 128805380 | 32 | 12874 | 9261 |
| Anhui | Maanshan | 2290 | 191810000 | 63 | 7922 | 5045 |
| Anhui | Suzhou | 6560 | 163022170 | 81 | 16323 | 9406 |
| Anhui | Tongling | 1710 | 122235960 | 30 | 7815 | 3546 |
| Anhui | Wuhu | 3880 | 327853170 | 81 | 18133 | 8885 |
| Anhui | Xuanchcng | 2800 | 131720470 | 47 | 10824 | 5506 |
| Beijing | Beijing | 13680 | 3031997870 | 736 | 116279 | 109376 |
| Chongqing | Chongqing | 33970 | 2036319000 | 800 | 162147 | 76361 |
| Fujian | Fuzhou | 6980 | 785681230 | 128 | 32569 | 22413 |
| Fujian | Longyan | 3180 | 239329510 | 46 | 17381 | 6364 |
| Fujian | Nanping | 3200 | 179251300 | 53 | 11965 | 5715 |
| Fujian | Ningde | 3530 | 194279510 | 46 | 11027 | 5786 |
| Fujian | Putian | 3570 | 224241340 | 49 | 11299 | 5801 |
| Fujian | Quanzhou | 7490 | 846797680 | 131 | 26596 | 17114 |
| Fujian | Sanming | 2880 | 235371620 | 53 | 11014 | 5945 |
| Fujian | Xiamen | 2370 | 479141310 | 54 | 15342 | 13830 |
| Fujian | Zhangzhou | 5180 | 394763470 | 82 | 18571 | 11241 |
| Gansu | Baiyin | 1820 | 51159680 | 99 | 8067 | 3603 |
| Gansu | Dingxi | 3040 | 35626090 | 48 | 12185 | 5133 |
| Gansu | Jiayuguan | 210 | 29962000 | 9 | 1663 | 993 |
| Gansu | Jinchang | 470 | 26424050 | 15 | 2727 | 1375 |
| Gansu | Jiuquan | 990 | 59688710 | 36 | 5410 | 3629 |
| Gansu | Lanzhou | 3270 | 273293730 | 125 | 26886 | 13954 |
| Gansu | Longnan | 2880 | 37922730 | 61 | 7977 | 3749 |
| Gansu | Pingliang | 2340 | 39516510 | 149 | 12490 | 4594 |
| Gansu | Qingyang | 2700 | 70814990 | 44 | 9619 | 4773 |
| Gansu | Tianshui | 3710 | 65205420 | 77 | 12438 | 5484 |
| Gansu | Wuwei | 1900 | 46926530 | 13 | 8310 | 4038 |
| Gansu | Zhangye | 1310 | 40770670 | 45 | 6109 | 3528 |
| Guangdong | Chaozhou | 2760 | 106727900 | 32 | 4919 | 4597 |
| Guangdong | Dongguan | 2210 | 827859200 | 102 | 30239 | 19516 |
| Guangdong | Foshan | 4280 | 993588450 | 120 | 34508 | 20001 |
| Guangdong | Guangzhou | 9130 | 2285934710 | 255 | 86011 | 54134 |
| Guangdong | Heyuan | 3090 | 100600270 | 56 | 8927 | 5992 |
| Guangdong | Huizhou | 3750 | 410305320 | 76 | 15916 | 13339 |
| Guangdong | Jieyang | 7040 | 215246810 | 62 | 14165 | 10373 |
| Guangdong | Jiangmen | 3980 | 290040730 | 48 | 17079 | 10298 |
| Guangdong | Maoming | 8070 | 309217680 | 73 | 21502 | 13993 |
| Guangdong | Meizhou | 5490 | 111021330 | 46 | 12616 | 9546 |
| Guangdong | Qingyuan | 4370 | 156519490 | 58 | 11775 | 7858 |
| Guangdong | Shantou | 5670 | 251205040 | 48 | 16283 | 10469 |
| Guangdong | Shanwei | 3550 | 92031830 | 35 | 7010 | 5316 |
| Guangdong | Shaoguan | 3360 | 134391100 | 54 | 13500 | 7385 |
| Guangdong | Shenzhen | 4450 | 2422197710 | 140 | 43569 | 36321 |
| Guangdong | Yangjiang | 2980 | 135031490 | 57 | 10929 | 5631 |
| Guangdong | Yunfu | 3000 | 84912840 | 23 | 6606 | 4625 |
| Guangdong | Zhanjiang | 8430 | 300839280 | 108 | 26850 | 13651 |
| Guangdong | Zhaoqing | 4480 | 220180120 | 56 | 13426 | 8140 |
| Guangdong | Zhongshan | 1660 | 363270130 | 62 | 15685 | 8800 |
| Guangdong | Zhuhai | 1230 | 291473500 | 45 | 8849 | 7090 |
| Guangxi | Baise | 4190 | 117677320 | 36 | 11519 | 6647 |
| Guangxi | Beihai | 1780 | 121330100 | 27 | 6056 | 3618 |
| Guangxi | Chongzuo | 2510 | 101649160 | 29 | 5060 | 3451 |
| Guangxi | Fangchenggang | 990 | 69681850 | 16 | 2730 | 2100 |
| Guangxi | Guigang | 5590 | 116987540 | 56 | 11493 | 6870 |
| Guangxi | Guilin | 5360 | 200360550 | 68 | 16474 | 11997 |
| Guangxi | Hechi | 4310 | 78829990 | 36 | 12359 | 6594 |
| Guangxi | Hezhou | 2450 | 60262880 | 31 | 5554 | 3760 |
| Guangxi | Laibin | 2690 | 69241120 | 25 | 6848 | 3898 |
| Guangxi | Liuzhou | 3890 | 305364570 | 64 | 18545 | 10946 |
| Guangxi | Nanning | 7640 | 402690530 | 120 | 38532 | 24582 |
| Guangxi | Qinzhou | 4130 | 129196470 | 26 | 9762 | 5653 |
| Guangxi | Wuzhou | 3500 | 102984700 | 44 | 11154 | 6322 |
| Guangxi | Yulin | 7280 | 161545710 | 51 | 17121 | 9636 |
| Guizhou | Anshun | 3020 | 84940000 | 74 | 10713 | 4174 |
| Guizhou | Bijie | 9260 | 192142530 | 514 | 36989 | 10321 |
| Guizhou | Guiyang | 4130 | 379845380 | 192 | 32707 | 17794 |
| Guizhou | Liupanshui | 3450 | 152569000 | 114 | 13370 | 5670 |
| Guizhou | Tongren | 4220 | 106652000 | 107 | 16576 | 6843 |
| Guizhou | Zunyi | Not available | Not available | Not available | Not available | Not available |
| Hainan | Danzhou | 960 | 32296930 | 19 | 3076 | 1772 |
| Hainan | Haikou | 1740 | 151051300 | 47 | 14470 | 9030 |
| Hainan | Sansha | Not available | Not available | 1 | 30 | 4 |
| Hainan | Sanya | 600 | 59550570 | 21 | 3957 | 2514 |
| Hebei | Baoding | 12040 | 358978850 | 370 | 46703 | 30808 |
| Hebei | Cangzhou | 7800 | 367641210 | 197 | 33884 | 20909 |
| Hebei | Chengde | 3810 | 148151020 | 86 | 17325 | 10614 |
| Hebei | Handan | 10540 | 345457050 | 265 | 37986 | 23482 |
| Hebei | Hcngshui | 4550 | 155869090 | 144 | 16527 | 11656 |
| Hebei | Langfang | 4770 | 310822490 | 188 | 18730 | 13556 |
| Hebei | Qinhuangdao | 2990 | 163556310 | 67 | 14207 | 9695 |
| Hebei | Shijiazhuang | 9770 | 608261800 | 256 | 48927 | 37438 |
| Hebei | Tangshan | 7570 | 695497000 | 203 | 35979 | 22677 |
| Hebei | Xingtai | 7930 | 215076110 | 204 | 29574 | 19902 |
| Hebei | Zhangjiakou | 2280 | 153662020 | 125 | 20837 | 10301 |
| Heilongjiang | Daqing | 2730 | 280115830 | 116 | 16728 | 9913 |
| Heilongjiang | Harbin | 9530 | 630047940 | 326 | 75343 | 26563 |
| Heilongjiang | Hegang | 1000 | 28957700 | 49 | 8455 | 3090 |
| Heilongjiang | Heihe | 1590 | 50512990 | 60 | 7974 | 4032 |
| Heilongjiang | Jiamusi | 2340 | 101204910 | 95 | 15359 | 6094 |
| Heilongjiang | Jixi | Not available | 1619691 | 67 | 11248 | 4625 |
| Heilongjiang | Mudanjiang | 2540 | 130271110 | 80 | 15850 | 7988 |
| Heilongjiang | Qiqihar | 5320 | 134015710 | 114 | 26749 | 10333 |
| Heilongjiang | Qitaihe | 780 | 25032580 | 27 | 4131 | 1761 |
| Heilongjiang | Shuangyashan | 1420 | 50700750 | 51 | 8867 | 2011 |
| Heilongjiang | Suihua | 5200 | 135958520 | 40 | 18122 | 10654 |
| Heilongjiang | Yichun | 1150 | 27415230 | 39 | 6463 | 2681 |
| Henan | Anyang | 6260 | 239322380 | 95 | 21943 | 13807 |
| Henan | Hebi | 1700 | 86190130 | 43 | 7907 | 3923 |
| Henan | Jiaozuo | 3720 | 237149830 | 83 | 17869 | 8918 |
| Henan | Kaifeng | 5590 | 200222690 | 89 | 22232 | 12354 |
| Henan | Luohe | 2680 | 123666330 | 45 | 10854 | 5402 |
| Henan | Luoyang | 7390 | 464078210 | 154 | 38026 | 19481 |
| Henan | Nanyang | 12360 | 356677340 | 126 | 38292 | 18907 |
| Henan | Pingdingshan | 5690 | 213522320 | 87 | 24484 | 11820 |
| Henan | Puyang | 4330 | 165447000 | 66 | 16575 | 8669 |
| Henan | Sanmenxia | 2280 | 152812440 | 51 | 12168 | 5715 |
| Henan | Shangqiu | 9930 | 238903460 | 82 | 26079 | 15046 |
| Henan | Xinxiang | 6520 | 252655350 | 118 | 27724 | 14987 |
| Henan | Xinyang | 9120 | 238779650 | 314 | 31228 | 12025 |
| Henan | Xuchang | 5090 | 283062180 | 103 | 18739 | 9951 |
| Henan | Zhengzhou | 8530 | 1014331730 | 246 | 86152 | 42051 |
| Henan | Zhoukou | 12580 | 268721000 | 198 | 33280 | 17602 |
| Henan | Zhumadian | 9630 | 237033000 | 112 | 30163 | 13200 |
| Hubei | Ezhou | 1080 | 100530000 | 20 | 4205 | 2219 |
| Hubei | Huanggang | 7430 | 203520000 | 68 | 22108 | 12776 |
| Hubei | Huangshi | 2730 | 158733000 | 42 | 12535 | 5973 |
| Hubei | Jingmen | 2940 | 184789000 | 56 | 13165 | 7374 |
| Hubei | Jingzhou | 6420 | 208218000 | 67 | 20827 | 12526 |
| Hubei | Shiyan | 3460 | 174782000 | 60 | 19827 | 10358 |
| Hubei | Suizhou | 2500 | 101119000 | 42 | 6962 | 5075 |
| Hubei | Wuhan | 8690 | 1484729000 | 398 | 81765 | 42271 |
| Hubei | Xiangyang | 5920 | 430979000 | 81 | 24938 | 14048 |
| Hubei | Xianning | 3050 | 136242000 | 35 | 9787 | 6917 |
| Hubei | Xiaogan | 5280 | 191290000 | 60 | 16312 | 9263 |
| Hubei | Yichang | 3920 | 406418000 | 95 | 22232 | 11308 |
| Hunan | Changde | 6060 | 339420300 | 109 | 24242 | 17387 |
| Hunan | Changsha | 7190 | 1100341160 | 232 | 62042 | 30793 |
| Hunan | Chenzhou | 5340 | 239186960 | 109 | 24303 | 11693 |
| Hunan | Hengyang | 8000 | 304602790 | 149 | 31912 | 17936 |
| Hunan | Huaihua | 5230 | 151326890 | 411 | 25338 | 12005 |
| Hunan | Loudi | 4540 | 154041370 | 112 | 20138 | 9484 |
| Hunan | Shaoyang | 8270 | 178264810 | 129 | 32588 | 14478 |
| Hunan | Xiangtan | 2890 | 216135800 | 68 | 15435 | 7530 |
| Hunan | Yiyang | 4780 | 175837800 | 92 | 18916 | 11167 |
| Hunan | Yongzhou | 6430 | 180564990 | 145 | 28582 | 13656 |
| Hunan | Yueyang | 5680 | 341100680 | 239 | 22406 | 15187 |
| Hunan | Zhangjiajie | 1700 | 57891550 | 32 | 5918 | 3303 |
| Hunan | Zhuzhou | 4030 | 263154230 | 185 | 26522 | 10534 |
| Inner Mongolia | Baotou | 2240 | 295179000 | 105 | 17530 | 8809 |
| Inner Mongolia | Bayannur | 1740 | 81313000 | 56 | 7710 | 5051 |
| Inner Mongolia | Chifeng | 4590 | 154984000 | 107 | 22402 | 12384 |
| Inner Mongolia | Erdos | 1620 | 376321000 | 96 | 10300 | 6074 |
| Inner Mongolia | Hohhot | 2440 | 290350000 | 108 | 17957 | 10908 |
| Inner Mongolia | Hulunbuir | 2560 | 125290000 | 201 | 12548 | 8000 |
| Inner Mongolia | Tongliao | 3180 | 130160000 | 85 | 12167 | 7533 |
| Inner Mongolia | Ulanqab | 2720 | 76452000 | 53 | 7212 | 4155 |
| Inner Mongolia | Wuhai | 560 | 49593600 | 28 | 3080 | 1544 |
| Jiangsu | Changzhou | 3810 | 705027000 | 78 | 20927 | 14087 |
| Jiangsu | Huai'an | 5610 | 360125000 | 64 | 18597 | 13435 |
| Jiangsu | Lianyungang | 5330 | 277170000 | 89 | 18215 | 12102 |
| Jiangsu | Nanjing | 6890 | 1282040000 | 222 | 49448 | 31560 |
| Jiangsu | Nantong | 7630 | 842700000 | 246 | 35425 | 19852 |
| Jiangsu | Suqian | 5910 | 275072000 | 229 | 28108 | 12169 |
| Jiangsu | Suzhou | 6970 | 1859747000 | 206 | 58022 | 32852 |
| Jiangsu | Taizhou | 5040 | 510763000 | 79 | 20253 | 12251 |
| Jiangsu | Wuxi | 4950 | 1143862000 | 185 | 39743 | 21004 |
| Jiangsu | Xuzhou | 10420 | 675523000 | 161 | 41553 | 25776 |
| Jiangsu | Yancheng | 8250 | 548708000 | 164 | 29155 | 18753 |
| Jiangsu | Yangzhou | 4590 | 546617000 | 80 | 17119 | 11209 |
| Jiangsu | Zhenjiang | 2710 | 405000000 | 50 | 11416 | 8200 |
| Jiangxi | Fuzhou | 4310 | 138240380 | 49 | 11028 | 5854 |
| Jiangxi | Ganzhou | 9780 | 280723720 | 98 | 30499 | 15049 |
| Jiangxi | Ji'an | 5380 | 174222920 | 74 | 17522 | 8405 |
| Jiangxi | Jingdezhen | 1700 | 84659820 | 31 | 7590 | 6043 |
| Jiangxi | Jiujiang | 5220 | 270018520 | 62 | 17367 | 10493 |
| Jiangxi | Nanchang | 5280 | 527467160 | 122 | 28222 | 14797 |
| Jiangxi | Pingxiang | 2000 | 100905050 | 33 | 8037 | 4467 |
| Jiangxi | Shangrao | 7860 | 221747580 | 148 | 25879 | 11032 |
| Jiangxi | Xinyu | 1180 | 102734430 | 16 | 4902 | 2611 |
| Jiangxi | Yichun | 6030 | 218085440 | 50 | 16100 | 8599 |
| Jiangxi | Yingtan | 1280 | 81897870 | 33 | 5697 | 2683 |
| Jilin | Baicheng | 1900 | 60650610 | 43 | 6562 | 4977 |
| Jilin | Baishan | 1190 | 66170990 | 40 | 7912 | 3715 |
| Jilin | Changchun | Not available | 717571170 | 186 | 48228 | 24470 |
| Jilin | Jilin | 4140 | 221023860 | 172 | 26193 | 13368 |
| Jilin | Liaoyuan | 1180 | 62160110 | 33 | 5330 | 3036 |
| Jilin | Siping | 3200 | 94432420 | 78 | 15932 | 7564 |
| Jilin | Songyuan | 2750 | 137360090 | 77 | 9085 | 6700 |
| Jilin | Tonghua | 2170 | 82930000 | 72 | 10895 | 6035 |
| Liaoning | Anshan | 3430 | 175105800 | 102 | 18745 | 6684 |
| Liaoning | Benxi | 1470 | 82308670 | 43 | 10716 | 3898 |
| Liaoning | Chaoyang | 3360 | 83142970 | 96 | 15540 | 7933 |
| Liaoning | Dalian | 5950 | 766848230 | 178 | 45129 | 21721 |
| Liaoning | Dandong | 2350 | 81673890 | 48 | 11356 | 5908 |
| Liaoning | Fushun | 2100 | 104882110 | 56 | 11886 | 5512 |
| Liaoning | Fuxin | 1860 | 44599060 | 56 | 10371 | 4446 |
| Liaoning | Huludao | 2770 | 81278410 | 107 | 11928 | 5250 |
| Liaoning | Jinzhou | 2960 | 119240930 | 79 | 15623 | 6349 |
| Liaoning | Liaoyang | 1760 | 86972600 | 62 | 12056 | 4908 |
| Liaoning | Panjin | 1300 | 121657820 | 61 | 8961 | 3987 |
| Liaoning | Shenyang | 7410 | 629239810 | 274 | 64835 | 30126 |
| Liaoning | Tiding | 2930 | 61661890 | 68 | 11994 | 5548 |
| Liaoning | Yingkou | 2320 | 134672080 | 139 | 14491 | 6510 |
| Ningxia | Guyuan | 1510 | 30319440 | 13 | 5572 | 2670 |
| Ningxia | Shizuishan | 740 | 60591840 | 36 | 4137 | 2240 |
| Ningxia | Wuzhong | 1440 | 53453360 | 50 | 5637 | 2836 |
| Ningxia | Yinchuan | 1910 | 190148240 | 86 | 16361 | 9684 |
| Ningxia | Zhongwei | 1220 | 40299470 | 32 | 4159 | 1985 |
| Qinghai | Haibei | Not available | Not available | Not available | Not available | Not available |
| Qinghai | Haidong | 1720 | 45146060 | 57 | 4713 | 2001 |
| Qinghai | Hainan | Not available | Not available | Not available | Not available | Not available |
| Qinghai | Haixi | Not available | Not available | Not available | Not available | Not available |
| Qinghai | Xining | 2360 | 128641370 | 78 | 18594 | 8322 |
| Qinghai | Yushu | Not available | Not available | Not available | Not available | Not available |
| Shaanxi | Ankang | 3040 | 113377000 | 50 | 11433 | 5523 |
| Shaanxi | Baoji | 3780 | 226516000 | 105 | 20508 | 10212 |
| Shaanxi | Hanzhong | 3830 | 147188000 | 84 | 18881 | 7194 |
| Shaanxi | Shangluo | 2520 | 82476700 | 180 | 10283 | 4581 |
| Shaanxi | Tongchuan | 810 | 32795500 | 45 | 5474 | 2534 |
| Shaanxi | Weinan | 5460 | 176771300 | 163 | 20622 | 9680 |
| Shaanxi | Xi'an | 9460 | 834986000 | 343 | 63365 | 33776 |
| Shaanxi | Xianyang | 4640 | 237645300 | 154 | 24172 | 10982 |
| Shaanxi | Yan'an | 2360 | 155891200 | 61 | 10480 | 5400 |
| Shaanxi | Yulin | 3840 | 384861900 | 104 | 16286 | 7622 |
| Shandong | Binzhou | 3950 | 264052000 | 107 | 16031 | 10724 |
| Shandong | Dezhou | 5970 | 338030000 | 110 | 18047 | 14537 |
| Shandong | Dongying | 1960 | 415247000 | 74 | 11624 | 7386 |
| Shandong | Heze | 10220 | 307878000 | 250 | 37414 | 22377 |
| Shandong | Jinan | 6500 | 785656000 | 246 | 48843 | 32131 |
| Shandong | Jining | 8850 | 493058000 | 215 | 38226 | 22731 |
| Shandong | Laiwu | 1290 | 100565000 | 33 | 5598 | 3509 |
| Shandong | Liaocheng | 6420 | 315215000 | 159 | 25329 | 13720 |
| Shandong | Linyi | 11710 | 471780000 | 202 | 43728 | 24308 |
| Shandong | Qingdao | 8110 | 1200152000 | 421 | 45136 | 34578 |
| Shandong | Rizhao | 3050 | 220217000 | 57 | 10641 | 7148 |
| Shandong | Tai'an | 5720 | 365153000 | 105 | 25251 | 14612 |
| Shandong | Weifang | 9110 | 615678000 | 210 | 42564 | 27158 |
| Shandong | Weihai | 2560 | 364148000 | 58 | 15189 | 9191 |
| Shandong | Yantai | 6540 | 783258000 | 193 | 31861 | 19486 |
| Shandong | Zaozhuang | 4180 | 240238000 | 82 | 17480 | 10337 |
| Shandong | Zibo | 4340 | 506835000 | 160 | 24226 | 16099 |
| Shanghai | Shanghai | 14590 | 3267987000 | 364 | 129007 | 74948 |
| Shanxi | Changzhi | 3390 | 164563360 | 137 | 14843 | 8696 |
| Shanxi | Datong | 3180 | 127195980 | 122 | 17008 | 9511 |
| Shanxi | Jinzhong | 3330 | 144760390 | 111 | 12885 | 7427 |
| Shanxi | Jincheng | 2210 | 135185300 | 85 | 9080 | 5875 |
| Shanxi | Linfen | 4330 | 144004300 | 186 | 17482 | 11602 |
| Shanxi | Lvliang | 3880 | 142031950 | 103 | 9140 | 7469 |
| Shanxi | Shuozhou | 1630 | 106563320 | 69 | 6822 | 3129 |
| Shanxi | Taiyuan | 3730 | 388447780 | 163 | 37296 | 23018 |
| Shanxi | Xinzhou | 3080 | 98912980 | 110 | 9536 | 6067 |
| Shanxi | Yangquan | 1320 | 73369440 | 49 | 6194 | 4059 |
| Shanxi | Yuncheng | Not available | 2768379 | 267 | 21117 | 12486 |
| Sichuan | Bazhong | 3720 | 64588420 | 78 | 14253 | 6543 |
| Sichuan | Chengdu | 14560 | 1534277160 | 892 | 135812 | 61548 |
| Sichuan | Dazhou | 6690 | 169017410 | 412 | 31822 | 7356 |
| Sichuan | Deyang | 3870 | 221386730 | 90 | 16496 | 9213 |
| Sichuan | Guang'an | 4630 | 125024220 | 73 | 13982 | 5555 |
| Sichuan | Guangyuan | 3020 | 80185000 | 80 | 15895 | 6343 |
| Sichuan | Leshan | 3510 | 161509490 | 102 | 17644 | 7807 |
| Sichuan | Luzhou | 5100 | 169497120 | 147 | 22407 | 9806 |
| Sichuan | Meishan | 3450 | 125602140 | 88 | 12978 | 6175 |
| Sichuan | Mianyang | 5370 | 230382140 | 117 | 25937 | 12838 |
| Sichuan | Nanchong | 7280 | 200603330 | 165 | 31167 | 13805 |
| Sichuan | Neijiang | 4130 | 141175210 | 76 | 17520 | 5711 |
| Sichuan | Panzhihua | 1090 | 117352380 | 31 | 9404 | 2663 |
| Sichuan | Suining | 3680 | 122139140 | 43 | 13822 | 6962 |
| Sichuan | Ya'an | 1540 | 64610490 | 43 | 10415 | 4157 |
| Sichuan | Yibin | 5540 | 202637070 | 135 | 25106 | 9684 |
| Sichuan | Zigong | 3230 | 140671310 | 72 | 16868 | 4857 |
| Sichuan | Ziyang | 3480 | 106653320 | 47 | 12145 | 5402 |
| Tianjin | Tianjin | 10660 | 1880964000 | 420 | 60337 | 43020 |
| Tibet | Lasa | 550 | 54078000 | 29 | 3644 | 2878 |
| Tibet | Linzhi | 190 | 15001000 | 17 | 803 | 451 |
| Tibet | Naqu | Not available | 573996 | 28 | 1498 | 878 |
| Tibet | Qamdo | 760 | 19142000 | 29 | 1798 | 934 |
| Tibet | Shannan | 370 | 16432000 | 18 | 785 | 1899 |
| Tibet | Xigaze | 800 | 24320490 | 29 | 2219 | 1454 |
| Xinjiang | Altay | Not available | Not available | Not available | Not available | Not available |
| Xinjiang | Hami | 560 | 53660850 | 35 | 3375 | 2240 |
| Xinjiang | Karamay | 310 | 89814200 | 13 | 1941 | 1723 |
| Xinjiang | Shihezi | Not available | Not available | Not available | Not available | Not available |
| Xinjiang | Turpan | 640 | 31059380 | 19 | 2832 | 1593 |
| Xinjiang | Urumqi | 2220 | 309976590 | 130 | 28471 | 15187 |
| Yunnan | Baoshan | 2630 | 73814480 | 48 | 9690 | 5133 |
| Yunnan | Kunming | 5670 | 520689790 | 322 | 55755 | 28606 |
| Yunnan | Lijiang | 1230 | 35076280 | 30 | 4703 | 2067 |
| Yunnan | Lincang | 2400 | 63001990 | 52 | 8829 | 3398 |
| Yunnan | Pu'er | 2540 | 66247680 | 47 | 10032 | 4434 |
| Yunnan | Qujing | 6620 | 201335560 | 114 | 25655 | 9835 |
| Yunnan | Yuxi | 2200 | 149303830 | 71 | 11126 | 6074 |
| Yunnan | Zhaotong | 6220 | 88954040 | 167 | 21739 | 7422 |
| Zhejiang | Hangzhou | 7640 | 1350915080 | 316 | 75186 | 44896 |
| Zhejiang | Huzhou | 2670 | 271906750 | 62 | 13598 | 8847 |
| Zhejiang | Jiaxing | 3580 | 487198360 | Not available | Not available | Not available |
| Zhejiang | Jinhua | 4870 | 410023190 | 137 | 29290 | 17286 |
| Zhejiang | Lishui | 2700 | 139466500 | 56 | 12340 | 8032 |
| Zhejiang | Ningbo | 6000 | 1074546320 | 170 | 35591 | 25850 |
| Zhejiang | Quzhou | 2580 | 147058160 | 84 | 12571 | 7294 |
| Zhejiang | Shaoxing | 4470 | 541689520 | 81 | 21651 | 15662 |
| Zhejiang | Taizhou | 6040 | 487466960 | 122 | 26879 | 18103 |
| Zhejiang | Wenzhou | 8270 | 600616160 | 145 | 37414 | 28387 |
| Zhejiang | Zhoushan | 970 | 131669860 | 33 | 5698 | 4105 |

# Supplement 3: Cities categorized according to the total bed numbers in each province.

| **Provinces** | **City categories** | | | | | |
| --- | --- | --- | --- | --- | --- | --- |
|  | **Category 1** | | **Category 2** | | **Category 3** | |
|  | **Unselected** | **Selected** | **Unselected** | **Selected** | **Unselected** | **Selected** |
| Anhui | 3 | 2 | 6 | 3 | 1 | 1 |
| Beijing | 0 | 0 | 0 | 0 | 0 | 1 |
| Fujian | 0 | 0 | 3 | 5 | 1 | 0 |
| Gansu | 4 | 4 | 2 | 2 | 0 | 0 |
| Guangdong | 4 | 1 | 8 | 4 | 3 | 1 |
| Guangxi | 4 | 2 | 3 | 4 | 1 | 0 |
| Guizhou | 0 | 1 | 0 | 3 | 0 | 2 |
| Hainan | 1 | 2 | 0 | 1 | 0 | 0 |
| Hebei | 0 | 0 | 3 | 3 | 2 | 3 |
| Henan | 1 | 0 | 5 | 5 | 5 | 1 |
| Heilongjiang | 3 | 2 | 3 | 3 | 0 | 1 |
| Hubei | 1 | 2 | 4 | 4 | 1 | 0 |
| Hunan | 0 | 1 | 6 | 3 | 1 | 2 |
| Jilin | 2 | 2 | 1 | 2 | 0 | 1 |
| Jiangsu | 0 | 0 | 4 | 4 | 3 | 2 |
| Jiangxi | 1 | 3 | 4 | 2 | 1 | 0 |
| Liaoning | 1 | 0 | 8 | 3 | 1 | 1 |
| Inner Mongolia | 1 | 2 | 2 | 4 | 0 | 0 |
| Ningxia | 0 | 4 | 0 | 1 | 0 | 0 |
| Qinghai | 0 | 1 | 0 | 1 | 0 | 0 |
| Shandong | 1 | 0 | 5 | 4 | 5 | 2 |
| Shanxi | 1 | 4 | 3 | 2 | 1 | 0 |
| Shaanxi | 1 | 0 | 3 | 5 | 0 | 1 |
| Shanghai | 0 | 0 | 0 | 0 | 0 | 1 |
| Sichuan | 1 | 0 | 9 | 5 | 2 | 1 |
| Tianjin | 0 | 0 | 0 | 0 | 0 | 1 |
| Tibet | 0 | 6 | 0 | 0 | 0 | 0 |
| Xinjiang | 0 | 3 | 0 | 1 | 0 | 0 |
| Yunnan | 2 | 1 | 0 | 4 | 0 | 1 |
| Zhejiang | 0 | 2 | 3 | 3 | 2 | 1 |
| Chongqing | 0 | 0 | 0 | 0 | 0 | 1 |

# Supplement 4: Hospitals grouped according to various levels in each city.

| **City** | **Tertiary hospitals in total (n)** | **Tertiary hospitals selected (n)** | **Secondary hospitals in total (n)** | **Secondary hospitals selected (n)** | **Primary hospitals in total (n)** | **Primary hospitals selected (n)** |
| --- | --- | --- | --- | --- | --- | --- |
| Shanghai | 88 | 12 | 103 | 12 | 47 | 12 |
| Chongqing | 55 | 11 | 102 | 13 | 39 | 12 |
| Beijing | 68 | 12 | 67 | 12 | 81 | 12 |
| Chengdu | 40 | 2 | 60 | 2 | 72 | 2 |
| Tianjin | 50 | 11 | 47 | 10 | 42 | 15 |
| Guangzhou | 45 | 2 | 43 | 3 | 62 | 1 |
| Xi'an | 21 | 2 | 40 | 2 | 50 | 2 |
| Nanjing | 19 | 2 | 32 | 2 | 29 | 2 |
| Kunming | 20 | 2 | 31 | 2 | 50 | 2 |
| Jinan | 24 | 2 | 30 | 2 | 31 | 2 |
| Guiyang | 15 | 2 | 30 | 2 | 20 | 2 |
| Wuxi | 8 | 2 | 30 | 2 | 20 | 2 |
| Harbin | 20 | 2 | 26 | 2 | 30 | 2 |
| Yingkou | 5 | 2 | 25 | 2 | 30 | 2 |
| Dalian | 8 | 2 | 24 | 2 | 19 | 2 |
| Lin fen | 5 | 2 | 24 | 2 | 27 | 2 |
| Changsha | 12 | 2 | 23 | 2 | 30 | 2 |
| Urumqi | 8 | 2 | 23 | 2 | 34 | 2 |
| Qingdao | 10 | 2 | 21 | 2 | 30 | 2 |
| Baoding | 9 | 2 | 21 | 2 | 30 | 2 |
| Jilin | 8 | 2 | 20 | 2 | 22 | 2 |
| Liaocheng | 5 | 2 | 20 | 2 | 21 | 2 |
| Yancheng | 4 | 2 | 20 | 2 | 22 | 2 |
| Zibo | 4 | 2 | 20 | 2 | 21 | 2 |
| Weinan | 3 | 2 | 20 | 2 | 26 | 2 |
| Changchun | 20 | 2 | 19 | 2 | 23 | 2 |
| Zhuzhou | 6 | 2 | 19 | 2 | 24 | 2 |
| Lvliang | 4 | 2 | 19 | 2 | 21 | 2 |
| Mianyang | 4 | 2 | 19 | 2 | 13 | 2 |
| Datong | 5 | 2 | 18 | 2 | 19 | 2 |
| Lanzhou | 12 | 2 | 17 | 2 | 14 | 2 |
| Hengyang | 6 | 2 | 17 | 2 | 18 | 2 |
| Qujing | 4 | 2 | 17 | 2 | 16 | 2 |
| Nanchang | 14 | 2 | 16 | 2 | 23 | 2 |
| Jiaxing | 8 | 2 | 16 | 2 | 25 | 2 |
| Luoyang | 6 | 2 | 16 | 2 | 20 | 2 |
| Baotou | 4 | 2 | 16 | 2 | 13 | 2 |
| Shangluo | 1 | 1 | 16 | 2 | 15 | 2 |
| Suqian | 6 | 2 | 15 | 2 | 20 | 2 |
| Hohhot | 6 | 2 | 15 | 2 | 20 | 2 |
| Liupanshui | 5 | 2 | 15 | 2 | 20 | 2 |
| Handan | 5 | 2 | 15 | 2 | 20 | 2 |
| Daqing | 5 | 2 | 15 | 2 | 14 | 2 |
| Tai'an | 5 | 2 | 15 | 2 | 26 | 2 |
| Bijie | 4 | 2 | 15 | 2 | 20 | 2 |
| Cangzhou | 4 | 2 | 15 | 2 | 20 | 2 |
| Yangquan | 2 | 2 | 15 | 2 | 8 | 2 |
| Tongren | 4 | 1 | 13 | 2 | 19 | 2 |
| Lianyungang | 4 | 2 | 13 | 2 | 15 | 2 |
| Zhangzhou | 3 | 1 | 13 | 3 | 9 | 2 |
| Zhuhai | 8 | 2 | 12 | 2 | 15 | 2 |
| Fuyang | 6 | 2 | 12 | 2 | 18 | 2 |
| Taizhou | 6 | 2 | 12 | 2 | 24 | 2 |
| Yinchuan | 5 | 2 | 12 | 2 | 12 | 2 |
| Baiyin | 4 | 2 | 12 | 2 | 14 | 2 |
| Chenzhou | 4 | 2 | 12 | 2 | 15 | 2 |
| Yiyang | 4 | 2 | 12 | 2 | 14 | 2 |
| Chifeng | 4 | 2 | 12 | 2 | 24 | 2 |
| Jincheng | 4 | 2 | 12 | 2 | 15 | 2 |
| Yan'an | 4 | 2 | 12 | 2 | 13 | 2 |
| Xinzhou | 3 | 2 | 11 | 2 | 15 | 2 |
| Wenzhou | 9 | 2 | 11 | 2 | 14 | 2 |
| Chaoyang | 4 | 2 | 11 | 2 | 14 | 2 |
| Zaozhuang | 4 | 2 | 11 | 3 | 15 | 1 |
| Anqing | 1 | 1 | 11 | 2 | 10 | 2 |
| Xining | 6 | 6 | 10 | 2 | 12 | 2 |
| Xinxiang | 4 | 2 | 10 | 2 | 12 | 2 |
| Yuxi | 4 | 2 | 10 | 2 | 11 | 2 |
| Anshun | 3 | 2 | 10 | 2 | 15 | 2 |
| Chengde | 3 | 2 | 10 | 2 | 13 | 2 |
| Luohe | 2 | 2 | 10 | 2 | 9 | 2 |
| Yulin | 2 | 2 | 10 | 2 | 8 | 2 |
| Guang'an | 1 | 1 | 10 | 2 | 23 | 2 |
| Naqu | 1 | 1 | 10 | 2 | 8 | 2 |
| Lincang | 1 | 1 | 10 | 2 | 6 | 2 |
| Huainan | 4 | 2 | 9 | 2 | 10 | 2 |
| Jiaozuo | 3 | 2 | 9 | 2 | 12 | 2 |
| Xiangyang | 3 | 2 | 9 | 2 | 10 | 2 |
| Jiujiang | 3 | 2 | 9 | 2 | 11 | 2 |
| Tieling | 3 | 2 | 9 | 2 | 12 | 2 |
| Deyang | 3 | 2 | 9 | 2 | 15 | 1 |
| Siping | 1 | 1 | 9 | 2 | 12 | 2 |
| Meishan | 1 | 1 | 9 | 2 | 15 | 2 |
| Zhaotong | 5 | 2 | 8 | 2 | 16 | 2 |
| Wuzhou | 4 | 2 | 8 | 2 | 6 | 2 |
| Zunyi | 4 | 2 | 8 | 2 | 15 | 2 |
| Jiamusi | 4 | 2 | 8 | 2 | 17 | 2 |
| Zhaoqing | 3 | 2 | 8 | 2 | 6 | 2 |
| Qinhuangdao | 3 | 2 | 8 | 2 | 14 | 2 |
| Anyang | 3 | 3 | 8 | 2 | 11 | 1 |
| Tongliao | 3 | 2 | 8 | 2 | 11 | 2 |
| Zhoushan | 3 | 2 | 8 | 2 | 9 | 2 |
| Ezhou | 2 | 2 | 8 | 2 | 6 | 2 |
| Huangshi | 2 | 2 | 8 | 2 | 10 | 2 |
| Tonghua | 2 | 2 | 8 | 2 | 11 | 2 |
| Ankang | 2 | 2 | 8 | 2 | 7 | 2 |
| Chuzhou | 1 | 1 | 8 | 2 | 12 | 2 |
| Yulin | 1 | 1 | 8 | 3 | 10 | 2 |
| Xingtai | 1 | 1 | 8 | 2 | 12 | 2 |
| Xiaogan | 1 | 1 | 8 | 2 | 11 | 2 |
| Danzhou | 0 | 0 | 8 | 2 | 8 | 2 |
| Xigaze | 0 | 0 | 8 | 2 | 7 | 2 |
| Jinhua | 8 | 2 | 7 | 2 | 29 | 2 |
| Liuzhou | 6 | 2 | 7 | 2 | 14 | 2 |
| Quzhou | 4 | 2 | 7 | 2 | 8 | 2 |
| Bayannur | 3 | 2 | 7 | 2 | 10 | 2 |
| Ningde | 2 | 2 | 7 | 2 | 5 | 2 |
| Yichun | 2 | 2 | 7 | 2 | 8 | 2 |
| Suihua | 1 | 1 | 7 | 2 | 7 | 2 |
| Nanping | 0 | 0 | 7 | 3 | 8 | 2 |
| Baishan | 0 | 0 | 7 | 2 | 8 | 2 |
| Wuzhong | 0 | 0 | 7 | 2 | 14 | 2 |
| Shaoguan | 4 | 2 | 6 | 2 | 7 | 2 |
| Beihai | 4 | 2 | 6 | 2 | 17 | 2 |
| Haikou | 4 | 2 | 6 | 2 | 8 | 2 |
| Lasa | 4 | 2 | 6 | 2 | 6 | 2 |
| Maanshan | 3 | 2 | 6 | 2 | 10 | 2 |
| Tianshui | 3 | 2 | 6 | 2 | 9 | 2 |
| Fuxin | 3 | 2 | 6 | 2 | 10 | 2 |
| Lishui | 3 | 2 | 6 | 2 | 6 | 2 |
| Pingxiang | 2 | 2 | 6 | 2 | 10 | 2 |
| Xinyu | 2 | 2 | 6 | 2 | 6 | 2 |
| Ji eyang | 1 | 1 | 6 | 1 | 13 | 3 |
| Yangjiang | 1 | 1 | 6 | 2 | 11 | 2 |
| Yichun | 1 | 1 | 6 | 3 | 7 | 2 |
| Jingmen | 1 | 1 | 6 | 2 | 8 | 2 |
| Suizhou | 1 | 1 | 6 | 2 | 5 | 2 |
| Yingtan | 1 | 1 | 6 | 2 | 10 | 2 |
| Ya'an | 1 | 1 | 6 | 2 | 7 | 2 |
| Karamay | 1 | 1 | 6 | 2 | 5 | 2 |
| Pu'er | 1 | 1 | 6 | 3 | 4 | 2 |
| Chongzuo | 0 | 0 | 6 | 2 | 10 | 2 |
| Zhangjiajie | 0 | 0 | 6 | 2 | 7 | 2 |
| Shizuishan | 0 | 0 | 6 | 2 | 8 | 2 |
| Haidong | 0 | 0 | 6 | 2 | 7 | 2 |
| Qamdo | 0 | 0 | 6 | 2 | 4 | 2 |
| Turpan | 0 | 0 | 6 | 2 | 5 | 2 |
| Sanming | 2 | 2 | 5 | 1 | 9 | 3 |
| Baise | 2 | 2 | 5 | 2 | 5 | 2 |
| Dandong | 2 | 2 | 5 | 2 | 8 | 2 |
| Sanya | 1 | 1 | 5 | 2 | 4 | 3 |
| Haixi | 1 | 1 | 5 | 2 | 6 | 2 |
| Shihezi | 1 | 1 | 5 | 2 | 7 | 2 |
| Shuangyashan | 0 | 0 | 5 | 2 | 10 | 2 |
| Zhongwei | 0 | 0 | 5 | 2 | 4 | 2 |
| Shannan | 0 | 0 | 5 | 2 | 5 | 2 |
| Hami | 0 | 0 | 5 | 2 | 4 | 2 |
| Longyan | 2 | 2 | 4 | 2 | 8 | 2 |
| Putian | 2 | 2 | 4 | 1 | 9 | 3 |
| Qingyang | 2 | 2 | 4 | 2 | 7 | 2 |
| Wuwei | 2 | 2 | 4 | 2 | 9 | 2 |
| Liaoyuan | 1 | 1 | 4 | 2 | 6 | 2 |
| Wuhai | 1 | 1 | 4 | 2 | 6 | 2 |
| Guyuan | 0 | 0 | 4 | 2 | 3 | 2 |
| Haibei | 0 | 0 | 4 | 2 | 4 | 2 |
| Hainan | 0 | 0 | 4 | 2 | 5 | 2 |
| Linzhi | 0 | 0 | 4 | 2 | 6 | 2 |
| Altay | 0 | 0 | 4 | 2 | 3 | 2 |
| Chizhou | 1 | 1 | 3 | 2 | 6 | 2 |
| Zhangye | 1 | 1 | 3 | 2 | 10 | 2 |
| Yushu | 0 | 0 | 3 | 2 | 4 | 2 |
| Sanmenxia | 1 | 1 | 1 | 1 | 15 | 2 |

# Supplement 5: The response rates in various groups

| **Provinces** | **Registered physicians of Ob & Gyn in each province** | **Responding physician in each province** | **Cities numbers selected in each province** | **Hospital numbers selected in each province** | **City** | **City categories** | **Hospital numbers selected in each city** | **Responding physicians in each city** | **Hospital Codes** | **Hospital levels** | **Hospital natures** | **Responding physicians in each hospital** | **Physicians of Ob & Gyn in each hospital** |
| --- | --- | --- | --- | --- | --- | --- | --- | --- | --- | --- | --- | --- | --- |
| Yunnan | 4256 | 263 | 6 | 22 | Qujing | 2 | 3 | 30 | 717 | Primary | Specialized | 1 | 11 |
| Xinjiang | 2686 | 231 | 6 | 24 | Turpan | 1 | 4 | 17 | 702 | Secondary | General | 1 | 11 |
| Xinjiang | 2686 | 231 | 6 | 24 | Turpan | 1 | 4 | 17 | 701 | Primary | Specialized | 1 | 10 |
| Fujian | 4074 | 244 | 5 | 20 | Zhangzhou | 2 | 3 | 12 | 63 | Tertiary | General | 2 | 18 |
| Anhui | 5363 | 342 | 6 | 25 | Maanshan | 1 | 2 | 14 | 25 | Secondary | Specialized | 2 | 17 |
| Fujian | 4074 | 244 | 5 | 20 | Nanping | 2 | 4 | 11 | 46 | Secondary | General | 2 | 15 |
| Xinjiang | 2686 | 231 | 6 | 24 | Altay | 1 | 4 | 16 | 686 | Primary | Specialized | 1 | 6 |
| Tibet | 322 | 177 | 6 | 17 | Naqu | 1 | 2 | 9 | 677 | Secondary | General | 1 | 6 |
| Fujian | 4074 | 244 | 5 | 20 | Sanming | 2 | 6 | 32 | 57 | Primary | General | 1 | 6 |
| Shandong | 12695 | 512 | 6 | 30 | Tai'an | 2 | 5 | 82 | 525 | Primary | Specialized | 2 | 12 |
| Gansu | 2492 | 181 | 6 | 23 | Zhangye | 1 | 4 | 17 | 87 | Primary | Specialized | 3 | 16 |
| Inner Mongolia | 3274 | 203 | 6 | 26 | Baotou | 2 | 5 | 34 | 445 | Secondary | General | 2 | 9 |
| Guangdong | 11859 | 490 | 6 | 19 | Shaoguan | 2 | 5 | 87 | 98 | Primary | Specialized | 3 | 13 |
| Xinjiang | 2686 | 231 | 6 | 24 | Altay | 1 | 4 | 16 | 685 | Secondary | General | 1 | 4 |
| Qinghai | 710 | 246 | 6 | 25 | Haixi | 1 | 4 | 21 | 495 | Primary | General | 1 | 4 |
| Shaanxi | 4289 | 429 | 6 | 27 | Weinan | 2 | 5 | 86 | 583 | Primary | General | 1 | 4 |
| Shanxi | 4442 | 388 | 6 | 33 | Yangquan | 1 | 6 | 43 | 571 | Secondary | General | 1 | 4 |
| Yunnan | 4256 | 263 | 6 | 22 | Zhaotong | 2 | 6 | 96 | 725 | Primary | Specialized | 2 | 8 |
| Yunnan | 4256 | 263 | 6 | 22 | Zhaotong | 2 | 6 | 96 | 723 | Secondary | General | 3 | 11 |
| Gansu | 2492 | 181 | 6 | 23 | Lanzhou | 2 | 3 | 26 | 69 | Tertiary | General | 5 | 18 |
| Inner Mongolia | 3274 | 203 | 6 | 26 | Bayannur | 1 | 5 | 27 | 441 | Secondary | General | 4 | 14 |
| Yunnan | 4256 | 263 | 6 | 22 | Lincang | 1 | 4 | 43 | 713 | Secondary | General | 4 | 14 |
| Inner Mongolia | 3274 | 203 | 6 | 26 | Baotou | 2 | 5 | 34 | 444 | Primary | General | 1 | 3 |
| Fujian | 4074 | 244 | 5 | 20 | Nanping | 2 | 4 | 11 | 48 | Primary | Specialized | 3 | 9 |
| Shaanxi | 4289 | 429 | 6 | 27 | Yan'an | 2 | 6 | 78 | 595 | Tertiary | General | 5 | 15 |
| Heilongjiang | 3956 | 346 | 6 | 28 | Yichun | 1 | 4 | 9 | 260 | Secondary | General | 2 | 6 |
| Yunnan | 4256 | 263 | 6 | 22 | Yuxi | 2 | 3 | 28 | 720 | Secondary | Specialized | 5 | 15 |
| Yunnan | 4256 | 263 | 6 | 22 | Pu'er | 2 | 2 | 17 | 716 | Secondary | General | 5 | 14 |
| Henan | 9336 | 416 | 6 | 31 | Luohe | 2 | 6 | 98 | 222 | Primary | General | 3 | 8 |
| Gansu | 2492 | 181 | 6 | 23 | Zhangye | 1 | 4 | 17 | 85 | Secondary | General | 3 | 8 |
| Liaoning | 5615 | 319 | 4 | 24 | Fuxin | 2 | 6 | 68 | 431 | Primary | General | 2 | 5 |
| Tianjin | 2097 | 387 | 1 | 13 | Tianjin | 3 | 13 | 387 | 661 | Primary | General | 2 | 5 |
| Guangxi | 4478 | 285 | 6 | 19 | Wuzhou | 2 | 4 | 27 | 121 | Secondary | Specialized | 4 | 10 |
| Gansu | 2492 | 181 | 6 | 23 | Zhangye | 1 | 4 | 17 | 88 | Tertiary | General | 6 | 15 |
| Hubei | 6554 | 376 | 6 | 32 | Xiaogan | 2 | 5 | 48 | 290 | Tertiary | General | 5 | 12 |
| Chongqing | 3199 | 177 | 1 | 15 | Chongqing | 3 | 15 | 177 | 770 | Tertiary | General | 6 | 14 |
| Ningxia | 885 | 221 | 5 | 21 | Zhongwei | 1 | 4 | 37 | 481 | Primary | Specialized | 4 | 9 |
| Henan | 9336 | 416 | 6 | 31 | Jiaozuo | 2 | 5 | 44 | 207 | Tertiary | General | 5 | 11 |
| Zhejiang | 8597 | 745 | 6 | 36 | Quzhou | 2 | 6 | 82 | 750 | Primary | Specialized | 6 | 13 |
| Hebei | 8525 | 687 | 6 | 33 | Chengde | 2 | 6 | 98 | 184 | Primary | Specialized | 7 | 15 |
| Inner Mongolia | 3274 | 203 | 6 | 26 | Baotou | 2 | 5 | 34 | 443 | Primary | General | 2 | 4 |
| Tibet | 322 | 177 | 6 | 17 | Qamdo | 1 | 4 | 76 | 669 | Secondary | General | 2 | 4 |
| Inner Mongolia | 3274 | 203 | 6 | 26 | Chifeng | 2 | 4 | 25 | 449 | Primary | General | 3 | 6 |
| Inner Mongolia | 3274 | 203 | 6 | 26 | Chifeng | 2 | 4 | 25 | 451 | Primary | General | 2 | 4 |
| Qinghai | 710 | 246 | 6 | 25 | Haibei | 1 | 2 | 7 | 486 | Primary | General | 1 | 2 |
| Guangdong | 11859 | 490 | 6 | 19 | Ji eyang | 2 | 4 | 38 | 95 | Primary | Specialized | 4 | 8 |
| Jilin | 3484 | 232 | 5 | 26 | Liaoyuan | 1 | 5 | 38 | 330 | Primary | Specialized | 4 | 8 |
| Shandong | 12695 | 512 | 6 | 30 | Liaocheng | 2 | 5 | 64 | 516 | Primary | General | 2 | 4 |
| Henan | 9336 | 416 | 6 | 31 | Luohe | 2 | 6 | 98 | 217 | Primary | General | 3 | 6 |
| Jiangxi | 3793 | 384 | 5 | 26 | Nanchang | 2 | 6 | 56 | 396 | Primary | General | 2 | 4 |
| Fujian | 4074 | 244 | 5 | 20 | Nanping | 2 | 4 | 11 | 47 | Secondary | General | 4 | 8 |
| Fujian | 4074 | 244 | 5 | 20 | Nanping | 2 | 4 | 11 | 49 | Secondary | General | 2 | 4 |
| Fujian | 4074 | 244 | 5 | 20 | Sanming | 2 | 6 | 32 | 58 | Tertiary | General | 4 | 8 |
| Shanghai | 3508 | 345 | 1 | 25 | Shanghai | 3 | 25 | 345 | 612 | Primary | General | 2 | 4 |
| Shanghai | 3508 | 345 | 1 | 25 | Shanghai | 3 | 25 | 345 | 604 | Primary | General | 1 | 2 |
| Shanghai | 3508 | 345 | 1 | 25 | Shanghai | 3 | 25 | 345 | 611 | Primary | General | 1 | 2 |
| Inner Mongolia | 3274 | 203 | 6 | 26 | Wuhai | 1 | 3 | 18 | 462 | Secondary | General | 2 | 4 |
| Xinjiang | 2686 | 231 | 6 | 24 | Urumqi | 2 | 4 | 18 | 704 | Tertiary | General | 4 | 8 |
| Guangxi | 4478 | 285 | 6 | 19 | Wuzhou | 2 | 4 | 27 | 122 | Primary | Specialized | 3 | 6 |
| Shaanxi | 4289 | 429 | 6 | 27 | Yan'an | 2 | 6 | 78 | 592 | Primary | Specialized | 3 | 6 |
| Ningxia | 885 | 221 | 5 | 21 | Yinchuan | 2 | 6 | 67 | 479 | Primary | General | 1 | 2 |
| Chongqing | 3199 | 177 | 1 | 15 | Chongqing | 3 | 15 | 177 | 777 | Secondary | General | 3 | 6 |
| Hunan | 7212 | 477 | 6 | 33 | Zhuzhou | 2 | 6 | 141 | 323 | Primary | General | 6 | 12 |
| Jilin | 3484 | 232 | 5 | 26 | Siping | 2 | 5 | 52 | 337 | Secondary | General | 11 | 21 |
| Chongqing | 3199 | 177 | 1 | 15 | Chongqing | 3 | 15 | 177 | 771 | Tertiary | Specialized | 10 | 19 |
| Guangxi | 4478 | 285 | 6 | 19 | Yulin | 2 | 2 | 13 | 126 | Secondary | General | 9 | 17 |
| Hunan | 7212 | 477 | 6 | 33 | Chenzhou | 2 | 6 | 94 | 298 | Tertiary | General | 8 | 15 |
| Shandong | 12695 | 512 | 6 | 30 | Liaocheng | 2 | 5 | 64 | 515 | Secondary | General | 6 | 11 |
| Yunnan | 4256 | 263 | 6 | 22 | Qujing | 2 | 3 | 30 | 719 | Primary | Specialized | 6 | 11 |
| Jilin | 3484 | 232 | 5 | 26 | Tonghua | 2 | 6 | 56 | 340 | Primary | Specialized | 6 | 11 |
| Guangxi | 4478 | 285 | 6 | 19 | Baise | 2 | 5 | 62 | 112 | Tertiary | General | 5 | 9 |
| Zhejiang | 8597 | 745 | 6 | 36 | Lishui | 2 | 6 | 138 | 745 | Primary | Specialized | 5 | 9 |
| Shaanxi | 4289 | 429 | 6 | 27 | Yulin | 2 | 4 | 50 | 598 | Primary | Specialized | 5 | 9 |
| Inner Mongolia | 3274 | 203 | 6 | 26 | Bayannur | 1 | 5 | 27 | 442 | Primary | Specialized | 4 | 7 |
| Jilin | 3484 | 232 | 5 | 26 | Liaoyuan | 1 | 5 | 38 | 332 | Secondary | General | 8 | 14 |
| Guangxi | 4478 | 285 | 6 | 19 | Yulin | 2 | 2 | 13 | 125 | Secondary | General | 4 | 7 |
| Heilongjiang | 3956 | 346 | 6 | 28 | Harbin | 3 | 6 | 76 | 242 | Tertiary | General | 7 | 12 |
| Guangxi | 4478 | 285 | 6 | 19 | Wuzhou | 2 | 4 | 27 | 124 | Tertiary | General | 7 | 12 |
| Guangxi | 4478 | 285 | 6 | 19 | Chongzuo | 1 | 3 | 18 | 117 | Secondary | General | 6 | 10 |
| Tibet | 322 | 177 | 6 | 17 | Lasa | 1 | 3 | 48 | 670 | Secondary | Specialized | 6 | 10 |
| Gansu | 2492 | 181 | 6 | 23 | Lanzhou | 2 | 3 | 26 | 71 | Primary | Specialized | 6 | 10 |
| Shanghai | 3508 | 345 | 1 | 25 | Shanghai | 3 | 25 | 345 | 618 | Secondary | General | 3 | 5 |
| Xinjiang | 2686 | 231 | 6 | 24 | Turpan | 1 | 4 | 17 | 699 | Primary | Specialized | 6 | 10 |
| Hebei | 8525 | 687 | 6 | 33 | Xingtai | 2 | 5 | 97 | 198 | Primary | General | 3 | 5 |
| Heilongjiang | 3956 | 346 | 6 | 28 | Shuangyashan | 1 | 4 | 31 | 251 | Primary | Specialized | 5 | 8 |
| Hubei | 6554 | 376 | 6 | 32 | Suizhou | 1 | 5 | 32 | 279 | Secondary | General | 5 | 8 |
| Ningxia | 885 | 221 | 5 | 21 | Yinchuan | 2 | 6 | 67 | 480 | Secondary | General | 5 | 8 |
| Shandong | 12695 | 512 | 6 | 30 | Zaozhuang | 2 | 5 | 78 | 533 | Secondary | General | 5 | 8 |
| Fujian | 4074 | 244 | 5 | 20 | Zhangzhou | 2 | 3 | 12 | 64 | Secondary | General | 5 | 8 |
| Jiangxi | 3793 | 384 | 5 | 26 | Jiujiang | 2 | 6 | 159 | 389 | Secondary | General | 7 | 11 |
| Shanxi | 4442 | 388 | 6 | 33 | Lvliang | 1 | 6 | 79 | 558 | Primary | General | 7 | 11 |
| Tianjin | 2097 | 387 | 1 | 13 | Tianjin | 3 | 13 | 387 | 654 | Secondary | Specialized | 7 | 11 |
| Yunnan | 4256 | 263 | 6 | 22 | Zhaotong | 2 | 6 | 96 | 724 | Secondary | General | 7 | 11 |
| Inner Mongolia | 3274 | 203 | 6 | 26 | Chifeng | 2 | 4 | 25 | 450 | Secondary | General | 6 | 9 |
| Hubei | 6554 | 376 | 6 | 32 | Ezhou | 1 | 5 | 34 | 261 | Primary | General | 2 | 3 |
| Guangdong | 11859 | 490 | 6 | 19 | Guangzhou | 3 | 4 | 191 | 90 | Primary | General | 4 | 6 |
| Heilongjiang | 3956 | 346 | 6 | 28 | Harbin | 3 | 6 | 76 | 241 | Secondary | General | 6 | 9 |
| Shandong | 12695 | 512 | 6 | 30 | Jinan | 3 | 5 | 134 | 512 | Primary | General | 4 | 6 |
| Heilongjiang | 3956 | 346 | 6 | 28 | Jiamusi | 2 | 5 | 61 | 244 | Primary | Specialized | 6 | 9 |
| Yunnan | 4256 | 263 | 6 | 22 | Lincang | 1 | 4 | 43 | 711 | Primary | Specialized | 6 | 9 |
| Shanxi | 4442 | 388 | 6 | 33 | Lin fen | 2 | 6 | 61 | 553 | Primary | General | 6 | 9 |
| Shanxi | 4442 | 388 | 6 | 33 | Lin fen | 2 | 6 | 61 | 554 | Primary | General | 4 | 6 |
| Jiangxi | 3793 | 384 | 5 | 26 | Nanchang | 2 | 6 | 56 | 394 | Tertiary | General | 18 | 27 |
| Jiangxi | 3793 | 384 | 5 | 26 | Nanchang | 2 | 6 | 56 | 398 | Secondary | General | 6 | 9 |
| Fujian | 4074 | 244 | 5 | 20 | Sanming | 2 | 6 | 32 | 61 | Primary | Specialized | 4 | 6 |
| Shanghai | 3508 | 345 | 1 | 25 | Shanghai | 3 | 25 | 345 | 617 | Primary | General | 2 | 3 |
| Hubei | 6554 | 376 | 6 | 32 | Suizhou | 1 | 5 | 32 | 281 | Primary | General | 4 | 6 |
| Inner Mongolia | 3274 | 203 | 6 | 26 | Tongliao | 2 | 5 | 50 | 456 | Primary | Specialized | 6 | 9 |
| Xinjiang | 2686 | 231 | 6 | 24 | Urumqi | 2 | 4 | 18 | 703 | Secondary | General | 2 | 3 |
| Shaanxi | 4289 | 429 | 6 | 27 | Yan'an | 2 | 6 | 78 | 594 | Secondary | General | 8 | 12 |
| Shanxi | 4442 | 388 | 6 | 33 | Yangquan | 1 | 6 | 43 | 568 | Primary | General | 2 | 3 |
| Heilongjiang | 3956 | 346 | 6 | 28 | Yichun | 1 | 4 | 9 | 259 | Tertiary | General | 2 | 3 |
| Ningxia | 885 | 221 | 5 | 21 | Zhongwei | 1 | 4 | 37 | 484 | Primary | General | 4 | 6 |
| Chongqing | 3199 | 177 | 1 | 15 | Chongqing | 3 | 15 | 177 | 766 | Primary | Specialized | 6 | 9 |
| Shandong | 12695 | 512 | 6 | 30 | Zibo | 2 | 5 | 85 | 535 | Secondary | General | 6 | 9 |
| Gansu | 2492 | 181 | 6 | 23 | Lanzhou | 2 | 3 | 26 | 70 | Tertiary | General | 15 | 21 |
| Shandong | 12695 | 512 | 6 | 30 | Qingdao | 3 | 5 | 69 | 524 | Tertiary | General | 15 | 21 |
| Liaoning | 5615 | 319 | 4 | 24 | Yingkou | 2 | 6 | 37 | 432 | Secondary | General | 5 | 7 |
| Gansu | 2492 | 181 | 6 | 23 | Zhangye | 1 | 4 | 17 | 86 | Primary | General | 5 | 7 |
| Henan | 9336 | 416 | 6 | 31 | Jiaozuo | 2 | 5 | 44 | 211 | Primary | Specialized | 8 | 11 |
| Yunnan | 4256 | 263 | 6 | 22 | Lincang | 1 | 4 | 43 | 712 | Primary | Specialized | 8 | 11 |
| Shandong | 12695 | 512 | 6 | 30 | Tai'an | 2 | 5 | 82 | 527 | Secondary | Specialized | 8 | 11 |
| Hubei | 6554 | 376 | 6 | 32 | Xiangyang | 2 | 6 | 93 | 287 | Primary | Specialized | 8 | 11 |
| Shanxi | 4442 | 388 | 6 | 33 | Datong | 2 | 5 | 83 | 543 | Secondary | General | 11 | 15 |
| Tibet | 322 | 177 | 6 | 17 | Qamdo | 1 | 4 | 76 | 666 | Primary | General | 3 | 4 |
| Inner Mongolia | 3274 | 203 | 6 | 26 | Hohhot | 2 | 4 | 49 | 453 | Secondary | General | 3 | 4 |
| Guangdong | 11859 | 490 | 6 | 19 | Ji eyang | 2 | 4 | 38 | 94 | Primary | General | 3 | 4 |
| Henan | 9336 | 416 | 6 | 31 | Luoyang | 3 | 5 | 64 | 214 | Secondary | General | 6 | 8 |
| Shanghai | 3508 | 345 | 1 | 25 | Shanghai | 3 | 25 | 345 | 603 | Primary | General | 6 | 8 |
| Ningxia | 885 | 221 | 5 | 21 | Shizuishan | 1 | 4 | 44 | 470 | Primary | General | 3 | 4 |
| Jilin | 3484 | 232 | 5 | 26 | Siping | 2 | 5 | 52 | 339 | Primary | General | 9 | 12 |
| Hubei | 6554 | 376 | 6 | 32 | Suizhou | 1 | 5 | 32 | 278 | Secondary | General | 6 | 8 |
| Jiangsu | 10033 | 740 | 6 | 36 | Taizhou | 2 | 6 | 78 | 365 | Primary | General | 6 | 8 |
| Tianjin | 2097 | 387 | 1 | 13 | Tianjin | 3 | 13 | 387 | 657 | Primary | General | 3 | 4 |
| Henan | 9336 | 416 | 6 | 31 | Xinxiang | 2 | 6 | 84 | 227 | Primary | Specialized | 6 | 8 |
| Shaanxi | 4289 | 429 | 6 | 27 | Yan'an | 2 | 6 | 78 | 591 | Primary | Specialized | 3 | 4 |
| Shandong | 12695 | 512 | 6 | 30 | Zaozhuang | 2 | 5 | 78 | 534 | Primary | Specialized | 6 | 8 |
| Jilin | 3484 | 232 | 5 | 26 | Changchun | 3 | 6 | 52 | 350 | Primary | General | 6 | 8 |
| Hebei | 8525 | 687 | 6 | 33 | Chengde | 2 | 6 | 98 | 182 | Secondary | General | 10 | 13 |
| Guangxi | 4478 | 285 | 6 | 19 | Chongzuo | 1 | 3 | 18 | 119 | Primary | Specialized | 7 | 9 |
| Hubei | 6554 | 376 | 6 | 32 | Ezhou | 1 | 5 | 34 | 262 | Tertiary | General | 7 | 9 |
| Hubei | 6554 | 376 | 6 | 32 | Huangshi | 2 | 6 | 76 | 266 | Secondary | General | 7 | 9 |
| Inner Mongolia | 3274 | 203 | 6 | 26 | Tongliao | 2 | 5 | 50 | 458 | Secondary | General | 7 | 9 |
| Guizhou | 3377 | 613 | 6 | 32 | Tongren | 2 | 5 | 89 | 153 | Primary | General | 7 | 9 |
| Hubei | 6554 | 376 | 6 | 32 | Xiaogan | 2 | 5 | 48 | 289 | Primary | Specialized | 7 | 9 |
| Shandong | 12695 | 512 | 6 | 30 | Zibo | 2 | 5 | 85 | 539 | Primary | Specialized | 7 | 9 |
| Beijing | 4843 | 473 | 1 | 20 | Beijing | 3 | 20 | 473 | 39 | Tertiary | General | 11 | 14 |
| Hainan | 969 | 134 | 3 | 10 | Haikou | 2 | 3 | 61 | 161 | Secondary | Specialized | 15 | 19 |
| Ningxia | 885 | 221 | 5 | 21 | Yinchuan | 2 | 6 | 67 | 476 | Tertiary | General | 15 | 19 |
| Inner Mongolia | 3274 | 203 | 6 | 26 | Bayannur | 1 | 5 | 27 | 438 | Tertiary | General | 8 | 10 |
| Anhui | 5363 | 342 | 6 | 25 | Chuzhou | 2 | 3 | 42 | 12 | Primary | General | 4 | 5 |
| Shanxi | 4442 | 388 | 6 | 33 | Lvliang | 1 | 6 | 79 | 559 | Primary | General | 8 | 10 |
| Hebei | 8525 | 687 | 6 | 33 | Qinhuangdao | 2 | 6 | 106 | 193 | Primary | Specialized | 8 | 10 |
| Gansu | 2492 | 181 | 6 | 23 | Qingyang | 1 | 5 | 37 | 74 | Secondary | General | 4 | 5 |
| Ningxia | 885 | 221 | 5 | 21 | Shizuishan | 1 | 4 | 44 | 471 | Primary | Specialized | 8 | 10 |
| Hubei | 6554 | 376 | 6 | 32 | Suizhou | 1 | 5 | 32 | 277 | Primary | General | 4 | 5 |
| Gansu | 2492 | 181 | 6 | 23 | Tianshui | 2 | 4 | 40 | 80 | Primary | General | 4 | 5 |
| Xinjiang | 2686 | 231 | 6 | 24 | Urumqi | 2 | 4 | 18 | 706 | Tertiary | General | 8 | 10 |
| Liaoning | 5615 | 319 | 4 | 24 | Yingkou | 2 | 6 | 37 | 437 | Tertiary | General | 4 | 5 |
| Hunan | 7212 | 477 | 6 | 33 | Changsha | 3 | 6 | 85 | 317 | Primary | General | 4 | 5 |
| Inner Mongolia | 3274 | 203 | 6 | 26 | Tongliao | 2 | 5 | 50 | 459 | Tertiary | General | 13 | 16 |
| Anhui | 5363 | 342 | 6 | 25 | Huainan | 2 | 6 | 70 | 18 | Primary | General | 9 | 11 |
| Zhejiang | 8597 | 745 | 6 | 36 | Jinhua | 2 | 6 | 125 | 738 | Primary | Specialized | 9 | 11 |
| Fujian | 4074 | 244 | 5 | 20 | Sanming | 2 | 6 | 32 | 59 | Tertiary | General | 9 | 11 |
| Tianjin | 2097 | 387 | 1 | 13 | Tianjin | 3 | 13 | 387 | 653 | Secondary | General | 9 | 11 |
| Shanxi | 4442 | 388 | 6 | 33 | Xinzhou | 1 | 6 | 66 | 565 | Secondary | General | 9 | 11 |
| Shaanxi | 4289 | 429 | 6 | 27 | Yan'an | 2 | 6 | 78 | 590 | Secondary | General | 9 | 11 |
| Anhui | 5363 | 342 | 6 | 25 | Anqing | 2 | 5 | 96 | 3 | Primary | General | 5 | 6 |
| Guizhou | 3377 | 613 | 6 | 32 | Anshun | 2 | 4 | 68 | 129 | Primary | General | 5 | 6 |
| Beijing | 4843 | 473 | 1 | 20 | Beijing | 3 | 20 | 473 | 35 | Secondary | General | 5 | 6 |
| Liaoning | 5615 | 319 | 4 | 24 | Chaoyang | 2 | 6 | 164 | 415 | Primary | General | 5 | 6 |
| Hubei | 6554 | 376 | 6 | 32 | Ezhou | 1 | 5 | 34 | 264 | Secondary | Specialized | 10 | 12 |
| Liaoning | 5615 | 319 | 4 | 24 | Fuxin | 2 | 6 | 68 | 429 | Primary | Specialized | 5 | 6 |
| Anhui | 5363 | 342 | 6 | 25 | Huainan | 2 | 6 | 70 | 20 | Secondary | General | 5 | 6 |
| Heilongjiang | 3956 | 346 | 6 | 28 | Jiamusi | 2 | 5 | 61 | 248 | Primary | General | 5 | 6 |
| Henan | 9336 | 416 | 6 | 31 | Jiaozuo | 2 | 5 | 44 | 209 | Secondary | General | 5 | 6 |
| Shanxi | 4442 | 388 | 6 | 33 | Lin fen | 2 | 6 | 61 | 549 | Secondary | General | 10 | 12 |
| Shandong | 12695 | 512 | 6 | 30 | Qingdao | 3 | 5 | 69 | 523 | Primary | Specialized | 10 | 12 |
| Gansu | 2492 | 181 | 6 | 23 | Qingyang | 1 | 5 | 37 | 72 | Secondary | Specialized | 10 | 12 |
| Zhejiang | 8597 | 745 | 6 | 36 | Quzhou | 2 | 6 | 82 | 749 | Primary | Specialized | 10 | 12 |
| Jiangsu | 10033 | 740 | 6 | 36 | Taizhou | 2 | 6 | 78 | 366 | Primary | General | 5 | 6 |
| Shaanxi | 4289 | 429 | 6 | 27 | Weinan | 2 | 5 | 86 | 585 | Primary | Specialized | 10 | 12 |
| Zhejiang | 8597 | 745 | 6 | 36 | Wenzhou | 3 | 6 | 178 | 754 | Primary | General | 5 | 6 |
| Hebei | 8525 | 687 | 6 | 33 | Xingtai | 2 | 5 | 97 | 200 | Primary | General | 5 | 6 |
| Jiangsu | 10033 | 740 | 6 | 36 | Yancheng | 2 | 6 | 155 | 387 | Primary | General | 10 | 12 |
| Hunan | 7212 | 477 | 6 | 33 | Yiyang | 2 | 5 | 48 | 305 | Primary | Specialized | 5 | 6 |
| Hunan | 7212 | 477 | 6 | 33 | Yiyang | 2 | 5 | 48 | 308 | Secondary | General | 10 | 12 |
| Ningxia | 885 | 221 | 5 | 21 | Yinchuan | 2 | 6 | 67 | 478 | Secondary | General | 5 | 6 |
| Fujian | 4074 | 244 | 5 | 20 | Zhangzhou | 2 | 3 | 12 | 65 | Primary | General | 5 | 6 |
| Guangdong | 11859 | 490 | 6 | 19 | Zhuhai | 1 | 3 | 117 | 107 | Primary | General | 5 | 6 |
| Hebei | 8525 | 687 | 6 | 33 | Baoding | 3 | 6 | 177 | 171 | Tertiary | General | 11 | 13 |
| Inner Mongolia | 3274 | 203 | 6 | 26 | Bayannur | 1 | 5 | 27 | 439 | Tertiary | General | 6 | 7 |
| Inner Mongolia | 3274 | 203 | 6 | 26 | Baotou | 2 | 5 | 34 | 447 | Tertiary | General | 12 | 14 |
| Shanxi | 4442 | 388 | 6 | 33 | Jincheng | 1 | 4 | 56 | 546 | Primary | Specialized | 6 | 7 |
| Jilin | 3484 | 232 | 5 | 26 | Liaoyuan | 1 | 5 | 38 | 333 | Secondary | General | 6 | 7 |
| Jiangxi | 3793 | 384 | 5 | 26 | Nanchang | 2 | 6 | 56 | 395 | Tertiary | General | 18 | 21 |
| Jiangsu | 10033 | 740 | 6 | 36 | Nanjing | 3 | 6 | 214 | 363 | Primary | Specialized | 12 | 14 |
| Gansu | 2492 | 181 | 6 | 23 | Wuwei | 1 | 4 | 45 | 81 | Secondary | General | 6 | 7 |
| Gansu | 2492 | 181 | 6 | 23 | Wuwei | 1 | 4 | 45 | 82 | Primary | General | 6 | 7 |
| Shaanxi | 4289 | 429 | 6 | 27 | Yulin | 2 | 4 | 50 | 597 | Primary | General | 6 | 7 |
| Hunan | 7212 | 477 | 6 | 33 | Changsha | 3 | 6 | 85 | 319 | Primary | General | 6 | 7 |
| Shanghai | 3508 | 345 | 1 | 25 | Shanghai | 3 | 25 | 345 | 600 | Tertiary | General | 13 | 15 |
| Guangxi | 4478 | 285 | 6 | 19 | Baise | 2 | 5 | 62 | 109 | Tertiary | General | 14 | 16 |
| Liaoning | 5615 | 319 | 4 | 24 | Dalian | 3 | 6 | 50 | 420 | Tertiary | General | 14 | 16 |
| Anhui | 5363 | 342 | 6 | 25 | Huainan | 2 | 6 | 70 | 22 | Secondary | General | 7 | 8 |
| Xinjiang | 2686 | 231 | 6 | 24 | Shihezi | 1 | 5 | 80 | 698 | Secondary | General | 7 | 8 |
| Inner Mongolia | 3274 | 203 | 6 | 26 | Tongliao | 2 | 5 | 50 | 460 | Secondary | General | 7 | 8 |
| Hubei | 6554 | 376 | 6 | 32 | Xiangyang | 2 | 6 | 93 | 283 | Primary | Specialized | 14 | 16 |
| Shanxi | 4442 | 388 | 6 | 33 | Datong | 2 | 5 | 83 | 544 | Primary | General | 8 | 9 |
| Anhui | 5363 | 342 | 6 | 25 | Fuyang | 3 | 4 | 68 | 17 | Secondary | General | 8 | 9 |
| Hebei | 8525 | 687 | 6 | 33 | Qinhuangdao | 2 | 6 | 106 | 192 | Secondary | General | 8 | 9 |
| Heilongjiang | 3956 | 346 | 6 | 28 | Suihua | 2 | 4 | 48 | 253 | Primary | General | 8 | 9 |
| Shaanxi | 4289 | 429 | 6 | 27 | Weinan | 2 | 5 | 86 | 582 | Secondary | General | 8 | 9 |
| Zhejiang | 8597 | 745 | 6 | 36 | Wenzhou | 3 | 6 | 178 | 758 | Secondary | General | 8 | 9 |
| Shandong | 12695 | 512 | 6 | 30 | Zibo | 2 | 5 | 85 | 536 | Tertiary | General | 8 | 9 |
| Sichuan | 8864 | 743 | 6 | 28 | Deyang | 2 | 5 | 244 | 632 | Primary | Specialized | 17 | 19 |
| Henan | 9336 | 416 | 6 | 31 | Anyang | 2 | 5 | 66 | 202 | Secondary | General | 9 | 10 |
| Jilin | 3484 | 232 | 5 | 26 | Baishan | 1 | 4 | 34 | 328 | Primary | General | 9 | 10 |
| Liaoning | 5615 | 319 | 4 | 24 | Chaoyang | 2 | 6 | 164 | 417 | Primary | General | 9 | 10 |
| Sichuan | 8864 | 743 | 6 | 28 | Guang'an | 2 | 5 | 62 | 636 | Primary | Specialized | 9 | 10 |
| Sichuan | 8864 | 743 | 6 | 28 | Meishan | 2 | 5 | 51 | 643 | Secondary | General | 9 | 10 |
| Heilongjiang | 3956 | 346 | 6 | 28 | Shuangyashan | 1 | 4 | 31 | 252 | Secondary | General | 9 | 10 |
| Guizhou | 3377 | 613 | 6 | 32 | Bijie | 3 | 6 | 80 | 132 | Secondary | General | 10 | 11 |
| Guizhou | 3377 | 613 | 6 | 32 | Bijie | 3 | 6 | 80 | 136 | Primary | General | 10 | 11 |
| Heilongjiang | 3956 | 346 | 6 | 28 | Daqing | 2 | 5 | 121 | 237 | Primary | Specialized | 10 | 11 |
| Shanxi | 4442 | 388 | 6 | 33 | Datong | 2 | 5 | 83 | 541 | Secondary | General | 10 | 11 |
| Heilongjiang | 3956 | 346 | 6 | 28 | Jiamusi | 2 | 5 | 61 | 246 | Secondary | General | 10 | 11 |
| Sichuan | 8864 | 743 | 6 | 28 | Meishan | 2 | 5 | 51 | 642 | Primary | Specialized | 10 | 11 |
| Hebei | 8525 | 687 | 6 | 33 | Qinhuangdao | 2 | 6 | 106 | 191 | Primary | Specialized | 20 | 22 |
| Hainan | 969 | 134 | 3 | 10 | Sanya | 1 | 5 | 50 | 164 | Tertiary | General | 10 | 11 |
| Henan | 9336 | 416 | 6 | 31 | Xinxiang | 2 | 6 | 84 | 230 | Secondary | General | 10 | 11 |
| Jiangsu | 10033 | 740 | 6 | 36 | Yancheng | 2 | 6 | 155 | 386 | Tertiary | General | 20 | 22 |
| Chongqing | 3199 | 177 | 1 | 15 | Chongqing | 3 | 15 | 177 | 778 | Tertiary | General | 20 | 22 |
| Sichuan | 8864 | 743 | 6 | 28 | Guang'an | 2 | 5 | 62 | 635 | Primary | Specialized | 11 | 12 |
| Zhejiang | 8597 | 745 | 6 | 36 | Lishui | 2 | 6 | 138 | 746 | Primary | Specialized | 11 | 12 |
| Tianjin | 2097 | 387 | 1 | 13 | Tianjin | 3 | 13 | 387 | 655 | Secondary | General | 11 | 12 |
| Hubei | 6554 | 376 | 6 | 32 | Xiaogan | 2 | 5 | 48 | 291 | Secondary | General | 11 | 12 |
| Hubei | 6554 | 376 | 6 | 32 | Huangshi | 2 | 6 | 76 | 270 | Primary | Specialized | 12 | 13 |
| Shaanxi | 4289 | 429 | 6 | 27 | Shangluo | 2 | 4 | 57 | 579 | Tertiary | General | 12 | 13 |
| Shaanxi | 4289 | 429 | 6 | 27 | Yulin | 2 | 4 | 50 | 599 | Secondary | General | 12 | 13 |
| Shandong | 12695 | 512 | 6 | 30 | Zaozhuang | 2 | 5 | 78 | 531 | Tertiary | General | 12 | 13 |
| Yunnan | 4256 | 263 | 6 | 22 | Yuxi | 2 | 3 | 28 | 721 | Tertiary | General | 13 | 14 |
| Beijing | 4843 | 473 | 1 | 20 | Beijing | 3 | 20 | 473 | 38 | Tertiary | General | 98 | 105 |
| Shanxi | 4442 | 388 | 6 | 33 | Lin fen | 2 | 6 | 61 | 552 | Tertiary | General | 14 | 15 |
| Tianjin | 2097 | 387 | 1 | 13 | Tianjin | 3 | 13 | 387 | 660 | Tertiary | General | 14 | 15 |
| Liaoning | 5615 | 319 | 4 | 24 | Yingkou | 2 | 6 | 37 | 434 | Secondary | Specialized | 14 | 15 |
| Henan | 9336 | 416 | 6 | 31 | Sanmenxia | 2 | 4 | 60 | 225 | Secondary | General | 15 | 16 |
| Shanghai | 3508 | 345 | 1 | 25 | Shanghai | 3 | 25 | 345 | 601 | Tertiary | General | 15 | 16 |
| Gansu | 2492 | 181 | 6 | 23 | Tianshui | 2 | 4 | 40 | 78 | Secondary | General | 15 | 16 |
| Shanxi | 4442 | 388 | 6 | 33 | Lin fen | 2 | 6 | 61 | 551 | Tertiary | General | 16 | 17 |
| Inner Mongolia | 3274 | 203 | 6 | 26 | Tongliao | 2 | 5 | 50 | 457 | Tertiary | General | 17 | 18 |
| Jiangxi | 3793 | 384 | 5 | 26 | Jiujiang | 2 | 6 | 159 | 388 | Primary | Specialized | 18 | 19 |
| Tibet | 322 | 177 | 6 | 17 | Lasa | 1 | 3 | 48 | 671 | Tertiary | General | 27 | 28 |
| Henan | 9336 | 416 | 6 | 31 | Luohe | 2 | 6 | 98 | 219 | Tertiary | General | 27 | 28 |
| Hainan | 969 | 134 | 3 | 10 | Haikou | 2 | 3 | 61 | 162 | Tertiary | General | 29 | 30 |
| Hebei | 8525 | 687 | 6 | 33 | Qinhuangdao | 2 | 6 | 106 | 195 | Tertiary | Specialized | 35 | 36 |
| Shaanxi | 4289 | 429 | 6 | 27 | Yan'an | 2 | 6 | 78 | 593 | Tertiary | General | 50 | 51 |
| Fujian | 4074 | 244 | 5 | 20 | Putian | 2 | 6 | 185 | 53 | Tertiary | General | 51 | 52 |
| Shandong | 12695 | 512 | 6 | 30 | Jinan | 3 | 5 | 134 | 513 | Tertiary | General | 60 | 61 |
| Jiangsu | 10033 | 740 | 6 | 36 | Nanjing | 3 | 6 | 214 | 360 | Tertiary | Specialized | 97 | 98 |
| Xinjiang | 2686 | 231 | 6 | 24 | Altay | 1 | 4 | 16 | 684 | Primary | Specialized | 5 | 5 |
| Xinjiang | 2686 | 231 | 6 | 24 | Altay | 1 | 4 | 16 | 683 | Secondary | General | 9 | 9 |
| Shaanxi | 4289 | 429 | 6 | 27 | Ankang | 2 | 5 | 103 | 574 | Secondary | Specialized | 32 | 32 |
| Shaanxi | 4289 | 429 | 6 | 27 | Ankang | 2 | 5 | 103 | 577 | Primary | Specialized | 8 | 8 |
| Shaanxi | 4289 | 429 | 6 | 27 | Ankang | 2 | 5 | 103 | 573 | Secondary | General | 12 | 12 |
| Shaanxi | 4289 | 429 | 6 | 27 | Ankang | 2 | 5 | 103 | 575 | Tertiary | General | 38 | 38 |
| Shaanxi | 4289 | 429 | 6 | 27 | Ankang | 2 | 5 | 103 | 576 | Tertiary | General | 13 | 13 |
| Anhui | 5363 | 342 | 6 | 25 | Anqing | 2 | 5 | 96 | 1 | Secondary | General | 9 | 9 |
| Anhui | 5363 | 342 | 6 | 25 | Anqing | 2 | 5 | 96 | 2 | Secondary | General | 25 | 25 |
| Anhui | 5363 | 342 | 6 | 25 | Anqing | 2 | 5 | 96 | 4 | Tertiary | General | 42 | 42 |
| Anhui | 5363 | 342 | 6 | 25 | Anqing | 2 | 5 | 96 | 5 | Primary | General | 15 | 15 |
| Guizhou | 3377 | 613 | 6 | 32 | Anshun | 2 | 4 | 68 | 127 | Secondary | Specialized | 14 | 14 |
| Guizhou | 3377 | 613 | 6 | 32 | Anshun | 2 | 4 | 68 | 128 | Tertiary | General | 19 | 19 |
| Guizhou | 3377 | 613 | 6 | 32 | Anshun | 2 | 4 | 68 | 130 | Primary | General | 30 | 30 |
| Henan | 9336 | 416 | 6 | 31 | Anyang | 2 | 5 | 66 | 203 | Tertiary | Specialized | 26 | 26 |
| Henan | 9336 | 416 | 6 | 31 | Anyang | 2 | 5 | 66 | 206 | Secondary | General | 12 | 12 |
| Henan | 9336 | 416 | 6 | 31 | Anyang | 2 | 5 | 66 | 204 | Tertiary | General | 12 | 12 |
| Henan | 9336 | 416 | 6 | 31 | Anyang | 2 | 5 | 66 | 205 | Primary | General | 7 | 7 |
| Inner Mongolia | 3274 | 203 | 6 | 26 | Bayannur | 1 | 5 | 27 | 440 | Primary | General | 5 | 5 |
| Jilin | 3484 | 232 | 5 | 26 | Baishan | 1 | 4 | 34 | 326 | Secondary | General | 9 | 9 |
| Jilin | 3484 | 232 | 5 | 26 | Baishan | 1 | 4 | 34 | 329 | Secondary | General | 12 | 12 |
| Jilin | 3484 | 232 | 5 | 26 | Baishan | 1 | 4 | 34 | 327 | Primary | General | 4 | 4 |
| Gansu | 2492 | 181 | 6 | 23 | Baiyin | 1 | 3 | 16 | 68 | Secondary | General | 6 | 6 |
| Gansu | 2492 | 181 | 6 | 23 | Baiyin | 1 | 3 | 16 | 67 | Tertiary | General | 6 | 6 |
| Gansu | 2492 | 181 | 6 | 23 | Baiyin | 1 | 3 | 16 | 66 | Primary | General | 4 | 4 |
| Guangxi | 4478 | 285 | 6 | 19 | Baise | 2 | 5 | 62 | 108 | Secondary | Specialized | 22 | 22 |
| Guangxi | 4478 | 285 | 6 | 19 | Baise | 2 | 5 | 62 | 110 | Secondary | General | 10 | 10 |
| Guangxi | 4478 | 285 | 6 | 19 | Baise | 2 | 5 | 62 | 111 | Primary | General | 11 | 11 |
| Inner Mongolia | 3274 | 203 | 6 | 26 | Baotou | 2 | 5 | 34 | 446 | Tertiary | General | 17 | 17 |
| Hebei | 8525 | 687 | 6 | 33 | Baoding | 3 | 6 | 177 | 172 | Tertiary | Specialized | 115 | 115 |
| Hebei | 8525 | 687 | 6 | 33 | Baoding | 3 | 6 | 177 | 174 | Primary | Specialized | 13 | 13 |
| Hebei | 8525 | 687 | 6 | 33 | Baoding | 3 | 6 | 177 | 170 | Secondary | General | 16 | 16 |
| Hebei | 8525 | 687 | 6 | 33 | Baoding | 3 | 6 | 177 | 173 | Secondary | General | 17 | 17 |
| Hebei | 8525 | 687 | 6 | 33 | Baoding | 3 | 6 | 177 | 169 | Primary | General | 5 | 5 |
| Guangxi | 4478 | 285 | 6 | 19 | Beihai | 1 | 4 | 142 | 114 | Secondary | Specialized | 87 | 87 |
| Guangxi | 4478 | 285 | 6 | 19 | Beihai | 1 | 4 | 142 | 113 | Secondary | General | 8 | 8 |
| Guangxi | 4478 | 285 | 6 | 19 | Beihai | 1 | 4 | 142 | 115 | Tertiary | General | 38 | 38 |
| Guangxi | 4478 | 285 | 6 | 19 | Beihai | 1 | 4 | 142 | 116 | Tertiary | General | 9 | 9 |
| Beijing | 4843 | 473 | 1 | 20 | Beijing | 3 | 20 | 473 | 40 | Secondary | Specialized | 26 | 26 |
| Beijing | 4843 | 473 | 1 | 20 | Beijing | 3 | 20 | 473 | 27 | Secondary | General | 16 | 16 |
| Beijing | 4843 | 473 | 1 | 20 | Beijing | 3 | 20 | 473 | 30 | Secondary | General | 30 | 30 |
| Beijing | 4843 | 473 | 1 | 20 | Beijing | 3 | 20 | 473 | 31 | Secondary | General | 23 | 23 |
| Beijing | 4843 | 473 | 1 | 20 | Beijing | 3 | 20 | 473 | 32 | Secondary | General | 6 | 6 |
| Beijing | 4843 | 473 | 1 | 20 | Beijing | 3 | 20 | 473 | 33 | Secondary | General | 27 | 27 |
| Beijing | 4843 | 473 | 1 | 20 | Beijing | 3 | 20 | 473 | 34 | Secondary | General | 6 | 6 |
| Beijing | 4843 | 473 | 1 | 20 | Beijing | 3 | 20 | 473 | 37 | Secondary | General | 13 | 13 |
| Beijing | 4843 | 473 | 1 | 20 | Beijing | 3 | 20 | 473 | 41 | Secondary | General | 12 | 12 |
| Beijing | 4843 | 473 | 1 | 20 | Beijing | 3 | 20 | 473 | 44 | Secondary | General | 29 | 29 |
| Beijing | 4843 | 473 | 1 | 20 | Beijing | 3 | 20 | 473 | 28 | Tertiary | General | 20 | 20 |
| Beijing | 4843 | 473 | 1 | 20 | Beijing | 3 | 20 | 473 | 29 | Tertiary | General | 14 | 14 |
| Beijing | 4843 | 473 | 1 | 20 | Beijing | 3 | 20 | 473 | 42 | Tertiary | General | 50 | 50 |
| Beijing | 4843 | 473 | 1 | 20 | Beijing | 3 | 20 | 473 | 43 | Tertiary | General | 36 | 36 |
| Beijing | 4843 | 473 | 1 | 20 | Beijing | 3 | 20 | 473 | 45 | Tertiary | General | 40 | 40 |
| Beijing | 4843 | 473 | 1 | 20 | Beijing | 3 | 20 | 473 | 26 | Primary | General | 5 | 5 |
| Beijing | 4843 | 473 | 1 | 20 | Beijing | 3 | 20 | 473 | 36 | Primary | General | 6 | 6 |
| Guizhou | 3377 | 613 | 6 | 32 | Bijie | 3 | 6 | 80 | 133 | Secondary | General | 14 | 14 |
| Guizhou | 3377 | 613 | 6 | 32 | Bijie | 3 | 6 | 80 | 131 | Tertiary | General | 16 | 16 |
| Guizhou | 3377 | 613 | 6 | 32 | Bijie | 3 | 6 | 80 | 134 | Tertiary | General | 18 | 18 |
| Guizhou | 3377 | 613 | 6 | 32 | Bijie | 3 | 6 | 80 | 135 | Primary | General | 12 | 12 |
| Hebei | 8525 | 687 | 6 | 33 | Cangzhou | 3 | 5 | 118 | 179 | Primary | Specialized | 14 | 14 |
| Hebei | 8525 | 687 | 6 | 33 | Cangzhou | 3 | 5 | 118 | 177 | Secondary | General | 18 | 18 |
| Hebei | 8525 | 687 | 6 | 33 | Cangzhou | 3 | 5 | 118 | 178 | Secondary | General | 15 | 15 |
| Hebei | 8525 | 687 | 6 | 33 | Cangzhou | 3 | 5 | 118 | 176 | Tertiary | General | 40 | 40 |
| Hebei | 8525 | 687 | 6 | 33 | Cangzhou | 3 | 5 | 118 | 175 | Tertiary | General | 31 | 31 |
| Tibet | 322 | 177 | 6 | 17 | Qamdo | 1 | 4 | 76 | 667 | Primary | Specialized | 52 | 52 |
| Tibet | 322 | 177 | 6 | 17 | Qamdo | 1 | 4 | 76 | 668 | Secondary | General | 19 | 19 |
| Liaoning | 5615 | 319 | 4 | 24 | Chaoyang | 2 | 6 | 164 | 418 | Secondary | General | 29 | 29 |
| Liaoning | 5615 | 319 | 4 | 24 | Chaoyang | 2 | 6 | 164 | 419 | Secondary | General | 34 | 34 |
| Liaoning | 5615 | 319 | 4 | 24 | Chaoyang | 2 | 6 | 164 | 414 | Tertiary | General | 55 | 55 |
| Liaoning | 5615 | 319 | 4 | 24 | Chaoyang | 2 | 6 | 164 | 416 | Tertiary | General | 32 | 32 |
| Hunan | 7212 | 477 | 6 | 33 | Chenzhou | 2 | 6 | 94 | 296 | Primary | Specialized | 13 | 13 |
| Hunan | 7212 | 477 | 6 | 33 | Chenzhou | 2 | 6 | 94 | 297 | Primary | Specialized | 16 | 16 |
| Hunan | 7212 | 477 | 6 | 33 | Chenzhou | 2 | 6 | 94 | 294 | Secondary | General | 15 | 15 |
| Hunan | 7212 | 477 | 6 | 33 | Chenzhou | 2 | 6 | 94 | 295 | Secondary | General | 15 | 15 |
| Hunan | 7212 | 477 | 6 | 33 | Chenzhou | 2 | 6 | 94 | 293 | Tertiary | General | 27 | 27 |
| Sichuan | 8864 | 743 | 6 | 28 | Chengdu | 3 | 4 | 177 | 626 | Primary | Specialized | 14 | 14 |
| Sichuan | 8864 | 743 | 6 | 28 | Chengdu | 3 | 4 | 177 | 625 | Secondary | General | 18 | 18 |
| Sichuan | 8864 | 743 | 6 | 28 | Chengdu | 3 | 4 | 177 | 627 | Secondary | General | 24 | 24 |
| Sichuan | 8864 | 743 | 6 | 28 | Chengdu | 3 | 4 | 177 | 628 | Tertiary | General | 121 | 121 |
| Hebei | 8525 | 687 | 6 | 33 | Chengde | 2 | 6 | 98 | 180 | Secondary | Specialized | 23 | 23 |
| Hebei | 8525 | 687 | 6 | 33 | Chengde | 2 | 6 | 98 | 185 | Primary | Specialized | 14 | 14 |
| Hebei | 8525 | 687 | 6 | 33 | Chengde | 2 | 6 | 98 | 181 | Tertiary | General | 19 | 19 |
| Hebei | 8525 | 687 | 6 | 33 | Chengde | 2 | 6 | 98 | 183 | Tertiary | General | 25 | 25 |
| Anhui | 5363 | 342 | 6 | 25 | Chizhou | 1 | 5 | 52 | 6 | Secondary | General | 16 | 16 |
| Anhui | 5363 | 342 | 6 | 25 | Chizhou | 1 | 5 | 52 | 9 | Secondary | General | 9 | 9 |
| Anhui | 5363 | 342 | 6 | 25 | Chizhou | 1 | 5 | 52 | 7 | Tertiary | General | 16 | 16 |
| Anhui | 5363 | 342 | 6 | 25 | Chizhou | 1 | 5 | 52 | 8 | Primary | General | 6 | 6 |
| Anhui | 5363 | 342 | 6 | 25 | Chizhou | 1 | 5 | 52 | 10 | Primary | General | 5 | 5 |
| Inner Mongolia | 3274 | 203 | 6 | 26 | Chifeng | 2 | 4 | 25 | 448 | Tertiary | General | 14 | 14 |
| Guangxi | 4478 | 285 | 6 | 19 | Chongzuo | 1 | 3 | 18 | 118 | Secondary | General | 5 | 5 |
| Anhui | 5363 | 342 | 6 | 25 | Chuzhou | 2 | 3 | 42 | 13 | Secondary | General | 8 | 8 |
| Anhui | 5363 | 342 | 6 | 25 | Chuzhou | 2 | 3 | 42 | 11 | Tertiary | General | 30 | 30 |
| Liaoning | 5615 | 319 | 4 | 24 | Dalian | 3 | 6 | 50 | 422 | Primary | Specialized | 12 | 12 |
| Liaoning | 5615 | 319 | 4 | 24 | Dalian | 3 | 6 | 50 | 421 | Secondary | General | 8 | 8 |
| Liaoning | 5615 | 319 | 4 | 24 | Dalian | 3 | 6 | 50 | 424 | Secondary | General | 4 | 4 |
| Liaoning | 5615 | 319 | 4 | 24 | Dalian | 3 | 6 | 50 | 425 | Tertiary | General | 10 | 10 |
| Liaoning | 5615 | 319 | 4 | 24 | Dalian | 3 | 6 | 50 | 423 | Primary | General | 2 | 2 |
| Heilongjiang | 3956 | 346 | 6 | 28 | Daqing | 2 | 5 | 121 | 233 | Secondary | General | 12 | 12 |
| Heilongjiang | 3956 | 346 | 6 | 28 | Daqing | 2 | 5 | 121 | 235 | Secondary | General | 7 | 7 |
| Heilongjiang | 3956 | 346 | 6 | 28 | Daqing | 2 | 5 | 121 | 234 | Tertiary | General | 46 | 46 |
| Heilongjiang | 3956 | 346 | 6 | 28 | Daqing | 2 | 5 | 121 | 236 | Tertiary | General | 46 | 46 |
| Shanxi | 4442 | 388 | 6 | 33 | Datong | 2 | 5 | 83 | 542 | Tertiary | General | 35 | 35 |
| Shanxi | 4442 | 388 | 6 | 33 | Datong | 2 | 5 | 83 | 540 | Tertiary | General | 19 | 19 |
| Hainan | 969 | 134 | 3 | 10 | Danzhou | 1 | 2 | 23 | 159 | Secondary | General | 20 | 20 |
| Hainan | 969 | 134 | 3 | 10 | Danzhou | 1 | 2 | 23 | 160 | Primary | General | 3 | 3 |
| Sichuan | 8864 | 743 | 6 | 28 | Deyang | 2 | 5 | 244 | 629 | Secondary | Specialized | 35 | 35 |
| Sichuan | 8864 | 743 | 6 | 28 | Deyang | 2 | 5 | 244 | 633 | Tertiary | Specialized | 85 | 85 |
| Sichuan | 8864 | 743 | 6 | 28 | Deyang | 2 | 5 | 244 | 631 | Secondary | General | 62 | 62 |
| Sichuan | 8864 | 743 | 6 | 28 | Deyang | 2 | 5 | 244 | 630 | Tertiary | General | 45 | 45 |
| Hubei | 6554 | 376 | 6 | 32 | Ezhou | 1 | 5 | 34 | 263 | Secondary | General | 11 | 11 |
| Hubei | 6554 | 376 | 6 | 32 | Ezhou | 1 | 5 | 34 | 265 | Tertiary | General | 4 | 4 |
| Liaoning | 5615 | 319 | 4 | 24 | Fuxin | 2 | 6 | 68 | 427 | Secondary | Specialized | 28 | 28 |
| Liaoning | 5615 | 319 | 4 | 24 | Fuxin | 2 | 6 | 68 | 428 | Secondary | General | 11 | 11 |
| Liaoning | 5615 | 319 | 4 | 24 | Fuxin | 2 | 6 | 68 | 426 | Tertiary | General | 12 | 12 |
| Liaoning | 5615 | 319 | 4 | 24 | Fuxin | 2 | 6 | 68 | 430 | Tertiary | General | 10 | 10 |
| Anhui | 5363 | 342 | 6 | 25 | Fuyang | 3 | 4 | 68 | 14 | Secondary | General | 12 | 12 |
| Anhui | 5363 | 342 | 6 | 25 | Fuyang | 3 | 4 | 68 | 15 | Tertiary | General | 38 | 38 |
| Anhui | 5363 | 342 | 6 | 25 | Fuyang | 3 | 4 | 68 | 16 | Tertiary | General | 10 | 10 |
| Ningxia | 885 | 221 | 5 | 21 | Guyuan | 1 | 4 | 47 | 467 | Primary | Specialized | 7 | 7 |
| Ningxia | 885 | 221 | 5 | 21 | Guyuan | 1 | 4 | 47 | 465 | Secondary | General | 15 | 15 |
| Ningxia | 885 | 221 | 5 | 21 | Guyuan | 1 | 4 | 47 | 466 | Secondary | General | 6 | 6 |
| Ningxia | 885 | 221 | 5 | 21 | Guyuan | 1 | 4 | 47 | 464 | Primary | General | 19 | 19 |
| Sichuan | 8864 | 743 | 6 | 28 | Guang'an | 2 | 5 | 62 | 637 | Secondary | General | 8 | 8 |
| Sichuan | 8864 | 743 | 6 | 28 | Guang'an | 2 | 5 | 62 | 638 | Secondary | General | 13 | 13 |
| Sichuan | 8864 | 743 | 6 | 28 | Guang'an | 2 | 5 | 62 | 634 | Tertiary | General | 21 | 21 |
| Guangdong | 11859 | 490 | 6 | 19 | Guangzhou | 3 | 4 | 191 | 89 | Tertiary | Specialized | 105 | 105 |
| Guangdong | 11859 | 490 | 6 | 19 | Guangzhou | 3 | 4 | 191 | 91 | Secondary | General | 47 | 47 |
| Guangdong | 11859 | 490 | 6 | 19 | Guangzhou | 3 | 4 | 191 | 92 | Tertiary | General | 35 | 35 |
| Guizhou | 3377 | 613 | 6 | 32 | Guiyang | 3 | 6 | 120 | 138 | Secondary | General | 15 | 15 |
| Guizhou | 3377 | 613 | 6 | 32 | Guiyang | 3 | 6 | 120 | 140 | Secondary | General | 20 | 20 |
| Guizhou | 3377 | 613 | 6 | 32 | Guiyang | 3 | 6 | 120 | 137 | Tertiary | General | 26 | 26 |
| Guizhou | 3377 | 613 | 6 | 32 | Guiyang | 3 | 6 | 120 | 139 | Tertiary | General | 40 | 40 |
| Guizhou | 3377 | 613 | 6 | 32 | Guiyang | 3 | 6 | 120 | 141 | Primary | General | 11 | 11 |
| Guizhou | 3377 | 613 | 6 | 32 | Guiyang | 3 | 6 | 120 | 142 | Primary | General | 8 | 8 |
| Heilongjiang | 3956 | 346 | 6 | 28 | Harbin | 3 | 6 | 76 | 238 | Primary | Specialized | 7 | 7 |
| Heilongjiang | 3956 | 346 | 6 | 28 | Harbin | 3 | 6 | 76 | 243 | Primary | Specialized | 10 | 10 |
| Heilongjiang | 3956 | 346 | 6 | 28 | Harbin | 3 | 6 | 76 | 239 | Secondary | General | 9 | 9 |
| Heilongjiang | 3956 | 346 | 6 | 28 | Harbin | 3 | 6 | 76 | 240 | Tertiary | General | 37 | 37 |
| Xinjiang | 2686 | 231 | 6 | 24 | Hami | 1 | 4 | 57 | 688 | Primary | Specialized | 13 | 13 |
| Xinjiang | 2686 | 231 | 6 | 24 | Hami | 1 | 4 | 57 | 689 | Secondary | General | 24 | 24 |
| Xinjiang | 2686 | 231 | 6 | 24 | Hami | 1 | 4 | 57 | 690 | Secondary | General | 14 | 14 |
| Xinjiang | 2686 | 231 | 6 | 24 | Hami | 1 | 4 | 57 | 687 | Primary | General | 6 | 6 |
| Qinghai | 710 | 246 | 6 | 25 | Haibei | 1 | 2 | 7 | 485 | Secondary | General | 6 | 6 |
| Qinghai | 710 | 246 | 6 | 25 | Haidong | 1 | 4 | 22 | 487 | Secondary | General | 4 | 4 |
| Qinghai | 710 | 246 | 6 | 25 | Haidong | 1 | 4 | 22 | 488 | Secondary | General | 13 | 13 |
| Qinghai | 710 | 246 | 6 | 25 | Haidong | 1 | 4 | 22 | 489 | Primary | General | 1 | 1 |
| Qinghai | 710 | 246 | 6 | 25 | Haidong | 1 | 4 | 22 | 490 | Primary | General | 4 | 4 |
| Hainan | 969 | 134 | 3 | 10 | Haikou | 2 | 3 | 61 | 163 | Tertiary | General | 17 | 17 |
| Qinghai | 710 | 246 | 6 | 25 | Hainan | 1 | 4 | 41 | 491 | Secondary | General | 8 | 8 |
| Qinghai | 710 | 246 | 6 | 25 | Hainan | 1 | 4 | 41 | 492 | Secondary | General | 22 | 22 |
| Qinghai | 710 | 246 | 6 | 25 | Hainan | 1 | 4 | 41 | 493 | Primary | General | 6 | 6 |
| Qinghai | 710 | 246 | 6 | 25 | Hainan | 1 | 4 | 41 | 494 | Primary | General | 5 | 5 |
| Qinghai | 710 | 246 | 6 | 25 | Haixi | 1 | 4 | 21 | 496 | Primary | Specialized | 10 | 10 |
| Qinghai | 710 | 246 | 6 | 25 | Haixi | 1 | 4 | 21 | 497 | Secondary | General | 8 | 8 |
| Qinghai | 710 | 246 | 6 | 25 | Haixi | 1 | 4 | 21 | 498 | Secondary | General | 2 | 2 |
| Hebei | 8525 | 687 | 6 | 33 | Handan | 3 | 5 | 91 | 186 | Primary | Specialized | 14 | 14 |
| Hebei | 8525 | 687 | 6 | 33 | Handan | 3 | 5 | 91 | 187 | Secondary | General | 14 | 14 |
| Hebei | 8525 | 687 | 6 | 33 | Handan | 3 | 5 | 91 | 190 | Secondary | General | 25 | 25 |
| Hebei | 8525 | 687 | 6 | 33 | Handan | 3 | 5 | 91 | 188 | Tertiary | General | 15 | 15 |
| Hebei | 8525 | 687 | 6 | 33 | Handan | 3 | 5 | 91 | 189 | Tertiary | General | 23 | 23 |
| Hunan | 7212 | 477 | 6 | 33 | Hengyang | 3 | 6 | 76 | 303 | Primary | Specialized | 8 | 8 |
| Hunan | 7212 | 477 | 6 | 33 | Hengyang | 3 | 6 | 76 | 300 | Secondary | General | 7 | 7 |
| Hunan | 7212 | 477 | 6 | 33 | Hengyang | 3 | 6 | 76 | 301 | Secondary | General | 7 | 7 |
| Hunan | 7212 | 477 | 6 | 33 | Hengyang | 3 | 6 | 76 | 299 | Tertiary | General | 20 | 20 |
| Hunan | 7212 | 477 | 6 | 33 | Hengyang | 3 | 6 | 76 | 302 | Tertiary | General | 28 | 28 |
| Hunan | 7212 | 477 | 6 | 33 | Hengyang | 3 | 6 | 76 | 304 | Primary | General | 6 | 6 |
| Inner Mongolia | 3274 | 203 | 6 | 26 | Hohhot | 2 | 4 | 49 | 452 | Primary | Specialized | 8 | 8 |
| Inner Mongolia | 3274 | 203 | 6 | 26 | Hohhot | 2 | 4 | 49 | 454 | Tertiary | General | 18 | 18 |
| Inner Mongolia | 3274 | 203 | 6 | 26 | Hohhot | 2 | 4 | 49 | 455 | Tertiary | General | 20 | 20 |
| Anhui | 5363 | 342 | 6 | 25 | Huainan | 2 | 6 | 70 | 21 | Tertiary | General | 32 | 32 |
| Anhui | 5363 | 342 | 6 | 25 | Huainan | 2 | 6 | 70 | 23 | Tertiary | General | 11 | 11 |
| Anhui | 5363 | 342 | 6 | 25 | Huainan | 2 | 6 | 70 | 19 | Primary | General | 6 | 6 |
| Hubei | 6554 | 376 | 6 | 32 | Huangshi | 2 | 6 | 76 | 271 | Secondary | General | 8 | 8 |
| Hubei | 6554 | 376 | 6 | 32 | Huangshi | 2 | 6 | 76 | 268 | Tertiary | General | 32 | 32 |
| Hubei | 6554 | 376 | 6 | 32 | Huangshi | 2 | 6 | 76 | 269 | Tertiary | General | 12 | 12 |
| Hubei | 6554 | 376 | 6 | 32 | Huangshi | 2 | 6 | 76 | 267 | Primary | General | 5 | 5 |
| Shandong | 12695 | 512 | 6 | 30 | Jinan | 3 | 5 | 134 | 514 | Primary | Specialized | 34 | 34 |
| Shandong | 12695 | 512 | 6 | 30 | Jinan | 3 | 5 | 134 | 510 | Secondary | General | 13 | 13 |
| Shandong | 12695 | 512 | 6 | 30 | Jinan | 3 | 5 | 134 | 511 | Tertiary | General | 23 | 23 |
| Heilongjiang | 3956 | 346 | 6 | 28 | Jiamusi | 2 | 5 | 61 | 245 | Tertiary | Specialized | 24 | 24 |
| Heilongjiang | 3956 | 346 | 6 | 28 | Jiamusi | 2 | 5 | 61 | 247 | Tertiary | General | 16 | 16 |
| Zhejiang | 8597 | 745 | 6 | 36 | Jiaxing | 2 | 6 | 96 | 730 | Primary | Specialized | 26 | 26 |
| Zhejiang | 8597 | 745 | 6 | 36 | Jiaxing | 2 | 6 | 96 | 729 | Secondary | General | 23 | 23 |
| Zhejiang | 8597 | 745 | 6 | 36 | Jiaxing | 2 | 6 | 96 | 734 | Secondary | General | 15 | 15 |
| Zhejiang | 8597 | 745 | 6 | 36 | Jiaxing | 2 | 6 | 96 | 731 | Tertiary | General | 9 | 9 |
| Zhejiang | 8597 | 745 | 6 | 36 | Jiaxing | 2 | 6 | 96 | 733 | Tertiary | General | 17 | 17 |
| Zhejiang | 8597 | 745 | 6 | 36 | Jiaxing | 2 | 6 | 96 | 732 | Primary | General | 6 | 6 |
| Henan | 9336 | 416 | 6 | 31 | Jiaozuo | 2 | 5 | 44 | 210 | Primary | Specialized | 12 | 12 |
| Henan | 9336 | 416 | 6 | 31 | Jiaozuo | 2 | 5 | 44 | 208 | Secondary | General | 14 | 14 |
| Guangdong | 11859 | 490 | 6 | 19 | Ji eyang | 2 | 4 | 38 | 93 | Secondary | General | 20 | 20 |
| Guangdong | 11859 | 490 | 6 | 19 | Ji eyang | 2 | 4 | 38 | 96 | Tertiary | General | 11 | 11 |
| Zhejiang | 8597 | 745 | 6 | 36 | Jinhua | 2 | 6 | 125 | 737 | Primary | Specialized | 12 | 12 |
| Zhejiang | 8597 | 745 | 6 | 36 | Jinhua | 2 | 6 | 125 | 739 | Secondary | General | 11 | 11 |
| Zhejiang | 8597 | 745 | 6 | 36 | Jinhua | 2 | 6 | 125 | 740 | Secondary | General | 11 | 11 |
| Zhejiang | 8597 | 745 | 6 | 36 | Jinhua | 2 | 6 | 125 | 735 | Tertiary | General | 51 | 51 |
| Zhejiang | 8597 | 745 | 6 | 36 | Jinhua | 2 | 6 | 125 | 736 | Tertiary | General | 31 | 31 |
| Shanxi | 4442 | 388 | 6 | 33 | Jincheng | 1 | 4 | 56 | 548 | Primary | Specialized | 18 | 18 |
| Shanxi | 4442 | 388 | 6 | 33 | Jincheng | 1 | 4 | 56 | 545 | Tertiary | General | 13 | 13 |
| Shanxi | 4442 | 388 | 6 | 33 | Jincheng | 1 | 4 | 56 | 547 | Tertiary | General | 19 | 19 |
| Hubei | 6554 | 376 | 6 | 32 | Jingmen | 2 | 5 | 93 | 273 | Secondary | General | 24 | 24 |
| Hubei | 6554 | 376 | 6 | 32 | Jingmen | 2 | 5 | 93 | 275 | Secondary | General | 20 | 20 |
| Hubei | 6554 | 376 | 6 | 32 | Jingmen | 2 | 5 | 93 | 274 | Tertiary | General | 41 | 41 |
| Hubei | 6554 | 376 | 6 | 32 | Jingmen | 2 | 5 | 93 | 272 | Primary | General | 6 | 6 |
| Hubei | 6554 | 376 | 6 | 32 | Jingmen | 2 | 5 | 93 | 276 | Primary | General | 2 | 2 |
| Jiangxi | 3793 | 384 | 5 | 26 | Jiujiang | 2 | 6 | 159 | 391 | Tertiary | Specialized | 93 | 93 |
| Jiangxi | 3793 | 384 | 5 | 26 | Jiujiang | 2 | 6 | 159 | 393 | Primary | Specialized | 15 | 15 |
| Jiangxi | 3793 | 384 | 5 | 26 | Jiujiang | 2 | 6 | 159 | 392 | Secondary | General | 4 | 4 |
| Jiangxi | 3793 | 384 | 5 | 26 | Jiujiang | 2 | 6 | 159 | 390 | Tertiary | General | 22 | 22 |
| Xinjiang | 2686 | 231 | 6 | 24 | Karamay | 1 | 3 | 43 | 692 | Secondary | General | 16 | 16 |
| Xinjiang | 2686 | 231 | 6 | 24 | Karamay | 1 | 3 | 43 | 693 | Secondary | General | 11 | 11 |
| Xinjiang | 2686 | 231 | 6 | 24 | Karamay | 1 | 3 | 43 | 691 | Tertiary | General | 16 | 16 |
| Yunnan | 4256 | 263 | 6 | 22 | Kunming | 3 | 4 | 49 | 707 | Primary | Specialized | 5 | 5 |
| Yunnan | 4256 | 263 | 6 | 22 | Kunming | 3 | 4 | 49 | 709 | Primary | Specialized | 9 | 9 |
| Yunnan | 4256 | 263 | 6 | 22 | Kunming | 3 | 4 | 49 | 708 | Tertiary | General | 10 | 10 |
| Yunnan | 4256 | 263 | 6 | 22 | Kunming | 3 | 4 | 49 | 710 | Tertiary | General | 25 | 25 |
| Tibet | 322 | 177 | 6 | 17 | Lasa | 1 | 3 | 48 | 672 | Secondary | General | 15 | 15 |
| Zhejiang | 8597 | 745 | 6 | 36 | Lishui | 2 | 6 | 138 | 743 | Secondary | General | 21 | 21 |
| Zhejiang | 8597 | 745 | 6 | 36 | Lishui | 2 | 6 | 138 | 744 | Secondary | General | 12 | 12 |
| Zhejiang | 8597 | 745 | 6 | 36 | Lishui | 2 | 6 | 138 | 741 | Tertiary | General | 44 | 44 |
| Zhejiang | 8597 | 745 | 6 | 36 | Lishui | 2 | 6 | 138 | 742 | Tertiary | General | 45 | 45 |
| Jiangsu | 10033 | 740 | 6 | 36 | Lianyungang | 2 | 6 | 89 | 353 | Secondary | General | 12 | 12 |
| Jiangsu | 10033 | 740 | 6 | 36 | Lianyungang | 2 | 6 | 89 | 354 | Secondary | General | 6 | 6 |
| Jiangsu | 10033 | 740 | 6 | 36 | Lianyungang | 2 | 6 | 89 | 356 | Tertiary | General | 33 | 33 |
| Jiangsu | 10033 | 740 | 6 | 36 | Lianyungang | 2 | 6 | 89 | 357 | Tertiary | General | 20 | 20 |
| Jiangsu | 10033 | 740 | 6 | 36 | Lianyungang | 2 | 6 | 89 | 352 | Primary | General | 12 | 12 |
| Jiangsu | 10033 | 740 | 6 | 36 | Lianyungang | 2 | 6 | 89 | 355 | Primary | General | 6 | 6 |
| Jilin | 3484 | 232 | 5 | 26 | Liaoyuan | 1 | 5 | 38 | 334 | Tertiary | General | 9 | 9 |
| Jilin | 3484 | 232 | 5 | 26 | Liaoyuan | 1 | 5 | 38 | 331 | Primary | General | 11 | 11 |
| Shandong | 12695 | 512 | 6 | 30 | Liaocheng | 2 | 5 | 64 | 517 | Secondary | General | 16 | 16 |
| Shandong | 12695 | 512 | 6 | 30 | Liaocheng | 2 | 5 | 64 | 518 | Tertiary | General | 30 | 30 |
| Shandong | 12695 | 512 | 6 | 30 | Liaocheng | 2 | 5 | 64 | 519 | Tertiary | General | 10 | 10 |
| Tibet | 322 | 177 | 6 | 17 | Linzhi | 1 | 4 | 16 | 674 | Primary | Specialized | 9 | 9 |
| Tibet | 322 | 177 | 6 | 17 | Linzhi | 1 | 4 | 16 | 673 | Secondary | General | 3 | 3 |
| Tibet | 322 | 177 | 6 | 17 | Linzhi | 1 | 4 | 16 | 675 | Secondary | General | 2 | 2 |
| Tibet | 322 | 177 | 6 | 17 | Linzhi | 1 | 4 | 16 | 676 | Primary | General | 2 | 2 |
| Yunnan | 4256 | 263 | 6 | 22 | Lincang | 1 | 4 | 43 | 714 | Tertiary | General | 25 | 25 |
| Shanxi | 4442 | 388 | 6 | 33 | Lin fen | 2 | 6 | 61 | 550 | Secondary | General | 11 | 11 |
| Guangxi | 4478 | 285 | 6 | 19 | Liuzhou | 2 | 1 | 23 | 120 | Tertiary | General | 23 | 23 |
| Guizhou | 3377 | 613 | 6 | 32 | Liupanshui | 2 | 6 | 197 | 145 | Secondary | General | 19 | 19 |
| Guizhou | 3377 | 613 | 6 | 32 | Liupanshui | 2 | 6 | 197 | 148 | Secondary | General | 24 | 24 |
| Guizhou | 3377 | 613 | 6 | 32 | Liupanshui | 2 | 6 | 197 | 143 | Tertiary | General | 64 | 64 |
| Guizhou | 3377 | 613 | 6 | 32 | Liupanshui | 2 | 6 | 197 | 144 | Tertiary | General | 68 | 68 |
| Guizhou | 3377 | 613 | 6 | 32 | Liupanshui | 2 | 6 | 197 | 146 | Primary | General | 11 | 11 |
| Guizhou | 3377 | 613 | 6 | 32 | Liupanshui | 2 | 6 | 197 | 147 | Primary | General | 11 | 11 |
| Henan | 9336 | 416 | 6 | 31 | Luoyang | 3 | 5 | 64 | 215 | Secondary | General | 12 | 12 |
| Henan | 9336 | 416 | 6 | 31 | Luoyang | 3 | 5 | 64 | 212 | Tertiary | General | 30 | 30 |
| Henan | 9336 | 416 | 6 | 31 | Luoyang | 3 | 5 | 64 | 213 | Tertiary | General | 8 | 8 |
| Henan | 9336 | 416 | 6 | 31 | Luoyang | 3 | 5 | 64 | 216 | Primary | General | 8 | 8 |
| Henan | 9336 | 416 | 6 | 31 | Luohe | 2 | 6 | 98 | 218 | Secondary | Specialized | 27 | 27 |
| Henan | 9336 | 416 | 6 | 31 | Luohe | 2 | 6 | 98 | 221 | Secondary | General | 10 | 10 |
| Henan | 9336 | 416 | 6 | 31 | Luohe | 2 | 6 | 98 | 220 | Tertiary | General | 28 | 28 |
| Shanxi | 4442 | 388 | 6 | 33 | Lvliang | 1 | 6 | 79 | 560 | Secondary | Specialized | 15 | 15 |
| Shanxi | 4442 | 388 | 6 | 33 | Lvliang | 1 | 6 | 79 | 555 | Secondary | General | 12 | 12 |
| Shanxi | 4442 | 388 | 6 | 33 | Lvliang | 1 | 6 | 79 | 556 | Tertiary | General | 21 | 21 |
| Shanxi | 4442 | 388 | 6 | 33 | Lvliang | 1 | 6 | 79 | 557 | Tertiary | General | 16 | 16 |
| Anhui | 5363 | 342 | 6 | 25 | Maanshan | 1 | 2 | 14 | 24 | Primary | General | 12 | 12 |
| Sichuan | 8864 | 743 | 6 | 28 | Meishan | 2 | 5 | 51 | 639 | Primary | Specialized | 10 | 10 |
| Sichuan | 8864 | 743 | 6 | 28 | Meishan | 2 | 5 | 51 | 641 | Secondary | General | 6 | 6 |
| Sichuan | 8864 | 743 | 6 | 28 | Meishan | 2 | 5 | 51 | 640 | Tertiary | General | 16 | 16 |
| Sichuan | 8864 | 743 | 6 | 28 | Mianyang | 2 | 5 | 155 | 646 | Secondary | Specialized | 39 | 39 |
| Sichuan | 8864 | 743 | 6 | 28 | Mianyang | 2 | 5 | 155 | 644 | Primary | Specialized | 16 | 16 |
| Sichuan | 8864 | 743 | 6 | 28 | Mianyang | 2 | 5 | 155 | 645 | Secondary | General | 26 | 26 |
| Sichuan | 8864 | 743 | 6 | 28 | Mianyang | 2 | 5 | 155 | 647 | Tertiary | General | 64 | 64 |
| Sichuan | 8864 | 743 | 6 | 28 | Mianyang | 2 | 5 | 155 | 648 | Tertiary | General | 10 | 10 |
| Tibet | 322 | 177 | 6 | 17 | Naqu | 1 | 2 | 9 | 678 | Primary | Specialized | 8 | 8 |
| Jiangxi | 3793 | 384 | 5 | 26 | Nanchang | 2 | 6 | 56 | 399 | Secondary | General | 8 | 8 |
| Jiangxi | 3793 | 384 | 5 | 26 | Nanchang | 2 | 6 | 56 | 397 | Primary | General | 4 | 4 |
| Jiangsu | 10033 | 740 | 6 | 36 | Nanjing | 3 | 6 | 214 | 359 | Secondary | General | 10 | 10 |
| Jiangsu | 10033 | 740 | 6 | 36 | Nanjing | 3 | 6 | 214 | 361 | Secondary | General | 15 | 15 |
| Jiangsu | 10033 | 740 | 6 | 36 | Nanjing | 3 | 6 | 214 | 358 | Tertiary | General | 70 | 70 |
| Jiangsu | 10033 | 740 | 6 | 36 | Nanjing | 3 | 6 | 214 | 362 | Primary | General | 10 | 10 |
| Fujian | 4074 | 244 | 5 | 20 | Ningde | 2 | 1 | 4 | 50 | Tertiary | General | 4 | 4 |
| Jiangxi | 3793 | 384 | 5 | 26 | Pingxiang | 1 | 5 | 57 | 400 | Secondary | General | 13 | 13 |
| Jiangxi | 3793 | 384 | 5 | 26 | Pingxiang | 1 | 5 | 57 | 401 | Tertiary | General | 12 | 12 |
| Jiangxi | 3793 | 384 | 5 | 26 | Pingxiang | 1 | 5 | 57 | 403 | Tertiary | General | 19 | 19 |
| Jiangxi | 3793 | 384 | 5 | 26 | Pingxiang | 1 | 5 | 57 | 402 | Primary | General | 6 | 6 |
| Jiangxi | 3793 | 384 | 5 | 26 | Pingxiang | 1 | 5 | 57 | 404 | Primary | General | 7 | 7 |
| Fujian | 4074 | 244 | 5 | 20 | Putian | 2 | 6 | 185 | 55 | Primary | Specialized | 18 | 18 |
| Fujian | 4074 | 244 | 5 | 20 | Putian | 2 | 6 | 185 | 51 | Secondary | General | 18 | 18 |
| Fujian | 4074 | 244 | 5 | 20 | Putian | 2 | 6 | 185 | 54 | Tertiary | General | 72 | 72 |
| Fujian | 4074 | 244 | 5 | 20 | Putian | 2 | 6 | 185 | 52 | Primary | General | 9 | 9 |
| Fujian | 4074 | 244 | 5 | 20 | Putian | 2 | 6 | 185 | 56 | Primary | General | 17 | 17 |
| Yunnan | 4256 | 263 | 6 | 22 | Pu'er | 2 | 2 | 17 | 715 | Secondary | General | 12 | 12 |
| Hebei | 8525 | 687 | 6 | 33 | Qinhuangdao | 2 | 6 | 106 | 196 | Secondary | General | 10 | 10 |
| Hebei | 8525 | 687 | 6 | 33 | Qinhuangdao | 2 | 6 | 106 | 194 | Tertiary | General | 25 | 25 |
| Shandong | 12695 | 512 | 6 | 30 | Qingdao | 3 | 5 | 69 | 521 | Primary | Specialized | 15 | 15 |
| Shandong | 12695 | 512 | 6 | 30 | Qingdao | 3 | 5 | 69 | 520 | Secondary | General | 19 | 19 |
| Shandong | 12695 | 512 | 6 | 30 | Qingdao | 3 | 5 | 69 | 522 | Secondary | General | 10 | 10 |
| Gansu | 2492 | 181 | 6 | 23 | Qingyang | 1 | 5 | 37 | 73 | Tertiary | General | 14 | 14 |
| Gansu | 2492 | 181 | 6 | 23 | Qingyang | 1 | 5 | 37 | 75 | Primary | General | 5 | 5 |
| Gansu | 2492 | 181 | 6 | 23 | Qingyang | 1 | 5 | 37 | 76 | Primary | General | 4 | 4 |
| Zhejiang | 8597 | 745 | 6 | 36 | Quzhou | 2 | 6 | 82 | 748 | Secondary | General | 15 | 15 |
| Zhejiang | 8597 | 745 | 6 | 36 | Quzhou | 2 | 6 | 82 | 752 | Secondary | General | 11 | 11 |
| Zhejiang | 8597 | 745 | 6 | 36 | Quzhou | 2 | 6 | 82 | 747 | Tertiary | General | 17 | 17 |
| Zhejiang | 8597 | 745 | 6 | 36 | Quzhou | 2 | 6 | 82 | 751 | Tertiary | General | 23 | 23 |
| Yunnan | 4256 | 263 | 6 | 22 | Qujing | 2 | 3 | 30 | 718 | Secondary | General | 23 | 23 |
| Tibet | 322 | 177 | 6 | 17 | Xigaze | 1 | 2 | 18 | 680 | Secondary | General | 14 | 14 |
| Tibet | 322 | 177 | 6 | 17 | Xigaze | 1 | 2 | 18 | 679 | Primary | General | 4 | 4 |
| Henan | 9336 | 416 | 6 | 31 | Sanmenxia | 2 | 4 | 60 | 226 | Tertiary | General | 25 | 25 |
| Henan | 9336 | 416 | 6 | 31 | Sanmenxia | 2 | 4 | 60 | 223 | Primary | General | 8 | 8 |
| Henan | 9336 | 416 | 6 | 31 | Sanmenxia | 2 | 4 | 60 | 224 | Primary | General | 12 | 12 |
| Fujian | 4074 | 244 | 5 | 20 | Sanming | 2 | 6 | 32 | 62 | Primary | Specialized | 8 | 8 |
| Fujian | 4074 | 244 | 5 | 20 | Sanming | 2 | 6 | 32 | 60 | Secondary | General | 6 | 6 |
| Hainan | 969 | 134 | 3 | 10 | Sanya | 1 | 5 | 50 | 166 | Primary | Specialized | 10 | 10 |
| Hainan | 969 | 134 | 3 | 10 | Sanya | 1 | 5 | 50 | 167 | Secondary | General | 17 | 17 |
| Hainan | 969 | 134 | 3 | 10 | Sanya | 1 | 5 | 50 | 168 | Secondary | General | 10 | 10 |
| Hainan | 969 | 134 | 3 | 10 | Sanya | 1 | 5 | 50 | 165 | Primary | General | 3 | 3 |
| Tibet | 322 | 177 | 6 | 17 | Shannan | 1 | 2 | 10 | 681 | Secondary | General | 2 | 2 |
| Tibet | 322 | 177 | 6 | 17 | Shannan | 1 | 2 | 10 | 682 | Secondary | General | 8 | 8 |
| Shaanxi | 4289 | 429 | 6 | 27 | Shangluo | 2 | 4 | 57 | 581 | Primary | Specialized | 29 | 29 |
| Shaanxi | 4289 | 429 | 6 | 27 | Shangluo | 2 | 4 | 57 | 578 | Secondary | General | 10 | 10 |
| Shaanxi | 4289 | 429 | 6 | 27 | Shangluo | 2 | 4 | 57 | 580 | Secondary | General | 6 | 6 |
| Shanghai | 3508 | 345 | 1 | 25 | Shanghai | 3 | 25 | 345 | 623 | Secondary | Specialized | 45 | 45 |
| Shanghai | 3508 | 345 | 1 | 25 | Shanghai | 3 | 25 | 345 | 602 | Secondary | General | 8 | 8 |
| Shanghai | 3508 | 345 | 1 | 25 | Shanghai | 3 | 25 | 345 | 609 | Secondary | General | 16 | 16 |
| Shanghai | 3508 | 345 | 1 | 25 | Shanghai | 3 | 25 | 345 | 610 | Secondary | General | 20 | 20 |
| Shanghai | 3508 | 345 | 1 | 25 | Shanghai | 3 | 25 | 345 | 613 | Secondary | General | 6 | 6 |
| Shanghai | 3508 | 345 | 1 | 25 | Shanghai | 3 | 25 | 345 | 616 | Secondary | General | 10 | 10 |
| Shanghai | 3508 | 345 | 1 | 25 | Shanghai | 3 | 25 | 345 | 622 | Secondary | General | 10 | 10 |
| Shanghai | 3508 | 345 | 1 | 25 | Shanghai | 3 | 25 | 345 | 624 | Secondary | General | 8 | 8 |
| Shanghai | 3508 | 345 | 1 | 25 | Shanghai | 3 | 25 | 345 | 605 | Tertiary | General | 35 | 35 |
| Shanghai | 3508 | 345 | 1 | 25 | Shanghai | 3 | 25 | 345 | 606 | Tertiary | General | 18 | 18 |
| Shanghai | 3508 | 345 | 1 | 25 | Shanghai | 3 | 25 | 345 | 607 | Tertiary | General | 33 | 33 |
| Shanghai | 3508 | 345 | 1 | 25 | Shanghai | 3 | 25 | 345 | 608 | Tertiary | General | 28 | 28 |
| Shanghai | 3508 | 345 | 1 | 25 | Shanghai | 3 | 25 | 345 | 614 | Tertiary | General | 11 | 11 |
| Shanghai | 3508 | 345 | 1 | 25 | Shanghai | 3 | 25 | 345 | 615 | Tertiary | General | 31 | 31 |
| Shanghai | 3508 | 345 | 1 | 25 | Shanghai | 3 | 25 | 345 | 619 | Tertiary | General | 6 | 6 |
| Shanghai | 3508 | 345 | 1 | 25 | Shanghai | 3 | 25 | 345 | 621 | Tertiary | General | 15 | 15 |
| Shanghai | 3508 | 345 | 1 | 25 | Shanghai | 3 | 25 | 345 | 620 | Primary | General | 2 | 2 |
| Guangdong | 11859 | 490 | 6 | 19 | Shaoguan | 2 | 5 | 87 | 97 | Secondary | General | 12 | 12 |
| Guangdong | 11859 | 490 | 6 | 19 | Shaoguan | 2 | 5 | 87 | 101 | Secondary | General | 9 | 9 |
| Guangdong | 11859 | 490 | 6 | 19 | Shaoguan | 2 | 5 | 87 | 99 | Tertiary | General | 18 | 18 |
| Guangdong | 11859 | 490 | 6 | 19 | Shaoguan | 2 | 5 | 87 | 100 | Tertiary | General | 45 | 45 |
| Xinjiang | 2686 | 231 | 6 | 24 | Shihezi | 1 | 5 | 80 | 697 | Secondary | General | 35 | 35 |
| Xinjiang | 2686 | 231 | 6 | 24 | Shihezi | 1 | 5 | 80 | 696 | Tertiary | General | 35 | 35 |
| Xinjiang | 2686 | 231 | 6 | 24 | Shihezi | 1 | 5 | 80 | 694 | Primary | General | 1 | 1 |
| Xinjiang | 2686 | 231 | 6 | 24 | Shihezi | 1 | 5 | 80 | 695 | Primary | General | 2 | 2 |
| Ningxia | 885 | 221 | 5 | 21 | Shizuishan | 1 | 4 | 44 | 468 | Secondary | General | 17 | 17 |
| Ningxia | 885 | 221 | 5 | 21 | Shizuishan | 1 | 4 | 44 | 469 | Secondary | General | 16 | 16 |
| Heilongjiang | 3956 | 346 | 6 | 28 | Shuangyashan | 1 | 4 | 31 | 250 | Secondary | General | 10 | 10 |
| Heilongjiang | 3956 | 346 | 6 | 28 | Shuangyashan | 1 | 4 | 31 | 249 | Primary | General | 7 | 7 |
| Jilin | 3484 | 232 | 5 | 26 | Siping | 2 | 5 | 52 | 336 | Primary | Specialized | 12 | 12 |
| Jilin | 3484 | 232 | 5 | 26 | Siping | 2 | 5 | 52 | 335 | Secondary | General | 9 | 9 |
| Jilin | 3484 | 232 | 5 | 26 | Siping | 2 | 5 | 52 | 338 | Tertiary | General | 11 | 11 |
| Heilongjiang | 3956 | 346 | 6 | 28 | Suihua | 2 | 4 | 48 | 254 | Secondary | General | 10 | 10 |
| Heilongjiang | 3956 | 346 | 6 | 28 | Suihua | 2 | 4 | 48 | 255 | Tertiary | General | 20 | 20 |
| Heilongjiang | 3956 | 346 | 6 | 28 | Suihua | 2 | 4 | 48 | 256 | Primary | General | 10 | 10 |
| Hubei | 6554 | 376 | 6 | 32 | Suizhou | 1 | 5 | 32 | 280 | Tertiary | General | 13 | 13 |
| Shandong | 12695 | 512 | 6 | 30 | Tai'an | 2 | 5 | 82 | 526 | Secondary | General | 11 | 11 |
| Shandong | 12695 | 512 | 6 | 30 | Tai'an | 2 | 5 | 82 | 528 | Tertiary | General | 50 | 50 |
| Shandong | 12695 | 512 | 6 | 30 | Tai'an | 2 | 5 | 82 | 529 | Tertiary | General | 11 | 11 |
| Jiangsu | 10033 | 740 | 6 | 36 | Taizhou | 2 | 6 | 78 | 364 | Secondary | General | 12 | 12 |
| Jiangsu | 10033 | 740 | 6 | 36 | Taizhou | 2 | 6 | 78 | 369 | Secondary | General | 11 | 11 |
| Jiangsu | 10033 | 740 | 6 | 36 | Taizhou | 2 | 6 | 78 | 367 | Tertiary | General | 28 | 28 |
| Jiangsu | 10033 | 740 | 6 | 36 | Taizhou | 2 | 6 | 78 | 368 | Tertiary | General | 16 | 16 |
| Tianjin | 2097 | 387 | 1 | 13 | Tianjin | 3 | 13 | 387 | 658 | Secondary | General | 111 | 111 |
| Tianjin | 2097 | 387 | 1 | 13 | Tianjin | 3 | 13 | 387 | 662 | Secondary | General | 21 | 21 |
| Tianjin | 2097 | 387 | 1 | 13 | Tianjin | 3 | 13 | 387 | 656 | Tertiary | General | 56 | 56 |
| Tianjin | 2097 | 387 | 1 | 13 | Tianjin | 3 | 13 | 387 | 663 | Tertiary | General | 113 | 113 |
| Tianjin | 2097 | 387 | 1 | 13 | Tianjin | 3 | 13 | 387 | 664 | Tertiary | General | 17 | 17 |
| Tianjin | 2097 | 387 | 1 | 13 | Tianjin | 3 | 13 | 387 | 665 | Tertiary | General | 20 | 20 |
| Tianjin | 2097 | 387 | 1 | 13 | Tianjin | 3 | 13 | 387 | 659 | Primary | General | 3 | 3 |
| Gansu | 2492 | 181 | 6 | 23 | Tianshui | 2 | 4 | 40 | 77 | Tertiary | General | 9 | 9 |
| Gansu | 2492 | 181 | 6 | 23 | Tianshui | 2 | 4 | 40 | 79 | Secondary | Specialized | 12 | 12 |
| Jilin | 3484 | 232 | 5 | 26 | Tonghua | 2 | 6 | 56 | 341 | Primary | Specialized | 11 | 11 |
| Jilin | 3484 | 232 | 5 | 26 | Tonghua | 2 | 6 | 56 | 342 | Secondary | General | 6 | 6 |
| Jilin | 3484 | 232 | 5 | 26 | Tonghua | 2 | 6 | 56 | 345 | Secondary | General | 8 | 8 |
| Jilin | 3484 | 232 | 5 | 26 | Tonghua | 2 | 6 | 56 | 343 | Tertiary | General | 12 | 12 |
| Jilin | 3484 | 232 | 5 | 26 | Tonghua | 2 | 6 | 56 | 344 | Tertiary | General | 13 | 13 |
| Guizhou | 3377 | 613 | 6 | 32 | Tongren | 2 | 5 | 89 | 149 | Secondary | General | 11 | 11 |
| Guizhou | 3377 | 613 | 6 | 32 | Tongren | 2 | 5 | 89 | 152 | Secondary | General | 13 | 13 |
| Guizhou | 3377 | 613 | 6 | 32 | Tongren | 2 | 5 | 89 | 150 | Tertiary | General | 46 | 46 |
| Guizhou | 3377 | 613 | 6 | 32 | Tongren | 2 | 5 | 89 | 151 | Primary | General | 12 | 12 |
| Xinjiang | 2686 | 231 | 6 | 24 | Turpan | 1 | 4 | 17 | 700 | Secondary | General | 9 | 9 |
| Shaanxi | 4289 | 429 | 6 | 27 | Weinan | 2 | 5 | 86 | 584 | Tertiary | Specialized | 40 | 40 |
| Shaanxi | 4289 | 429 | 6 | 27 | Weinan | 2 | 5 | 86 | 586 | Tertiary | General | 27 | 27 |
| Zhejiang | 8597 | 745 | 6 | 36 | Wenzhou | 3 | 6 | 178 | 755 | Primary | Specialized | 32 | 32 |
| Zhejiang | 8597 | 745 | 6 | 36 | Wenzhou | 3 | 6 | 178 | 753 | Secondary | General | 15 | 15 |
| Zhejiang | 8597 | 745 | 6 | 36 | Wenzhou | 3 | 6 | 178 | 756 | Tertiary | General | 95 | 95 |
| Zhejiang | 8597 | 745 | 6 | 36 | Wenzhou | 3 | 6 | 178 | 757 | Tertiary | General | 23 | 23 |
| Inner Mongolia | 3274 | 203 | 6 | 26 | Wuhai | 1 | 3 | 18 | 463 | Tertiary | General | 15 | 15 |
| Inner Mongolia | 3274 | 203 | 6 | 26 | Wuhai | 1 | 3 | 18 | 461 | Primary | General | 1 | 1 |
| Xinjiang | 2686 | 231 | 6 | 24 | Urumqi | 2 | 4 | 18 | 705 | Primary | General | 4 | 4 |
| Jiangsu | 10033 | 740 | 6 | 36 | Wuxi | 3 | 6 | 146 | 371 | Tertiary | Specialized | 31 | 31 |
| Jiangsu | 10033 | 740 | 6 | 36 | Wuxi | 3 | 6 | 146 | 373 | Secondary | General | 27 | 27 |
| Jiangsu | 10033 | 740 | 6 | 36 | Wuxi | 3 | 6 | 146 | 374 | Secondary | General | 45 | 45 |
| Jiangsu | 10033 | 740 | 6 | 36 | Wuxi | 3 | 6 | 146 | 372 | Tertiary | General | 21 | 21 |
| Jiangsu | 10033 | 740 | 6 | 36 | Wuxi | 3 | 6 | 146 | 370 | Primary | General | 15 | 15 |
| Jiangsu | 10033 | 740 | 6 | 36 | Wuxi | 3 | 6 | 146 | 375 | Primary | General | 7 | 7 |
| Ningxia | 885 | 221 | 5 | 21 | Wuzhong | 1 | 3 | 26 | 472 | Secondary | General | 11 | 11 |
| Ningxia | 885 | 221 | 5 | 21 | Wuzhong | 1 | 3 | 26 | 473 | Secondary | General | 6 | 6 |
| Ningxia | 885 | 221 | 5 | 21 | Wuzhong | 1 | 3 | 26 | 474 | Primary | General | 9 | 9 |
| Guangxi | 4478 | 285 | 6 | 19 | Wuzhou | 2 | 4 | 27 | 123 | Tertiary | General | 13 | 13 |
| Gansu | 2492 | 181 | 6 | 23 | Wuwei | 1 | 4 | 45 | 83 | Tertiary | General | 26 | 26 |
| Gansu | 2492 | 181 | 6 | 23 | Wuwei | 1 | 4 | 45 | 84 | Tertiary | General | 7 | 7 |
| Shaanxi | 4289 | 429 | 6 | 27 | Xi'an | 3 | 3 | 55 | 589 | Primary | Specialized | 8 | 8 |
| Shaanxi | 4289 | 429 | 6 | 27 | Xi'an | 3 | 3 | 55 | 587 | Tertiary | General | 21 | 21 |
| Shaanxi | 4289 | 429 | 6 | 27 | Xi'an | 3 | 3 | 55 | 588 | Tertiary | General | 26 | 26 |
| Qinghai | 710 | 246 | 6 | 25 | Xining | 2 | 7 | 128 | 501 | Secondary | General | 8 | 8 |
| Qinghai | 710 | 246 | 6 | 25 | Xining | 2 | 7 | 128 | 504 | Secondary | General | 8 | 8 |
| Qinghai | 710 | 246 | 6 | 25 | Xining | 2 | 7 | 128 | 499 | Tertiary | General | 19 | 19 |
| Qinghai | 710 | 246 | 6 | 25 | Xining | 2 | 7 | 128 | 500 | Tertiary | General | 38 | 38 |
| Qinghai | 710 | 246 | 6 | 25 | Xining | 2 | 7 | 128 | 502 | Tertiary | General | 22 | 22 |
| Qinghai | 710 | 246 | 6 | 25 | Xining | 2 | 7 | 128 | 503 | Tertiary | General | 12 | 12 |
| Qinghai | 710 | 246 | 6 | 25 | Xining | 2 | 7 | 128 | 505 | Tertiary | General | 21 | 21 |
| Hubei | 6554 | 376 | 6 | 32 | Xiangyang | 2 | 6 | 93 | 282 | Secondary | General | 9 | 9 |
| Hubei | 6554 | 376 | 6 | 32 | Xiangyang | 2 | 6 | 93 | 284 | Secondary | General | 12 | 12 |
| Hubei | 6554 | 376 | 6 | 32 | Xiangyang | 2 | 6 | 93 | 285 | Tertiary | General | 26 | 26 |
| Hubei | 6554 | 376 | 6 | 32 | Xiangyang | 2 | 6 | 93 | 286 | Tertiary | General | 24 | 24 |
| Hubei | 6554 | 376 | 6 | 32 | Xiaogan | 2 | 5 | 48 | 288 | Primary | Specialized | 14 | 14 |
| Hubei | 6554 | 376 | 6 | 32 | Xiaogan | 2 | 5 | 48 | 292 | Secondary | General | 11 | 11 |
| Shanxi | 4442 | 388 | 6 | 33 | Xinzhou | 1 | 6 | 66 | 561 | Primary | Specialized | 13 | 13 |
| Shanxi | 4442 | 388 | 6 | 33 | Xinzhou | 1 | 6 | 66 | 563 | Primary | Specialized | 12 | 12 |
| Shanxi | 4442 | 388 | 6 | 33 | Xinzhou | 1 | 6 | 66 | 566 | Secondary | General | 6 | 6 |
| Shanxi | 4442 | 388 | 6 | 33 | Xinzhou | 1 | 6 | 66 | 562 | Tertiary | General | 8 | 8 |
| Shanxi | 4442 | 388 | 6 | 33 | Xinzhou | 1 | 6 | 66 | 564 | Tertiary | General | 18 | 18 |
| Henan | 9336 | 416 | 6 | 31 | Xinxiang | 2 | 6 | 84 | 229 | Secondary | Specialized | 19 | 19 |
| Henan | 9336 | 416 | 6 | 31 | Xinxiang | 2 | 6 | 84 | 228 | Tertiary | General | 13 | 13 |
| Henan | 9336 | 416 | 6 | 31 | Xinxiang | 2 | 6 | 84 | 231 | Tertiary | General | 28 | 28 |
| Henan | 9336 | 416 | 6 | 31 | Xinxiang | 2 | 6 | 84 | 232 | Primary | General | 8 | 8 |
| Jiangxi | 3793 | 384 | 5 | 26 | Xinyu | 1 | 6 | 86 | 408 | Secondary | Specialized | 34 | 34 |
| Jiangxi | 3793 | 384 | 5 | 26 | Xinyu | 1 | 6 | 86 | 405 | Secondary | General | 11 | 11 |
| Jiangxi | 3793 | 384 | 5 | 26 | Xinyu | 1 | 6 | 86 | 409 | Tertiary | General | 20 | 20 |
| Jiangxi | 3793 | 384 | 5 | 26 | Xinyu | 1 | 6 | 86 | 410 | Tertiary | General | 9 | 9 |
| Jiangxi | 3793 | 384 | 5 | 26 | Xinyu | 1 | 6 | 86 | 406 | Primary | General | 6 | 6 |
| Jiangxi | 3793 | 384 | 5 | 26 | Xinyu | 1 | 6 | 86 | 407 | Primary | General | 6 | 6 |
| Hebei | 8525 | 687 | 6 | 33 | Xingtai | 2 | 5 | 97 | 197 | Secondary | General | 22 | 22 |
| Hebei | 8525 | 687 | 6 | 33 | Xingtai | 2 | 5 | 97 | 199 | Secondary | General | 29 | 29 |
| Hebei | 8525 | 687 | 6 | 33 | Xingtai | 2 | 5 | 97 | 201 | Tertiary | General | 38 | 38 |
| Jiangsu | 10033 | 740 | 6 | 36 | Suqian | 2 | 6 | 58 | 376 | Secondary | General | 10 | 10 |
| Jiangsu | 10033 | 740 | 6 | 36 | Suqian | 2 | 6 | 58 | 377 | Secondary | General | 7 | 7 |
| Jiangsu | 10033 | 740 | 6 | 36 | Suqian | 2 | 6 | 58 | 378 | Tertiary | General | 15 | 15 |
| Jiangsu | 10033 | 740 | 6 | 36 | Suqian | 2 | 6 | 58 | 380 | Tertiary | General | 20 | 20 |
| Jiangsu | 10033 | 740 | 6 | 36 | Suqian | 2 | 6 | 58 | 379 | Primary | General | 4 | 4 |
| Jiangsu | 10033 | 740 | 6 | 36 | Suqian | 2 | 6 | 58 | 381 | Primary | General | 2 | 2 |
| Sichuan | 8864 | 743 | 6 | 28 | Ya'an | 2 | 4 | 54 | 650 | Primary | Specialized | 17 | 17 |
| Sichuan | 8864 | 743 | 6 | 28 | Ya'an | 2 | 4 | 54 | 649 | Secondary | General | 12 | 12 |
| Sichuan | 8864 | 743 | 6 | 28 | Ya'an | 2 | 4 | 54 | 651 | Tertiary | General | 21 | 21 |
| Sichuan | 8864 | 743 | 6 | 28 | Ya'an | 2 | 4 | 54 | 652 | Primary | General | 4 | 4 |
| Jiangsu | 10033 | 740 | 6 | 36 | Yancheng | 2 | 6 | 155 | 382 | Secondary | General | 15 | 15 |
| Jiangsu | 10033 | 740 | 6 | 36 | Yancheng | 2 | 6 | 155 | 383 | Secondary | General | 16 | 16 |
| Jiangsu | 10033 | 740 | 6 | 36 | Yancheng | 2 | 6 | 155 | 385 | Tertiary | General | 65 | 65 |
| Jiangsu | 10033 | 740 | 6 | 36 | Yancheng | 2 | 6 | 155 | 384 | Primary | General | 29 | 29 |
| Guangdong | 11859 | 490 | 6 | 19 | Yangjiang | 2 | 2 | 18 | 102 | Primary | Specialized | 8 | 8 |
| Guangdong | 11859 | 490 | 6 | 19 | Yangjiang | 2 | 2 | 18 | 103 | Tertiary | General | 10 | 10 |
| Shanxi | 4442 | 388 | 6 | 33 | Yangquan | 1 | 6 | 43 | 567 | Secondary | General | 9 | 9 |
| Shanxi | 4442 | 388 | 6 | 33 | Yangquan | 1 | 6 | 43 | 569 | Tertiary | General | 15 | 15 |
| Shanxi | 4442 | 388 | 6 | 33 | Yangquan | 1 | 6 | 43 | 570 | Tertiary | General | 12 | 12 |
| Shanxi | 4442 | 388 | 6 | 33 | Yangquan | 1 | 6 | 43 | 572 | Primary | General | 4 | 4 |
| Heilongjiang | 3956 | 346 | 6 | 28 | Yichun | 1 | 4 | 9 | 258 | Secondary | General | 4 | 4 |
| Heilongjiang | 3956 | 346 | 6 | 28 | Yichun | 1 | 4 | 9 | 257 | Primary | General | 1 | 1 |
| Hunan | 7212 | 477 | 6 | 33 | Yiyang | 2 | 5 | 48 | 306 | Secondary | General | 9 | 9 |
| Hunan | 7212 | 477 | 6 | 33 | Yiyang | 2 | 5 | 48 | 307 | Tertiary | General | 11 | 11 |
| Hunan | 7212 | 477 | 6 | 33 | Yiyang | 2 | 5 | 48 | 309 | Tertiary | General | 13 | 13 |
| Ningxia | 885 | 221 | 5 | 21 | Yinchuan | 2 | 6 | 67 | 475 | Primary | Specialized | 12 | 12 |
| Ningxia | 885 | 221 | 5 | 21 | Yinchuan | 2 | 6 | 67 | 477 | Tertiary | General | 29 | 29 |
| Jiangxi | 3793 | 384 | 5 | 26 | Yingtan | 1 | 3 | 26 | 413 | Primary | Specialized | 8 | 8 |
| Jiangxi | 3793 | 384 | 5 | 26 | Yingtan | 1 | 3 | 26 | 412 | Tertiary | General | 14 | 14 |
| Jiangxi | 3793 | 384 | 5 | 26 | Yingtan | 1 | 3 | 26 | 411 | Primary | General | 4 | 4 |
| Liaoning | 5615 | 319 | 4 | 24 | Yingkou | 2 | 6 | 37 | 433 | Tertiary | General | 10 | 10 |
| Liaoning | 5615 | 319 | 4 | 24 | Yingkou | 2 | 6 | 37 | 435 | Primary | General | 2 | 2 |
| Liaoning | 5615 | 319 | 4 | 24 | Yingkou | 2 | 6 | 37 | 436 | Primary | General | 2 | 2 |
| Shaanxi | 4289 | 429 | 6 | 27 | Yulin | 2 | 4 | 50 | 596 | Tertiary | General | 27 | 27 |
| Qinghai | 710 | 246 | 6 | 25 | Yushu | 1 | 4 | 27 | 506 | Secondary | Specialized | 10 | 10 |
| Qinghai | 710 | 246 | 6 | 25 | Yushu | 1 | 4 | 27 | 507 | Secondary | General | 7 | 7 |
| Qinghai | 710 | 246 | 6 | 25 | Yushu | 1 | 4 | 27 | 508 | Primary | General | 6 | 6 |
| Qinghai | 710 | 246 | 6 | 25 | Yushu | 1 | 4 | 27 | 509 | Primary | General | 4 | 4 |
| Yunnan | 4256 | 263 | 6 | 22 | Yuxi | 2 | 3 | 28 | 722 | Tertiary | General | 10 | 10 |
| Shandong | 12695 | 512 | 6 | 30 | Zaozhuang | 2 | 5 | 78 | 530 | Tertiary | Specialized | 47 | 47 |
| Shandong | 12695 | 512 | 6 | 30 | Zaozhuang | 2 | 5 | 78 | 532 | Secondary | General | 8 | 8 |
| Hunan | 7212 | 477 | 6 | 33 | Zhangjiajie | 1 | 4 | 33 | 310 | Primary | Specialized | 8 | 8 |
| Hunan | 7212 | 477 | 6 | 33 | Zhangjiajie | 1 | 4 | 33 | 311 | Secondary | General | 15 | 15 |
| Hunan | 7212 | 477 | 6 | 33 | Zhangjiajie | 1 | 4 | 33 | 313 | Secondary | General | 4 | 4 |
| Hunan | 7212 | 477 | 6 | 33 | Zhangjiajie | 1 | 4 | 33 | 312 | Primary | General | 6 | 6 |
| Jilin | 3484 | 232 | 5 | 26 | Changchun | 3 | 6 | 52 | 347 | Primary | Specialized | 7 | 7 |
| Jilin | 3484 | 232 | 5 | 26 | Changchun | 3 | 6 | 52 | 349 | Secondary | General | 5 | 5 |
| Jilin | 3484 | 232 | 5 | 26 | Changchun | 3 | 6 | 52 | 351 | Secondary | General | 8 | 8 |
| Jilin | 3484 | 232 | 5 | 26 | Changchun | 3 | 6 | 52 | 346 | Tertiary | General | 10 | 10 |
| Jilin | 3484 | 232 | 5 | 26 | Changchun | 3 | 6 | 52 | 348 | Tertiary | General | 16 | 16 |
| Hunan | 7212 | 477 | 6 | 33 | Changsha | 3 | 6 | 85 | 316 | Secondary | General | 17 | 17 |
| Hunan | 7212 | 477 | 6 | 33 | Changsha | 3 | 6 | 85 | 318 | Secondary | General | 10 | 10 |
| Hunan | 7212 | 477 | 6 | 33 | Changsha | 3 | 6 | 85 | 314 | Tertiary | General | 15 | 15 |
| Hunan | 7212 | 477 | 6 | 33 | Changsha | 3 | 6 | 85 | 315 | Tertiary | General | 33 | 33 |
| Yunnan | 4256 | 263 | 6 | 22 | Zhaotong | 2 | 6 | 96 | 727 | Tertiary | General | 49 | 49 |
| Yunnan | 4256 | 263 | 6 | 22 | Zhaotong | 2 | 6 | 96 | 728 | Tertiary | General | 21 | 21 |
| Yunnan | 4256 | 263 | 6 | 22 | Zhaotong | 2 | 6 | 96 | 726 | Primary | General | 14 | 14 |
| Guangdong | 11859 | 490 | 6 | 19 | Zhaoqing | 2 | 1 | 39 | 104 | Tertiary | General | 39 | 39 |
| Ningxia | 885 | 221 | 5 | 21 | Zhongwei | 1 | 4 | 37 | 482 | Secondary | General | 20 | 20 |
| Ningxia | 885 | 221 | 5 | 21 | Zhongwei | 1 | 4 | 37 | 483 | Secondary | General | 9 | 9 |
| Chongqing | 3199 | 177 | 1 | 15 | Chongqing | 3 | 15 | 177 | 765 | Secondary | Specialized | 7 | 7 |
| Chongqing | 3199 | 177 | 1 | 15 | Chongqing | 3 | 15 | 177 | 768 | Secondary | Specialized | 26 | 26 |
| Chongqing | 3199 | 177 | 1 | 15 | Chongqing | 3 | 15 | 177 | 775 | Secondary | Specialized | 8 | 8 |
| Chongqing | 3199 | 177 | 1 | 15 | Chongqing | 3 | 15 | 177 | 776 | Secondary | Specialized | 15 | 15 |
| Chongqing | 3199 | 177 | 1 | 15 | Chongqing | 3 | 15 | 177 | 772 | Secondary | General | 8 | 8 |
| Chongqing | 3199 | 177 | 1 | 15 | Chongqing | 3 | 15 | 177 | 767 | Tertiary | General | 12 | 12 |
| Chongqing | 3199 | 177 | 1 | 15 | Chongqing | 3 | 15 | 177 | 769 | Tertiary | General | 20 | 20 |
| Chongqing | 3199 | 177 | 1 | 15 | Chongqing | 3 | 15 | 177 | 779 | Tertiary | General | 32 | 32 |
| Chongqing | 3199 | 177 | 1 | 15 | Chongqing | 3 | 15 | 177 | 773 | Primary | General | 2 | 2 |
| Chongqing | 3199 | 177 | 1 | 15 | Chongqing | 3 | 15 | 177 | 774 | Primary | General | 2 | 2 |
| Zhejiang | 8597 | 745 | 6 | 36 | Zhoushan | 1 | 6 | 126 | 762 | Tertiary | Specialized | 54 | 54 |
| Zhejiang | 8597 | 745 | 6 | 36 | Zhoushan | 1 | 6 | 126 | 759 | Primary | Specialized | 4 | 4 |
| Zhejiang | 8597 | 745 | 6 | 36 | Zhoushan | 1 | 6 | 126 | 760 | Secondary | General | 9 | 9 |
| Zhejiang | 8597 | 745 | 6 | 36 | Zhoushan | 1 | 6 | 126 | 763 | Secondary | General | 35 | 35 |
| Zhejiang | 8597 | 745 | 6 | 36 | Zhoushan | 1 | 6 | 126 | 761 | Tertiary | General | 23 | 23 |
| Zhejiang | 8597 | 745 | 6 | 36 | Zhoushan | 1 | 6 | 126 | 764 | Primary | General | 1 | 1 |
| Guangdong | 11859 | 490 | 6 | 19 | Zhuhai | 1 | 3 | 117 | 105 | Tertiary | Specialized | 80 | 80 |
| Guangdong | 11859 | 490 | 6 | 19 | Zhuhai | 1 | 3 | 117 | 106 | Tertiary | General | 32 | 32 |
| Hunan | 7212 | 477 | 6 | 33 | Zhuzhou | 2 | 6 | 141 | 321 | Secondary | Specialized | 34 | 34 |
| Hunan | 7212 | 477 | 6 | 33 | Zhuzhou | 2 | 6 | 141 | 320 | Primary | Specialized | 24 | 24 |
| Hunan | 7212 | 477 | 6 | 33 | Zhuzhou | 2 | 6 | 141 | 325 | Secondary | General | 22 | 22 |
| Hunan | 7212 | 477 | 6 | 33 | Zhuzhou | 2 | 6 | 141 | 322 | Tertiary | General | 22 | 22 |
| Hunan | 7212 | 477 | 6 | 33 | Zhuzhou | 2 | 6 | 141 | 324 | Tertiary | General | 33 | 33 |
| Shandong | 12695 | 512 | 6 | 30 | Zibo | 2 | 5 | 85 | 538 | Secondary | General | 19 | 19 |
| Shandong | 12695 | 512 | 6 | 30 | Zibo | 2 | 5 | 85 | 537 | Tertiary | General | 45 | 45 |
| Guizhou | 3377 | 613 | 6 | 32 | Zunyi | 1 | 5 | 59 | 154 | Secondary | General | 12 | 12 |
| Guizhou | 3377 | 613 | 6 | 32 | Zunyi | 1 | 5 | 59 | 156 | Secondary | General | 15 | 15 |
| Guizhou | 3377 | 613 | 6 | 32 | Zunyi | 1 | 5 | 59 | 158 | Tertiary | General | 22 | 22 |
| Guizhou | 3377 | 613 | 6 | 32 | Zunyi | 1 | 5 | 59 | 155 | Primary | General | 4 | 4 |
| Guizhou | 3377 | 613 | 6 | 32 | Zunyi | 1 | 5 | 59 | 157 | Primary | General | 6 | 6 |

# Supplement 6: The results of the weighting analysis

| Province | Cities | Regional classification | Stratified variable | Physician numbers in each province | Number of hospitals in the city | Hospital codes | FPC variable 1 | FPC variable 2 | Sampling probability 1 | Sampling probability 2 | Sampling weight | Individual non-response rate | Institutional non-response rate | Total non-response rate | Adjustment factor of non-response rate | Proportion adjustment factor of each layer | Total weight |
| --- | --- | --- | --- | --- | --- | --- | --- | --- | --- | --- | --- | --- | --- | --- | --- | --- | --- |
| Chongqing | Chongqing | 3 | 190 | 583 | 800 | 773 | 1 | 39 | 1.000 | 0.308 | 3.250 | 1.000 | 0.300 | 0.300 | 3.333 | 1.369 | 14.836 |
| Chongqing | Chongqing | 3 | 190 | 583 | 800 | 774 | 1 | 39 | 1.000 | 0.308 | 3.250 | 1.000 | 0.300 | 0.300 | 3.333 | 1.369 | 14.836 |
| Chongqing | Chongqing | 3 | 190 | 583 | 800 | 766 | 1 | 39 | 1.000 | 0.308 | 3.250 | 0.667 | 0.300 | 0.200 | 5.000 | 1.369 | 22.254 |
| Chongqing | Chongqing | 3 | 190 | 583 | 800 | N/A | 1 | 39 | 1.000 | 0.308 |  |  |  |  |  |  |  |
| Chongqing | Chongqing | 3 | 190 | 583 | 800 | N/A | 1 | 39 | 1.000 | 0.308 |  |  |  |  |  |  |  |
| Chongqing | Chongqing | 3 | 190 | 583 | 800 | N/A | 1 | 39 | 1.000 | 0.308 |  |  |  |  |  |  |  |
| Chongqing | Chongqing | 3 | 190 | 583 | 800 | N/A | 1 | 39 | 1.000 | 0.308 |  |  |  |  |  |  |  |
| Chongqing | Chongqing | 3 | 190 | 583 | 800 | N/A | 1 | 39 | 1.000 | 0.308 |  |  |  |  |  |  |  |
| Chongqing | Chongqing | 3 | 190 | 583 | 800 | N/A | 1 | 39 | 1.000 | 0.308 |  |  |  |  |  |  |  |
| Chongqing | Chongqing | 3 | 190 | 583 | 800 | N/A | 1 | 39 | 1.000 | 0.308 |  |  |  |  |  |  |  |
| Chongqing | Chongqing | 3 | 190 | 583 | 800 | N/A | 1 | 39 | 1.000 | 0.308 |  |  |  |  |  |  |  |
| Chongqing | Chongqing | 3 | 190 | 583 | 800 | N/A | 1 | 39 | 1.000 | 0.308 |  |  |  |  |  |  |  |
| Chongqing | Chongqing | 3 | 189 | 583 | 800 | 779 | 1 | 55 | 1.000 | 0.200 | 5.000 | 1.000 | 0.600 | 0.600 | 1.667 | 1.369 | 11.412 |
| Chongqing | Chongqing | 3 | 189 | 583 | 800 | 769 | 1 | 55 | 1.000 | 0.200 | 5.000 | 1.000 | 0.600 | 0.600 | 1.667 | 1.369 | 11.412 |
| Chongqing | Chongqing | 3 | 189 | 583 | 800 | 767 | 1 | 55 | 1.000 | 0.200 | 5.000 | 1.000 | 0.600 | 0.600 | 1.667 | 1.369 | 11.412 |
| Chongqing | Chongqing | 3 | 189 | 583 | 800 | 778 | 1 | 55 | 1.000 | 0.200 | 5.000 | 0.909 | 0.600 | 0.545 | 1.833 | 1.369 | 12.554 |
| Chongqing | Chongqing | 3 | 189 | 583 | 800 | 771 | 1 | 55 | 1.000 | 0.200 | 5.000 | 0.526 | 0.600 | 0.316 | 3.167 | 1.369 | 21.684 |
| Chongqing | Chongqing | 3 | 189 | 583 | 800 | 770 | 1 | 55 | 1.000 | 0.200 | 5.000 | 0.429 | 0.600 | 0.257 | 3.889 | 1.369 | 26.629 |
| Chongqing | Chongqing | 3 | 189 | 583 | 800 | N/A | 1 | 55 | 1.000 | 0.200 |  |  |  |  |  |  |  |
| Chongqing | Chongqing | 3 | 189 | 583 | 800 | N/A | 1 | 55 | 1.000 | 0.200 |  |  |  |  |  |  |  |
| Chongqing | Chongqing | 3 | 189 | 583 | 800 | N/A | 1 | 55 | 1.000 | 0.200 |  |  |  |  |  |  |  |
| Chongqing | Chongqing | 3 | 189 | 583 | 800 | N/A | 1 | 55 | 1.000 | 0.200 |  |  |  |  |  |  |  |
| Chongqing | Chongqing | 3 | 189 | 583 | 800 | N/A | 1 | 55 | 1.000 | 0.200 |  |  |  |  |  |  |  |
| Chongqing | Chongqing | 3 | 188 | 583 | 800 | 775 | 1 | 102 | 1.000 | 0.127 | 7.846 | 1.000 | 0.545 | 0.545 | 1.833 | 1.369 | 19.700 |
| Chongqing | Chongqing | 3 | 188 | 583 | 800 | 776 | 1 | 102 | 1.000 | 0.127 | 7.846 | 1.000 | 0.545 | 0.545 | 1.833 | 1.369 | 19.700 |
| Chongqing | Chongqing | 3 | 188 | 583 | 800 | 772 | 1 | 102 | 1.000 | 0.127 | 7.846 | 1.000 | 0.545 | 0.545 | 1.833 | 1.369 | 19.700 |
| Chongqing | Chongqing | 3 | 188 | 583 | 800 | 768 | 1 | 102 | 1.000 | 0.127 | 7.846 | 1.000 | 0.545 | 0.545 | 1.833 | 1.369 | 19.700 |
| Chongqing | Chongqing | 3 | 188 | 583 | 800 | 765 | 1 | 102 | 1.000 | 0.127 | 7.846 | 1.000 | 0.545 | 0.545 | 1.833 | 1.369 | 19.700 |
| Chongqing | Chongqing | 3 | 188 | 583 | 800 | 777 | 1 | 102 | 1.000 | 0.127 | 7.846 | 0.500 | 0.545 | 0.273 | 3.667 | 1.369 | 39.399 |
| Chongqing | Chongqing | 3 | 188 | 583 | 800 | N/A | 1 | 102 | 1.000 | 0.127 |  |  |  |  |  |  |  |
| Chongqing | Chongqing | 3 | 188 | 583 | 800 | N/A | 1 | 102 | 1.000 | 0.127 |  |  |  |  |  |  |  |
| Chongqing | Chongqing | 3 | 188 | 583 | 800 | N/A | 1 | 102 | 1.000 | 0.127 |  |  |  |  |  |  |  |
| Chongqing | Chongqing | 3 | 188 | 583 | 800 | N/A | 1 | 102 | 1.000 | 0.127 |  |  |  |  |  |  |  |
| Chongqing | Chongqing | 3 | 188 | 583 | 800 | N/A | 1 | 102 | 1.000 | 0.127 |  |  |  |  |  |  |  |
| Chongqing | Chongqing | 3 | 188 | 583 | 800 | N/A | 1 | 102 | 1.000 | 0.127 |  |  |  |  |  |  |  |
| Chongqing | Chongqing | 3 | 188 | 583 | 800 | N/A | 1 | 102 | 1.000 | 0.127 |  |  |  |  |  |  |  |
| Zhejiang | Wenzhou | 3 | 187 | 1275 | 145 | 755 | 3 | 14 | 0.333 | 0.143 | 21.000 | 1.000 | 1.000 | 1.000 | 1.000 | 0.874 | 18.364 |
| Zhejiang | Wenzhou | 3 | 187 | 1275 | 145 | 754 | 3 | 14 | 0.333 | 0.143 | 21.000 | 0.833 | 1.000 | 0.833 | 1.200 | 0.874 | 22.037 |
| Zhejiang | Wenzhou | 3 | 186 | 1275 | 145 | 757 | 3 | 9 | 0.333 | 0.222 | 13.500 | 1.000 | 1.000 | 1.000 | 1.000 | 0.874 | 11.806 |
| Zhejiang | Wenzhou | 3 | 186 | 1275 | 145 | 756 | 3 | 9 | 0.333 | 0.222 | 13.500 | 1.000 | 1.000 | 1.000 | 1.000 | 0.874 | 11.806 |
| Zhejiang | Wenzhou | 3 | 185 | 1275 | 145 | 753 | 3 | 11 | 0.333 | 0.182 | 16.500 | 1.000 | 1.000 | 1.000 | 1.000 | 0.874 | 14.429 |
| Zhejiang | Wenzhou | 3 | 185 | 1275 | 145 | 758 | 3 | 11 | 0.333 | 0.182 | 16.500 | 0.889 | 1.000 | 0.889 | 1.125 | 0.874 | 16.233 |
| Zhejiang | Lishui | 2 | 184 | 1275 | 56 | 746 | 6 | 6 | 0.500 | 0.333 | 6.000 | 0.917 | 1.000 | 0.917 | 1.091 | 0.874 | 5.724 |
| Zhejiang | Quzhou | 2 | 184 | 1275 | 84 | 749 | 6 | 8 | 0.500 | 0.250 | 8.000 | 0.833 | 1.000 | 0.833 | 1.200 | 0.874 | 8.395 |
| Zhejiang | Lishui | 2 | 184 | 1275 | 56 | 745 | 6 | 6 | 0.500 | 0.333 | 6.000 | 0.556 | 1.000 | 0.556 | 1.800 | 0.874 | 9.445 |
| Zhejiang | Quzhou | 2 | 184 | 1275 | 84 | 750 | 6 | 8 | 0.500 | 0.250 | 8.000 | 0.462 | 1.000 | 0.462 | 2.167 | 0.874 | 15.158 |
| Zhejiang | Jinhua | 2 | 184 | 1275 | 137 | 737 | 6 | 29 | 0.500 | 0.069 | 29.000 | 1.000 | 1.000 | 1.000 | 1.000 | 0.874 | 25.360 |
| Zhejiang | Jinhua | 2 | 184 | 1275 | 137 | 738 | 6 | 29 | 0.500 | 0.069 | 29.000 | 0.818 | 1.000 | 0.818 | 1.222 | 0.874 | 30.996 |
| Zhejiang | Lishui | 2 | 183 | 1275 | 56 | 742 | 6 | 3 | 0.500 | 0.667 | 3.000 | 1.000 | 1.000 | 1.000 | 1.000 | 0.874 | 2.623 |
| Zhejiang | Lishui | 2 | 183 | 1275 | 56 | 741 | 6 | 3 | 0.500 | 0.667 | 3.000 | 1.000 | 1.000 | 1.000 | 1.000 | 0.874 | 2.623 |
| Zhejiang | Quzhou | 2 | 183 | 1275 | 84 | 751 | 6 | 4 | 0.500 | 0.500 | 4.000 | 1.000 | 1.000 | 1.000 | 1.000 | 0.874 | 3.498 |
| Zhejiang | Quzhou | 2 | 183 | 1275 | 84 | 747 | 6 | 4 | 0.500 | 0.500 | 4.000 | 1.000 | 1.000 | 1.000 | 1.000 | 0.874 | 3.498 |
| Zhejiang | Jinhua | 2 | 183 | 1275 | 137 | 736 | 6 | 8 | 0.500 | 0.250 | 8.000 | 1.000 | 1.000 | 1.000 | 1.000 | 0.874 | 6.996 |
| Zhejiang | Jinhua | 2 | 183 | 1275 | 137 | 735 | 6 | 8 | 0.500 | 0.250 | 8.000 | 1.000 | 1.000 | 1.000 | 1.000 | 0.874 | 6.996 |
| Zhejiang | Lishui | 2 | 182 | 1275 | 56 | 744 | 6 | 6 | 0.500 | 0.333 | 6.000 | 1.000 | 1.000 | 1.000 | 1.000 | 0.874 | 5.247 |
| Zhejiang | Lishui | 2 | 182 | 1275 | 56 | 743 | 6 | 6 | 0.500 | 0.333 | 6.000 | 1.000 | 1.000 | 1.000 | 1.000 | 0.874 | 5.247 |
| Zhejiang | Jinhua | 2 | 182 | 1275 | 137 | 740 | 6 | 7 | 0.500 | 0.286 | 7.000 | 1.000 | 1.000 | 1.000 | 1.000 | 0.874 | 6.121 |
| Zhejiang | Jinhua | 2 | 182 | 1275 | 137 | 739 | 6 | 7 | 0.500 | 0.286 | 7.000 | 1.000 | 1.000 | 1.000 | 1.000 | 0.874 | 6.121 |
| Zhejiang | Quzhou | 2 | 182 | 1275 | 84 | 748 | 6 | 7 | 0.500 | 0.286 | 7.000 | 1.000 | 1.000 | 1.000 | 1.000 | 0.874 | 6.121 |
| Zhejiang | Quzhou | 2 | 182 | 1275 | 84 | 752 | 6 | 7 | 0.500 | 0.286 | 7.000 | 1.000 | 1.000 | 1.000 | 1.000 | 0.874 | 6.121 |
| Zhejiang | Zhoushan | 1 | 181 | 1275 | 33 | 764 | 2 | 9 | 1.000 | 0.222 | 4.500 | 1.000 | 1.000 | 1.000 | 1.000 | 0.874 | 3.935 |
| Zhejiang | Zhoushan | 1 | 181 | 1275 | 33 | 759 | 2 | 9 | 1.000 | 0.222 | 4.500 | 1.000 | 1.000 | 1.000 | 1.000 | 0.874 | 3.935 |
| Zhejiang | Jiaxing | 1 | 181 | 1275 | 0 | 732 | 2 | 25 | 1.000 | 0.080 | 12.500 | 1.000 | 1.000 | 1.000 | 1.000 | 0.874 | 10.931 |
| Zhejiang | Jiaxing | 1 | 181 | 1275 | 0 | 730 | 2 | 25 | 1.000 | 0.080 | 12.500 | 1.000 | 1.000 | 1.000 | 1.000 | 0.874 | 10.931 |
| Zhejiang | Zhoushan | 1 | 180 | 1275 | 33 | 762 | 2 | 3 | 1.000 | 0.667 | 1.500 | 1.000 | 1.000 | 1.000 | 1.000 | 0.874 | 1.312 |
| Zhejiang | Zhoushan | 1 | 180 | 1275 | 33 | 761 | 2 | 3 | 1.000 | 0.667 | 1.500 | 1.000 | 1.000 | 1.000 | 1.000 | 0.874 | 1.312 |
| Zhejiang | Jiaxing | 1 | 180 | 1275 | 0 | 733 | 2 | 8 | 1.000 | 0.250 | 4.000 | 1.000 | 1.000 | 1.000 | 1.000 | 0.874 | 3.498 |
| Zhejiang | Jiaxing | 1 | 180 | 1275 | 0 | 731 | 2 | 8 | 1.000 | 0.250 | 4.000 | 1.000 | 1.000 | 1.000 | 1.000 | 0.874 | 3.498 |
| Zhejiang | Zhoushan | 1 | 179 | 1275 | 33 | 763 | 2 | 8 | 1.000 | 0.250 | 4.000 | 1.000 | 1.000 | 1.000 | 1.000 | 0.874 | 3.498 |
| Zhejiang | Zhoushan | 1 | 179 | 1275 | 33 | 760 | 2 | 8 | 1.000 | 0.250 | 4.000 | 1.000 | 1.000 | 1.000 | 1.000 | 0.874 | 3.498 |
| Zhejiang | Jiaxing | 1 | 179 | 1275 | 0 | 734 | 2 | 16 | 1.000 | 0.125 | 8.000 | 1.000 | 1.000 | 1.000 | 1.000 | 0.874 | 6.996 |
| Zhejiang | Jiaxing | 1 | 179 | 1275 | 0 | 729 | 2 | 16 | 1.000 | 0.125 | 8.000 | 1.000 | 1.000 | 1.000 | 1.000 | 0.874 | 6.996 |
| Yunnan | Kunming | 3 | 178 | 195 | 322 | 709 | 1 | 50 | 1.000 | 0.040 | 25.000 | 1.000 | 1.000 | 1.000 | 1.000 | 1.226 | 30.656 |
| Yunnan | Kunming | 3 | 178 | 195 | 322 | 707 | 1 | 50 | 1.000 | 0.040 | 25.000 | 1.000 | 1.000 | 1.000 | 1.000 | 1.226 | 30.656 |
| Yunnan | Kunming | 3 | 177 | 195 | 322 | 710 | 1 | 20 | 1.000 | 0.100 | 10.000 | 1.000 | 1.000 | 1.000 | 1.000 | 1.226 | 12.262 |
| Yunnan | Kunming | 3 | 177 | 195 | 322 | 708 | 1 | 20 | 1.000 | 0.100 | 10.000 | 1.000 | 1.000 | 1.000 | 1.000 | 1.226 | 12.262 |
| Yunnan | Kunming | 3 | 176 | 195 | 322 | N/A | 1 | 31 | 1.000 | 0.065 |  |  |  |  |  |  |  |
| Yunnan | Kunming | 3 | 176 | 195 | 322 | N/A | 1 | 31 | 1.000 | 0.065 |  |  |  |  |  |  |  |
| Yunnan | Zhaotong | 2 | 175 | 195 | 167 | 726 | 4 | 16 | 1.000 | 0.125 | 8.000 | 1.000 | 1.000 | 1.000 | 1.000 | 1.226 | 9.810 |
| Yunnan | Qujing | 2 | 175 | 195 | 114 | 719 | 4 | 16 | 1.000 | 0.125 | 8.000 | 0.545 | 1.000 | 0.545 | 1.833 | 1.226 | 17.985 |
| Yunnan | Zhaotong | 2 | 175 | 195 | 167 | 725 | 4 | 16 | 1.000 | 0.125 | 8.000 | 0.250 | 1.000 | 0.250 | 4.000 | 1.226 | 39.239 |
| Yunnan | Qujing | 2 | 175 | 195 | 114 | 717 | 4 | 16 | 1.000 | 0.125 | 8.000 | 0.091 | 1.000 | 0.091 | 11.000 | 1.226 | 107.908 |
| Yunnan | Pu'er | 2 | 175 | 195 | 47 | N/A | 4 | 4 | 1.000 | 0.500 |  |  |  |  |  |  |  |
| Yunnan | Pu'er | 2 | 175 | 195 | 47 | N/A | 4 | 4 | 1.000 | 0.500 |  |  |  |  |  |  |  |
| Yunnan | Yuxi | 2 | 175 | 195 | 71 | N/A | 4 | 11 | 1.000 | 0.182 |  |  |  |  |  |  |  |
| Yunnan | Yuxi | 2 | 175 | 195 | 71 | N/A | 4 | 11 | 1.000 | 0.182 |  |  |  |  |  |  |  |
| Yunnan | Yuxi | 2 | 174 | 195 | 71 | 722 | 4 | 4 | 1.000 | 0.500 | 2.000 | 1.000 | 1.000 | 1.000 | 1.000 | 1.226 | 2.452 |
| Yunnan | Yuxi | 2 | 174 | 195 | 71 | 721 | 4 | 4 | 1.000 | 0.500 | 2.000 | 0.929 | 1.000 | 0.929 | 1.077 | 1.226 | 2.641 |
| Yunnan | Zhaotong | 2 | 174 | 195 | 167 | 728 | 4 | 5 | 1.000 | 0.400 | 2.500 | 1.000 | 1.000 | 1.000 | 1.000 | 1.226 | 3.066 |
| Yunnan | Zhaotong | 2 | 174 | 195 | 167 | 727 | 4 | 5 | 1.000 | 0.400 | 2.500 | 1.000 | 1.000 | 1.000 | 1.000 | 1.226 | 3.066 |
| Yunnan | Pu'er | 2 | 174 | 195 | 47 | N/A | 4 | 1 | 1.000 | 1.000 |  |  |  |  |  |  |  |
| Yunnan | Qujing | 2 | 174 | 195 | 114 | N/A | 4 | 4 | 1.000 | 0.500 |  |  |  |  |  |  |  |
| Yunnan | Qujing | 2 | 174 | 195 | 114 | N/A | 4 | 4 | 1.000 | 0.500 |  |  |  |  |  |  |  |
| Yunnan | Pu'er | 2 | 173 | 195 | 47 | 715 | 4 | 6 | 1.000 | 0.500 | 2.000 | 1.000 | 0.667 | 0.667 | 1.500 | 1.226 | 3.679 |
| Yunnan | Zhaotong | 2 | 173 | 195 | 167 | 724 | 4 | 8 | 1.000 | 0.250 | 4.000 | 0.636 | 1.000 | 0.636 | 1.571 | 1.226 | 7.708 |
| Yunnan | Pu'er | 2 | 173 | 195 | 47 | 716 | 4 | 6 | 1.000 | 0.500 | 2.000 | 0.357 | 0.667 | 0.238 | 4.200 | 1.226 | 10.300 |
| Yunnan | Zhaotong | 2 | 173 | 195 | 167 | 723 | 4 | 8 | 1.000 | 0.250 | 4.000 | 0.273 | 1.000 | 0.273 | 3.667 | 1.226 | 17.985 |
| Yunnan | Qujing | 2 | 173 | 195 | 114 | 718 | 4 | 17 | 1.000 | 0.118 | 8.500 | 1.000 | 0.500 | 0.500 | 2.000 | 1.226 | 20.846 |
| Yunnan | Yuxi | 2 | 173 | 195 | 71 | 720 | 4 | 10 | 1.000 | 0.200 | 5.000 | 0.333 | 0.500 | 0.167 | 6.000 | 1.226 | 36.787 |
| Yunnan | Pu'er | 2 | 173 | 195 | 47 | N/A | 4 | 6 | 1.000 | 0.500 |  |  |  |  |  |  |  |
| Yunnan | Qujing | 2 | 173 | 195 | 114 | N/A | 4 | 17 | 1.000 | 0.118 |  |  |  |  |  |  |  |
| Yunnan | Yuxi | 2 | 173 | 195 | 71 | N/A | 4 | 10 | 1.000 | 0.200 |  |  |  |  |  |  |  |
| Yunnan | Lincang | 1 | 172 | 195 | 52 | 712 | 3 | 6 | 0.333 | 0.333 | 9.000 | 0.727 | 1.000 | 0.727 | 1.375 | 1.226 | 15.175 |
| Yunnan | Lincang | 1 | 172 | 195 | 52 | 711 | 3 | 6 | 0.333 | 0.333 | 9.000 | 0.667 | 1.000 | 0.667 | 1.500 | 1.226 | 16.554 |
| Yunnan | Lincang | 1 | 171 | 195 | 52 | 714 | 3 | 1 | 0.333 | 1.000 | 3.000 | 1.000 | 1.000 | 1.000 | 1.000 | 1.226 | 3.679 |
| Yunnan | Lincang | 1 | 170 | 195 | 52 | 713 | 3 | 10 | 0.333 | 0.200 | 15.000 | 0.286 | 0.500 | 0.143 | 7.000 | 1.226 | 128.754 |
| Yunnan | Lincang | 1 | 170 | 195 | 52 | N/A | 3 | 10 | 0.333 | 0.200 |  |  |  |  |  |  |  |
| Xinjiang | Shihezi | 2 | 169 | 902 | 0 | 695 | 1 | 7 | 1.000 | 0.286 | 3.500 | 1.000 | 1.000 | 1.000 | 1.000 | 0.881 | 3.085 |
| Xinjiang | Shihezi | 2 | 169 | 902 | 0 | 694 | 1 | 7 | 1.000 | 0.286 | 3.500 | 1.000 | 1.000 | 1.000 | 1.000 | 0.881 | 3.085 |
| Xinjiang | Urumqi | 2 | 169 | 902 | 130 | 705 | 1 | 34 | 1.000 | 0.059 | 17.000 | 1.000 | 0.500 | 0.500 | 2.000 | 0.881 | 29.965 |
| Xinjiang | Urumqi | 2 | 169 | 902 | 130 | N/A | 1 | 34 | 1.000 | 0.059 |  |  |  |  |  |  |  |
| Xinjiang | Shihezi | 2 | 168 | 902 | 0 | 696 | 1 | 1 | 1.000 | 1.000 | 1.000 | 1.000 | 1.000 | 1.000 | 1.000 | 0.881 | 0.881 |
| Xinjiang | Urumqi | 2 | 168 | 902 | 130 | 706 | 1 | 8 | 1.000 | 0.250 | 4.000 | 0.800 | 1.000 | 0.800 | 1.250 | 0.881 | 4.407 |
| Xinjiang | Urumqi | 2 | 168 | 902 | 130 | 704 | 1 | 8 | 1.000 | 0.250 | 4.000 | 0.500 | 1.000 | 0.500 | 2.000 | 0.881 | 7.051 |
| Xinjiang | Shihezi | 2 | 167 | 902 | 0 | 697 | 1 | 5 | 1.000 | 0.400 | 2.500 | 1.000 | 1.000 | 1.000 | 1.000 | 0.881 | 2.203 |
| Xinjiang | Shihezi | 2 | 167 | 902 | 0 | 698 | 1 | 5 | 1.000 | 0.400 | 2.500 | 0.875 | 1.000 | 0.875 | 1.143 | 0.881 | 2.518 |
| Xinjiang | Urumqi | 2 | 167 | 902 | 130 | 703 | 1 | 23 | 1.000 | 0.087 | 11.500 | 0.667 | 1.000 | 0.667 | 1.500 | 0.881 | 15.203 |
| Xinjiang | Urumqi | 2 | 167 | 902 | 130 | N/A | 1 | 23 | 1.000 | 0.087 |  |  |  |  |  |  |  |
| Xinjiang | Altay | 1 | 166 | 902 | 0 | 684 | 3 | 3 | 1.000 | 0.667 | 1.500 | 1.000 | 1.000 | 1.000 | 1.000 | 0.881 | 1.322 |
| Xinjiang | Hami | 1 | 166 | 902 | 35 | 688 | 3 | 4 | 1.000 | 0.500 | 2.000 | 1.000 | 1.000 | 1.000 | 1.000 | 0.881 | 1.763 |
| Xinjiang | Hami | 1 | 166 | 902 | 35 | 687 | 3 | 4 | 1.000 | 0.500 | 2.000 | 1.000 | 1.000 | 1.000 | 1.000 | 0.881 | 1.763 |
| Xinjiang | Turpan | 1 | 166 | 902 | 19 | 699 | 3 | 5 | 1.000 | 0.400 | 2.500 | 0.600 | 1.000 | 0.600 | 1.667 | 0.881 | 3.672 |
| Xinjiang | Altay | 1 | 166 | 902 | 0 | 686 | 3 | 3 | 1.000 | 0.667 | 1.500 | 0.167 | 1.000 | 0.167 | 6.000 | 0.881 | 7.932 |
| Xinjiang | Turpan | 1 | 166 | 902 | 19 | 701 | 3 | 5 | 1.000 | 0.400 | 2.500 | 0.100 | 1.000 | 0.100 | 10.000 | 0.881 | 22.033 |
| Xinjiang | Karamay | 1 | 166 | 902 | 13 | N/A | 3 | 5 | 1.000 | 0.400 |  |  |  |  |  |  |  |
| Xinjiang | Karamay | 1 | 166 | 902 | 13 | N/A | 3 | 5 | 1.000 | 0.400 |  |  |  |  |  |  |  |
| Xinjiang | Karamay | 1 | 165 | 902 | 13 | 691 | 3 | 1 | 1.000 | 1.000 | 1.000 | 1.000 | 1.000 | 1.000 | 1.000 | 0.881 | 0.881 |
| Xinjiang | Altay | 1 | 164 | 902 | 0 | 683 | 3 | 4 | 1.000 | 0.500 | 2.000 | 1.000 | 1.000 | 1.000 | 1.000 | 0.881 | 1.763 |
| Xinjiang | Hami | 1 | 164 | 902 | 35 | 690 | 3 | 5 | 1.000 | 0.400 | 2.500 | 1.000 | 1.000 | 1.000 | 1.000 | 0.881 | 2.203 |
| Xinjiang | Hami | 1 | 164 | 902 | 35 | 689 | 3 | 5 | 1.000 | 0.400 | 2.500 | 1.000 | 1.000 | 1.000 | 1.000 | 0.881 | 2.203 |
| Xinjiang | Karamay | 1 | 164 | 902 | 13 | 693 | 3 | 6 | 1.000 | 0.333 | 3.000 | 1.000 | 1.000 | 1.000 | 1.000 | 0.881 | 2.644 |
| Xinjiang | Karamay | 1 | 164 | 902 | 13 | 692 | 3 | 6 | 1.000 | 0.333 | 3.000 | 1.000 | 1.000 | 1.000 | 1.000 | 0.881 | 2.644 |
| Xinjiang | Turpan | 1 | 164 | 902 | 19 | 700 | 3 | 6 | 1.000 | 0.333 | 3.000 | 1.000 | 1.000 | 1.000 | 1.000 | 0.881 | 2.644 |
| Xinjiang | Altay | 1 | 164 | 902 | 0 | 685 | 3 | 4 | 1.000 | 0.500 | 2.000 | 0.250 | 1.000 | 0.250 | 4.000 | 0.881 | 7.051 |
| Xinjiang | Turpan | 1 | 164 | 902 | 19 | 702 | 3 | 6 | 1.000 | 0.333 | 3.000 | 0.091 | 1.000 | 0.091 | 11.000 | 0.881 | 29.084 |
| Tibet | Qamdo | 1 | 163 | 1 | 29 | 667 | 6 | 4 | 1.000 | 0.500 | 2.000 | 1.000 | 1.000 | 1.000 | 1.000 | 0.138 | 0.276 |
| Tibet | Qamdo | 1 | 163 | 1 | 29 | 666 | 6 | 4 | 1.000 | 0.500 | 2.000 | 0.750 | 1.000 | 0.750 | 1.333 | 0.138 | 0.368 |
| Tibet | Linzhi | 1 | 163 | 1 | 17 | 676 | 6 | 6 | 1.000 | 0.333 | 3.000 | 1.000 | 1.000 | 1.000 | 1.000 | 0.138 | 0.414 |
| Tibet | Linzhi | 1 | 163 | 1 | 17 | 674 | 6 | 6 | 1.000 | 0.333 | 3.000 | 1.000 | 1.000 | 1.000 | 1.000 | 0.138 | 0.414 |
| Tibet | Xigaze | 1 | 163 | 1 | 29 | 679 | 6 | 7 | 1.000 | 0.286 | 3.500 | 1.000 | 0.500 | 0.500 | 2.000 | 0.138 | 0.965 |
| Tibet | Naqu | 1 | 163 | 1 | 28 | 678 | 6 | 8 | 1.000 | 0.250 | 4.000 | 1.000 | 0.500 | 0.500 | 2.000 | 0.138 | 1.103 |
| Tibet | Lasa | 1 | 163 | 1 | 29 | N/A | 6 | 6 | 1.000 | 0.333 |  |  |  |  |  |  |  |
| Tibet | Lasa | 1 | 163 | 1 | 29 | N/A | 6 | 6 | 1.000 | 0.333 |  |  |  |  |  |  |  |
| Tibet | Naqu | 1 | 163 | 1 | 28 | N/A | 6 | 8 | 1.000 | 0.250 |  |  |  |  |  |  |  |
| Tibet | Xigaze | 1 | 163 | 1 | 29 | N/A | 6 | 7 | 1.000 | 0.286 |  |  |  |  |  |  |  |
| Tibet | Shannan | 1 | 163 | 1 | 18 | N/A | 6 | 5 | 1.000 | 0.400 |  |  |  |  |  |  |  |
| Tibet | Shannan | 1 | 163 | 1 | 18 | N/A | 6 | 5 | 1.000 | 0.400 |  |  |  |  |  |  |  |
| Tibet | Lasa | 1 | 162 | 1 | 29 | 671 | 6 | 4 | 1.000 | 0.500 | 2.000 | 0.964 | 1.000 | 0.964 | 1.037 | 0.138 | 0.286 |
| Tibet | Lasa | 1 | 162 | 1 | 29 | N/A | 6 | 4 | 1.000 | 0.500 |  |  |  |  |  |  |  |
| Tibet | Naqu | 1 | 162 | 1 | 28 | N/A | 6 | 1 | 1.000 | 1.000 |  |  |  |  |  |  |  |
| Tibet | Linzhi | 1 | 161 | 1 | 17 | 675 | 6 | 4 | 1.000 | 0.500 | 2.000 | 1.000 | 1.000 | 1.000 | 1.000 | 0.138 | 0.276 |
| Tibet | Linzhi | 1 | 161 | 1 | 17 | 673 | 6 | 4 | 1.000 | 0.500 | 2.000 | 1.000 | 1.000 | 1.000 | 1.000 | 0.138 | 0.276 |
| Tibet | Shannan | 1 | 161 | 1 | 18 | 682 | 6 | 5 | 1.000 | 0.400 | 2.500 | 1.000 | 1.000 | 1.000 | 1.000 | 0.138 | 0.345 |
| Tibet | Shannan | 1 | 161 | 1 | 18 | 681 | 6 | 5 | 1.000 | 0.400 | 2.500 | 1.000 | 1.000 | 1.000 | 1.000 | 0.138 | 0.345 |
| Tibet | Qamdo | 1 | 161 | 1 | 29 | 668 | 6 | 6 | 1.000 | 0.333 | 3.000 | 1.000 | 1.000 | 1.000 | 1.000 | 0.138 | 0.414 |
| Tibet | Lasa | 1 | 161 | 1 | 29 | 672 | 6 | 6 | 1.000 | 0.333 | 3.000 | 1.000 | 1.000 | 1.000 | 1.000 | 0.138 | 0.414 |
| Tibet | Xigaze | 1 | 161 | 1 | 29 | 680 | 6 | 8 | 1.000 | 0.250 | 4.000 | 1.000 | 1.000 | 1.000 | 1.000 | 0.138 | 0.551 |
| Tibet | Lasa | 1 | 161 | 1 | 29 | 670 | 6 | 6 | 1.000 | 0.333 | 3.000 | 0.600 | 1.000 | 0.600 | 1.667 | 0.138 | 0.689 |
| Tibet | Qamdo | 1 | 161 | 1 | 29 | 669 | 6 | 6 | 1.000 | 0.333 | 3.000 | 0.500 | 1.000 | 0.500 | 2.000 | 0.138 | 0.827 |
| Tibet | Naqu | 1 | 161 | 1 | 28 | 677 | 6 | 10 | 1.000 | 0.200 | 5.000 | 0.167 | 0.500 | 0.083 | 12.000 | 0.138 | 8.272 |
| Tibet | Naqu | 1 | 161 | 1 | 28 | N/A | 6 | 10 | 1.000 | 0.200 |  |  |  |  |  |  |  |
| Tibet | Xigaze | 1 | 161 | 1 | 29 | N/A | 6 | 8 | 1.000 | 0.250 |  |  |  |  |  |  |  |
| Tianjin | Tianjin | 3 | 160 | 209 | 420 | 659 | 1 | 42 | 1.000 | 0.357 | 2.800 | 1.000 | 1.000 | 1.000 | 1.000 | 0.411 | 1.150 |
| Tianjin | Tianjin | 3 | 160 | 209 | 420 | 657 | 1 | 42 | 1.000 | 0.357 | 2.800 | 0.750 | 1.000 | 0.750 | 1.333 | 0.411 | 1.533 |
| Tianjin | Tianjin | 3 | 160 | 209 | 420 | 661 | 1 | 42 | 1.000 | 0.357 | 2.800 | 0.400 | 1.000 | 0.400 | 2.500 | 0.411 | 2.874 |
| Tianjin | Tianjin | 3 | 160 | 209 | 420 | N/A | 1 | 42 | 1.000 | 0.357 |  |  |  |  |  |  |  |
| Tianjin | Tianjin | 3 | 160 | 209 | 420 | N/A | 1 | 42 | 1.000 | 0.357 |  |  |  |  |  |  |  |
| Tianjin | Tianjin | 3 | 160 | 209 | 420 | N/A | 1 | 42 | 1.000 | 0.357 |  |  |  |  |  |  |  |
| Tianjin | Tianjin | 3 | 160 | 209 | 420 | N/A | 1 | 42 | 1.000 | 0.357 |  |  |  |  |  |  |  |
| Tianjin | Tianjin | 3 | 160 | 209 | 420 | N/A | 1 | 42 | 1.000 | 0.357 |  |  |  |  |  |  |  |
| Tianjin | Tianjin | 3 | 160 | 209 | 420 | N/A | 1 | 42 | 1.000 | 0.357 |  |  |  |  |  |  |  |
| Tianjin | Tianjin | 3 | 160 | 209 | 420 | N/A | 1 | 42 | 1.000 | 0.357 |  |  |  |  |  |  |  |
| Tianjin | Tianjin | 3 | 160 | 209 | 420 | N/A | 1 | 42 | 1.000 | 0.357 |  |  |  |  |  |  |  |
| Tianjin | Tianjin | 3 | 160 | 209 | 420 | N/A | 1 | 42 | 1.000 | 0.357 |  |  |  |  |  |  |  |
| Tianjin | Tianjin | 3 | 160 | 209 | 420 | N/A | 1 | 42 | 1.000 | 0.357 |  |  |  |  |  |  |  |
| Tianjin | Tianjin | 3 | 160 | 209 | 420 | N/A | 1 | 42 | 1.000 | 0.357 |  |  |  |  |  |  |  |
| Tianjin | Tianjin | 3 | 160 | 209 | 420 | N/A | 1 | 42 | 1.000 | 0.357 |  |  |  |  |  |  |  |
| Tianjin | Tianjin | 3 | 159 | 209 | 420 | 665 | 1 | 50 | 1.000 | 0.220 | 4.545 | 1.000 | 0.556 | 0.556 | 1.800 | 0.411 | 3.359 |
| Tianjin | Tianjin | 3 | 159 | 209 | 420 | 664 | 1 | 50 | 1.000 | 0.220 | 4.545 | 1.000 | 0.556 | 0.556 | 1.800 | 0.411 | 3.359 |
| Tianjin | Tianjin | 3 | 159 | 209 | 420 | 663 | 1 | 50 | 1.000 | 0.220 | 4.545 | 1.000 | 0.556 | 0.556 | 1.800 | 0.411 | 3.359 |
| Tianjin | Tianjin | 3 | 159 | 209 | 420 | 656 | 1 | 50 | 1.000 | 0.220 | 4.545 | 1.000 | 0.556 | 0.556 | 1.800 | 0.411 | 3.359 |
| Tianjin | Tianjin | 3 | 159 | 209 | 420 | 660 | 1 | 50 | 1.000 | 0.220 | 4.545 | 0.933 | 0.556 | 0.519 | 1.929 | 0.411 | 3.599 |
| Tianjin | Tianjin | 3 | 159 | 209 | 420 | N/A | 1 | 50 | 1.000 | 0.220 |  |  |  |  |  |  |  |
| Tianjin | Tianjin | 3 | 159 | 209 | 420 | N/A | 1 | 50 | 1.000 | 0.220 |  |  |  |  |  |  |  |
| Tianjin | Tianjin | 3 | 159 | 209 | 420 | N/A | 1 | 50 | 1.000 | 0.220 |  |  |  |  |  |  |  |
| Tianjin | Tianjin | 3 | 159 | 209 | 420 | N/A | 1 | 50 | 1.000 | 0.220 |  |  |  |  |  |  |  |
| Tianjin | Tianjin | 3 | 159 | 209 | 420 | N/A | 1 | 50 | 1.000 | 0.220 |  |  |  |  |  |  |  |
| Tianjin | Tianjin | 3 | 159 | 209 | 420 | N/A | 1 | 50 | 1.000 | 0.220 |  |  |  |  |  |  |  |
| Tianjin | Tianjin | 3 | 158 | 209 | 420 | 662 | 1 | 47 | 1.000 | 0.213 | 4.700 | 1.000 | 0.500 | 0.500 | 2.000 | 0.411 | 3.859 |
| Tianjin | Tianjin | 3 | 158 | 209 | 420 | 658 | 1 | 47 | 1.000 | 0.213 | 4.700 | 1.000 | 0.500 | 0.500 | 2.000 | 0.411 | 3.859 |
| Tianjin | Tianjin | 3 | 158 | 209 | 420 | 655 | 1 | 47 | 1.000 | 0.213 | 4.700 | 0.917 | 0.500 | 0.458 | 2.182 | 0.411 | 4.210 |
| Tianjin | Tianjin | 3 | 158 | 209 | 420 | 653 | 1 | 47 | 1.000 | 0.213 | 4.700 | 0.818 | 0.500 | 0.409 | 2.444 | 0.411 | 4.717 |
| Tianjin | Tianjin | 3 | 158 | 209 | 420 | 654 | 1 | 47 | 1.000 | 0.213 | 4.700 | 0.636 | 0.500 | 0.318 | 3.143 | 0.411 | 6.065 |
| Tianjin | Tianjin | 3 | 158 | 209 | 420 | N/A | 1 | 47 | 1.000 | 0.213 |  |  |  |  |  |  |  |
| Tianjin | Tianjin | 3 | 158 | 209 | 420 | N/A | 1 | 47 | 1.000 | 0.213 |  |  |  |  |  |  |  |
| Tianjin | Tianjin | 3 | 158 | 209 | 420 | N/A | 1 | 47 | 1.000 | 0.213 |  |  |  |  |  |  |  |
| Tianjin | Tianjin | 3 | 158 | 209 | 420 | N/A | 1 | 47 | 1.000 | 0.213 |  |  |  |  |  |  |  |
| Tianjin | Tianjin | 3 | 158 | 209 | 420 | N/A | 1 | 47 | 1.000 | 0.213 |  |  |  |  |  |  |  |
| Sichuan | Chengdu | 3 | 157 | 626 | 892 | 626 | 3 | 72 | 0.333 | 0.028 | 108.000 | 1.000 | 1.000 | 1.000 | 1.000 | 0.904 | 97.637 |
| Sichuan | Chengdu | 3 | 157 | 626 | 892 | N/A | 3 | 72 | 0.333 | 0.028 |  |  |  |  |  |  |  |
| Sichuan | Chengdu | 3 | 156 | 626 | 892 | 628 | 3 | 40 | 0.333 | 0.050 | 60.000 | 1.000 | 1.000 | 1.000 | 1.000 | 0.904 | 54.243 |
| Sichuan | Chengdu | 3 | 156 | 626 | 892 | N/A | 3 | 40 | 0.333 | 0.050 |  |  |  |  |  |  |  |
| Sichuan | Chengdu | 3 | 155 | 626 | 892 | 627 | 3 | 60 | 0.333 | 0.033 | 90.000 | 1.000 | 1.000 | 1.000 | 1.000 | 0.904 | 81.364 |
| Sichuan | Chengdu | 3 | 155 | 626 | 892 | 625 | 3 | 60 | 0.333 | 0.033 | 90.000 | 1.000 | 1.000 | 1.000 | 1.000 | 0.904 | 81.364 |
| Sichuan | Ya'an | 2 | 154 | 626 | 43 | 652 | 14 | 7 | 0.357 | 0.286 | 9.800 | 1.000 | 1.000 | 1.000 | 1.000 | 0.904 | 8.860 |
| Sichuan | Ya'an | 2 | 154 | 626 | 43 | 650 | 14 | 7 | 0.357 | 0.286 | 9.800 | 1.000 | 1.000 | 1.000 | 1.000 | 0.904 | 8.860 |
| Sichuan | Meishan | 2 | 154 | 626 | 88 | 639 | 14 | 15 | 0.357 | 0.133 | 21.000 | 1.000 | 1.000 | 1.000 | 1.000 | 0.904 | 18.985 |
| Sichuan | Meishan | 2 | 154 | 626 | 88 | 642 | 14 | 15 | 0.357 | 0.133 | 21.000 | 0.909 | 1.000 | 0.909 | 1.100 | 0.904 | 20.884 |
| Sichuan | Guang'an | 2 | 154 | 626 | 73 | 635 | 14 | 23 | 0.357 | 0.087 | 32.200 | 0.917 | 1.000 | 0.917 | 1.091 | 0.904 | 31.757 |
| Sichuan | Guang'an | 2 | 154 | 626 | 73 | 636 | 14 | 23 | 0.357 | 0.087 | 32.200 | 0.900 | 1.000 | 0.900 | 1.111 | 0.904 | 32.345 |
| Sichuan | Mianyang | 2 | 154 | 626 | 117 | 644 | 14 | 13 | 0.357 | 0.154 | 18.200 | 1.000 | 0.500 | 0.500 | 2.000 | 0.904 | 32.907 |
| Sichuan | Deyang | 2 | 154 | 626 | 90 | 632 | 14 | 15 | 0.357 | 0.067 | 42.000 | 0.895 | 1.000 | 0.895 | 1.118 | 0.904 | 42.437 |
| Sichuan | Mianyang | 2 | 154 | 626 | 117 | N/A | 14 | 13 | 0.357 | 0.154 |  |  |  |  |  |  |  |
| Sichuan | Guang'an | 2 | 153 | 626 | 73 | 634 | 14 | 1 | 0.357 | 1.000 | 2.800 | 1.000 | 1.000 | 1.000 | 1.000 | 0.904 | 2.531 |
| Sichuan | Meishan | 2 | 153 | 626 | 88 | 640 | 14 | 1 | 0.357 | 1.000 | 2.800 | 1.000 | 1.000 | 1.000 | 1.000 | 0.904 | 2.531 |
| Sichuan | Ya'an | 2 | 153 | 626 | 43 | 651 | 14 | 1 | 0.357 | 1.000 | 2.800 | 1.000 | 1.000 | 1.000 | 1.000 | 0.904 | 2.531 |
| Sichuan | Deyang | 2 | 153 | 626 | 90 | 633 | 14 | 3 | 0.357 | 0.667 | 4.200 | 1.000 | 1.000 | 1.000 | 1.000 | 0.904 | 3.797 |
| Sichuan | Deyang | 2 | 153 | 626 | 90 | 630 | 14 | 3 | 0.357 | 0.667 | 4.200 | 1.000 | 1.000 | 1.000 | 1.000 | 0.904 | 3.797 |
| Sichuan | Mianyang | 2 | 153 | 626 | 117 | 648 | 14 | 4 | 0.357 | 0.500 | 5.600 | 1.000 | 1.000 | 1.000 | 1.000 | 0.904 | 5.063 |
| Sichuan | Mianyang | 2 | 153 | 626 | 117 | 647 | 14 | 4 | 0.357 | 0.500 | 5.600 | 1.000 | 1.000 | 1.000 | 1.000 | 0.904 | 5.063 |
| Sichuan | Deyang | 2 | 152 | 626 | 90 | 631 | 14 | 9 | 0.357 | 0.222 | 12.600 | 1.000 | 1.000 | 1.000 | 1.000 | 0.904 | 11.391 |
| Sichuan | Deyang | 2 | 152 | 626 | 90 | 629 | 14 | 9 | 0.357 | 0.222 | 12.600 | 1.000 | 1.000 | 1.000 | 1.000 | 0.904 | 11.391 |
| Sichuan | Meishan | 2 | 152 | 626 | 88 | 641 | 14 | 9 | 0.357 | 0.222 | 12.600 | 1.000 | 1.000 | 1.000 | 1.000 | 0.904 | 11.391 |
| Sichuan | Guang'an | 2 | 152 | 626 | 73 | 638 | 14 | 10 | 0.357 | 0.200 | 14.000 | 1.000 | 1.000 | 1.000 | 1.000 | 0.904 | 12.657 |
| Sichuan | Guang'an | 2 | 152 | 626 | 73 | 637 | 14 | 10 | 0.357 | 0.200 | 14.000 | 1.000 | 1.000 | 1.000 | 1.000 | 0.904 | 12.657 |
| Sichuan | Meishan | 2 | 152 | 626 | 88 | 643 | 14 | 9 | 0.357 | 0.222 | 12.600 | 0.900 | 1.000 | 0.900 | 1.111 | 0.904 | 12.657 |
| Sichuan | Ya'an | 2 | 152 | 626 | 43 | 649 | 14 | 6 | 0.357 | 0.333 | 8.400 | 1.000 | 0.500 | 0.500 | 2.000 | 0.904 | 15.188 |
| Sichuan | Mianyang | 2 | 152 | 626 | 117 | 646 | 14 | 19 | 0.357 | 0.105 | 26.600 | 1.000 | 1.000 | 1.000 | 1.000 | 0.904 | 24.048 |
| Sichuan | Mianyang | 2 | 152 | 626 | 117 | 645 | 14 | 19 | 0.357 | 0.105 | 26.600 | 1.000 | 1.000 | 1.000 | 1.000 | 0.904 | 24.048 |
| Sichuan | Ya'an | 2 | 152 | 626 | 43 | N/A | 14 | 6 | 0.357 | 0.333 |  |  |  |  |  |  |  |
| Shanghai | Shanghai | 3 | 151 | 842 | 364 | 620 | 1 | 47 | 1.000 | 0.255 | 3.917 | 1.000 | 1.000 | 1.000 | 1.000 | 0.771 | 3.018 |
| Shanghai | Shanghai | 3 | 151 | 842 | 364 | 603 | 1 | 47 | 1.000 | 0.255 | 3.917 | 0.750 | 1.000 | 0.750 | 1.333 | 0.771 | 4.024 |
| Shanghai | Shanghai | 3 | 151 | 842 | 364 | 617 | 1 | 47 | 1.000 | 0.255 | 3.917 | 0.667 | 1.000 | 0.667 | 1.500 | 0.771 | 4.527 |
| Shanghai | Shanghai | 3 | 151 | 842 | 364 | 612 | 1 | 47 | 1.000 | 0.255 | 3.917 | 0.500 | 1.000 | 0.500 | 2.000 | 0.771 | 6.036 |
| Shanghai | Shanghai | 3 | 151 | 842 | 364 | 611 | 1 | 47 | 1.000 | 0.255 | 3.917 | 0.500 | 1.000 | 0.500 | 2.000 | 0.771 | 6.036 |
| Shanghai | Shanghai | 3 | 151 | 842 | 364 | 604 | 1 | 47 | 1.000 | 0.255 | 3.917 | 0.500 | 1.000 | 0.500 | 2.000 | 0.771 | 6.036 |
| Shanghai | Shanghai | 3 | 151 | 842 | 364 | N/A | 1 | 47 | 1.000 | 0.255 |  |  |  |  |  |  |  |
| Shanghai | Shanghai | 3 | 151 | 842 | 364 | N/A | 1 | 47 | 1.000 | 0.255 |  |  |  |  |  |  |  |
| Shanghai | Shanghai | 3 | 151 | 842 | 364 | N/A | 1 | 47 | 1.000 | 0.255 |  |  |  |  |  |  |  |
| Shanghai | Shanghai | 3 | 151 | 842 | 364 | N/A | 1 | 47 | 1.000 | 0.255 |  |  |  |  |  |  |  |
| Shanghai | Shanghai | 3 | 151 | 842 | 364 | N/A | 1 | 47 | 1.000 | 0.255 |  |  |  |  |  |  |  |
| Shanghai | Shanghai | 3 | 151 | 842 | 364 | N/A | 1 | 47 | 1.000 | 0.255 |  |  |  |  |  |  |  |
| Shanghai | Shanghai | 3 | 150 | 842 | 364 | 621 | 1 | 88 | 1.000 | 0.136 | 7.333 | 1.000 | 0.833 | 0.833 | 1.200 | 0.771 | 6.781 |
| Shanghai | Shanghai | 3 | 150 | 842 | 364 | 619 | 1 | 88 | 1.000 | 0.136 | 7.333 | 1.000 | 0.833 | 0.833 | 1.200 | 0.771 | 6.781 |
| Shanghai | Shanghai | 3 | 150 | 842 | 364 | 615 | 1 | 88 | 1.000 | 0.136 | 7.333 | 1.000 | 0.833 | 0.833 | 1.200 | 0.771 | 6.781 |
| Shanghai | Shanghai | 3 | 150 | 842 | 364 | 614 | 1 | 88 | 1.000 | 0.136 | 7.333 | 1.000 | 0.833 | 0.833 | 1.200 | 0.771 | 6.781 |
| Shanghai | Shanghai | 3 | 150 | 842 | 364 | 608 | 1 | 88 | 1.000 | 0.136 | 7.333 | 1.000 | 0.833 | 0.833 | 1.200 | 0.771 | 6.781 |
| Shanghai | Shanghai | 3 | 150 | 842 | 364 | 607 | 1 | 88 | 1.000 | 0.136 | 7.333 | 1.000 | 0.833 | 0.833 | 1.200 | 0.771 | 6.781 |
| Shanghai | Shanghai | 3 | 150 | 842 | 364 | 606 | 1 | 88 | 1.000 | 0.136 | 7.333 | 1.000 | 0.833 | 0.833 | 1.200 | 0.771 | 6.781 |
| Shanghai | Shanghai | 3 | 150 | 842 | 364 | 605 | 1 | 88 | 1.000 | 0.136 | 7.333 | 1.000 | 0.833 | 0.833 | 1.200 | 0.771 | 6.781 |
| Shanghai | Shanghai | 3 | 150 | 842 | 364 | 601 | 1 | 88 | 1.000 | 0.136 | 7.333 | 0.938 | 0.833 | 0.781 | 1.280 | 0.771 | 7.233 |
| Shanghai | Shanghai | 3 | 150 | 842 | 364 | 600 | 1 | 88 | 1.000 | 0.136 | 7.333 | 0.867 | 0.833 | 0.722 | 1.385 | 0.771 | 7.825 |
| Shanghai | Shanghai | 3 | 150 | 842 | 364 | N/A | 1 | 88 | 1.000 | 0.136 |  |  |  |  |  |  |  |
| Shanghai | Shanghai | 3 | 150 | 842 | 364 | N/A | 1 | 88 | 1.000 | 0.136 |  |  |  |  |  |  |  |
| Shanghai | Shanghai | 3 | 149 | 842 | 364 | 624 | 1 | 103 | 1.000 | 0.117 | 8.583 | 1.000 | 0.750 | 0.750 | 1.333 | 0.771 | 8.819 |
| Shanghai | Shanghai | 3 | 149 | 842 | 364 | 623 | 1 | 103 | 1.000 | 0.117 | 8.583 | 1.000 | 0.750 | 0.750 | 1.333 | 0.771 | 8.819 |
| Shanghai | Shanghai | 3 | 149 | 842 | 364 | 622 | 1 | 103 | 1.000 | 0.117 | 8.583 | 1.000 | 0.750 | 0.750 | 1.333 | 0.771 | 8.819 |
| Shanghai | Shanghai | 3 | 149 | 842 | 364 | 616 | 1 | 103 | 1.000 | 0.117 | 8.583 | 1.000 | 0.750 | 0.750 | 1.333 | 0.771 | 8.819 |
| Shanghai | Shanghai | 3 | 149 | 842 | 364 | 613 | 1 | 103 | 1.000 | 0.117 | 8.583 | 1.000 | 0.750 | 0.750 | 1.333 | 0.771 | 8.819 |
| Shanghai | Shanghai | 3 | 149 | 842 | 364 | 610 | 1 | 103 | 1.000 | 0.117 | 8.583 | 1.000 | 0.750 | 0.750 | 1.333 | 0.771 | 8.819 |
| Shanghai | Shanghai | 3 | 149 | 842 | 364 | 609 | 1 | 103 | 1.000 | 0.117 | 8.583 | 1.000 | 0.750 | 0.750 | 1.333 | 0.771 | 8.819 |
| Shanghai | Shanghai | 3 | 149 | 842 | 364 | 602 | 1 | 103 | 1.000 | 0.117 | 8.583 | 1.000 | 0.750 | 0.750 | 1.333 | 0.771 | 8.819 |
| Shanghai | Shanghai | 3 | 149 | 842 | 364 | 618 | 1 | 103 | 1.000 | 0.117 | 8.583 | 0.600 | 0.750 | 0.450 | 2.222 | 0.771 | 14.699 |
| Shanghai | Shanghai | 3 | 149 | 842 | 364 | N/A | 1 | 103 | 1.000 | 0.117 |  |  |  |  |  |  |  |
| Shanghai | Shanghai | 3 | 149 | 842 | 364 | N/A | 1 | 103 | 1.000 | 0.117 |  |  |  |  |  |  |  |
| Shanghai | Shanghai | 3 | 149 | 842 | 364 | N/A | 1 | 103 | 1.000 | 0.117 |  |  |  |  |  |  |  |
| Shaanxi | Xi'an | 3 | 148 | 356 | 343 | 589 | 1 | 50 | 1.000 | 0.040 | 25.000 | 1.000 | 1.000 | 1.000 | 1.000 | 0.758 | 18.942 |
| Shaanxi | Xi'an | 3 | 148 | 356 | 343 | N/A | 1 | 50 | 1.000 | 0.040 |  |  |  |  |  |  |  |
| Shaanxi | Xi'an | 3 | 147 | 356 | 343 | 588 | 1 | 21 | 1.000 | 0.095 | 10.500 | 1.000 | 1.000 | 1.000 | 1.000 | 0.758 | 7.955 |
| Shaanxi | Xi'an | 3 | 147 | 356 | 343 | 587 | 1 | 21 | 1.000 | 0.095 | 10.500 | 1.000 | 1.000 | 1.000 | 1.000 | 0.758 | 7.955 |
| Shaanxi | Xi'an | 3 | 146 | 356 | 343 | N/A | 1 | 40 | 1.000 | 0.050 |  |  |  |  |  |  |  |
| Shaanxi | Xi'an | 3 | 146 | 356 | 343 | N/A | 1 | 40 | 1.000 | 0.050 |  |  |  |  |  |  |  |
| Shaanxi | Yulin | 2 | 145 | 356 | 104 | 597 | 8 | 8 | 0.625 | 0.250 | 6.400 | 0.857 | 1.000 | 0.857 | 1.167 | 0.758 | 5.657 |
| Shaanxi | Ankang | 2 | 145 | 356 | 50 | 577 | 8 | 7 | 0.625 | 0.286 | 5.600 | 1.000 | 0.500 | 0.500 | 2.000 | 0.758 | 8.486 |
| Shaanxi | Yulin | 2 | 145 | 356 | 104 | 598 | 8 | 8 | 0.625 | 0.250 | 6.400 | 0.556 | 1.000 | 0.556 | 1.800 | 0.758 | 8.728 |
| Shaanxi | Yan'an | 2 | 145 | 356 | 61 | 591 | 8 | 13 | 0.625 | 0.154 | 10.400 | 0.750 | 1.000 | 0.750 | 1.333 | 0.758 | 10.506 |
| Shaanxi | Yan'an | 2 | 145 | 356 | 61 | 592 | 8 | 13 | 0.625 | 0.154 | 10.400 | 0.500 | 1.000 | 0.500 | 2.000 | 0.758 | 15.759 |
| Shaanxi | Shangluo | 2 | 145 | 356 | 180 | 581 | 8 | 15 | 0.625 | 0.133 | 12.000 | 1.000 | 0.500 | 0.500 | 2.000 | 0.758 | 18.184 |
| Shaanxi | Weinan | 2 | 145 | 356 | 163 | 585 | 8 | 26 | 0.625 | 0.077 | 20.800 | 0.833 | 1.000 | 0.833 | 1.200 | 0.758 | 18.911 |
| Shaanxi | Weinan | 2 | 145 | 356 | 163 | 583 | 8 | 26 | 0.625 | 0.077 | 20.800 | 0.250 | 1.000 | 0.250 | 4.000 | 0.758 | 63.037 |
| Shaanxi | Ankang | 2 | 145 | 356 | 50 | N/A | 8 | 7 | 0.625 | 0.286 |  |  |  |  |  |  |  |
| Shaanxi | Shangluo | 2 | 145 | 356 | 180 | N/A | 8 | 15 | 0.625 | 0.133 |  |  |  |  |  |  |  |
| Shaanxi | Ankang | 2 | 144 | 356 | 50 | 576 | 8 | 2 | 0.625 | 1.000 | 1.600 | 1.000 | 1.000 | 1.000 | 1.000 | 0.758 | 1.212 |
| Shaanxi | Ankang | 2 | 144 | 356 | 50 | 575 | 8 | 2 | 0.625 | 1.000 | 1.600 | 1.000 | 1.000 | 1.000 | 1.000 | 0.758 | 1.212 |
| Shaanxi | Shangluo | 2 | 144 | 356 | 180 | 579 | 8 | 1 | 0.625 | 1.000 | 1.600 | 0.923 | 1.000 | 0.923 | 1.083 | 0.758 | 1.313 |
| Shaanxi | Weinan | 2 | 144 | 356 | 163 | 586 | 8 | 3 | 0.625 | 0.667 | 2.400 | 1.000 | 1.000 | 1.000 | 1.000 | 0.758 | 1.818 |
| Shaanxi | Weinan | 2 | 144 | 356 | 163 | 584 | 8 | 3 | 0.625 | 0.667 | 2.400 | 1.000 | 1.000 | 1.000 | 1.000 | 0.758 | 1.818 |
| Shaanxi | Yulin | 2 | 144 | 356 | 104 | 596 | 8 | 2 | 0.625 | 1.000 | 1.600 | 1.000 | 0.500 | 0.500 | 2.000 | 0.758 | 2.425 |
| Shaanxi | Yan'an | 2 | 144 | 356 | 61 | 593 | 8 | 4 | 0.625 | 0.500 | 3.200 | 0.980 | 1.000 | 0.980 | 1.020 | 0.758 | 2.473 |
| Shaanxi | Yan'an | 2 | 144 | 356 | 61 | 595 | 8 | 4 | 0.625 | 0.500 | 3.200 | 0.333 | 1.000 | 0.333 | 3.000 | 0.758 | 7.274 |
| Shaanxi | Yulin | 2 | 144 | 356 | 104 | N/A | 8 | 2 | 0.625 | 1.000 |  |  |  |  |  |  |  |
| Shaanxi | Ankang | 2 | 143 | 356 | 50 | 574 | 8 | 8 | 0.625 | 0.250 | 6.400 | 1.000 | 1.000 | 1.000 | 1.000 | 0.758 | 4.849 |
| Shaanxi | Ankang | 2 | 143 | 356 | 50 | 573 | 8 | 8 | 0.625 | 0.250 | 6.400 | 1.000 | 1.000 | 1.000 | 1.000 | 0.758 | 4.849 |
| Shaanxi | Yan'an | 2 | 143 | 356 | 61 | 590 | 8 | 12 | 0.625 | 0.167 | 9.600 | 0.818 | 1.000 | 0.818 | 1.222 | 0.758 | 8.890 |
| Shaanxi | Shangluo | 2 | 143 | 356 | 180 | 580 | 8 | 16 | 0.625 | 0.125 | 12.800 | 1.000 | 1.000 | 1.000 | 1.000 | 0.758 | 9.698 |
| Shaanxi | Shangluo | 2 | 143 | 356 | 180 | 578 | 8 | 16 | 0.625 | 0.125 | 12.800 | 1.000 | 1.000 | 1.000 | 1.000 | 0.758 | 9.698 |
| Shaanxi | Yan'an | 2 | 143 | 356 | 61 | 594 | 8 | 12 | 0.625 | 0.167 | 9.600 | 0.667 | 1.000 | 0.667 | 1.500 | 0.758 | 10.910 |
| Shaanxi | Yulin | 2 | 143 | 356 | 104 | 599 | 8 | 10 | 0.625 | 0.200 | 8.000 | 0.923 | 0.500 | 0.462 | 2.167 | 0.758 | 13.133 |
| Shaanxi | Weinan | 2 | 143 | 356 | 163 | 582 | 8 | 20 | 0.625 | 0.100 | 16.000 | 0.889 | 0.500 | 0.444 | 2.250 | 0.758 | 27.276 |
| Shaanxi | Weinan | 2 | 143 | 356 | 163 | N/A | 8 | 20 | 0.625 | 0.100 |  |  |  |  |  |  |  |
| Shaanxi | Yulin | 2 | 143 | 356 | 104 | N/A | 8 | 10 | 0.625 | 0.200 |  |  |  |  |  |  |  |
| Shanxi | Lin fen | 2 | 142 | 201 | 186 | 554 | 5 | 27 | 0.400 | 0.074 | 33.750 | 0.667 | 1.000 | 0.667 | 1.500 | 0.868 | 43.921 |
| Shanxi | Lin fen | 2 | 142 | 201 | 186 | 553 | 5 | 27 | 0.400 | 0.074 | 33.750 | 0.667 | 1.000 | 0.667 | 1.500 | 0.868 | 43.921 |
| Shanxi | Datong | 2 | 142 | 201 | 122 | 544 | 5 | 19 | 0.400 | 0.105 | 23.750 | 0.889 | 0.500 | 0.444 | 2.250 | 0.868 | 46.361 |
| Shanxi | Datong | 2 | 142 | 201 | 122 | N/A | 5 | 19 | 0.400 | 0.105 |  |  |  |  |  |  |  |
| Shanxi | Datong | 2 | 141 | 201 | 122 | 542 | 5 | 5 | 0.400 | 0.400 | 6.250 | 1.000 | 1.000 | 1.000 | 1.000 | 0.868 | 5.422 |
| Shanxi | Datong | 2 | 141 | 201 | 122 | 540 | 5 | 5 | 0.400 | 0.400 | 6.250 | 1.000 | 1.000 | 1.000 | 1.000 | 0.868 | 5.422 |
| Shanxi | Lin fen | 2 | 141 | 201 | 186 | 551 | 5 | 5 | 0.400 | 0.400 | 6.250 | 0.941 | 1.000 | 0.941 | 1.063 | 0.868 | 5.761 |
| Shanxi | Lin fen | 2 | 141 | 201 | 186 | 552 | 5 | 5 | 0.400 | 0.400 | 6.250 | 0.933 | 1.000 | 0.933 | 1.071 | 0.868 | 5.810 |
| Shanxi | Datong | 2 | 140 | 201 | 122 | 541 | 5 | 18 | 0.400 | 0.111 | 22.500 | 0.909 | 1.000 | 0.909 | 1.100 | 0.868 | 21.473 |
| Shanxi | Lin fen | 2 | 140 | 201 | 186 | 550 | 5 | 24 | 0.400 | 0.083 | 30.000 | 1.000 | 1.000 | 1.000 | 1.000 | 0.868 | 26.027 |
| Shanxi | Datong | 2 | 140 | 201 | 122 | 543 | 5 | 18 | 0.400 | 0.111 | 22.500 | 0.733 | 1.000 | 0.733 | 1.364 | 0.868 | 26.619 |
| Shanxi | Lin fen | 2 | 140 | 201 | 186 | 549 | 5 | 24 | 0.400 | 0.083 | 30.000 | 0.833 | 1.000 | 0.833 | 1.200 | 0.868 | 31.233 |
| Shanxi | Yangquan | 1 | 139 | 201 | 49 | 572 | 5 | 8 | 0.800 | 0.250 | 5.000 | 1.000 | 1.000 | 1.000 | 1.000 | 0.868 | 4.338 |
| Shanxi | Yangquan | 1 | 139 | 201 | 49 | 568 | 5 | 8 | 0.800 | 0.250 | 5.000 | 0.667 | 1.000 | 0.667 | 1.500 | 0.868 | 6.507 |
| Shanxi | Jincheng | 1 | 139 | 201 | 85 | 548 | 5 | 15 | 0.800 | 0.133 | 9.375 | 1.000 | 1.000 | 1.000 | 1.000 | 0.868 | 8.134 |
| Shanxi | Xinzhou | 1 | 139 | 201 | 110 | 563 | 5 | 15 | 0.800 | 0.133 | 9.375 | 1.000 | 1.000 | 1.000 | 1.000 | 0.868 | 8.134 |
| Shanxi | Xinzhou | 1 | 139 | 201 | 110 | 561 | 5 | 15 | 0.800 | 0.133 | 9.375 | 1.000 | 1.000 | 1.000 | 1.000 | 0.868 | 8.134 |
| Shanxi | Jincheng | 1 | 139 | 201 | 85 | 546 | 5 | 15 | 0.800 | 0.133 | 9.375 | 0.857 | 1.000 | 0.857 | 1.167 | 0.868 | 9.489 |
| Shanxi | Lvliang | 1 | 139 | 201 | 103 | 559 | 5 | 21 | 0.800 | 0.095 | 13.125 | 0.800 | 1.000 | 0.800 | 1.250 | 0.868 | 14.234 |
| Shanxi | Lvliang | 1 | 139 | 201 | 103 | 558 | 5 | 21 | 0.800 | 0.095 | 13.125 | 0.636 | 1.000 | 0.636 | 1.571 | 0.868 | 17.894 |
| Shanxi | Yangquan | 1 | 138 | 201 | 49 | 570 | 5 | 2 | 0.800 | 1.000 | 1.250 | 1.000 | 1.000 | 1.000 | 1.000 | 0.868 | 1.084 |
| Shanxi | Yangquan | 1 | 138 | 201 | 49 | 569 | 5 | 2 | 0.800 | 1.000 | 1.250 | 1.000 | 1.000 | 1.000 | 1.000 | 0.868 | 1.084 |
| Shanxi | Xinzhou | 1 | 138 | 201 | 110 | 564 | 5 | 3 | 0.800 | 0.667 | 1.875 | 1.000 | 1.000 | 1.000 | 1.000 | 0.868 | 1.627 |
| Shanxi | Xinzhou | 1 | 138 | 201 | 110 | 562 | 5 | 3 | 0.800 | 0.667 | 1.875 | 1.000 | 1.000 | 1.000 | 1.000 | 0.868 | 1.627 |
| Shanxi | Jincheng | 1 | 138 | 201 | 85 | 547 | 5 | 4 | 0.800 | 0.500 | 2.500 | 1.000 | 1.000 | 1.000 | 1.000 | 0.868 | 2.169 |
| Shanxi | Jincheng | 1 | 138 | 201 | 85 | 545 | 5 | 4 | 0.800 | 0.500 | 2.500 | 1.000 | 1.000 | 1.000 | 1.000 | 0.868 | 2.169 |
| Shanxi | Lvliang | 1 | 138 | 201 | 103 | 557 | 5 | 4 | 0.800 | 0.500 | 2.500 | 1.000 | 1.000 | 1.000 | 1.000 | 0.868 | 2.169 |
| Shanxi | Lvliang | 1 | 138 | 201 | 103 | 556 | 5 | 4 | 0.800 | 0.500 | 2.500 | 1.000 | 1.000 | 1.000 | 1.000 | 0.868 | 2.169 |
| Shanxi | Xinzhou | 1 | 137 | 201 | 110 | 566 | 5 | 11 | 0.800 | 0.182 | 6.875 | 1.000 | 1.000 | 1.000 | 1.000 | 0.868 | 5.965 |
| Shanxi | Xinzhou | 1 | 137 | 201 | 110 | 565 | 5 | 11 | 0.800 | 0.182 | 6.875 | 0.818 | 1.000 | 0.818 | 1.222 | 0.868 | 7.290 |
| Shanxi | Yangquan | 1 | 137 | 201 | 49 | 567 | 5 | 15 | 0.800 | 0.133 | 9.375 | 1.000 | 1.000 | 1.000 | 1.000 | 0.868 | 8.134 |
| Shanxi | Lvliang | 1 | 137 | 201 | 103 | 560 | 5 | 19 | 0.800 | 0.105 | 11.875 | 1.000 | 1.000 | 1.000 | 1.000 | 0.868 | 10.303 |
| Shanxi | Lvliang | 1 | 137 | 201 | 103 | 555 | 5 | 19 | 0.800 | 0.105 | 11.875 | 1.000 | 1.000 | 1.000 | 1.000 | 0.868 | 10.303 |
| Shanxi | Yangquan | 1 | 137 | 201 | 49 | 571 | 5 | 15 | 0.800 | 0.133 | 9.375 | 0.250 | 1.000 | 0.250 | 4.000 | 0.868 | 32.534 |
| Shanxi | Jincheng | 1 | 137 | 201 | 85 | N/A | 5 | 12 | 0.800 | 0.167 |  |  |  |  |  |  |  |
| Shanxi | Jincheng | 1 | 137 | 201 | 85 | N/A | 5 | 12 | 0.800 | 0.167 |  |  |  |  |  |  |  |
| Shandong | Qingdao | 3 | 136 | 822 | 421 | 521 | 7 | 30 | 0.286 | 0.067 | 52.500 | 1.000 | 1.000 | 1.000 | 1.000 | 1.879 | 98.648 |
| Shandong | Jinan | 3 | 136 | 822 | 246 | 514 | 7 | 31 | 0.286 | 0.065 | 54.250 | 1.000 | 1.000 | 1.000 | 1.000 | 1.879 | 101.936 |
| Shandong | Qingdao | 3 | 136 | 822 | 421 | 523 | 7 | 30 | 0.286 | 0.067 | 52.500 | 0.833 | 1.000 | 0.833 | 1.200 | 1.879 | 118.377 |
| Shandong | Jinan | 3 | 136 | 822 | 246 | 512 | 7 | 31 | 0.286 | 0.065 | 54.250 | 0.667 | 1.000 | 0.667 | 1.500 | 1.879 | 152.904 |
| Shandong | Jinan | 3 | 135 | 822 | 246 | 511 | 7 | 24 | 0.286 | 0.083 | 42.000 | 1.000 | 1.000 | 1.000 | 1.000 | 1.879 | 78.918 |
| Shandong | Jinan | 3 | 135 | 822 | 246 | 513 | 7 | 24 | 0.286 | 0.083 | 42.000 | 0.984 | 1.000 | 0.984 | 1.017 | 1.879 | 80.233 |
| Shandong | Qingdao | 3 | 135 | 822 | 421 | 524 | 7 | 10 | 0.286 | 0.200 | 17.500 | 0.714 | 0.500 | 0.357 | 2.800 | 1.879 | 92.071 |
| Shandong | Qingdao | 3 | 135 | 822 | 421 | N/A | 7 | 10 | 0.286 | 0.200 |  |  |  |  |  |  |  |
| Shandong | Qingdao | 3 | 134 | 822 | 421 | 522 | 7 | 21 | 0.286 | 0.095 | 36.750 | 1.000 | 1.000 | 1.000 | 1.000 | 1.879 | 69.053 |
| Shandong | Qingdao | 3 | 134 | 822 | 421 | 520 | 7 | 21 | 0.286 | 0.095 | 36.750 | 1.000 | 1.000 | 1.000 | 1.000 | 1.879 | 69.053 |
| Shandong | Jinan | 3 | 134 | 822 | 246 | 510 | 7 | 30 | 0.286 | 0.067 | 52.500 | 1.000 | 0.500 | 0.500 | 2.000 | 1.879 | 197.295 |
| Shandong | Jinan | 3 | 134 | 822 | 246 | N/A | 7 | 30 | 0.286 | 0.067 |  |  |  |  |  |  |  |
| Shandong | Zaozhuang | 2 | 133 | 822 | 82 | 534 | 9 | 15 | 0.444 | 0.067 | 33.750 | 0.750 | 1.000 | 0.750 | 1.333 | 1.879 | 84.555 |
| Shandong | Zibo | 2 | 133 | 822 | 160 | 539 | 9 | 21 | 0.444 | 0.095 | 23.625 | 0.778 | 0.500 | 0.389 | 2.571 | 1.879 | 114.149 |
| Shandong | Liaocheng | 2 | 133 | 822 | 159 | 516 | 9 | 21 | 0.444 | 0.095 | 23.625 | 0.500 | 0.500 | 0.250 | 4.000 | 1.879 | 177.566 |
| Shandong | Tai'an | 2 | 133 | 822 | 105 | 525 | 9 | 26 | 0.444 | 0.077 | 29.250 | 0.167 | 1.000 | 0.167 | 6.000 | 1.879 | 329.765 |
| Shandong | Liaocheng | 2 | 133 | 822 | 159 | N/A | 9 | 21 | 0.444 | 0.095 |  |  |  |  |  |  |  |
| Shandong | Tai'an | 2 | 133 | 822 | 105 | N/A | 9 | 26 | 0.444 | 0.077 |  |  |  |  |  |  |  |
| Shandong | Zibo | 2 | 133 | 822 | 160 | N/A | 9 | 21 | 0.444 | 0.095 |  |  |  |  |  |  |  |
| Shandong | Zaozhuang | 2 | 132 | 822 | 82 | 530 | 9 | 4 | 0.444 | 0.500 | 4.500 | 1.000 | 1.000 | 1.000 | 1.000 | 1.879 | 8.456 |
| Shandong | Zibo | 2 | 132 | 822 | 160 | 537 | 9 | 4 | 0.444 | 0.500 | 4.500 | 1.000 | 1.000 | 1.000 | 1.000 | 1.879 | 8.456 |
| Shandong | Zaozhuang | 2 | 132 | 822 | 82 | 531 | 9 | 4 | 0.444 | 0.500 | 4.500 | 0.923 | 1.000 | 0.923 | 1.083 | 1.879 | 9.160 |
| Shandong | Zibo | 2 | 132 | 822 | 160 | 536 | 9 | 4 | 0.444 | 0.500 | 4.500 | 0.889 | 1.000 | 0.889 | 1.125 | 1.879 | 9.512 |
| Shandong | Liaocheng | 2 | 132 | 822 | 159 | 519 | 9 | 5 | 0.444 | 0.400 | 5.625 | 1.000 | 1.000 | 1.000 | 1.000 | 1.879 | 10.569 |
| Shandong | Liaocheng | 2 | 132 | 822 | 159 | 518 | 9 | 5 | 0.444 | 0.400 | 5.625 | 1.000 | 1.000 | 1.000 | 1.000 | 1.879 | 10.569 |
| Shandong | Tai'an | 2 | 132 | 822 | 105 | 529 | 9 | 5 | 0.444 | 0.400 | 5.625 | 1.000 | 1.000 | 1.000 | 1.000 | 1.879 | 10.569 |
| Shandong | Tai'an | 2 | 132 | 822 | 105 | 528 | 9 | 5 | 0.444 | 0.400 | 5.625 | 1.000 | 1.000 | 1.000 | 1.000 | 1.879 | 10.569 |
| Shandong | Zaozhuang | 2 | 131 | 822 | 82 | 532 | 9 | 11 | 0.444 | 0.273 | 8.250 | 1.000 | 0.667 | 0.667 | 1.500 | 1.879 | 23.253 |
| Shandong | Tai'an | 2 | 131 | 822 | 105 | 526 | 9 | 15 | 0.444 | 0.133 | 16.875 | 1.000 | 1.000 | 1.000 | 1.000 | 1.879 | 31.708 |
| Shandong | Zaozhuang | 2 | 131 | 822 | 82 | 533 | 9 | 11 | 0.444 | 0.273 | 8.250 | 0.625 | 0.667 | 0.417 | 2.400 | 1.879 | 37.204 |
| Shandong | Liaocheng | 2 | 131 | 822 | 159 | 517 | 9 | 20 | 0.444 | 0.100 | 22.500 | 1.000 | 1.000 | 1.000 | 1.000 | 1.879 | 42.278 |
| Shandong | Zibo | 2 | 131 | 822 | 160 | 538 | 9 | 20 | 0.444 | 0.100 | 22.500 | 1.000 | 1.000 | 1.000 | 1.000 | 1.879 | 42.278 |
| Shandong | Tai'an | 2 | 131 | 822 | 105 | 527 | 9 | 15 | 0.444 | 0.133 | 16.875 | 0.727 | 1.000 | 0.727 | 1.375 | 1.879 | 43.599 |
| Shandong | Zibo | 2 | 131 | 822 | 160 | 535 | 9 | 20 | 0.444 | 0.100 | 22.500 | 0.667 | 1.000 | 0.667 | 1.500 | 1.879 | 63.416 |
| Shandong | Liaocheng | 2 | 131 | 822 | 159 | 515 | 9 | 20 | 0.444 | 0.100 | 22.500 | 0.545 | 1.000 | 0.545 | 1.833 | 1.879 | 77.509 |
| Shandong | Zaozhuang | 2 | 131 | 822 | 82 | N/A | 9 | 11 | 0.444 | 0.273 |  |  |  |  |  |  |  |
| Qinghai | Xining | 2 | 130 | 18 | 78 | N/A | 1 | 12 | 1.000 | 0.167 |  |  |  |  |  |  |  |
| Qinghai | Xining | 2 | 130 | 18 | 78 | N/A | 1 | 12 | 1.000 | 0.167 |  |  |  |  |  |  |  |
| Qinghai | Xining | 2 | 129 | 18 | 78 | 505 | 1 | 6 | 1.000 | 1.000 | 1.000 | 1.000 | 0.833 | 0.833 | 1.200 | 0.219 | 0.263 |
| Qinghai | Xining | 2 | 129 | 18 | 78 | 503 | 1 | 6 | 1.000 | 1.000 | 1.000 | 1.000 | 0.833 | 0.833 | 1.200 | 0.219 | 0.263 |
| Qinghai | Xining | 2 | 129 | 18 | 78 | 502 | 1 | 6 | 1.000 | 1.000 | 1.000 | 1.000 | 0.833 | 0.833 | 1.200 | 0.219 | 0.263 |
| Qinghai | Xining | 2 | 129 | 18 | 78 | 500 | 1 | 6 | 1.000 | 1.000 | 1.000 | 1.000 | 0.833 | 0.833 | 1.200 | 0.219 | 0.263 |
| Qinghai | Xining | 2 | 129 | 18 | 78 | 499 | 1 | 6 | 1.000 | 1.000 | 1.000 | 1.000 | 0.833 | 0.833 | 1.200 | 0.219 | 0.263 |
| Qinghai | Xining | 2 | 129 | 18 | 78 | N/A | 1 | 6 | 1.000 | 1.000 |  |  |  |  |  |  |  |
| Qinghai | Xining | 2 | 128 | 18 | 78 | 504 | 1 | 10 | 1.000 | 0.200 | 5.000 | 1.000 | 1.000 | 1.000 | 1.000 | 0.219 | 1.094 |
| Qinghai | Xining | 2 | 128 | 18 | 78 | 501 | 1 | 10 | 1.000 | 0.200 | 5.000 | 1.000 | 1.000 | 1.000 | 1.000 | 0.219 | 1.094 |
| Qinghai | Yushu | 1 | 127 | 18 |  | 509 | 1 | 4 | 1.000 | 0.500 | 2.000 | 1.000 | 1.000 | 1.000 | 1.000 | 0.219 | 0.438 |
| Qinghai | Yushu | 1 | 127 | 18 |  | 508 | 1 | 4 | 1.000 | 0.500 | 2.000 | 1.000 | 1.000 | 1.000 | 1.000 | 0.219 | 0.438 |
| Qinghai | Hainan | 1 | 127 | 18 | 0 | 494 | 1 | 5 | 1.000 | 0.400 | 2.500 | 1.000 | 1.000 | 1.000 | 1.000 | 0.219 | 0.547 |
| Qinghai | Hainan | 1 | 127 | 18 | 0 | 493 | 1 | 5 | 1.000 | 0.400 | 2.500 | 1.000 | 1.000 | 1.000 | 1.000 | 0.219 | 0.547 |
| Qinghai | Haixi | 1 | 127 | 18 | 0 | 496 | 1 | 6 | 1.000 | 0.333 | 3.000 | 1.000 | 1.000 | 1.000 | 1.000 | 0.219 | 0.656 |
| Qinghai | Haidong | 1 | 127 | 18 | 57 | 490 | 1 | 7 | 1.000 | 0.286 | 3.500 | 1.000 | 1.000 | 1.000 | 1.000 | 0.219 | 0.766 |
| Qinghai | Haidong | 1 | 127 | 18 | 57 | 489 | 1 | 7 | 1.000 | 0.286 | 3.500 | 1.000 | 1.000 | 1.000 | 1.000 | 0.219 | 0.766 |
| Qinghai | Haibei | 1 | 127 | 18 | 0 | 486 | 1 | 4 | 1.000 | 0.500 | 2.000 | 0.500 | 1.000 | 0.500 | 2.000 | 0.219 | 0.875 |
| Qinghai | Haixi | 1 | 127 | 18 | 0 | 495 | 1 | 6 | 1.000 | 0.333 | 3.000 | 0.250 | 1.000 | 0.250 | 4.000 | 0.219 | 2.626 |
| Qinghai | Haibei | 1 | 127 | 18 | 0 | N/A | 1 | 4 | 1.000 | 0.500 |  |  |  |  |  |  |  |
| Qinghai | Haixi | 1 | 126 | 18 | 0 | N/A | 1 | 1 | 1.000 | 1.000 |  |  |  |  |  |  |  |
| Qinghai | Yushu | 1 | 125 | 18 |  | 507 | 1 | 3 | 1.000 | 0.667 | 1.500 | 1.000 | 1.000 | 1.000 | 1.000 | 0.219 | 0.328 |
| Qinghai | Yushu | 1 | 125 | 18 |  | 506 | 1 | 3 | 1.000 | 0.667 | 1.500 | 1.000 | 1.000 | 1.000 | 1.000 | 0.219 | 0.328 |
| Qinghai | Hainan | 1 | 125 | 18 | 0 | 492 | 1 | 4 | 1.000 | 0.500 | 2.000 | 1.000 | 1.000 | 1.000 | 1.000 | 0.219 | 0.438 |
| Qinghai | Hainan | 1 | 125 | 18 | 0 | 491 | 1 | 4 | 1.000 | 0.500 | 2.000 | 1.000 | 1.000 | 1.000 | 1.000 | 0.219 | 0.438 |
| Qinghai | Haixi | 1 | 125 | 18 | 0 | 498 | 1 | 5 | 1.000 | 0.400 | 2.500 | 1.000 | 1.000 | 1.000 | 1.000 | 0.219 | 0.547 |
| Qinghai | Haixi | 1 | 125 | 18 | 0 | 497 | 1 | 5 | 1.000 | 0.400 | 2.500 | 1.000 | 1.000 | 1.000 | 1.000 | 0.219 | 0.547 |
| Qinghai | Haidong | 1 | 125 | 18 | 57 | 488 | 1 | 6 | 1.000 | 0.333 | 3.000 | 1.000 | 1.000 | 1.000 | 1.000 | 0.219 | 0.656 |
| Qinghai | Haidong | 1 | 125 | 18 | 57 | 487 | 1 | 6 | 1.000 | 0.333 | 3.000 | 1.000 | 1.000 | 1.000 | 1.000 | 0.219 | 0.656 |
| Qinghai | Haibei | 1 | 125 | 18 | 0 | 485 | 1 | 4 | 1.000 | 0.500 | 2.000 | 1.000 | 0.500 | 0.500 | 2.000 | 0.219 | 0.875 |
| Qinghai | Haibei | 1 | 125 | 18 | 0 | N/A | 1 | 4 | 1.000 | 0.500 |  |  |  |  |  |  |  |
| Ningxia | Yinchuan | 2 | 124 | 75 | 86 | 475 | 1 | 12 | 1.000 | 0.167 | 6.000 | 1.000 | 1.000 | 1.000 | 1.000 | 0.303 | 1.820 |
| Ningxia | Yinchuan | 2 | 124 | 75 | 86 | 479 | 1 | 12 | 1.000 | 0.167 | 6.000 | 0.500 | 1.000 | 0.500 | 2.000 | 0.303 | 3.640 |
| Ningxia | Yinchuan | 2 | 123 | 75 | 86 | 477 | 1 | 5 | 1.000 | 0.400 | 2.500 | 1.000 | 1.000 | 1.000 | 1.000 | 0.303 | 0.758 |
| Ningxia | Yinchuan | 2 | 123 | 75 | 86 | 476 | 1 | 5 | 1.000 | 0.400 | 2.500 | 0.789 | 1.000 | 0.789 | 1.267 | 0.303 | 0.960 |
| Ningxia | Yinchuan | 2 | 122 | 75 | 86 | 478 | 1 | 12 | 1.000 | 0.167 | 6.000 | 0.833 | 1.000 | 0.833 | 1.200 | 0.303 | 2.184 |
| Ningxia | Yinchuan | 2 | 122 | 75 | 86 | 480 | 1 | 12 | 1.000 | 0.167 | 6.000 | 0.625 | 1.000 | 0.625 | 1.600 | 0.303 | 2.912 |
| Ningxia | Guyuan | 1 | 121 | 75 | 13 | 467 | 4 | 3 | 1.000 | 0.667 | 1.500 | 1.000 | 1.000 | 1.000 | 1.000 | 0.303 | 0.455 |
| Ningxia | Guyuan | 1 | 121 | 75 | 13 | 464 | 4 | 3 | 1.000 | 0.667 | 1.500 | 1.000 | 1.000 | 1.000 | 1.000 | 0.303 | 0.455 |
| Ningxia | Zhongwei | 1 | 121 | 75 | 32 | 484 | 4 | 4 | 1.000 | 0.500 | 2.000 | 0.667 | 1.000 | 0.667 | 1.500 | 0.303 | 0.910 |
| Ningxia | Zhongwei | 1 | 121 | 75 | 32 | 481 | 4 | 4 | 1.000 | 0.500 | 2.000 | 0.444 | 1.000 | 0.444 | 2.250 | 0.303 | 1.365 |
| Ningxia | Shizuishan | 1 | 121 | 75 | 36 | 471 | 4 | 8 | 1.000 | 0.250 | 4.000 | 0.800 | 1.000 | 0.800 | 1.250 | 0.303 | 1.517 |
| Ningxia | Shizuishan | 1 | 121 | 75 | 36 | 470 | 4 | 8 | 1.000 | 0.250 | 4.000 | 0.750 | 1.000 | 0.750 | 1.333 | 0.303 | 1.618 |
| Ningxia | Wuzhong | 1 | 121 | 75 | 50 | 474 | 4 | 14 | 1.000 | 0.143 | 7.000 | 1.000 | 0.500 | 0.500 | 2.000 | 0.303 | 4.246 |
| Ningxia | Wuzhong | 1 | 121 | 75 | 50 | N/A | 4 | 14 | 1.000 | 0.143 |  |  |  |  |  |  |  |
| Ningxia | Guyuan | 1 | 120 | 75 | 13 | 466 | 4 | 4 | 1.000 | 0.500 | 2.000 | 1.000 | 1.000 | 1.000 | 1.000 | 0.303 | 0.607 |
| Ningxia | Guyuan | 1 | 120 | 75 | 13 | 465 | 4 | 4 | 1.000 | 0.500 | 2.000 | 1.000 | 1.000 | 1.000 | 1.000 | 0.303 | 0.607 |
| Ningxia | Zhongwei | 1 | 120 | 75 | 32 | 483 | 4 | 5 | 1.000 | 0.400 | 2.500 | 1.000 | 1.000 | 1.000 | 1.000 | 0.303 | 0.758 |
| Ningxia | Zhongwei | 1 | 120 | 75 | 32 | 482 | 4 | 5 | 1.000 | 0.400 | 2.500 | 1.000 | 1.000 | 1.000 | 1.000 | 0.303 | 0.758 |
| Ningxia | Shizuishan | 1 | 120 | 75 | 36 | 469 | 4 | 6 | 1.000 | 0.333 | 3.000 | 1.000 | 1.000 | 1.000 | 1.000 | 0.303 | 0.910 |
| Ningxia | Shizuishan | 1 | 120 | 75 | 36 | 468 | 4 | 6 | 1.000 | 0.333 | 3.000 | 1.000 | 1.000 | 1.000 | 1.000 | 0.303 | 0.910 |
| Ningxia | Wuzhong | 1 | 120 | 75 | 50 | 473 | 4 | 7 | 1.000 | 0.286 | 3.500 | 1.000 | 1.000 | 1.000 | 1.000 | 0.303 | 1.062 |
| Ningxia | Wuzhong | 1 | 120 | 75 | 50 | 472 | 4 | 7 | 1.000 | 0.286 | 3.500 | 1.000 | 1.000 | 1.000 | 1.000 | 0.303 | 1.062 |
| Inner Mongolia | Baotou | 2 | 119 | 260 | 105 | 443 | 6 | 13 | 0.667 | 0.154 | 9.750 | 0.500 | 1.000 | 0.500 | 2.000 | 1.222 | 23.835 |
| Inner Mongolia | Tongliao | 2 | 119 | 260 | 85 | 456 | 6 | 11 | 0.667 | 0.182 | 8.250 | 0.667 | 0.500 | 0.333 | 3.000 | 1.222 | 30.252 |
| Inner Mongolia | Baotou | 2 | 119 | 260 | 105 | 444 | 6 | 13 | 0.667 | 0.154 | 9.750 | 0.333 | 1.000 | 0.333 | 3.000 | 1.222 | 35.753 |
| Inner Mongolia | Hohhot | 2 | 119 | 260 | 108 | 452 | 6 | 20 | 0.667 | 0.100 | 15.000 | 1.000 | 0.500 | 0.500 | 2.000 | 1.222 | 36.669 |
| Inner Mongolia | Chifeng | 2 | 119 | 260 | 107 | 451 | 6 | 24 | 0.667 | 0.083 | 18.000 | 0.500 | 1.000 | 0.500 | 2.000 | 1.222 | 44.003 |
| Inner Mongolia | Chifeng | 2 | 119 | 260 | 107 | 449 | 6 | 24 | 0.667 | 0.083 | 18.000 | 0.500 | 1.000 | 0.500 | 2.000 | 1.222 | 44.003 |
| Inner Mongolia | Hohhot | 2 | 119 | 260 | 108 | N/A | 6 | 20 | 0.667 | 0.100 |  |  |  |  |  |  |  |
| Inner Mongolia | Tongliao | 2 | 119 | 260 | 85 | N/A | 6 | 11 | 0.667 | 0.182 |  |  |  |  |  |  |  |
| Inner Mongolia | Tongliao | 2 | 118 | 260 | 85 | 457 | 6 | 3 | 0.667 | 0.667 | 2.250 | 0.944 | 1.000 | 0.944 | 1.059 | 1.222 | 2.912 |
| Inner Mongolia | Tongliao | 2 | 118 | 260 | 85 | 459 | 6 | 3 | 0.667 | 0.667 | 2.250 | 0.813 | 1.000 | 0.813 | 1.231 | 1.222 | 3.385 |
| Inner Mongolia | Baotou | 2 | 118 | 260 | 105 | 446 | 6 | 4 | 0.667 | 0.500 | 3.000 | 1.000 | 1.000 | 1.000 | 1.000 | 1.222 | 3.667 |
| Inner Mongolia | Baotou | 2 | 118 | 260 | 105 | 447 | 6 | 4 | 0.667 | 0.500 | 3.000 | 0.857 | 1.000 | 0.857 | 1.167 | 1.222 | 4.278 |
| Inner Mongolia | Hohhot | 2 | 118 | 260 | 108 | 455 | 6 | 6 | 0.667 | 0.333 | 4.500 | 1.000 | 1.000 | 1.000 | 1.000 | 1.222 | 5.500 |
| Inner Mongolia | Hohhot | 2 | 118 | 260 | 108 | 454 | 6 | 6 | 0.667 | 0.333 | 4.500 | 1.000 | 1.000 | 1.000 | 1.000 | 1.222 | 5.500 |
| Inner Mongolia | Chifeng | 2 | 118 | 260 | 107 | 448 | 6 | 4 | 0.667 | 0.500 | 3.000 | 1.000 | 0.500 | 0.500 | 2.000 | 1.222 | 7.334 |
| Inner Mongolia | Chifeng | 2 | 118 | 260 | 107 | N/A | 6 | 4 | 0.667 | 0.500 |  |  |  |  |  |  |  |
| Inner Mongolia | Tongliao | 2 | 117 | 260 | 85 | 460 | 6 | 8 | 0.667 | 0.250 | 6.000 | 0.875 | 1.000 | 0.875 | 1.143 | 1.222 | 8.382 |
| Inner Mongolia | Tongliao | 2 | 117 | 260 | 85 | 458 | 6 | 8 | 0.667 | 0.250 | 6.000 | 0.778 | 1.000 | 0.778 | 1.286 | 1.222 | 9.429 |
| Inner Mongolia | Chifeng | 2 | 117 | 260 | 107 | 450 | 6 | 12 | 0.667 | 0.167 | 9.000 | 0.667 | 0.500 | 0.333 | 3.000 | 1.222 | 33.003 |
| Inner Mongolia | Hohhot | 2 | 117 | 260 | 108 | 453 | 6 | 15 | 0.667 | 0.133 | 11.250 | 0.750 | 0.500 | 0.375 | 2.667 | 1.222 | 36.669 |
| Inner Mongolia | Baotou | 2 | 117 | 260 | 105 | 445 | 6 | 16 | 0.667 | 0.125 | 12.000 | 0.222 | 0.500 | 0.111 | 9.000 | 1.222 | 132.010 |
| Inner Mongolia | Baotou | 2 | 117 | 260 | 105 | N/A | 6 | 16 | 0.667 | 0.125 |  |  |  |  |  |  |  |
| Inner Mongolia | Chifeng | 2 | 117 | 260 | 107 | N/A | 6 | 12 | 0.667 | 0.167 |  |  |  |  |  |  |  |
| Inner Mongolia | Hohhot | 2 | 117 | 260 | 108 | N/A | 6 | 15 | 0.667 | 0.133 |  |  |  |  |  |  |  |
| Inner Mongolia | Wuhai | 1 | 116 | 260 | 28 | 461 | 3 | 6 | 0.667 | 0.333 | 4.500 | 1.000 | 1.000 | 1.000 | 1.000 | 1.222 | 5.500 |
| Inner Mongolia | Bayannur | 1 | 116 | 260 | 56 | 440 | 3 | 10 | 0.667 | 0.200 | 7.500 | 1.000 | 1.000 | 1.000 | 1.000 | 1.222 | 9.167 |
| Inner Mongolia | Bayannur | 1 | 116 | 260 | 56 | 442 | 3 | 10 | 0.667 | 0.200 | 7.500 | 0.571 | 1.000 | 0.571 | 1.750 | 1.222 | 16.043 |
| Inner Mongolia | Wuhai | 1 | 116 | 260 | 28 | N/A | 3 | 6 | 0.667 | 0.333 |  |  |  |  |  |  |  |
| Inner Mongolia | Wuhai | 1 | 115 | 260 | 28 | 463 | 3 | 1 | 0.667 | 1.000 | 1.500 | 1.000 | 1.000 | 1.000 | 1.000 | 1.222 | 1.833 |
| Inner Mongolia | Bayannur | 1 | 115 | 260 | 56 | 439 | 3 | 3 | 0.667 | 0.667 | 2.250 | 0.857 | 1.000 | 0.857 | 1.167 | 1.222 | 3.209 |
| Inner Mongolia | Bayannur | 1 | 115 | 260 | 56 | 438 | 3 | 3 | 0.667 | 0.667 | 2.250 | 0.800 | 1.000 | 0.800 | 1.250 | 1.222 | 3.438 |
| Inner Mongolia | Wuhai | 1 | 114 | 260 | 28 | 462 | 3 | 4 | 0.667 | 0.500 | 3.000 | 0.500 | 0.500 | 0.250 | 4.000 | 1.222 | 14.668 |
| Inner Mongolia | Bayannur | 1 | 114 | 260 | 56 | 441 | 3 | 7 | 0.667 | 0.286 | 5.250 | 0.286 | 1.000 | 0.286 | 3.500 | 1.222 | 22.460 |
| Inner Mongolia | Bayannur | 1 | 114 | 260 | 56 | N/A | 3 | 7 | 0.667 | 0.286 |  |  |  |  |  |  |  |
| Inner Mongolia | Wuhai | 1 | 114 | 260 | 28 | N/A | 3 | 4 | 0.667 | 0.500 |  |  |  |  |  |  |  |
| Liaoning | Dalian | 3 | 113 | 261 | 178 | 423 | 2 | 19 | 0.500 | 0.105 | 19.000 | 1.000 | 1.000 | 1.000 | 1.000 | 1.334 | 25.344 |
| Liaoning | Dalian | 3 | 113 | 261 | 178 | 422 | 2 | 19 | 0.500 | 0.105 | 19.000 | 1.000 | 1.000 | 1.000 | 1.000 | 1.334 | 25.344 |
| Liaoning | Dalian | 3 | 112 | 261 | 178 | 425 | 2 | 8 | 0.500 | 0.250 | 8.000 | 1.000 | 1.000 | 1.000 | 1.000 | 1.334 | 10.671 |
| Liaoning | Dalian | 3 | 112 | 261 | 178 | 420 | 2 | 8 | 0.500 | 0.250 | 8.000 | 0.875 | 1.000 | 0.875 | 1.143 | 1.334 | 12.195 |
| Liaoning | Dalian | 3 | 111 | 261 | 178 | 424 | 2 | 24 | 0.500 | 0.083 | 24.000 | 1.000 | 1.000 | 1.000 | 1.000 | 1.334 | 32.013 |
| Liaoning | Dalian | 3 | 111 | 261 | 178 | 421 | 2 | 24 | 0.500 | 0.083 | 24.000 | 1.000 | 1.000 | 1.000 | 1.000 | 1.334 | 32.013 |
| Liaoning | Fuxin | 2 | 110 | 261 | 56 | 429 | 11 | 10 | 0.273 | 0.200 | 18.333 | 0.833 | 1.000 | 0.833 | 1.200 | 1.334 | 29.345 |
| Liaoning | Chaoyang | 2 | 110 | 261 | 96 | 417 | 11 | 14 | 0.273 | 0.143 | 25.667 | 0.900 | 1.000 | 0.900 | 1.111 | 1.334 | 38.040 |
| Liaoning | Chaoyang | 2 | 110 | 261 | 96 | 415 | 11 | 14 | 0.273 | 0.143 | 25.667 | 0.833 | 1.000 | 0.833 | 1.200 | 1.334 | 41.084 |
| Liaoning | Fuxin | 2 | 110 | 261 | 56 | 431 | 11 | 10 | 0.273 | 0.200 | 18.333 | 0.400 | 1.000 | 0.400 | 2.500 | 1.334 | 61.136 |
| Liaoning | Yingkou | 2 | 110 | 261 | 139 | 436 | 11 | 30 | 0.273 | 0.067 | 55.000 | 1.000 | 1.000 | 1.000 | 1.000 | 1.334 | 73.363 |
| Liaoning | Yingkou | 2 | 110 | 261 | 139 | 435 | 11 | 30 | 0.273 | 0.067 | 55.000 | 1.000 | 1.000 | 1.000 | 1.000 | 1.334 | 73.363 |
| Liaoning | #N/A | 2 | 110 | 261 |  | N/A | 11 | 8 | 0.273 | 0.250 |  |  |  |  |  |  |  |
| Liaoning | #N/A | 2 | 110 | 261 |  | N/A | 11 | 8 | 0.273 | 0.250 |  |  |  |  |  |  |  |
| Liaoning | #N/A | 2 | 110 | 261 |  | N/A | 11 | 12 | 0.273 | 0.167 |  |  |  |  |  |  |  |
| Liaoning | #N/A | 2 | 110 | 261 |  | N/A | 11 | 12 | 0.273 | 0.167 |  |  |  |  |  |  |  |
| Liaoning | Fuxin | 2 | 109 | 261 | 56 | 430 | 11 | 3 | 0.273 | 0.667 | 5.500 | 1.000 | 1.000 | 1.000 | 1.000 | 1.334 | 7.336 |
| Liaoning | Fuxin | 2 | 109 | 261 | 56 | 426 | 11 | 3 | 0.273 | 0.667 | 5.500 | 1.000 | 1.000 | 1.000 | 1.000 | 1.334 | 7.336 |
| Liaoning | Chaoyang | 2 | 109 | 261 | 96 | 416 | 11 | 4 | 0.273 | 0.500 | 7.333 | 1.000 | 1.000 | 1.000 | 1.000 | 1.334 | 9.782 |
| Liaoning | Chaoyang | 2 | 109 | 261 | 96 | 414 | 11 | 4 | 0.273 | 0.500 | 7.333 | 1.000 | 1.000 | 1.000 | 1.000 | 1.334 | 9.782 |
| Liaoning | Yingkou | 2 | 109 | 261 | 139 | 433 | 11 | 5 | 0.273 | 0.400 | 9.167 | 1.000 | 1.000 | 1.000 | 1.000 | 1.334 | 12.227 |
| Liaoning | Yingkou | 2 | 109 | 261 | 139 | 437 | 11 | 5 | 0.273 | 0.400 | 9.167 | 0.800 | 1.000 | 0.800 | 1.250 | 1.334 | 15.284 |
| Liaoning | #N/A | 2 | 109 | 261 |  | N/A | 11 | 2 | 0.273 | 1.000 |  |  |  |  |  |  |  |
| Liaoning | #N/A | 2 | 109 | 261 |  | N/A | 11 | 2 | 0.273 | 1.000 |  |  |  |  |  |  |  |
| Liaoning | #N/A | 2 | 109 | 261 |  | N/A | 11 | 3 | 0.273 | 0.667 |  |  |  |  |  |  |  |
| Liaoning | #N/A | 2 | 109 | 261 |  | N/A | 11 | 3 | 0.273 | 0.667 |  |  |  |  |  |  |  |
| Liaoning | Fuxin | 2 | 108 | 261 | 56 | 428 | 11 | 6 | 0.273 | 0.333 | 11.000 | 1.000 | 1.000 | 1.000 | 1.000 | 1.334 | 14.673 |
| Liaoning | Fuxin | 2 | 108 | 261 | 56 | 427 | 11 | 6 | 0.273 | 0.333 | 11.000 | 1.000 | 1.000 | 1.000 | 1.000 | 1.334 | 14.673 |
| Liaoning | Chaoyang | 2 | 108 | 261 | 96 | 419 | 11 | 11 | 0.273 | 0.182 | 20.167 | 1.000 | 1.000 | 1.000 | 1.000 | 1.334 | 26.900 |
| Liaoning | Chaoyang | 2 | 108 | 261 | 96 | 418 | 11 | 11 | 0.273 | 0.182 | 20.167 | 1.000 | 1.000 | 1.000 | 1.000 | 1.334 | 26.900 |
| Liaoning | Yingkou | 2 | 108 | 261 | 139 | 434 | 11 | 25 | 0.273 | 0.080 | 45.833 | 0.933 | 1.000 | 0.933 | 1.071 | 1.334 | 65.503 |
| Liaoning | Yingkou | 2 | 108 | 261 | 139 | 432 | 11 | 25 | 0.273 | 0.080 | 45.833 | 0.714 | 1.000 | 0.714 | 1.400 | 1.334 | 85.591 |
| Liaoning | #N/A | 2 | 108 | 261 |  | N/A | 11 | 5 | 0.273 | 0.400 |  |  |  |  |  |  |  |
| Liaoning | #N/A | 2 | 108 | 261 |  | N/A | 11 | 5 | 0.273 | 0.400 |  |  |  |  |  |  |  |
| Liaoning | #N/A | 2 | 108 | 261 |  | N/A | 11 | 9 | 0.273 | 0.222 |  |  |  |  |  |  |  |
| Liaoning | #N/A | 2 | 108 | 261 |  | N/A | 11 | 9 | 0.273 | 0.222 |  |  |  |  |  |  |  |
| Jiangxi | Jiujiang | 2 | 107 | 932 | 62 | 393 | 6 | 11 | 0.333 | 0.182 | 16.500 | 1.000 | 1.000 | 1.000 | 1.000 | 0.749 | 12.350 |
| Jiangxi | Jiujiang | 2 | 107 | 932 | 62 | 388 | 6 | 11 | 0.333 | 0.182 | 16.500 | 0.947 | 1.000 | 0.947 | 1.056 | 0.749 | 13.036 |
| Jiangxi | Nanchang | 2 | 107 | 932 | 122 | 397 | 6 | 23 | 0.333 | 0.087 | 34.500 | 1.000 | 1.000 | 1.000 | 1.000 | 0.749 | 25.823 |
| Jiangxi | Nanchang | 2 | 107 | 932 | 122 | 396 | 6 | 23 | 0.333 | 0.087 | 34.500 | 0.500 | 1.000 | 0.500 | 2.000 | 0.749 | 51.647 |
| Jiangxi | #N/A | 2 | 107 | 932 |  | N/A | 6 | 8 | 0.333 | 0.250 |  |  |  |  |  |  |  |
| Jiangxi | #N/A | 2 | 107 | 932 |  | N/A | 6 | 8 | 0.333 | 0.250 |  |  |  |  |  |  |  |
| Jiangxi | Jiujiang | 2 | 106 | 932 | 62 | 390 | 6 | 3 | 0.333 | 0.667 | 4.500 | 1.000 | 1.000 | 1.000 | 1.000 | 0.749 | 3.368 |
| Jiangxi | Jiujiang | 2 | 106 | 932 | 62 | 391 | 6 | 3 | 0.333 | 0.667 | 4.500 | 1.000 | 1.000 | 1.000 | 1.000 | 0.749 | 3.368 |
| Jiangxi | Nanchang | 2 | 106 | 932 | 122 | 395 | 6 | 14 | 0.333 | 0.143 | 21.000 | 0.857 | 1.000 | 0.857 | 1.167 | 0.749 | 18.338 |
| Jiangxi | Nanchang | 2 | 106 | 932 | 122 | 394 | 6 | 14 | 0.333 | 0.143 | 21.000 | 0.667 | 1.000 | 0.667 | 1.500 | 0.749 | 23.578 |
| Jiangxi | #N/A | 2 | 106 | 932 |  | N/A | 6 | 2 | 0.333 | 1.000 |  |  |  |  |  |  |  |
| Jiangxi | #N/A | 2 | 106 | 932 |  | N/A | 6 | 2 | 0.333 | 1.000 |  |  |  |  |  |  |  |
| Jiangxi | Jiujiang | 2 | 105 | 932 | 62 | 392 | 6 | 9 | 0.333 | 0.222 | 13.500 | 1.000 | 1.000 | 1.000 | 1.000 | 0.749 | 10.105 |
| Jiangxi | Jiujiang | 2 | 105 | 932 | 62 | 389 | 6 | 9 | 0.333 | 0.222 | 13.500 | 0.636 | 1.000 | 0.636 | 1.571 | 0.749 | 15.879 |
| Jiangxi | Nanchang | 2 | 105 | 932 | 122 | 399 | 6 | 16 | 0.333 | 0.125 | 24.000 | 1.000 | 1.000 | 1.000 | 1.000 | 0.749 | 17.964 |
| Jiangxi | Nanchang | 2 | 105 | 932 | 122 | 398 | 6 | 16 | 0.333 | 0.125 | 24.000 | 0.667 | 1.000 | 0.667 | 1.500 | 0.749 | 26.946 |
| Jiangxi | #N/A | 2 | 105 | 932 |  | N/A | 6 | 7 | 0.333 | 0.286 |  |  |  |  |  |  |  |
| Jiangxi | #N/A | 2 | 105 | 932 |  | N/A | 6 | 7 | 0.333 | 0.286 |  |  |  |  |  |  |  |
| Jiangxi | Xinyu | 1 | 104 | 932 | 16 | 407 | 4 | 6 | 0.750 | 0.333 | 4.000 | 1.000 | 1.000 | 1.000 | 1.000 | 0.749 | 2.994 |
| Jiangxi | Xinyu | 1 | 104 | 932 | 16 | 406 | 4 | 6 | 0.750 | 0.333 | 4.000 | 1.000 | 1.000 | 1.000 | 1.000 | 0.749 | 2.994 |
| Jiangxi | Pingxiang | 1 | 104 | 932 | 33 | 404 | 4 | 10 | 0.750 | 0.200 | 6.667 | 1.000 | 1.000 | 1.000 | 1.000 | 0.749 | 4.990 |
| Jiangxi | Pingxiang | 1 | 104 | 932 | 33 | 402 | 4 | 10 | 0.750 | 0.200 | 6.667 | 1.000 | 1.000 | 1.000 | 1.000 | 0.749 | 4.990 |
| Jiangxi | Yingtan | 1 | 104 | 932 | 33 | 413 | 4 | 10 | 0.750 | 0.200 | 6.667 | 1.000 | 1.000 | 1.000 | 1.000 | 0.749 | 4.990 |
| Jiangxi | Yingtan | 1 | 104 | 932 | 33 | 411 | 4 | 10 | 0.750 | 0.200 | 6.667 | 1.000 | 1.000 | 1.000 | 1.000 | 0.749 | 4.990 |
| Jiangxi | Pingxiang | 1 | 103 | 932 | 33 | 403 | 4 | 2 | 0.750 | 1.000 | 1.333 | 1.000 | 1.000 | 1.000 | 1.000 | 0.749 | 0.998 |
| Jiangxi | Pingxiang | 1 | 103 | 932 | 33 | 401 | 4 | 2 | 0.750 | 1.000 | 1.333 | 1.000 | 1.000 | 1.000 | 1.000 | 0.749 | 0.998 |
| Jiangxi | Xinyu | 1 | 103 | 932 | 16 | 410 | 4 | 2 | 0.750 | 1.000 | 1.333 | 1.000 | 1.000 | 1.000 | 1.000 | 0.749 | 0.998 |
| Jiangxi | Xinyu | 1 | 103 | 932 | 16 | 409 | 4 | 2 | 0.750 | 1.000 | 1.333 | 1.000 | 1.000 | 1.000 | 1.000 | 0.749 | 0.998 |
| Jiangxi | Yingtan | 1 | 103 | 932 | 33 | 412 | 4 | 1 | 0.750 | 1.000 | 1.333 | 1.000 | 1.000 | 1.000 | 1.000 | 0.749 | 0.998 |
| Jiangxi | Pingxiang | 1 | 102 | 932 | 33 | 400 | 4 | 6 | 0.750 | 0.333 | 4.000 | 1.000 | 1.000 | 1.000 | 1.000 | 0.749 | 2.994 |
| Jiangxi | Xinyu | 1 | 102 | 932 | 16 | 408 | 4 | 6 | 0.750 | 0.333 | 4.000 | 1.000 | 1.000 | 1.000 | 1.000 | 0.749 | 2.994 |
| Jiangxi | Xinyu | 1 | 102 | 932 | 16 | 405 | 4 | 6 | 0.750 | 0.333 | 4.000 | 1.000 | 1.000 | 1.000 | 1.000 | 0.749 | 2.994 |
| Jiangxi | Pingxiang | 1 | 102 | 932 | 33 | N/A | 4 | 6 | 0.750 | 0.333 |  |  |  |  |  |  |  |
| Jiangxi | Yingtan | 1 | 102 | 932 | 33 | N/A | 4 | 6 | 0.750 | 0.333 |  |  |  |  |  |  |  |
| Jiangxi | Yingtan | 1 | 102 | 932 | 33 | N/A | 4 | 6 | 0.750 | 0.333 |  |  |  |  |  |  |  |
| Jiangsu | Wuxi | 3 | 101 | 664 | 185 | 375 | 5 | 20 | 0.400 | 0.100 | 25.000 | 1.000 | 1.000 | 1.000 | 1.000 | 1.027 | 25.687 |
| Jiangsu | Wuxi | 3 | 101 | 664 | 185 | 370 | 5 | 20 | 0.400 | 0.100 | 25.000 | 1.000 | 1.000 | 1.000 | 1.000 | 1.027 | 25.687 |
| Jiangsu | Nanjing | 3 | 101 | 664 | 222 | 362 | 5 | 29 | 0.400 | 0.069 | 36.250 | 1.000 | 1.000 | 1.000 | 1.000 | 1.027 | 37.247 |
| Jiangsu | Nanjing | 3 | 101 | 664 | 222 | 363 | 5 | 29 | 0.400 | 0.069 | 36.250 | 0.857 | 1.000 | 0.857 | 1.167 | 1.027 | 43.454 |
| Jiangsu | Wuxi | 3 | 100 | 664 | 185 | 372 | 5 | 8 | 0.400 | 0.250 | 10.000 | 1.000 | 1.000 | 1.000 | 1.000 | 1.027 | 10.275 |
| Jiangsu | Wuxi | 3 | 100 | 664 | 185 | 371 | 5 | 8 | 0.400 | 0.250 | 10.000 | 1.000 | 1.000 | 1.000 | 1.000 | 1.027 | 10.275 |
| Jiangsu | Nanjing | 3 | 100 | 664 | 222 | 358 | 5 | 19 | 0.400 | 0.105 | 23.750 | 1.000 | 1.000 | 1.000 | 1.000 | 1.027 | 24.403 |
| Jiangsu | Nanjing | 3 | 100 | 664 | 222 | 360 | 5 | 19 | 0.400 | 0.105 | 23.750 | 0.990 | 1.000 | 0.990 | 1.010 | 1.027 | 24.655 |
| Jiangsu | Wuxi | 3 | 99 | 664 | 185 | 374 | 5 | 30 | 0.400 | 0.067 | 37.500 | 1.000 | 1.000 | 1.000 | 1.000 | 1.027 | 38.531 |
| Jiangsu | Wuxi | 3 | 99 | 664 | 185 | 373 | 5 | 30 | 0.400 | 0.067 | 37.500 | 1.000 | 1.000 | 1.000 | 1.000 | 1.027 | 38.531 |
| Jiangsu | Nanjing | 3 | 99 | 664 | 222 | 361 | 5 | 32 | 0.400 | 0.063 | 40.000 | 1.000 | 1.000 | 1.000 | 1.000 | 1.027 | 41.100 |
| Jiangsu | Nanjing | 3 | 99 | 664 | 222 | 359 | 5 | 32 | 0.400 | 0.063 | 40.000 | 1.000 | 1.000 | 1.000 | 1.000 | 1.027 | 41.100 |
| Jiangsu | Lianyungang | 2 | 98 | 664 | 89 | 355 | 8 | 15 | 0.500 | 0.133 | 15.000 | 1.000 | 1.000 | 1.000 | 1.000 | 1.027 | 15.412 |
| Jiangsu | Lianyungang | 2 | 98 | 664 | 89 | 352 | 8 | 15 | 0.500 | 0.133 | 15.000 | 1.000 | 1.000 | 1.000 | 1.000 | 1.027 | 15.412 |
| Jiangsu | Suqian | 2 | 98 | 664 | 229 | 381 | 8 | 20 | 0.500 | 0.100 | 20.000 | 1.000 | 1.000 | 1.000 | 1.000 | 1.027 | 20.550 |
| Jiangsu | Suqian | 2 | 98 | 664 | 229 | 379 | 8 | 20 | 0.500 | 0.100 | 20.000 | 1.000 | 1.000 | 1.000 | 1.000 | 1.027 | 20.550 |
| Jiangsu | Yancheng | 2 | 98 | 664 | 164 | 384 | 8 | 22 | 0.500 | 0.091 | 22.000 | 1.000 | 1.000 | 1.000 | 1.000 | 1.027 | 22.605 |
| Jiangsu | Yancheng | 2 | 98 | 664 | 164 | 387 | 8 | 22 | 0.500 | 0.091 | 22.000 | 0.833 | 1.000 | 0.833 | 1.200 | 1.027 | 27.126 |
| Jiangsu | Taizhou | 2 | 98 | 664 | 79 | 366 | 8 | 24 | 0.500 | 0.083 | 24.000 | 0.833 | 1.000 | 0.833 | 1.200 | 1.027 | 29.592 |
| Jiangsu | Taizhou | 2 | 98 | 664 | 79 | 365 | 8 | 24 | 0.500 | 0.083 | 24.000 | 0.750 | 1.000 | 0.750 | 1.333 | 1.027 | 32.880 |
| Jiangsu | Lianyungang | 2 | 97 | 664 | 89 | 357 | 8 | 4 | 0.500 | 0.500 | 4.000 | 1.000 | 1.000 | 1.000 | 1.000 | 1.027 | 4.110 |
| Jiangsu | Lianyungang | 2 | 97 | 664 | 89 | 356 | 8 | 4 | 0.500 | 0.500 | 4.000 | 1.000 | 1.000 | 1.000 | 1.000 | 1.027 | 4.110 |
| Jiangsu | Yancheng | 2 | 97 | 664 | 164 | 385 | 8 | 4 | 0.500 | 0.500 | 4.000 | 1.000 | 1.000 | 1.000 | 1.000 | 1.027 | 4.110 |
| Jiangsu | Yancheng | 2 | 97 | 664 | 164 | 386 | 8 | 4 | 0.500 | 0.500 | 4.000 | 0.909 | 1.000 | 0.909 | 1.100 | 1.027 | 4.521 |
| Jiangsu | Taizhou | 2 | 97 | 664 | 79 | 368 | 8 | 6 | 0.500 | 0.333 | 6.000 | 1.000 | 1.000 | 1.000 | 1.000 | 1.027 | 6.165 |
| Jiangsu | Taizhou | 2 | 97 | 664 | 79 | 367 | 8 | 6 | 0.500 | 0.333 | 6.000 | 1.000 | 1.000 | 1.000 | 1.000 | 1.027 | 6.165 |
| Jiangsu | Suqian | 2 | 97 | 664 | 229 | 380 | 8 | 6 | 0.500 | 0.333 | 6.000 | 1.000 | 1.000 | 1.000 | 1.000 | 1.027 | 6.165 |
| Jiangsu | Suqian | 2 | 97 | 664 | 229 | 378 | 8 | 6 | 0.500 | 0.333 | 6.000 | 1.000 | 1.000 | 1.000 | 1.000 | 1.027 | 6.165 |
| Jiangsu | Taizhou | 2 | 96 | 664 | 79 | 369 | 8 | 12 | 0.500 | 0.167 | 12.000 | 1.000 | 1.000 | 1.000 | 1.000 | 1.027 | 12.330 |
| Jiangsu | Taizhou | 2 | 96 | 664 | 79 | 364 | 8 | 12 | 0.500 | 0.167 | 12.000 | 1.000 | 1.000 | 1.000 | 1.000 | 1.027 | 12.330 |
| Jiangsu | Lianyungang | 2 | 96 | 664 | 89 | 354 | 8 | 13 | 0.500 | 0.154 | 13.000 | 1.000 | 1.000 | 1.000 | 1.000 | 1.027 | 13.357 |
| Jiangsu | Lianyungang | 2 | 96 | 664 | 89 | 353 | 8 | 13 | 0.500 | 0.154 | 13.000 | 1.000 | 1.000 | 1.000 | 1.000 | 1.027 | 13.357 |
| Jiangsu | Suqian | 2 | 96 | 664 | 229 | 377 | 8 | 15 | 0.500 | 0.133 | 15.000 | 1.000 | 1.000 | 1.000 | 1.000 | 1.027 | 15.412 |
| Jiangsu | Suqian | 2 | 96 | 664 | 229 | 376 | 8 | 15 | 0.500 | 0.133 | 15.000 | 1.000 | 1.000 | 1.000 | 1.000 | 1.027 | 15.412 |
| Jiangsu | Yancheng | 2 | 96 | 664 | 164 | 383 | 8 | 20 | 0.500 | 0.100 | 20.000 | 1.000 | 1.000 | 1.000 | 1.000 | 1.027 | 20.550 |
| Jiangsu | Yancheng | 2 | 96 | 664 | 164 | 382 | 8 | 20 | 0.500 | 0.100 | 20.000 | 1.000 | 1.000 | 1.000 | 1.000 | 1.027 | 20.550 |
| Jilin | Changchun | 3 | 95 | 157 | 186 | 347 | 1 | 23 | 1.000 | 0.087 | 11.500 | 1.000 | 1.000 | 1.000 | 1.000 | 1.138 | 13.089 |
| Jilin | Changchun | 3 | 95 | 157 | 186 | 350 | 1 | 23 | 1.000 | 0.087 | 11.500 | 0.750 | 1.000 | 0.750 | 1.333 | 1.138 | 17.451 |
| Jilin | Changchun | 3 | 94 | 157 | 186 | 348 | 1 | 20 | 1.000 | 0.100 | 10.000 | 1.000 | 1.000 | 1.000 | 1.000 | 1.138 | 11.381 |
| Jilin | Changchun | 3 | 94 | 157 | 186 | 346 | 1 | 20 | 1.000 | 0.100 | 10.000 | 1.000 | 1.000 | 1.000 | 1.000 | 1.138 | 11.381 |
| Jilin | Changchun | 3 | 93 | 157 | 186 | 351 | 1 | 19 | 1.000 | 0.105 | 9.500 | 1.000 | 1.000 | 1.000 | 1.000 | 1.138 | 10.812 |
| Jilin | Changchun | 3 | 93 | 157 | 186 | 349 | 1 | 19 | 1.000 | 0.105 | 9.500 | 1.000 | 1.000 | 1.000 | 1.000 | 1.138 | 10.812 |
| Jilin | Tonghua | 2 | 92 | 157 | 72 | 341 | 3 | 11 | 0.667 | 0.182 | 8.250 | 1.000 | 1.000 | 1.000 | 1.000 | 1.138 | 9.390 |
| Jilin | Siping | 2 | 92 | 157 | 78 | 336 | 3 | 12 | 0.667 | 0.167 | 9.000 | 1.000 | 1.000 | 1.000 | 1.000 | 1.138 | 10.243 |
| Jilin | Siping | 2 | 92 | 157 | 78 | 339 | 3 | 12 | 0.667 | 0.167 | 9.000 | 0.750 | 1.000 | 0.750 | 1.333 | 1.138 | 13.658 |
| Jilin | Tonghua | 2 | 92 | 157 | 72 | 340 | 3 | 11 | 0.667 | 0.182 | 8.250 | 0.545 | 1.000 | 0.545 | 1.833 | 1.138 | 17.214 |
| Jilin | #N/A | 2 | 92 | 157 |  | N/A | 3 | 22 | 0.667 | 0.091 |  |  |  |  |  |  |  |
| Jilin | #N/A | 2 | 92 | 157 |  | N/A | 3 | 22 | 0.667 | 0.091 |  |  |  |  |  |  |  |
| Jilin | Siping | 2 | 91 | 157 | 78 | 338 | 3 | 1 | 0.667 | 1.000 | 1.500 | 1.000 | 1.000 | 1.000 | 1.000 | 1.138 | 1.707 |
| Jilin | Tonghua | 2 | 91 | 157 | 72 | 344 | 3 | 2 | 0.667 | 1.000 | 1.500 | 1.000 | 1.000 | 1.000 | 1.000 | 1.138 | 1.707 |
| Jilin | Tonghua | 2 | 91 | 157 | 72 | 343 | 3 | 2 | 0.667 | 1.000 | 1.500 | 1.000 | 1.000 | 1.000 | 1.000 | 1.138 | 1.707 |
| Jilin | #N/A | 2 | 91 | 157 |  | N/A | 3 | 8 | 0.667 | 0.250 |  |  |  |  |  |  |  |
| Jilin | #N/A | 2 | 91 | 157 |  | N/A | 3 | 8 | 0.667 | 0.250 |  |  |  |  |  |  |  |
| Jilin | Tonghua | 2 | 90 | 157 | 72 | 345 | 3 | 8 | 0.667 | 0.250 | 6.000 | 1.000 | 1.000 | 1.000 | 1.000 | 1.138 | 6.829 |
| Jilin | Tonghua | 2 | 90 | 157 | 72 | 342 | 3 | 8 | 0.667 | 0.250 | 6.000 | 1.000 | 1.000 | 1.000 | 1.000 | 1.138 | 6.829 |
| Jilin | Siping | 2 | 90 | 157 | 78 | 335 | 3 | 9 | 0.667 | 0.222 | 6.750 | 1.000 | 1.000 | 1.000 | 1.000 | 1.138 | 7.682 |
| Jilin | Siping | 2 | 90 | 157 | 78 | 337 | 3 | 9 | 0.667 | 0.222 | 6.750 | 0.524 | 1.000 | 0.524 | 1.909 | 1.138 | 14.666 |
| Jilin | #N/A | 2 | 90 | 157 |  | N/A | 3 | 20 | 0.667 | 0.100 |  |  |  |  |  |  |  |
| Jilin | #N/A | 2 | 90 | 157 |  | N/A | 3 | 20 | 0.667 | 0.100 |  |  |  |  |  |  |  |
| Jilin | Liaoyuan | 1 | 89 | 157 | 33 | 331 | 4 | 6 | 0.500 | 0.333 | 6.000 | 1.000 | 1.000 | 1.000 | 1.000 | 1.138 | 6.829 |
| Jilin | Baishan | 1 | 89 | 157 | 40 | 327 | 4 | 8 | 0.500 | 0.250 | 8.000 | 1.000 | 1.000 | 1.000 | 1.000 | 1.138 | 9.105 |
| Jilin | Baishan | 1 | 89 | 157 | 40 | 328 | 4 | 8 | 0.500 | 0.250 | 8.000 | 0.900 | 1.000 | 0.900 | 1.111 | 1.138 | 10.117 |
| Jilin | Liaoyuan | 1 | 89 | 157 | 33 | 330 | 4 | 6 | 0.500 | 0.333 | 6.000 | 0.500 | 1.000 | 0.500 | 2.000 | 1.138 | 13.658 |
| Jilin | Liaoyuan | 1 | 88 | 157 | 33 | 334 | 4 | 1 | 0.500 | 1.000 | 2.000 | 1.000 | 1.000 | 1.000 | 1.000 | 1.138 | 2.276 |
| Jilin | Liaoyuan | 1 | 87 | 157 | 33 | 333 | 4 | 4 | 0.500 | 0.500 | 4.000 | 0.857 | 1.000 | 0.857 | 1.167 | 1.138 | 5.311 |
| Jilin | Liaoyuan | 1 | 87 | 157 | 33 | 332 | 4 | 4 | 0.500 | 0.500 | 4.000 | 0.571 | 1.000 | 0.571 | 1.750 | 1.138 | 7.967 |
| Jilin | Baishan | 1 | 87 | 157 | 40 | 329 | 4 | 7 | 0.500 | 0.286 | 7.000 | 1.000 | 1.000 | 1.000 | 1.000 | 1.138 | 7.967 |
| Jilin | Baishan | 1 | 87 | 157 | 40 | 326 | 4 | 7 | 0.500 | 0.286 | 7.000 | 1.000 | 1.000 | 1.000 | 1.000 | 1.138 | 7.967 |
| Hunan | Hengyang | 3 | 86 | 1019 | 149 | 304 | 3 | 18 | 0.667 | 0.111 | 13.500 | 1.000 | 1.000 | 1.000 | 1.000 | 1.146 | 15.469 |
| Hunan | Hengyang | 3 | 86 | 1019 | 149 | 303 | 3 | 18 | 0.667 | 0.111 | 13.500 | 1.000 | 1.000 | 1.000 | 1.000 | 1.146 | 15.469 |
| Hunan | Changsha | 3 | 86 | 1019 | 232 | 319 | 3 | 30 | 0.667 | 0.067 | 22.500 | 0.857 | 1.000 | 0.857 | 1.167 | 1.146 | 30.078 |
| Hunan | Changsha | 3 | 86 | 1019 | 232 | 317 | 3 | 30 | 0.667 | 0.067 | 22.500 | 0.800 | 1.000 | 0.800 | 1.250 | 1.146 | 32.226 |
| Hunan | Hengyang | 3 | 85 | 1019 | 149 | 302 | 3 | 6 | 0.667 | 0.333 | 4.500 | 1.000 | 1.000 | 1.000 | 1.000 | 1.146 | 5.156 |
| Hunan | Hengyang | 3 | 85 | 1019 | 149 | 299 | 3 | 6 | 0.667 | 0.333 | 4.500 | 1.000 | 1.000 | 1.000 | 1.000 | 1.146 | 5.156 |
| Hunan | Changsha | 3 | 85 | 1019 | 232 | 315 | 3 | 12 | 0.667 | 0.167 | 9.000 | 1.000 | 1.000 | 1.000 | 1.000 | 1.146 | 10.312 |
| Hunan | Changsha | 3 | 85 | 1019 | 232 | 314 | 3 | 12 | 0.667 | 0.167 | 9.000 | 1.000 | 1.000 | 1.000 | 1.000 | 1.146 | 10.312 |
| Hunan | Hengyang | 3 | 84 | 1019 | 149 | 301 | 3 | 17 | 0.667 | 0.118 | 12.750 | 1.000 | 1.000 | 1.000 | 1.000 | 1.146 | 14.609 |
| Hunan | Hengyang | 3 | 84 | 1019 | 149 | 300 | 3 | 17 | 0.667 | 0.118 | 12.750 | 1.000 | 1.000 | 1.000 | 1.000 | 1.146 | 14.609 |
| Hunan | Changsha | 3 | 84 | 1019 | 232 | 318 | 3 | 23 | 0.667 | 0.087 | 17.250 | 1.000 | 1.000 | 1.000 | 1.000 | 1.146 | 19.766 |
| Hunan | Changsha | 3 | 84 | 1019 | 232 | 316 | 3 | 23 | 0.667 | 0.087 | 17.250 | 1.000 | 1.000 | 1.000 | 1.000 | 1.146 | 19.766 |
| Hunan | Chenzhou | 2 | 83 | 1019 | 109 | 297 | 9 | 15 | 0.333 | 0.133 | 22.500 | 1.000 | 1.000 | 1.000 | 1.000 | 1.146 | 25.781 |
| Hunan | Chenzhou | 2 | 83 | 1019 | 109 | 296 | 9 | 15 | 0.333 | 0.133 | 22.500 | 1.000 | 1.000 | 1.000 | 1.000 | 1.146 | 25.781 |
| Hunan | Yiyang | 2 | 83 | 1019 | 92 | 305 | 9 | 14 | 0.333 | 0.143 | 21.000 | 0.833 | 1.000 | 0.833 | 1.200 | 1.146 | 28.875 |
| Hunan | Zhuzhou | 2 | 83 | 1019 | 185 | 320 | 9 | 24 | 0.333 | 0.083 | 36.000 | 1.000 | 1.000 | 1.000 | 1.000 | 1.146 | 41.250 |
| Hunan | Zhuzhou | 2 | 83 | 1019 | 185 | 323 | 9 | 24 | 0.333 | 0.083 | 36.000 | 0.500 | 1.000 | 0.500 | 2.000 | 1.146 | 82.500 |
| Hunan | Yiyang | 2 | 83 | 1019 | 92 | N/A | 9 | 14 | 0.333 | 0.143 |  |  |  |  |  |  |  |
| Hunan | Chenzhou | 2 | 82 | 1019 | 109 | 293 | 9 | 4 | 0.333 | 0.500 | 6.000 | 1.000 | 1.000 | 1.000 | 1.000 | 1.146 | 6.875 |
| Hunan | Yiyang | 2 | 82 | 1019 | 92 | 309 | 9 | 4 | 0.333 | 0.500 | 6.000 | 1.000 | 1.000 | 1.000 | 1.000 | 1.146 | 6.875 |
| Hunan | Yiyang | 2 | 82 | 1019 | 92 | 307 | 9 | 4 | 0.333 | 0.500 | 6.000 | 1.000 | 1.000 | 1.000 | 1.000 | 1.146 | 6.875 |
| Hunan | Zhuzhou | 2 | 82 | 1019 | 185 | 324 | 9 | 6 | 0.333 | 0.333 | 9.000 | 1.000 | 1.000 | 1.000 | 1.000 | 1.146 | 10.312 |
| Hunan | Zhuzhou | 2 | 82 | 1019 | 185 | 322 | 9 | 6 | 0.333 | 0.333 | 9.000 | 1.000 | 1.000 | 1.000 | 1.000 | 1.146 | 10.312 |
| Hunan | Chenzhou | 2 | 82 | 1019 | 109 | 298 | 9 | 4 | 0.333 | 0.500 | 6.000 | 0.533 | 1.000 | 0.533 | 1.875 | 1.146 | 12.891 |
| Hunan | Chenzhou | 2 | 81 | 1019 | 109 | 295 | 9 | 12 | 0.333 | 0.167 | 18.000 | 1.000 | 1.000 | 1.000 | 1.000 | 1.146 | 20.625 |
| Hunan | Chenzhou | 2 | 81 | 1019 | 109 | 294 | 9 | 12 | 0.333 | 0.167 | 18.000 | 1.000 | 1.000 | 1.000 | 1.000 | 1.146 | 20.625 |
| Hunan | Yiyang | 2 | 81 | 1019 | 92 | 306 | 9 | 12 | 0.333 | 0.167 | 18.000 | 1.000 | 1.000 | 1.000 | 1.000 | 1.146 | 20.625 |
| Hunan | Yiyang | 2 | 81 | 1019 | 92 | 308 | 9 | 12 | 0.333 | 0.167 | 18.000 | 0.833 | 1.000 | 0.833 | 1.200 | 1.146 | 24.750 |
| Hunan | Zhuzhou | 2 | 81 | 1019 | 185 | 321 | 9 | 19 | 0.333 | 0.105 | 28.500 | 1.000 | 1.000 | 1.000 | 1.000 | 1.146 | 32.656 |
| Hunan | Zhuzhou | 2 | 81 | 1019 | 185 | 325 | 9 | 19 | 0.333 | 0.105 | 28.500 | 1.000 | 1.000 | 1.000 | 1.000 | 1.146 | 32.656 |
| Hunan | Zhangjiajie | 1 | 80 | 1019 | 32 | 312 | 1 | 7 | 1.000 | 0.286 | 3.500 | 1.000 | 1.000 | 1.000 | 1.000 | 1.146 | 4.010 |
| Hunan | Zhangjiajie | 1 | 80 | 1019 | 32 | 310 | 1 | 7 | 1.000 | 0.286 | 3.500 | 1.000 | 1.000 | 1.000 | 1.000 | 1.146 | 4.010 |
| Hunan | Zhangjiajie | 1 | 79 | 1019 | 32 | 313 | 1 | 6 | 1.000 | 0.333 | 3.000 | 1.000 | 1.000 | 1.000 | 1.000 | 1.146 | 3.437 |
| Hunan | Zhangjiajie | 1 | 79 | 1019 | 32 | 311 | 1 | 6 | 1.000 | 0.333 | 3.000 | 1.000 | 1.000 | 1.000 | 1.000 | 1.146 | 3.437 |
| Hubei | Jingmen | 2 | 78 | 68139 | 56 | 276 | 8 | 8 | 0.500 | 0.250 | 8.000 | 1.000 | 1.000 | 1.000 | 1.000 | 1.321 | 10.567 |
| Hubei | Jingmen | 2 | 78 | 68139 | 56 | 272 | 8 | 8 | 0.500 | 0.250 | 8.000 | 1.000 | 1.000 | 1.000 | 1.000 | 1.321 | 10.567 |
| Hubei | Huangshi | 2 | 78 | 68139 | 42 | 267 | 8 | 10 | 0.500 | 0.200 | 10.000 | 1.000 | 1.000 | 1.000 | 1.000 | 1.321 | 13.209 |
| Hubei | Huangshi | 2 | 78 | 68139 | 42 | 270 | 8 | 10 | 0.500 | 0.200 | 10.000 | 0.923 | 1.000 | 0.923 | 1.083 | 1.321 | 14.310 |
| Hubei | Xiaogan | 2 | 78 | 68139 | 60 | 288 | 8 | 11 | 0.500 | 0.182 | 11.000 | 1.000 | 1.000 | 1.000 | 1.000 | 1.321 | 14.530 |
| Hubei | Xiangyang | 2 | 78 | 68139 | 81 | 283 | 8 | 10 | 0.500 | 0.200 | 10.000 | 0.875 | 1.000 | 0.875 | 1.143 | 1.321 | 15.096 |
| Hubei | Xiangyang | 2 | 78 | 68139 | 81 | 287 | 8 | 10 | 0.500 | 0.200 | 10.000 | 0.727 | 1.000 | 0.727 | 1.375 | 1.321 | 18.163 |
| Hubei | Xiaogan | 2 | 78 | 68139 | 60 | 289 | 8 | 11 | 0.500 | 0.182 | 11.000 | 0.778 | 1.000 | 0.778 | 1.286 | 1.321 | 18.682 |
| Hubei | Huangshi | 2 | 77 | 68139 | 42 | 269 | 8 | 2 | 0.500 | 1.000 | 2.000 | 1.000 | 1.000 | 1.000 | 1.000 | 1.321 | 2.642 |
| Hubei | Huangshi | 2 | 77 | 68139 | 42 | 268 | 8 | 2 | 0.500 | 1.000 | 2.000 | 1.000 | 1.000 | 1.000 | 1.000 | 1.321 | 2.642 |
| Hubei | Jingmen | 2 | 77 | 68139 | 56 | 274 | 8 | 1 | 0.500 | 1.000 | 2.000 | 1.000 | 1.000 | 1.000 | 1.000 | 1.321 | 2.642 |
| Hubei | Xiangyang | 2 | 77 | 68139 | 81 | 286 | 8 | 3 | 0.500 | 0.667 | 3.000 | 1.000 | 1.000 | 1.000 | 1.000 | 1.321 | 3.963 |
| Hubei | Xiangyang | 2 | 77 | 68139 | 81 | 285 | 8 | 3 | 0.500 | 0.667 | 3.000 | 1.000 | 1.000 | 1.000 | 1.000 | 1.321 | 3.963 |
| Hubei | Xiaogan | 2 | 77 | 68139 | 60 | 290 | 8 | 1 | 0.500 | 1.000 | 2.000 | 0.417 | 1.000 | 0.417 | 2.400 | 1.321 | 6.340 |
| Hubei | Jingmen | 2 | 76 | 68139 | 56 | 275 | 8 | 6 | 0.500 | 0.333 | 6.000 | 1.000 | 1.000 | 1.000 | 1.000 | 1.321 | 7.926 |
| Hubei | Jingmen | 2 | 76 | 68139 | 56 | 273 | 8 | 6 | 0.500 | 0.333 | 6.000 | 1.000 | 1.000 | 1.000 | 1.000 | 1.321 | 7.926 |
| Hubei | Huangshi | 2 | 76 | 68139 | 42 | 271 | 8 | 8 | 0.500 | 0.250 | 8.000 | 1.000 | 1.000 | 1.000 | 1.000 | 1.321 | 10.567 |
| Hubei | Xiaogan | 2 | 76 | 68139 | 60 | 292 | 8 | 8 | 0.500 | 0.250 | 8.000 | 1.000 | 1.000 | 1.000 | 1.000 | 1.321 | 10.567 |
| Hubei | Xiaogan | 2 | 76 | 68139 | 60 | 291 | 8 | 8 | 0.500 | 0.250 | 8.000 | 0.917 | 1.000 | 0.917 | 1.091 | 1.321 | 11.528 |
| Hubei | Xiangyang | 2 | 76 | 68139 | 81 | 284 | 8 | 9 | 0.500 | 0.222 | 9.000 | 1.000 | 1.000 | 1.000 | 1.000 | 1.321 | 11.888 |
| Hubei | Xiangyang | 2 | 76 | 68139 | 81 | 282 | 8 | 9 | 0.500 | 0.222 | 9.000 | 1.000 | 1.000 | 1.000 | 1.000 | 1.321 | 11.888 |
| Hubei | Huangshi | 2 | 76 | 68139 | 42 | 266 | 8 | 8 | 0.500 | 0.250 | 8.000 | 0.778 | 1.000 | 0.778 | 1.286 | 1.321 | 13.587 |
| Hubei | Suizhou | 1 | 75 | 68139 | 42 | 277 | 3 | 5 | 0.667 | 0.400 | 3.750 | 0.800 | 1.000 | 0.800 | 1.250 | 1.321 | 6.192 |
| Hubei | Suizhou | 1 | 75 | 68139 | 42 | 281 | 3 | 5 | 0.667 | 0.400 | 3.750 | 0.667 | 1.000 | 0.667 | 1.500 | 1.321 | 7.430 |
| Hubei | Ezhou | 1 | 75 | 68139 | 20 | 261 | 3 | 6 | 0.667 | 0.333 | 4.500 | 0.667 | 0.500 | 0.333 | 3.000 | 1.321 | 17.833 |
| Hubei | Ezhou | 1 | 75 | 68139 | 20 | N/A | 3 | 6 | 0.667 | 0.333 |  |  |  |  |  |  |  |
| Hubei | Ezhou | 1 | 74 | 68139 | 20 | 265 | 3 | 2 | 0.667 | 1.000 | 1.500 | 1.000 | 1.000 | 1.000 | 1.000 | 1.321 | 1.981 |
| Hubei | Suizhou | 1 | 74 | 68139 | 42 | 280 | 3 | 1 | 0.667 | 1.000 | 1.500 | 1.000 | 1.000 | 1.000 | 1.000 | 1.321 | 1.981 |
| Hubei | Ezhou | 1 | 74 | 68139 | 20 | 262 | 3 | 2 | 0.667 | 1.000 | 1.500 | 0.778 | 1.000 | 0.778 | 1.286 | 1.321 | 2.548 |
| Hubei | Suizhou | 1 | 73 | 68139 | 42 | 278 | 3 | 6 | 0.667 | 0.333 | 4.500 | 0.750 | 1.000 | 0.750 | 1.333 | 1.321 | 7.926 |
| Hubei | Ezhou | 1 | 73 | 68139 | 20 | 263 | 3 | 8 | 0.667 | 0.250 | 6.000 | 1.000 | 1.000 | 1.000 | 1.000 | 1.321 | 7.926 |
| Hubei | Ezhou | 1 | 73 | 68139 | 20 | 264 | 3 | 8 | 0.667 | 0.250 | 6.000 | 0.833 | 1.000 | 0.833 | 1.200 | 1.321 | 9.511 |
| Hubei | Suizhou | 1 | 73 | 68139 | 42 | 279 | 3 | 6 | 0.667 | 0.333 | 4.500 | 0.625 | 1.000 | 0.625 | 1.600 | 1.321 | 9.511 |
| Heilongjiang | Harbin | 3 | 72 | 948 | 326 | 243 | 1 | 30 | 1.000 | 0.067 | 15.000 | 1.000 | 1.000 | 1.000 | 1.000 | 0.866 | 12.997 |
| Heilongjiang | Harbin | 3 | 72 | 948 | 326 | 238 | 1 | 30 | 1.000 | 0.067 | 15.000 | 1.000 | 1.000 | 1.000 | 1.000 | 0.866 | 12.997 |
| Heilongjiang | Harbin | 3 | 71 | 948 | 326 | 240 | 1 | 20 | 1.000 | 0.100 | 10.000 | 1.000 | 1.000 | 1.000 | 1.000 | 0.866 | 8.665 |
| Heilongjiang | Harbin | 3 | 71 | 948 | 326 | 242 | 1 | 20 | 1.000 | 0.100 | 10.000 | 0.583 | 1.000 | 0.583 | 1.714 | 0.866 | 14.853 |
| Heilongjiang | Harbin | 3 | 70 | 948 | 326 | 239 | 1 | 26 | 1.000 | 0.077 | 13.000 | 1.000 | 1.000 | 1.000 | 1.000 | 0.866 | 11.264 |
| Heilongjiang | Harbin | 3 | 70 | 948 | 326 | 241 | 1 | 26 | 1.000 | 0.077 | 13.000 | 0.667 | 1.000 | 0.667 | 1.500 | 0.866 | 16.896 |
| Heilongjiang | Suihua | 2 | 69 | 948 | 40 | 256 | 6 | 7 | 0.500 | 0.286 | 7.000 | 1.000 | 1.000 | 1.000 | 1.000 | 0.866 | 6.065 |
| Heilongjiang | Suihua | 2 | 69 | 948 | 40 | 253 | 6 | 7 | 0.500 | 0.286 | 7.000 | 0.889 | 1.000 | 0.889 | 1.125 | 0.866 | 6.823 |
| Heilongjiang | Daqing | 2 | 69 | 948 | 116 | 237 | 6 | 14 | 0.500 | 0.143 | 14.000 | 0.909 | 1.000 | 0.909 | 1.100 | 0.866 | 13.343 |
| Heilongjiang | Jiamusi | 2 | 69 | 948 | 95 | 248 | 6 | 17 | 0.500 | 0.118 | 17.000 | 0.833 | 1.000 | 0.833 | 1.200 | 0.866 | 17.676 |
| Heilongjiang | Jiamusi | 2 | 69 | 948 | 95 | 244 | 6 | 17 | 0.500 | 0.118 | 17.000 | 0.667 | 1.000 | 0.667 | 1.500 | 0.866 | 22.094 |
| Heilongjiang | Daqing | 2 | 69 | 948 | 116 | N/A | 6 | 14 | 0.500 | 0.143 |  |  |  |  |  |  |  |
| Heilongjiang | Suihua | 2 | 68 | 948 | 40 | 255 | 6 | 1 | 0.500 | 1.000 | 2.000 | 1.000 | 1.000 | 1.000 | 1.000 | 0.866 | 1.733 |
| Heilongjiang | Jiamusi | 2 | 68 | 948 | 95 | 245 | 6 | 4 | 0.500 | 0.500 | 4.000 | 1.000 | 1.000 | 1.000 | 1.000 | 0.866 | 3.466 |
| Heilongjiang | Jiamusi | 2 | 68 | 948 | 95 | 247 | 6 | 4 | 0.500 | 0.500 | 4.000 | 1.000 | 1.000 | 1.000 | 1.000 | 0.866 | 3.466 |
| Heilongjiang | Daqing | 2 | 68 | 948 | 116 | 236 | 6 | 5 | 0.500 | 0.400 | 5.000 | 1.000 | 1.000 | 1.000 | 1.000 | 0.866 | 4.332 |
| Heilongjiang | Daqing | 2 | 68 | 948 | 116 | 234 | 6 | 5 | 0.500 | 0.400 | 5.000 | 1.000 | 1.000 | 1.000 | 1.000 | 0.866 | 4.332 |
| Heilongjiang | Suihua | 2 | 67 | 948 | 40 | 254 | 6 | 7 | 0.500 | 0.286 | 7.000 | 1.000 | 1.000 | 1.000 | 1.000 | 0.866 | 6.065 |
| Heilongjiang | Jiamusi | 2 | 67 | 948 | 95 | 246 | 6 | 8 | 0.500 | 0.250 | 8.000 | 0.909 | 1.000 | 0.909 | 1.100 | 0.866 | 7.625 |
| Heilongjiang | Daqing | 2 | 67 | 948 | 116 | 235 | 6 | 15 | 0.500 | 0.133 | 15.000 | 1.000 | 1.000 | 1.000 | 1.000 | 0.866 | 12.997 |
| Heilongjiang | Daqing | 2 | 67 | 948 | 116 | 233 | 6 | 15 | 0.500 | 0.133 | 15.000 | 1.000 | 1.000 | 1.000 | 1.000 | 0.866 | 12.997 |
| Heilongjiang | Jiamusi | 2 | 67 | 948 | 95 | N/A | 6 | 8 | 0.500 | 0.250 |  |  |  |  |  |  |  |
| Heilongjiang | Suihua | 2 | 67 | 948 | 40 | N/A | 6 | 7 | 0.500 | 0.286 |  |  |  |  |  |  |  |
| Heilongjiang | Shuangyashan | 1 | 66 | 948 | 51 | 249 | 5 | 10 | 0.400 | 0.200 | 12.500 | 1.000 | 1.000 | 1.000 | 1.000 | 0.866 | 10.831 |
| Heilongjiang | Yichun | 1 | 66 | 948 | 39 | 257 | 5 | 7 | 0.400 | 0.286 | 8.750 | 1.000 | 0.500 | 0.500 | 2.000 | 0.866 | 15.163 |
| Heilongjiang | Shuangyashan | 1 | 66 | 948 | 51 | 251 | 5 | 10 | 0.400 | 0.200 | 12.500 | 0.625 | 1.000 | 0.625 | 1.600 | 0.866 | 17.329 |
| Heilongjiang | Yichun | 1 | 66 | 948 | 39 | N/A | 5 | 7 | 0.400 | 0.286 |  |  |  |  |  |  |  |
| Heilongjiang | Yichun | 1 | 65 | 948 | 39 | 259 | 5 | 1 | 0.400 | 1.000 | 2.500 | 0.667 | 1.000 | 0.667 | 1.500 | 0.866 | 3.249 |
| Heilongjiang | Yichun | 1 | 64 | 948 | 39 | 258 | 5 | 6 | 0.400 | 0.500 | 5.000 | 1.000 | 1.000 | 1.000 | 1.000 | 0.866 | 4.332 |
| Heilongjiang | Shuangyashan | 1 | 64 | 948 | 51 | 250 | 5 | 5 | 0.400 | 0.400 | 6.250 | 1.000 | 1.000 | 1.000 | 1.000 | 0.866 | 5.415 |
| Heilongjiang | Shuangyashan | 1 | 64 | 948 | 51 | 252 | 5 | 5 | 0.400 | 0.400 | 6.250 | 0.900 | 1.000 | 0.900 | 1.111 | 0.866 | 6.017 |
| Heilongjiang | Yichun | 1 | 64 | 948 | 39 | 260 | 5 | 6 | 0.400 | 0.500 | 5.000 | 0.333 | 1.000 | 0.333 | 3.000 | 0.866 | 12.997 |
| Heilongjiang | Yichun | 1 | 64 | 948 | 39 | N/A | 5 | 6 | 0.400 | 0.500 |  |  |  |  |  |  |  |
| Henan | Luoyang | 3 | 63 | 1276 | 154 | 216 | 6 | 20 | 0.167 | 0.100 | 60.000 | 1.000 | 1.000 | 1.000 | 1.000 | 1.701 | 102.043 |
| Henan | Luoyang | 3 | 63 | 1276 | 154 | N/A | 6 | 20 | 0.167 | 0.100 |  |  |  |  |  |  |  |
| Henan | Luoyang | 3 | 62 | 1276 | 154 | 213 | 6 | 6 | 0.167 | 0.333 | 18.000 | 1.000 | 1.000 | 1.000 | 1.000 | 1.701 | 30.613 |
| Henan | Luoyang | 3 | 62 | 1276 | 154 | 212 | 6 | 6 | 0.167 | 0.333 | 18.000 | 1.000 | 1.000 | 1.000 | 1.000 | 1.701 | 30.613 |
| Henan | Luoyang | 3 | 61 | 1276 | 154 | 215 | 6 | 16 | 0.167 | 0.125 | 48.000 | 1.000 | 1.000 | 1.000 | 1.000 | 1.701 | 81.634 |
| Henan | Luoyang | 3 | 61 | 1276 | 154 | 214 | 6 | 16 | 0.167 | 0.125 | 48.000 | 0.750 | 1.000 | 0.750 | 1.333 | 1.701 | 108.845 |
| Henan | Jiaozuo | 2 | 60 | 1276 | 83 | 210 | 10 | 12 | 0.500 | 0.167 | 12.000 | 1.000 | 1.000 | 1.000 | 1.000 | 1.701 | 20.409 |
| Henan | Xinxiang | 2 | 60 | 1276 | 118 | 232 | 10 | 12 | 0.500 | 0.167 | 12.000 | 1.000 | 1.000 | 1.000 | 1.000 | 1.701 | 20.409 |
| Henan | Sanmenxia | 2 | 60 | 1276 | 51 | 224 | 10 | 15 | 0.500 | 0.133 | 15.000 | 1.000 | 1.000 | 1.000 | 1.000 | 1.701 | 25.511 |
| Henan | Sanmenxia | 2 | 60 | 1276 | 51 | 223 | 10 | 15 | 0.500 | 0.133 | 15.000 | 1.000 | 1.000 | 1.000 | 1.000 | 1.701 | 25.511 |
| Henan | Xinxiang | 2 | 60 | 1276 | 118 | 227 | 10 | 12 | 0.500 | 0.167 | 12.000 | 0.750 | 1.000 | 0.750 | 1.333 | 1.701 | 27.211 |
| Henan | Jiaozuo | 2 | 60 | 1276 | 83 | 211 | 10 | 12 | 0.500 | 0.167 | 12.000 | 0.727 | 1.000 | 0.727 | 1.375 | 1.701 | 28.062 |
| Henan | Luohe | 2 | 60 | 1276 | 45 | 217 | 10 | 9 | 0.500 | 0.222 | 9.000 | 0.500 | 1.000 | 0.500 | 2.000 | 1.701 | 30.613 |
| Henan | Anyang | 2 | 60 | 1276 | 95 | 205 | 10 | 11 | 0.500 | 0.091 | 22.000 | 1.000 | 1.000 | 1.000 | 1.000 | 1.701 | 37.416 |
| Henan | Luohe | 2 | 60 | 1276 | 45 | 222 | 10 | 9 | 0.500 | 0.222 | 9.000 | 0.375 | 1.000 | 0.375 | 2.667 | 1.701 | 40.817 |
| Henan | Luohe | 2 | 59 | 1276 | 45 | 220 | 10 | 2 | 0.500 | 1.000 | 2.000 | 1.000 | 1.000 | 1.000 | 1.000 | 1.701 | 3.401 |
| Henan | Sanmenxia | 2 | 59 | 1276 | 51 | 226 | 10 | 1 | 0.500 | 1.000 | 2.000 | 1.000 | 1.000 | 1.000 | 1.000 | 1.701 | 3.401 |
| Henan | Luohe | 2 | 59 | 1276 | 45 | 219 | 10 | 2 | 0.500 | 1.000 | 2.000 | 0.964 | 1.000 | 0.964 | 1.037 | 1.701 | 3.527 |
| Henan | Anyang | 2 | 59 | 1276 | 95 | 203 | 10 | 3 | 0.500 | 1.000 | 2.000 | 1.000 | 0.667 | 0.667 | 1.500 | 1.701 | 5.102 |
| Henan | Anyang | 2 | 59 | 1276 | 95 | 204 | 10 | 3 | 0.500 | 1.000 | 2.000 | 1.000 | 0.667 | 0.667 | 1.500 | 1.701 | 5.102 |
| Henan | Xinxiang | 2 | 59 | 1276 | 118 | 231 | 10 | 4 | 0.500 | 0.500 | 4.000 | 1.000 | 1.000 | 1.000 | 1.000 | 1.701 | 6.803 |
| Henan | Xinxiang | 2 | 59 | 1276 | 118 | 228 | 10 | 4 | 0.500 | 0.500 | 4.000 | 1.000 | 1.000 | 1.000 | 1.000 | 1.701 | 6.803 |
| Henan | Jiaozuo | 2 | 59 | 1276 | 83 | 207 | 10 | 3 | 0.500 | 0.667 | 3.000 | 0.455 | 0.500 | 0.227 | 4.400 | 1.701 | 22.449 |
| Henan | Anyang | 2 | 59 | 1276 | 95 | N/A | 10 | 3 | 0.500 | 1.000 |  |  |  |  |  |  |  |
| Henan | Jiaozuo | 2 | 59 | 1276 | 83 | N/A | 10 | 3 | 0.500 | 0.667 |  |  |  |  |  |  |  |
| Henan | Sanmenxia | 2 | 58 | 1276 | 51 | 225 | 10 | 1 | 0.500 | 1.000 | 2.000 | 0.938 | 1.000 | 0.938 | 1.067 | 1.701 | 3.628 |
| Henan | Anyang | 2 | 58 | 1276 | 95 | 206 | 10 | 8 | 0.500 | 0.250 | 8.000 | 1.000 | 1.000 | 1.000 | 1.000 | 1.701 | 13.606 |
| Henan | Anyang | 2 | 58 | 1276 | 95 | 202 | 10 | 8 | 0.500 | 0.250 | 8.000 | 0.900 | 1.000 | 0.900 | 1.111 | 1.701 | 15.117 |
| Henan | Jiaozuo | 2 | 58 | 1276 | 83 | 208 | 10 | 9 | 0.500 | 0.222 | 9.000 | 1.000 | 1.000 | 1.000 | 1.000 | 1.701 | 15.306 |
| Henan | Luohe | 2 | 58 | 1276 | 45 | 221 | 10 | 10 | 0.500 | 0.200 | 10.000 | 1.000 | 1.000 | 1.000 | 1.000 | 1.701 | 17.007 |
| Henan | Luohe | 2 | 58 | 1276 | 45 | 218 | 10 | 10 | 0.500 | 0.200 | 10.000 | 1.000 | 1.000 | 1.000 | 1.000 | 1.701 | 17.007 |
| Henan | Xinxiang | 2 | 58 | 1276 | 118 | 229 | 10 | 10 | 0.500 | 0.200 | 10.000 | 1.000 | 1.000 | 1.000 | 1.000 | 1.701 | 17.007 |
| Henan | Jiaozuo | 2 | 58 | 1276 | 83 | 209 | 10 | 9 | 0.500 | 0.222 | 9.000 | 0.833 | 1.000 | 0.833 | 1.200 | 1.701 | 18.368 |
| Henan | Xinxiang | 2 | 58 | 1276 | 118 | 230 | 10 | 10 | 0.500 | 0.200 | 10.000 | 0.909 | 1.000 | 0.909 | 1.100 | 1.701 | 18.708 |
| Hebei | Baoding | 3 | 57 | 351 | 370 | 174 | 5 | 30 | 0.600 | 0.067 | 25.000 | 1.000 | 1.000 | 1.000 | 1.000 | 0.940 | 23.510 |
| Hebei | Baoding | 3 | 57 | 351 | 370 | 169 | 5 | 30 | 0.600 | 0.067 | 25.000 | 1.000 | 1.000 | 1.000 | 1.000 | 0.940 | 23.510 |
| Hebei | Cangzhou | 3 | 57 | 351 | 197 | 179 | 5 | 20 | 0.600 | 0.100 | 16.667 | 1.000 | 0.500 | 0.500 | 2.000 | 0.940 | 31.346 |
| Hebei | Handan | 3 | 57 | 351 | 265 | 186 | 5 | 20 | 0.600 | 0.100 | 16.667 | 1.000 | 0.500 | 0.500 | 2.000 | 0.940 | 31.346 |
| Hebei | Cangzhou | 3 | 57 | 351 | 197 | N/A | 5 | 20 | 0.600 | 0.100 |  |  |  |  |  |  |  |
| Hebei | Handan | 3 | 57 | 351 | 265 | N/A | 5 | 20 | 0.600 | 0.100 |  |  |  |  |  |  |  |
| Hebei | Cangzhou | 3 | 56 | 351 | 197 | 176 | 5 | 4 | 0.600 | 0.500 | 3.333 | 1.000 | 1.000 | 1.000 | 1.000 | 0.940 | 3.135 |
| Hebei | Cangzhou | 3 | 56 | 351 | 197 | 175 | 5 | 4 | 0.600 | 0.500 | 3.333 | 1.000 | 1.000 | 1.000 | 1.000 | 0.940 | 3.135 |
| Hebei | Handan | 3 | 56 | 351 | 265 | 189 | 5 | 5 | 0.600 | 0.400 | 4.167 | 1.000 | 1.000 | 1.000 | 1.000 | 0.940 | 3.918 |
| Hebei | Handan | 3 | 56 | 351 | 265 | 188 | 5 | 5 | 0.600 | 0.400 | 4.167 | 1.000 | 1.000 | 1.000 | 1.000 | 0.940 | 3.918 |
| Hebei | Baoding | 3 | 56 | 351 | 370 | 172 | 5 | 9 | 0.600 | 0.222 | 7.500 | 1.000 | 1.000 | 1.000 | 1.000 | 0.940 | 7.053 |
| Hebei | Baoding | 3 | 56 | 351 | 370 | 171 | 5 | 9 | 0.600 | 0.222 | 7.500 | 0.846 | 1.000 | 0.846 | 1.182 | 0.940 | 8.335 |
| Hebei | Cangzhou | 3 | 55 | 351 | 197 | 178 | 5 | 15 | 0.600 | 0.133 | 12.500 | 1.000 | 1.000 | 1.000 | 1.000 | 0.940 | 11.755 |
| Hebei | Cangzhou | 3 | 55 | 351 | 197 | 177 | 5 | 15 | 0.600 | 0.133 | 12.500 | 1.000 | 1.000 | 1.000 | 1.000 | 0.940 | 11.755 |
| Hebei | Handan | 3 | 55 | 351 | 265 | 190 | 5 | 15 | 0.600 | 0.133 | 12.500 | 1.000 | 1.000 | 1.000 | 1.000 | 0.940 | 11.755 |
| Hebei | Handan | 3 | 55 | 351 | 265 | 187 | 5 | 15 | 0.600 | 0.133 | 12.500 | 1.000 | 1.000 | 1.000 | 1.000 | 0.940 | 11.755 |
| Hebei | Baoding | 3 | 55 | 351 | 370 | 173 | 5 | 21 | 0.600 | 0.095 | 17.500 | 1.000 | 1.000 | 1.000 | 1.000 | 0.940 | 16.457 |
| Hebei | Baoding | 3 | 55 | 351 | 370 | 170 | 5 | 21 | 0.600 | 0.095 | 17.500 | 1.000 | 1.000 | 1.000 | 1.000 | 0.940 | 16.457 |
| Hebei | Chengde | 2 | 54 | 351 | 86 | 185 | 6 | 13 | 0.500 | 0.154 | 13.000 | 1.000 | 1.000 | 1.000 | 1.000 | 0.940 | 12.225 |
| Hebei | Xingtai | 2 | 54 | 351 | 204 | 200 | 6 | 12 | 0.500 | 0.167 | 12.000 | 0.833 | 1.000 | 0.833 | 1.200 | 0.940 | 13.542 |
| Hebei | Qinhuangdao | 2 | 54 | 351 | 67 | 191 | 6 | 14 | 0.500 | 0.143 | 14.000 | 0.909 | 1.000 | 0.909 | 1.100 | 0.940 | 14.482 |
| Hebei | Qinhuangdao | 2 | 54 | 351 | 67 | 193 | 6 | 14 | 0.500 | 0.143 | 14.000 | 0.800 | 1.000 | 0.800 | 1.250 | 0.940 | 16.457 |
| Hebei | Xingtai | 2 | 54 | 351 | 204 | 198 | 6 | 12 | 0.500 | 0.167 | 12.000 | 0.600 | 1.000 | 0.600 | 1.667 | 0.940 | 18.808 |
| Hebei | Chengde | 2 | 54 | 351 | 86 | 184 | 6 | 13 | 0.500 | 0.154 | 13.000 | 0.467 | 1.000 | 0.467 | 2.143 | 0.940 | 26.196 |
| Hebei | Xingtai | 2 | 53 | 351 | 204 | 201 | 6 | 1 | 0.500 | 1.000 | 2.000 | 1.000 | 1.000 | 1.000 | 1.000 | 0.940 | 1.881 |
| Hebei | Chengde | 2 | 53 | 351 | 86 | 183 | 6 | 3 | 0.500 | 0.667 | 3.000 | 1.000 | 1.000 | 1.000 | 1.000 | 0.940 | 2.821 |
| Hebei | Chengde | 2 | 53 | 351 | 86 | 181 | 6 | 3 | 0.500 | 0.667 | 3.000 | 1.000 | 1.000 | 1.000 | 1.000 | 0.940 | 2.821 |
| Hebei | Qinhuangdao | 2 | 53 | 351 | 67 | 194 | 6 | 3 | 0.500 | 0.667 | 3.000 | 1.000 | 1.000 | 1.000 | 1.000 | 0.940 | 2.821 |
| Hebei | Qinhuangdao | 2 | 53 | 351 | 67 | 195 | 6 | 3 | 0.500 | 0.667 | 3.000 | 0.972 | 1.000 | 0.972 | 1.029 | 0.940 | 2.902 |
| Hebei | Qinhuangdao | 2 | 52 | 351 | 67 | 196 | 6 | 8 | 0.500 | 0.250 | 8.000 | 1.000 | 1.000 | 1.000 | 1.000 | 0.940 | 7.523 |
| Hebei | Xingtai | 2 | 52 | 351 | 204 | 199 | 6 | 8 | 0.500 | 0.250 | 8.000 | 1.000 | 1.000 | 1.000 | 1.000 | 0.940 | 7.523 |
| Hebei | Xingtai | 2 | 52 | 351 | 204 | 197 | 6 | 8 | 0.500 | 0.250 | 8.000 | 1.000 | 1.000 | 1.000 | 1.000 | 0.940 | 7.523 |
| Hebei | Qinhuangdao | 2 | 52 | 351 | 67 | 192 | 6 | 8 | 0.500 | 0.250 | 8.000 | 0.889 | 1.000 | 0.889 | 1.125 | 0.940 | 8.463 |
| Hebei | Chengde | 2 | 52 | 351 | 86 | 180 | 6 | 10 | 0.500 | 0.200 | 10.000 | 1.000 | 1.000 | 1.000 | 1.000 | 0.940 | 9.404 |
| Hebei | Chengde | 2 | 52 | 351 | 86 | 182 | 6 | 10 | 0.500 | 0.200 | 10.000 | 0.769 | 1.000 | 0.769 | 1.300 | 0.940 | 12.225 |
| Hainan | Haikou | 2 | 51 | 171 | 47 | N/A | 1 | 8 | 1.000 | 0.250 |  |  |  |  |  |  |  |
| Hainan | Haikou | 2 | 51 | 171 | 47 | N/A | 1 | 8 | 1.000 | 0.250 |  |  |  |  |  |  |  |
| Hainan | Haikou | 2 | 50 | 171 | 47 | 163 | 1 | 4 | 1.000 | 0.500 | 2.000 | 1.000 | 1.000 | 1.000 | 1.000 | 0.548 | 1.096 |
| Hainan | Haikou | 2 | 50 | 171 | 47 | 162 | 1 | 4 | 1.000 | 0.500 | 2.000 | 0.967 | 1.000 | 0.967 | 1.034 | 0.548 | 1.134 |
| Hainan | Haikou | 2 | 49 | 171 | 47 | 161 | 1 | 6 | 1.000 | 0.333 | 3.000 | 0.789 | 1.000 | 0.789 | 1.267 | 0.548 | 2.083 |
| Hainan | Haikou | 2 | 49 | 171 | 47 | N/A | 1 | 6 | 1.000 | 0.333 |  |  |  |  |  |  |  |
| Hainan | Sanya | 1 | 48 | 171 | 21 | 166 | 3 | 4 | 0.667 | 0.750 | 2.000 | 1.000 | 1.000 | 1.000 | 1.000 | 0.548 | 1.096 |
| Hainan | Sanya | 1 | 48 | 171 | 21 | 165 | 3 | 4 | 0.667 | 0.750 | 2.000 | 1.000 | 1.000 | 1.000 | 1.000 | 0.548 | 1.096 |
| Hainan | Danzhou | 1 | 48 | 171 | 19 | 160 | 3 | 8 | 0.667 | 0.250 | 6.000 | 1.000 | 1.000 | 1.000 | 1.000 | 0.548 | 3.289 |
| Hainan | Danzhou | 1 | 48 | 171 | 19 | N/A | 3 | 8 | 0.667 | 0.250 |  |  |  |  |  |  |  |
| Hainan | Sanya | 1 | 48 | 171 | 21 | N/A | 3 | 4 | 0.667 | 0.750 |  |  |  |  |  |  |  |
| Hainan | Sanya | 1 | 47 | 171 | 21 | 164 | 3 | 1 | 0.667 | 1.000 | 1.500 | 0.909 | 1.000 | 0.909 | 1.100 | 0.548 | 0.905 |
| Hainan | Sanya | 1 | 46 | 171 | 21 | 168 | 3 | 5 | 0.667 | 0.400 | 3.750 | 1.000 | 1.000 | 1.000 | 1.000 | 0.548 | 2.056 |
| Hainan | Sanya | 1 | 46 | 171 | 21 | 167 | 3 | 5 | 0.667 | 0.400 | 3.750 | 1.000 | 1.000 | 1.000 | 1.000 | 0.548 | 2.056 |
| Hainan | Danzhou | 1 | 46 | 171 | 19 | 159 | 3 | 8 | 0.667 | 0.250 | 6.000 | 1.000 | 0.500 | 0.500 | 2.000 | 0.548 | 6.579 |
| Hainan | Danzhou | 1 | 46 | 171 | 19 | N/A | 3 | 8 | 0.667 | 0.250 |  |  |  |  |  |  |  |
| Guizhou | Bijie | 3 | 45 | 147 | 514 | 135 | 2 | 20 | 1.000 | 0.100 | 10.000 | 1.000 | 1.000 | 1.000 | 1.000 | 0.418 | 4.175 |
| Guizhou | Guiyang | 3 | 45 | 147 | 192 | 142 | 2 | 20 | 1.000 | 0.100 | 10.000 | 1.000 | 1.000 | 1.000 | 1.000 | 0.418 | 4.175 |
| Guizhou | Guiyang | 3 | 45 | 147 | 192 | 141 | 2 | 20 | 1.000 | 0.100 | 10.000 | 1.000 | 1.000 | 1.000 | 1.000 | 0.418 | 4.175 |
| Guizhou | Bijie | 3 | 45 | 147 | 514 | 136 | 2 | 20 | 1.000 | 0.100 | 10.000 | 0.909 | 1.000 | 0.909 | 1.100 | 0.418 | 4.593 |
| Guizhou | Bijie | 3 | 44 | 147 | 514 | 134 | 2 | 4 | 1.000 | 0.500 | 2.000 | 1.000 | 1.000 | 1.000 | 1.000 | 0.418 | 0.835 |
| Guizhou | Bijie | 3 | 44 | 147 | 514 | 131 | 2 | 4 | 1.000 | 0.500 | 2.000 | 1.000 | 1.000 | 1.000 | 1.000 | 0.418 | 0.835 |
| Guizhou | Guiyang | 3 | 44 | 147 | 192 | 139 | 2 | 15 | 1.000 | 0.133 | 7.500 | 1.000 | 1.000 | 1.000 | 1.000 | 0.418 | 3.132 |
| Guizhou | Guiyang | 3 | 44 | 147 | 192 | 137 | 2 | 15 | 1.000 | 0.133 | 7.500 | 1.000 | 1.000 | 1.000 | 1.000 | 0.418 | 3.132 |
| Guizhou | Bijie | 3 | 43 | 147 | 514 | 133 | 2 | 15 | 1.000 | 0.133 | 7.500 | 1.000 | 1.000 | 1.000 | 1.000 | 0.418 | 3.132 |
| Guizhou | Bijie | 3 | 43 | 147 | 514 | 132 | 2 | 15 | 1.000 | 0.133 | 7.500 | 0.909 | 1.000 | 0.909 | 1.100 | 0.418 | 3.445 |
| Guizhou | Guiyang | 3 | 43 | 147 | 192 | 140 | 2 | 30 | 1.000 | 0.067 | 15.000 | 1.000 | 1.000 | 1.000 | 1.000 | 0.418 | 6.263 |
| Guizhou | Guiyang | 3 | 43 | 147 | 192 | 138 | 2 | 30 | 1.000 | 0.067 | 15.000 | 1.000 | 1.000 | 1.000 | 1.000 | 0.418 | 6.263 |
| Guizhou | Anshun | 2 | 42 | 147 | 74 | 130 | 3 | 15 | 1.000 | 0.133 | 7.500 | 1.000 | 1.000 | 1.000 | 1.000 | 0.418 | 3.132 |
| Guizhou | Anshun | 2 | 42 | 147 | 74 | 129 | 3 | 15 | 1.000 | 0.133 | 7.500 | 0.833 | 1.000 | 0.833 | 1.200 | 0.418 | 3.758 |
| Guizhou | Tongren | 2 | 42 | 147 | 107 | 151 | 3 | 19 | 1.000 | 0.105 | 9.500 | 1.000 | 1.000 | 1.000 | 1.000 | 0.418 | 3.967 |
| Guizhou | Liupanshui | 2 | 42 | 147 | 114 | 147 | 3 | 20 | 1.000 | 0.100 | 10.000 | 1.000 | 1.000 | 1.000 | 1.000 | 0.418 | 4.175 |
| Guizhou | Liupanshui | 2 | 42 | 147 | 114 | 146 | 3 | 20 | 1.000 | 0.100 | 10.000 | 1.000 | 1.000 | 1.000 | 1.000 | 0.418 | 4.175 |
| Guizhou | Tongren | 2 | 42 | 147 | 107 | 153 | 3 | 19 | 1.000 | 0.105 | 9.500 | 0.778 | 1.000 | 0.778 | 1.286 | 0.418 | 5.100 |
| Guizhou | Liupanshui | 2 | 41 | 147 | 114 | 144 | 3 | 5 | 1.000 | 0.400 | 2.500 | 1.000 | 1.000 | 1.000 | 1.000 | 0.418 | 1.044 |
| Guizhou | Liupanshui | 2 | 41 | 147 | 114 | 143 | 3 | 5 | 1.000 | 0.400 | 2.500 | 1.000 | 1.000 | 1.000 | 1.000 | 0.418 | 1.044 |
| Guizhou | Anshun | 2 | 41 | 147 | 74 | 128 | 3 | 3 | 1.000 | 0.667 | 1.500 | 1.000 | 0.500 | 0.500 | 2.000 | 0.418 | 1.253 |
| Guizhou | Tongren | 2 | 41 | 147 | 107 | 150 | 3 | 4 | 1.000 | 0.250 | 4.000 | 1.000 | 1.000 | 1.000 | 1.000 | 0.418 | 1.670 |
| Guizhou | Anshun | 2 | 41 | 147 | 74 | N/A | 3 | 3 | 1.000 | 0.667 |  |  |  |  |  |  |  |
| Guizhou | Anshun | 2 | 40 | 147 | 74 | 127 | 3 | 10 | 1.000 | 0.200 | 5.000 | 1.000 | 1.000 | 1.000 | 1.000 | 0.418 | 2.088 |
| Guizhou | Tongren | 2 | 40 | 147 | 107 | 152 | 3 | 13 | 1.000 | 0.154 | 6.500 | 1.000 | 1.000 | 1.000 | 1.000 | 0.418 | 2.714 |
| Guizhou | Tongren | 2 | 40 | 147 | 107 | 149 | 3 | 13 | 1.000 | 0.154 | 6.500 | 1.000 | 1.000 | 1.000 | 1.000 | 0.418 | 2.714 |
| Guizhou | Liupanshui | 2 | 40 | 147 | 114 | 148 | 3 | 15 | 1.000 | 0.133 | 7.500 | 1.000 | 1.000 | 1.000 | 1.000 | 0.418 | 3.132 |
| Guizhou | Liupanshui | 2 | 40 | 147 | 114 | 145 | 3 | 15 | 1.000 | 0.133 | 7.500 | 1.000 | 1.000 | 1.000 | 1.000 | 0.418 | 3.132 |
| Guizhou | Anshun | 2 | 40 | 147 | 74 | N/A | 3 | 10 | 1.000 | 0.200 |  |  |  |  |  |  |  |
| Guizhou | Zunyi | 1 | 39 | 147 |  | 157 | 1 | 15 | 1.000 | 0.133 | 7.500 | 1.000 | 1.000 | 1.000 | 1.000 | 0.418 | 3.132 |
| Guizhou | Zunyi | 1 | 39 | 147 |  | 155 | 1 | 15 | 1.000 | 0.133 | 7.500 | 1.000 | 1.000 | 1.000 | 1.000 | 0.418 | 3.132 |
| Guizhou | Zunyi | 1 | 38 | 147 |  | 158 | 1 | 4 | 1.000 | 0.500 | 2.000 | 1.000 | 1.000 | 1.000 | 1.000 | 0.418 | 0.835 |
| Guizhou | Zunyi | 1 | 38 | 147 |  | N/A | 1 | 4 | 1.000 | 0.500 |  |  |  |  |  |  |  |
| Guizhou | Zunyi | 1 | 37 | 147 |  | 156 | 1 | 8 | 1.000 | 0.250 | 4.000 | 1.000 | 1.000 | 1.000 | 1.000 | 0.418 | 1.670 |
| Guizhou | Zunyi | 1 | 37 | 147 |  | 154 | 1 | 8 | 1.000 | 0.250 | 4.000 | 1.000 | 1.000 | 1.000 | 1.000 | 0.418 | 1.670 |
| Guangxi | Baise | 2 | 36 | 355 | 36 | 111 | 7 | 5 | 0.571 | 0.400 | 4.375 | 1.000 | 0.500 | 0.500 | 2.000 | 1.191 | 10.420 |
| Guangxi | Wuzhou | 2 | 36 | 355 | 44 | 122 | 7 | 6 | 0.571 | 0.333 | 5.250 | 0.500 | 1.000 | 0.500 | 2.000 | 1.191 | 12.504 |
| Guangxi | Baise | 2 | 36 | 355 | 36 | N/A | 7 | 5 | 0.571 | 0.400 |  |  |  |  |  |  |  |
| Guangxi | Liuzhou | 2 | 36 | 355 | 64 | N/A | 7 | 14 | 0.571 | 0.143 |  |  |  |  |  |  |  |
| Guangxi | Liuzhou | 2 | 36 | 355 | 64 | N/A | 7 | 14 | 0.571 | 0.143 |  |  |  |  |  |  |  |
| Guangxi | Wuzhou | 2 | 36 | 355 | 44 | N/A | 7 | 6 | 0.571 | 0.333 |  |  |  |  |  |  |  |
| Guangxi | Yulin | 2 | 36 | 355 | 51 | N/A | 7 | 10 | 0.571 | 0.200 |  |  |  |  |  |  |  |
| Guangxi | Yulin | 2 | 36 | 355 | 51 | N/A | 7 | 10 | 0.571 | 0.200 |  |  |  |  |  |  |  |
| Guangxi | Baise | 2 | 35 | 355 | 36 | 109 | 7 | 2 | 0.571 | 1.000 | 1.750 | 0.875 | 1.000 | 0.875 | 1.143 | 1.191 | 2.382 |
| Guangxi | Baise | 2 | 35 | 355 | 36 | 112 | 7 | 2 | 0.571 | 1.000 | 1.750 | 0.556 | 1.000 | 0.556 | 1.800 | 1.191 | 3.751 |
| Guangxi | Wuzhou | 2 | 35 | 355 | 44 | 123 | 7 | 4 | 0.571 | 0.500 | 3.500 | 1.000 | 1.000 | 1.000 | 1.000 | 1.191 | 4.168 |
| Guangxi | Liuzhou | 2 | 35 | 355 | 64 | 120 | 7 | 6 | 0.571 | 0.333 | 5.250 | 1.000 | 1.000 | 1.000 | 1.000 | 1.191 | 6.252 |
| Guangxi | Wuzhou | 2 | 35 | 355 | 44 | 124 | 7 | 4 | 0.571 | 0.500 | 3.500 | 0.583 | 1.000 | 0.583 | 1.714 | 1.191 | 7.145 |
| Guangxi | Liuzhou | 2 | 35 | 355 | 64 | N/A | 7 | 6 | 0.571 | 0.333 |  |  |  |  |  |  |  |
| Guangxi | Yulin | 2 | 35 | 355 | 51 | N/A | 7 | 1 | 0.571 | 1.000 |  |  |  |  |  |  |  |
| Guangxi | Baise | 2 | 34 | 355 | 36 | 110 | 7 | 5 | 0.571 | 0.400 | 4.375 | 1.000 | 1.000 | 1.000 | 1.000 | 1.191 | 5.210 |
| Guangxi | Baise | 2 | 34 | 355 | 36 | 108 | 7 | 5 | 0.571 | 0.400 | 4.375 | 1.000 | 1.000 | 1.000 | 1.000 | 1.191 | 5.210 |
| Guangxi | Yulin | 2 | 34 | 355 | 51 | 125 | 7 | 8 | 0.571 | 0.375 | 4.667 | 0.571 | 0.667 | 0.381 | 2.625 | 1.191 | 14.588 |
| Guangxi | Yulin | 2 | 34 | 355 | 51 | 126 | 7 | 8 | 0.571 | 0.375 | 4.667 | 0.529 | 0.667 | 0.353 | 2.833 | 1.191 | 15.746 |
| Guangxi | Wuzhou | 2 | 34 | 355 | 44 | 121 | 7 | 8 | 0.571 | 0.250 | 7.000 | 0.400 | 1.000 | 0.400 | 2.500 | 1.191 | 20.840 |
| Guangxi | Liuzhou | 2 | 34 | 355 | 64 | N/A | 7 | 7 | 0.571 | 0.286 |  |  |  |  |  |  |  |
| Guangxi | Liuzhou | 2 | 34 | 355 | 64 | N/A | 7 | 7 | 0.571 | 0.286 |  |  |  |  |  |  |  |
| Guangxi | Wuzhou | 2 | 34 | 355 | 44 | N/A | 7 | 8 | 0.571 | 0.250 |  |  |  |  |  |  |  |
| Guangxi | Yulin | 2 | 34 | 355 | 51 | N/A | 7 | 8 | 0.571 | 0.375 |  |  |  |  |  |  |  |
| Guangxi | Chongzuo | 1 | 33 | 355 | 29 | 119 | 6 | 10 | 0.333 | 0.200 | 15.000 | 0.778 | 0.500 | 0.389 | 2.571 | 1.191 | 45.933 |
| Guangxi | Beihai | 1 | 33 | 355 | 27 | N/A | 6 | 17 | 0.333 | 0.118 |  |  |  |  |  |  |  |
| Guangxi | Beihai | 1 | 33 | 355 | 27 | N/A | 6 | 17 | 0.333 | 0.118 |  |  |  |  |  |  |  |
| Guangxi | Chongzuo | 1 | 33 | 355 | 29 | N/A | 6 | 10 | 0.333 | 0.200 |  |  |  |  |  |  |  |
| Guangxi | Beihai | 1 | 32 | 355 | 27 | 116 | 6 | 4 | 0.333 | 0.500 | 6.000 | 1.000 | 1.000 | 1.000 | 1.000 | 1.191 | 7.145 |
| Guangxi | Beihai | 1 | 32 | 355 | 27 | 115 | 6 | 4 | 0.333 | 0.500 | 6.000 | 1.000 | 1.000 | 1.000 | 1.000 | 1.191 | 7.145 |
| Guangxi | Beihai | 1 | 31 | 355 | 27 | 114 | 6 | 6 | 0.333 | 0.333 | 9.000 | 1.000 | 1.000 | 1.000 | 1.000 | 1.191 | 10.718 |
| Guangxi | Beihai | 1 | 31 | 355 | 27 | 113 | 6 | 6 | 0.333 | 0.333 | 9.000 | 1.000 | 1.000 | 1.000 | 1.000 | 1.191 | 10.718 |
| Guangxi | Chongzuo | 1 | 31 | 355 | 29 | 118 | 6 | 6 | 0.333 | 0.333 | 9.000 | 1.000 | 1.000 | 1.000 | 1.000 | 1.191 | 10.718 |
| Guangxi | Chongzuo | 1 | 31 | 355 | 29 | 117 | 6 | 6 | 0.333 | 0.333 | 9.000 | 0.600 | 1.000 | 0.600 | 1.667 | 1.191 | 17.863 |
| Guangdong | Guangzhou | 3 | 30 | 1721 | 255 | 90 | 4 | 62 | 0.250 | 0.016 | 248.000 | 0.667 | 1.000 | 0.667 | 1.500 | 1.834 | 682.303 |
| Guangdong | Guangzhou | 3 | 29 | 1721 | 255 | 92 | 4 | 45 | 0.250 | 0.044 | 90.000 | 1.000 | 1.000 | 1.000 | 1.000 | 1.834 | 165.073 |
| Guangdong | Guangzhou | 3 | 29 | 1721 | 255 | 89 | 4 | 45 | 0.250 | 0.044 | 90.000 | 1.000 | 1.000 | 1.000 | 1.000 | 1.834 | 165.073 |
| Guangdong | Guangzhou | 3 | 28 | 1721 | 255 | 91 | 4 | 43 | 0.250 | 0.070 | 57.333 | 1.000 | 0.333 | 0.333 | 3.000 | 1.834 | 315.473 |
| Guangdong | Guangzhou | 3 | 28 | 1721 | 255 | N/A | 4 | 43 | 0.250 | 0.070 |  |  |  |  |  |  |  |
| Guangdong | Guangzhou | 3 | 28 | 1721 | 255 | N/A | 4 | 43 | 0.250 | 0.070 |  |  |  |  |  |  |  |
| Guangdong | Ji eyang | 2 | 27 | 1721 | 62 | 94 | 12 | 13 | 0.333 | 0.231 | 13.000 | 0.750 | 0.667 | 0.500 | 2.000 | 1.834 | 47.688 |
| Guangdong | Yangjiang | 2 | 27 | 1721 | 57 | 102 | 12 | 11 | 0.333 | 0.182 | 16.500 | 1.000 | 0.500 | 0.500 | 2.000 | 1.834 | 60.527 |
| Guangdong | Ji eyang | 2 | 27 | 1721 | 62 | 95 | 12 | 13 | 0.333 | 0.231 | 13.000 | 0.500 | 0.667 | 0.333 | 3.000 | 1.834 | 71.532 |
| Guangdong | Shaoguan | 2 | 27 | 1721 | 54 | 98 | 12 | 7 | 0.333 | 0.286 | 10.500 | 0.231 | 0.500 | 0.115 | 8.667 | 1.834 | 166.907 |
| Guangdong | Ji eyang | 2 | 27 | 1721 | 62 | N/A | 12 | 13 | 0.333 | 0.231 |  |  |  |  |  |  |  |
| Guangdong | Shaoguan | 2 | 27 | 1721 | 54 | N/A | 12 | 7 | 0.333 | 0.286 |  |  |  |  |  |  |  |
| Guangdong | Yangjiang | 2 | 27 | 1721 | 57 | N/A | 12 | 11 | 0.333 | 0.182 |  |  |  |  |  |  |  |
| Guangdong | Zhaoqing | 2 | 27 | 1721 | 56 | N/A | 12 | 6 | 0.333 | 0.333 |  |  |  |  |  |  |  |
| Guangdong | Zhaoqing | 2 | 27 | 1721 | 56 | N/A | 12 | 6 | 0.333 | 0.333 |  |  |  |  |  |  |  |
| Guangdong | Ji eyang | 2 | 26 | 1721 | 62 | 96 | 12 | 1 | 0.333 | 1.000 | 3.000 | 1.000 | 1.000 | 1.000 | 1.000 | 1.834 | 5.502 |
| Guangdong | Yangjiang | 2 | 26 | 1721 | 57 | 103 | 12 | 1 | 0.333 | 1.000 | 3.000 | 1.000 | 1.000 | 1.000 | 1.000 | 1.834 | 5.502 |
| Guangdong | Shaoguan | 2 | 26 | 1721 | 54 | 100 | 12 | 4 | 0.333 | 0.500 | 6.000 | 1.000 | 1.000 | 1.000 | 1.000 | 1.834 | 11.005 |
| Guangdong | Shaoguan | 2 | 26 | 1721 | 54 | 99 | 12 | 4 | 0.333 | 0.500 | 6.000 | 1.000 | 1.000 | 1.000 | 1.000 | 1.834 | 11.005 |
| Guangdong | Zhaoqing | 2 | 26 | 1721 | 56 | 104 | 12 | 3 | 0.333 | 0.667 | 4.500 | 1.000 | 0.500 | 0.500 | 2.000 | 1.834 | 16.507 |
| Guangdong | Zhaoqing | 2 | 26 | 1721 | 56 | N/A | 12 | 3 | 0.333 | 0.667 |  |  |  |  |  |  |  |
| Guangdong | Shaoguan | 2 | 25 | 1721 | 54 | 101 | 12 | 6 | 0.333 | 0.333 | 9.000 | 1.000 | 1.000 | 1.000 | 1.000 | 1.834 | 16.507 |
| Guangdong | Shaoguan | 2 | 25 | 1721 | 54 | 97 | 12 | 6 | 0.333 | 0.333 | 9.000 | 1.000 | 1.000 | 1.000 | 1.000 | 1.834 | 16.507 |
| Guangdong | Ji eyang | 2 | 25 | 1721 | 62 | 93 | 12 | 6 | 0.333 | 0.167 | 18.000 | 1.000 | 1.000 | 1.000 | 1.000 | 1.834 | 33.015 |
| Guangdong | Yangjiang | 2 | 25 | 1721 | 57 | N/A | 12 | 6 | 0.333 | 0.333 |  |  |  |  |  |  |  |
| Guangdong | Yangjiang | 2 | 25 | 1721 | 57 | N/A | 12 | 6 | 0.333 | 0.333 |  |  |  |  |  |  |  |
| Guangdong | Zhaoqing | 2 | 25 | 1721 | 56 | N/A | 12 | 8 | 0.333 | 0.250 |  |  |  |  |  |  |  |
| Guangdong | Zhaoqing | 2 | 25 | 1721 | 56 | N/A | 12 | 8 | 0.333 | 0.250 |  |  |  |  |  |  |  |
| Guangdong | Zhuhai | 1 | 24 | 1721 | 45 | 107 | 5 | 15 | 0.200 | 0.133 | 37.500 | 0.833 | 1.000 | 0.833 | 1.200 | 1.834 | 82.537 |
| Guangdong | Zhuhai | 1 | 24 | 1721 | 45 | N/A | 5 | 15 | 0.200 | 0.133 |  |  |  |  |  |  |  |
| Guangdong | Zhuhai | 1 | 23 | 1721 | 45 | 106 | 5 | 8 | 0.200 | 0.250 | 20.000 | 1.000 | 1.000 | 1.000 | 1.000 | 1.834 | 36.683 |
| Guangdong | Zhuhai | 1 | 23 | 1721 | 45 | 105 | 5 | 8 | 0.200 | 0.250 | 20.000 | 1.000 | 1.000 | 1.000 | 1.000 | 1.834 | 36.683 |
| Guangdong | Zhuhai | 1 | 22 | 1721 | 45 | N/A | 5 | 12 | 0.200 | 0.167 |  |  |  |  |  |  |  |
| Guangdong | Zhuhai | 1 | 22 | 1721 | 45 | N/A | 5 | 12 | 0.200 | 0.167 |  |  |  |  |  |  |  |
| Gansu | Tianshui | 2 | 21 | 169 | 77 | 80 | 4 | 9 | 0.500 | 0.222 | 9.000 | 0.800 | 0.500 | 0.400 | 2.500 | 1.043 | 23.474 |
| Gansu | Lanzhou | 2 | 21 | 169 | 125 | 71 | 4 | 14 | 0.500 | 0.143 | 14.000 | 0.600 | 1.000 | 0.600 | 1.667 | 1.043 | 24.343 |
| Gansu | Lanzhou | 2 | 21 | 169 | 125 | N/A | 4 | 14 | 0.500 | 0.143 |  |  |  |  |  |  |  |
| Gansu | Tianshui | 2 | 21 | 169 | 77 | N/A | 4 | 9 | 0.500 | 0.222 |  |  |  |  |  |  |  |
| Gansu | Tianshui | 2 | 20 | 169 | 77 | 77 | 4 | 3 | 0.500 | 0.667 | 3.000 | 1.000 | 0.500 | 0.500 | 2.000 | 1.043 | 6.260 |
| Gansu | Lanzhou | 2 | 20 | 169 | 125 | 70 | 4 | 12 | 0.500 | 0.167 | 12.000 | 0.714 | 1.000 | 0.714 | 1.400 | 1.043 | 17.527 |
| Gansu | Lanzhou | 2 | 20 | 169 | 125 | 69 | 4 | 12 | 0.500 | 0.167 | 12.000 | 0.278 | 1.000 | 0.278 | 3.600 | 1.043 | 45.070 |
| Gansu | Tianshui | 2 | 20 | 169 | 77 | N/A | 4 | 3 | 0.500 | 0.667 |  |  |  |  |  |  |  |
| Gansu | Tianshui | 2 | 19 | 169 | 77 | 79 | 4 | 6 | 0.500 | 0.333 | 6.000 | 1.000 | 1.000 | 1.000 | 1.000 | 1.043 | 6.260 |
| Gansu | Tianshui | 2 | 19 | 169 | 77 | 78 | 4 | 6 | 0.500 | 0.333 | 6.000 | 0.938 | 1.000 | 0.938 | 1.067 | 1.043 | 6.677 |
| Gansu | Lanzhou | 2 | 19 | 169 | 125 | N/A | 4 | 17 | 0.500 | 0.118 |  |  |  |  |  |  |  |
| Gansu | Lanzhou | 2 | 19 | 169 | 125 | N/A | 4 | 17 | 0.500 | 0.118 |  |  |  |  |  |  |  |
| Gansu | Qingyang | 1 | 18 | 169 | 44 | 76 | 8 | 7 | 0.500 | 0.286 | 7.000 | 1.000 | 1.000 | 1.000 | 1.000 | 1.043 | 7.303 |
| Gansu | Qingyang | 1 | 18 | 169 | 44 | 75 | 8 | 7 | 0.500 | 0.286 | 7.000 | 1.000 | 1.000 | 1.000 | 1.000 | 1.043 | 7.303 |
| Gansu | Zhangye | 1 | 18 | 169 | 45 | 86 | 8 | 10 | 0.500 | 0.200 | 10.000 | 0.714 | 1.000 | 0.714 | 1.400 | 1.043 | 14.606 |
| Gansu | Wuwei | 1 | 18 | 169 | 13 | 82 | 8 | 9 | 0.500 | 0.222 | 9.000 | 0.857 | 0.500 | 0.429 | 2.333 | 1.043 | 21.909 |
| Gansu | Baiyin | 1 | 18 | 169 | 99 | 66 | 8 | 14 | 0.500 | 0.143 | 14.000 | 1.000 | 0.500 | 0.500 | 2.000 | 1.043 | 29.212 |
| Gansu | Zhangye | 1 | 18 | 169 | 45 | 87 | 8 | 10 | 0.500 | 0.200 | 10.000 | 0.188 | 1.000 | 0.188 | 5.333 | 1.043 | 55.641 |
| Gansu | Baiyin | 1 | 18 | 169 | 99 | N/A | 8 | 14 | 0.500 | 0.143 |  |  |  |  |  |  |  |
| Gansu | Wuwei | 1 | 18 | 169 | 13 | N/A | 8 | 9 | 0.500 | 0.222 |  |  |  |  |  |  |  |
| Gansu | Wuwei | 1 | 17 | 169 | 13 | 84 | 8 | 2 | 0.500 | 1.000 | 2.000 | 1.000 | 1.000 | 1.000 | 1.000 | 1.043 | 2.087 |
| Gansu | Wuwei | 1 | 17 | 169 | 13 | 83 | 8 | 2 | 0.500 | 1.000 | 2.000 | 1.000 | 1.000 | 1.000 | 1.000 | 1.043 | 2.087 |
| Gansu | Qingyang | 1 | 17 | 169 | 44 | 73 | 8 | 2 | 0.500 | 1.000 | 2.000 | 1.000 | 0.500 | 0.500 | 2.000 | 1.043 | 4.173 |
| Gansu | Zhangye | 1 | 17 | 169 | 45 | 88 | 8 | 1 | 0.500 | 1.000 | 2.000 | 0.400 | 1.000 | 0.400 | 2.500 | 1.043 | 5.216 |
| Gansu | Baiyin | 1 | 17 | 169 | 99 | 67 | 8 | 4 | 0.500 | 0.500 | 4.000 | 1.000 | 0.500 | 0.500 | 2.000 | 1.043 | 8.346 |
| Gansu | Baiyin | 1 | 17 | 169 | 99 | N/A | 8 | 4 | 0.500 | 0.500 |  |  |  |  |  |  |  |
| Gansu | Qingyang | 1 | 17 | 169 | 44 | N/A | 8 | 2 | 0.500 | 1.000 |  |  |  |  |  |  |  |
| Gansu | Wuwei | 1 | 16 | 169 | 13 | 81 | 8 | 4 | 0.500 | 0.500 | 4.000 | 0.857 | 1.000 | 0.857 | 1.167 | 1.043 | 4.869 |
| Gansu | Qingyang | 1 | 16 | 169 | 44 | 72 | 8 | 4 | 0.500 | 0.500 | 4.000 | 0.833 | 1.000 | 0.833 | 1.200 | 1.043 | 5.008 |
| Gansu | Qingyang | 1 | 16 | 169 | 44 | 74 | 8 | 4 | 0.500 | 0.500 | 4.000 | 0.800 | 1.000 | 0.800 | 1.250 | 1.043 | 5.216 |
| Gansu | Zhangye | 1 | 16 | 169 | 45 | 85 | 8 | 3 | 0.500 | 0.667 | 3.000 | 0.375 | 0.500 | 0.188 | 5.333 | 1.043 | 16.692 |
| Gansu | Baiyin | 1 | 16 | 169 | 99 | 68 | 8 | 12 | 0.500 | 0.167 | 12.000 | 1.000 | 0.500 | 0.500 | 2.000 | 1.043 | 25.039 |
| Gansu | Baiyin | 1 | 16 | 169 | 99 | N/A | 8 | 12 | 0.500 | 0.167 |  |  |  |  |  |  |  |
| Gansu | Wuwei | 1 | 16 | 169 | 13 | N/A | 8 | 4 | 0.500 | 0.500 |  |  |  |  |  |  |  |
| Gansu | Zhangye | 1 | 16 | 169 | 45 | N/A | 8 | 3 | 0.500 | 0.667 |  |  |  |  |  |  |  |
| Fujian | Putian | 2 | 15 | 370 | 49 | 55 | 8 | 9 | 0.625 | 0.333 | 4.800 | 1.000 | 1.000 | 1.000 | 1.000 | 1.265 | 6.073 |
| Fujian | Putian | 2 | 15 | 370 | 49 | 52 | 8 | 9 | 0.625 | 0.333 | 4.800 | 1.000 | 1.000 | 1.000 | 1.000 | 1.265 | 6.073 |
| Fujian | Putian | 2 | 15 | 370 | 49 | 56 | 8 | 9 | 0.625 | 0.333 | 4.800 | 1.000 | 1.000 | 1.000 | 1.000 | 1.265 | 6.073 |
| Fujian | Sanming | 2 | 15 | 370 | 53 | 62 | 8 | 9 | 0.625 | 0.333 | 4.800 | 1.000 | 1.000 | 1.000 | 1.000 | 1.265 | 6.073 |
| Fujian | Sanming | 2 | 15 | 370 | 53 | 61 | 8 | 9 | 0.625 | 0.333 | 4.800 | 0.667 | 1.000 | 0.667 | 1.500 | 1.265 | 9.110 |
| Fujian | Zhangzhou | 2 | 15 | 370 | 82 | 65 | 8 | 9 | 0.625 | 0.222 | 7.200 | 0.833 | 0.500 | 0.417 | 2.400 | 1.265 | 21.864 |
| Fujian | Sanming | 2 | 15 | 370 | 53 | 57 | 8 | 9 | 0.625 | 0.333 | 4.800 | 0.167 | 1.000 | 0.167 | 6.000 | 1.265 | 36.441 |
| Fujian | Nanping | 2 | 15 | 370 | 53 | 48 | 8 | 8 | 0.625 | 0.250 | 6.400 | 0.333 | 0.500 | 0.167 | 6.000 | 1.265 | 48.588 |
| Fujian | #N/A | 2 | 15 | 370 |  | N/A | 8 | 8 | 0.625 | 0.250 |  |  |  |  |  |  |  |
| Fujian | #N/A | 2 | 15 | 370 |  | N/A | 8 | 8 | 0.625 | 0.250 |  |  |  |  |  |  |  |
| Fujian | Nanping | 2 | 15 | 370 | 53 | N/A | 8 | 8 | 0.625 | 0.250 |  |  |  |  |  |  |  |
| Fujian | Ningde | 2 | 15 | 370 | 46 | N/A | 8 | 5 | 0.625 | 0.400 |  |  |  |  |  |  |  |
| Fujian | Ningde | 2 | 15 | 370 | 46 | N/A | 8 | 5 | 0.625 | 0.400 |  |  |  |  |  |  |  |
| Fujian | Zhangzhou | 2 | 15 | 370 | 82 | N/A | 8 | 9 | 0.625 | 0.222 |  |  |  |  |  |  |  |
| Fujian | Putian | 2 | 14 | 370 | 49 | 54 | 8 | 2 | 0.625 | 1.000 | 1.600 | 1.000 | 1.000 | 1.000 | 1.000 | 1.265 | 2.024 |
| Fujian | Putian | 2 | 14 | 370 | 49 | 53 | 8 | 2 | 0.625 | 1.000 | 1.600 | 0.981 | 1.000 | 0.981 | 1.020 | 1.265 | 2.064 |
| Fujian | Sanming | 2 | 14 | 370 | 53 | 59 | 8 | 2 | 0.625 | 1.000 | 1.600 | 0.818 | 1.000 | 0.818 | 1.222 | 1.265 | 2.474 |
| Fujian | Ningde | 2 | 14 | 370 | 46 | 50 | 8 | 2 | 0.625 | 1.000 | 1.600 | 1.000 | 0.500 | 0.500 | 2.000 | 1.265 | 4.049 |
| Fujian | Sanming | 2 | 14 | 370 | 53 | 58 | 8 | 2 | 0.625 | 1.000 | 1.600 | 0.500 | 1.000 | 0.500 | 2.000 | 1.265 | 4.049 |
| Fujian | Zhangzhou | 2 | 14 | 370 | 82 | 63 | 8 | 3 | 0.625 | 0.333 | 4.800 | 0.111 | 1.000 | 0.111 | 9.000 | 1.265 | 54.661 |
| Fujian | #N/A | 2 | 14 | 370 |  | N/A | 8 | 2 | 0.625 | 1.000 |  |  |  |  |  |  |  |
| Fujian | #N/A | 2 | 14 | 370 |  | N/A | 8 | 2 | 0.625 | 1.000 |  |  |  |  |  |  |  |
| Fujian | Ningde | 2 | 14 | 370 | 46 | N/A | 8 | 2 | 0.625 | 1.000 |  |  |  |  |  |  |  |
| Fujian | Putian | 2 | 13 | 370 | 49 | 51 | 8 | 4 | 0.625 | 0.250 | 6.400 | 1.000 | 1.000 | 1.000 | 1.000 | 1.265 | 8.098 |
| Fujian | Nanping | 2 | 13 | 370 | 53 | 49 | 8 | 7 | 0.625 | 0.429 | 3.733 | 0.500 | 1.000 | 0.500 | 2.000 | 1.265 | 9.448 |
| Fujian | Nanping | 2 | 13 | 370 | 53 | 47 | 8 | 7 | 0.625 | 0.429 | 3.733 | 0.500 | 1.000 | 0.500 | 2.000 | 1.265 | 9.448 |
| Fujian | Sanming | 2 | 13 | 370 | 53 | 60 | 8 | 5 | 0.625 | 0.200 | 8.000 | 1.000 | 1.000 | 1.000 | 1.000 | 1.265 | 10.122 |
| Fujian | Nanping | 2 | 13 | 370 | 53 | 46 | 8 | 7 | 0.625 | 0.429 | 3.733 | 0.133 | 1.000 | 0.133 | 7.500 | 1.265 | 35.428 |
| Fujian | Zhangzhou | 2 | 13 | 370 | 82 | 64 | 8 | 13 | 0.625 | 0.231 | 6.933 | 0.625 | 0.333 | 0.208 | 4.800 | 1.265 | 42.109 |
| Fujian | #N/A | 2 | 13 | 370 |  | N/A | 8 | 4 | 0.625 | 0.500 |  |  |  |  |  |  |  |
| Fujian | #N/A | 2 | 13 | 370 |  | N/A | 8 | 4 | 0.625 | 0.500 |  |  |  |  |  |  |  |
| Fujian | Ningde | 2 | 13 | 370 | 46 | N/A | 8 | 7 | 0.625 | 0.286 |  |  |  |  |  |  |  |
| Fujian | Ningde | 2 | 13 | 370 | 46 | N/A | 8 | 7 | 0.625 | 0.286 |  |  |  |  |  |  |  |
| Fujian | Zhangzhou | 2 | 13 | 370 | 82 | N/A | 8 | 13 | 0.625 | 0.231 |  |  |  |  |  |  |  |
| Fujian | Zhangzhou | 2 | 13 | 370 | 82 | N/A | 8 | 13 | 0.625 | 0.231 |  |  |  |  |  |  |  |
| Beijing | Beijing | 3 | 12 | 935 | 736 | 36 | 1 | 81 | 1.000 | 0.148 | 6.750 | 1.000 | 0.250 | 0.250 | 4.000 | 0.776 | 20.951 |
| Beijing | Beijing | 3 | 12 | 935 | 736 | 26 | 1 | 81 | 1.000 | 0.148 | 6.750 | 1.000 | 0.250 | 0.250 | 4.000 | 0.776 | 20.951 |
| Beijing | Beijing | 3 | 12 | 935 | 736 | N/A | 1 | 81 | 1.000 | 0.148 |  |  |  |  |  |  |  |
| Beijing | Beijing | 3 | 12 | 935 | 736 | N/A | 1 | 81 | 1.000 | 0.148 |  |  |  |  |  |  |  |
| Beijing | Beijing | 3 | 12 | 935 | 736 | N/A | 1 | 81 | 1.000 | 0.148 |  |  |  |  |  |  |  |
| Beijing | Beijing | 3 | 12 | 935 | 736 | N/A | 1 | 81 | 1.000 | 0.148 |  |  |  |  |  |  |  |
| Beijing | Beijing | 3 | 12 | 935 | 736 | N/A | 1 | 81 | 1.000 | 0.148 |  |  |  |  |  |  |  |
| Beijing | Beijing | 3 | 12 | 935 | 736 | N/A | 1 | 81 | 1.000 | 0.148 |  |  |  |  |  |  |  |
| Beijing | Beijing | 3 | 12 | 935 | 736 | N/A | 1 | 81 | 1.000 | 0.148 |  |  |  |  |  |  |  |
| Beijing | Beijing | 3 | 12 | 935 | 736 | N/A | 1 | 81 | 1.000 | 0.148 |  |  |  |  |  |  |  |
| Beijing | Beijing | 3 | 12 | 935 | 736 | N/A | 1 | 81 | 1.000 | 0.148 |  |  |  |  |  |  |  |
| Beijing | Beijing | 3 | 12 | 935 | 736 | N/A | 1 | 81 | 1.000 | 0.148 |  |  |  |  |  |  |  |
| Beijing | Beijing | 3 | 11 | 935 | 736 | 45 | 1 | 68 | 1.000 | 0.176 | 5.667 | 1.000 | 0.583 | 0.583 | 1.714 | 0.776 | 7.538 |
| Beijing | Beijing | 3 | 11 | 935 | 736 | 43 | 1 | 68 | 1.000 | 0.176 | 5.667 | 1.000 | 0.583 | 0.583 | 1.714 | 0.776 | 7.538 |
| Beijing | Beijing | 3 | 11 | 935 | 736 | 42 | 1 | 68 | 1.000 | 0.176 | 5.667 | 1.000 | 0.583 | 0.583 | 1.714 | 0.776 | 7.538 |
| Beijing | Beijing | 3 | 11 | 935 | 736 | 29 | 1 | 68 | 1.000 | 0.176 | 5.667 | 1.000 | 0.583 | 0.583 | 1.714 | 0.776 | 7.538 |
| Beijing | Beijing | 3 | 11 | 935 | 736 | 28 | 1 | 68 | 1.000 | 0.176 | 5.667 | 1.000 | 0.583 | 0.583 | 1.714 | 0.776 | 7.538 |
| Beijing | Beijing | 3 | 11 | 935 | 736 | 38 | 1 | 68 | 1.000 | 0.176 | 5.667 | 0.933 | 0.583 | 0.544 | 1.837 | 0.776 | 8.076 |
| Beijing | Beijing | 3 | 11 | 935 | 736 | 39 | 1 | 68 | 1.000 | 0.176 | 5.667 | 0.786 | 0.583 | 0.458 | 2.182 | 0.776 | 9.594 |
| Beijing | Beijing | 3 | 11 | 935 | 736 | N/A | 1 | 68 | 1.000 | 0.176 |  |  |  |  |  |  |  |
| Beijing | Beijing | 3 | 11 | 935 | 736 | N/A | 1 | 68 | 1.000 | 0.176 |  |  |  |  |  |  |  |
| Beijing | Beijing | 3 | 11 | 935 | 736 | N/A | 1 | 68 | 1.000 | 0.176 |  |  |  |  |  |  |  |
| Beijing | Beijing | 3 | 11 | 935 | 736 | N/A | 1 | 68 | 1.000 | 0.176 |  |  |  |  |  |  |  |
| Beijing | Beijing | 3 | 11 | 935 | 736 | N/A | 1 | 68 | 1.000 | 0.176 |  |  |  |  |  |  |  |
| Beijing | Beijing | 3 | 10 | 935 | 736 | 44 | 1 | 67 | 1.000 | 0.179 | 5.583 | 1.000 | 0.917 | 0.917 | 1.091 | 0.776 | 4.726 |
| Beijing | Beijing | 3 | 10 | 935 | 736 | 41 | 1 | 67 | 1.000 | 0.179 | 5.583 | 1.000 | 0.917 | 0.917 | 1.091 | 0.776 | 4.726 |
| Beijing | Beijing | 3 | 10 | 935 | 736 | 40 | 1 | 67 | 1.000 | 0.179 | 5.583 | 1.000 | 0.917 | 0.917 | 1.091 | 0.776 | 4.726 |
| Beijing | Beijing | 3 | 10 | 935 | 736 | 37 | 1 | 67 | 1.000 | 0.179 | 5.583 | 1.000 | 0.917 | 0.917 | 1.091 | 0.776 | 4.726 |
| Beijing | Beijing | 3 | 10 | 935 | 736 | 34 | 1 | 67 | 1.000 | 0.179 | 5.583 | 1.000 | 0.917 | 0.917 | 1.091 | 0.776 | 4.726 |
| Beijing | Beijing | 3 | 10 | 935 | 736 | 33 | 1 | 67 | 1.000 | 0.179 | 5.583 | 1.000 | 0.917 | 0.917 | 1.091 | 0.776 | 4.726 |
| Beijing | Beijing | 3 | 10 | 935 | 736 | 32 | 1 | 67 | 1.000 | 0.179 | 5.583 | 1.000 | 0.917 | 0.917 | 1.091 | 0.776 | 4.726 |
| Beijing | Beijing | 3 | 10 | 935 | 736 | 31 | 1 | 67 | 1.000 | 0.179 | 5.583 | 1.000 | 0.917 | 0.917 | 1.091 | 0.776 | 4.726 |
| Beijing | Beijing | 3 | 10 | 935 | 736 | 30 | 1 | 67 | 1.000 | 0.179 | 5.583 | 1.000 | 0.917 | 0.917 | 1.091 | 0.776 | 4.726 |
| Beijing | Beijing | 3 | 10 | 935 | 736 | 27 | 1 | 67 | 1.000 | 0.179 | 5.583 | 1.000 | 0.917 | 0.917 | 1.091 | 0.776 | 4.726 |
| Beijing | Beijing | 3 | 10 | 935 | 736 | 35 | 1 | 67 | 1.000 | 0.179 | 5.583 | 0.833 | 0.917 | 0.764 | 1.309 | 0.776 | 5.672 |
| Beijing | Beijing | 3 | 10 | 935 | 736 | N/A | 1 | 67 | 1.000 | 0.179 |  |  |  |  |  |  |  |
| Anhui | Fuyang | 3 | 9 | 991 | 120 | N/A | 2 | 18 | 0.500 | 0.111 |  |  |  |  |  |  |  |
| Anhui | Fuyang | 3 | 9 | 991 | 120 | N/A | 2 | 18 | 0.500 | 0.111 |  |  |  |  |  |  |  |
| Anhui | Fuyang | 3 | 8 | 991 | 120 | 16 | 2 | 6 | 0.500 | 0.333 | 6.000 | 1.000 | 1.000 | 1.000 | 1.000 | 1.188 | 7.130 |
| Anhui | Fuyang | 3 | 8 | 991 | 120 | 15 | 2 | 6 | 0.500 | 0.333 | 6.000 | 1.000 | 1.000 | 1.000 | 1.000 | 1.188 | 7.130 |
| Anhui | Fuyang | 3 | 7 | 991 | 120 | 14 | 2 | 12 | 0.500 | 0.167 | 12.000 | 1.000 | 1.000 | 1.000 | 1.000 | 1.188 | 14.260 |
| Anhui | Fuyang | 3 | 7 | 991 | 120 | 17 | 2 | 12 | 0.500 | 0.167 | 12.000 | 0.889 | 1.000 | 0.889 | 1.125 | 1.188 | 16.042 |
| Anhui | Anqing | 2 | 6 | 991 | 73 | 5 | 9 | 10 | 0.333 | 0.200 | 15.000 | 1.000 | 1.000 | 1.000 | 1.000 | 1.188 | 17.825 |
| Anhui | Huainan | 2 | 6 | 991 | 78 | 19 | 9 | 10 | 0.333 | 0.200 | 15.000 | 1.000 | 1.000 | 1.000 | 1.000 | 1.188 | 17.825 |
| Anhui | Anqing | 2 | 6 | 991 | 73 | 3 | 9 | 10 | 0.333 | 0.200 | 15.000 | 0.833 | 1.000 | 0.833 | 1.200 | 1.188 | 21.390 |
| Anhui | Huainan | 2 | 6 | 991 | 78 | 18 | 9 | 10 | 0.333 | 0.200 | 15.000 | 0.818 | 1.000 | 0.818 | 1.222 | 1.188 | 21.786 |
| Anhui | Chuzhou | 2 | 6 | 991 | 70 | 12 | 9 | 12 | 0.333 | 0.167 | 18.000 | 0.800 | 1.000 | 0.800 | 1.250 | 1.188 | 26.737 |
| Anhui | Chuzhou | 2 | 6 | 991 | 70 | N/A | 9 | 12 | 0.333 | 0.167 |  |  |  |  |  |  |  |
| Anhui | Anqing | 2 | 5 | 991 | 73 | 4 | 9 | 1 | 0.333 | 1.000 | 3.000 | 1.000 | 1.000 | 1.000 | 1.000 | 1.188 | 3.565 |
| Anhui | Chuzhou | 2 | 5 | 991 | 70 | 11 | 9 | 1 | 0.333 | 1.000 | 3.000 | 1.000 | 1.000 | 1.000 | 1.000 | 1.188 | 3.565 |
| Anhui | Huainan | 2 | 5 | 991 | 78 | 23 | 9 | 4 | 0.333 | 0.500 | 6.000 | 1.000 | 1.000 | 1.000 | 1.000 | 1.188 | 7.130 |
| Anhui | Huainan | 2 | 5 | 991 | 78 | 21 | 9 | 4 | 0.333 | 0.500 | 6.000 | 1.000 | 1.000 | 1.000 | 1.000 | 1.188 | 7.130 |
| Anhui | Huainan | 2 | 4 | 991 | 78 | 22 | 9 | 9 | 0.333 | 0.222 | 13.500 | 0.875 | 1.000 | 0.875 | 1.143 | 1.188 | 18.334 |
| Anhui | Huainan | 2 | 4 | 991 | 78 | 20 | 9 | 9 | 0.333 | 0.222 | 13.500 | 0.833 | 1.000 | 0.833 | 1.200 | 1.188 | 19.251 |
| Anhui | Anqing | 2 | 4 | 991 | 73 | 2 | 9 | 11 | 0.333 | 0.182 | 16.500 | 1.000 | 1.000 | 1.000 | 1.000 | 1.188 | 19.607 |
| Anhui | Anqing | 2 | 4 | 991 | 73 | 1 | 9 | 11 | 0.333 | 0.182 | 16.500 | 1.000 | 1.000 | 1.000 | 1.000 | 1.188 | 19.607 |
| Anhui | Chuzhou | 2 | 4 | 991 | 70 | 13 | 9 | 8 | 0.333 | 0.250 | 12.000 | 1.000 | 0.500 | 0.500 | 2.000 | 1.188 | 28.520 |
| Anhui | Chuzhou | 2 | 4 | 991 | 70 | N/A | 9 | 8 | 0.333 | 0.250 |  |  |  |  |  |  |  |
| Anhui | Chizhou | 1 | 3 | 991 | 33 | 10 | 5 | 6 | 0.400 | 0.333 | 7.500 | 1.000 | 1.000 | 1.000 | 1.000 | 1.188 | 8.912 |
| Anhui | Chizhou | 1 | 3 | 991 | 33 | 8 | 5 | 6 | 0.400 | 0.333 | 7.500 | 1.000 | 1.000 | 1.000 | 1.000 | 1.188 | 8.912 |
| Anhui | Maanshan | 1 | 3 | 991 | 63 | 24 | 5 | 10 | 0.400 | 0.200 | 12.500 | 1.000 | 1.000 | 1.000 | 1.000 | 1.188 | 14.854 |
| Anhui | Maanshan | 1 | 3 | 991 | 63 | N/A | 5 | 10 | 0.400 | 0.200 |  |  |  |  |  |  |  |
| Anhui | Chizhou | 1 | 2 | 991 | 33 | 7 | 5 | 1 | 0.400 | 1.000 | 2.500 | 1.000 | 1.000 | 1.000 | 1.000 | 1.188 | 2.971 |
| Anhui | Maanshan | 1 | 2 | 991 | 63 | N/A | 5 | 3 | 0.400 | 0.667 |  |  |  |  |  |  |  |
| Anhui | Maanshan | 1 | 2 | 991 | 63 | N/A | 5 | 3 | 0.400 | 0.667 |  |  |  |  |  |  |  |
| Anhui | Chizhou | 1 | 1 | 991 | 33 | 9 | 5 | 3 | 0.400 | 0.667 | 3.750 | 1.000 | 1.000 | 1.000 | 1.000 | 1.188 | 4.456 |
| Anhui | Chizhou | 1 | 1 | 991 | 33 | 6 | 5 | 3 | 0.400 | 0.667 | 3.750 | 1.000 | 1.000 | 1.000 | 1.000 | 1.188 | 4.456 |
| Anhui | Maanshan | 1 | 1 | 991 | 63 | 25 | 5 | 6 | 0.400 | 0.333 | 7.500 | 0.118 | 0.500 | 0.059 | 17.000 | 1.188 | 151.511 |
| Anhui | Maanshan | 1 | 1 | 991 | 63 | N/A | 5 | 6 | 0.400 | 0.333 |  |  |  |  |  |  |  |
| Hubei | Suizhou | 1 |  | 68139 | 42 | N/A | 3 |  | 0.667 |  |  |  |  |  |  |  |  |
| Hubei | Jingmen | 2 |  | 68139 | 56 | N/A | 8 |  | 0.500 |  |  |  |  |  |  |  |  |
| Hubei | Xiaogan | 2 |  | 68139 | 60 | N/A | 8 |  | 0.500 |  |  |  |  |  |  |  |  |
| Guangdong | Ji eyang | 2 |  | 1721 | 62 | N/A | 12 |  | 0.333 |  |  |  |  |  |  |  |  |
| Guangdong | Yangjiang | 2 |  | 1721 | 57 | N/A | 12 |  | 0.333 |  |  |  |  |  |  |  |  |
| Henan | Sanmenxia | 2 |  | 1276 | 51 | N/A | 10 |  | 0.500 |  |  |  |  |  |  |  |  |
| Henan | Sanmenxia | 2 |  | 1276 | 51 | N/A | 10 |  | 0.500 |  |  |  |  |  |  |  |  |
| Hunan | Zhangjiajie | 1 |  | 1019 | 32 | N/A | 1 |  | 1.000 |  |  |  |  |  |  |  |  |
| Hunan | Zhangjiajie | 1 |  | 1019 | 32 | N/A | 1 |  | 1.000 |  |  |  |  |  |  |  |  |
| Anhui | Chizhou | 1 |  | 991 | 33 | N/A | 5 |  | 0.400 |  |  |  |  |  |  |  |  |
| Anhui | Anqing | 2 |  | 991 | 73 | N/A | 9 |  | 0.333 |  |  |  |  |  |  |  |  |
| Anhui | Chuzhou | 2 |  | 991 | 70 | N/A | 9 |  | 0.333 |  |  |  |  |  |  |  |  |
| Heilongjiang | Shuangyashan | 1 |  | 948 | 51 | N/A | 5 |  | 0.400 |  |  |  |  |  |  |  |  |
| Heilongjiang | Shuangyashan | 1 |  | 948 | 51 | N/A | 5 |  | 0.400 |  |  |  |  |  |  |  |  |
| Heilongjiang | Suihua | 2 |  | 948 | 40 | N/A | 6 |  | 0.500 |  |  |  |  |  |  |  |  |
| Jiangxi | Yingtan | 1 |  | 932 | 33 | N/A | 4 |  | 0.750 |  |  |  |  |  |  |  |  |
| Xinjiang | Shihezi | 2 |  | 902 |  | N/A | 1 |  | 1.000 |  |  |  |  |  |  |  |  |
| Xinjiang | Altay | 1 |  | 902 | 0 | N/A | 3 |  | 1.000 |  |  |  |  |  |  |  |  |
| Xinjiang | Altay | 1 |  | 902 | 0 | N/A | 3 |  | 1.000 |  |  |  |  |  |  |  |  |
| Xinjiang | Hami | 1 |  | 902 | 35 | N/A | 3 |  | 1.000 |  |  |  |  |  |  |  |  |
| Xinjiang | Hami | 1 |  | 902 | 35 | N/A | 3 |  | 1.000 |  |  |  |  |  |  |  |  |
| Xinjiang | Karamay | 1 |  | 902 | 13 | N/A | 3 |  | 1.000 |  |  |  |  |  |  |  |  |
| Xinjiang | Turpan | 1 |  | 902 | 19 | N/A | 3 |  | 1.000 |  |  |  |  |  |  |  |  |
| Xinjiang | Turpan | 1 |  | 902 | 19 | N/A | 3 |  | 1.000 |  |  |  |  |  |  |  |  |
| Sichuan | Deyang | 2 |  | 626 | 90 | N/A | 14 |  | 0.357 |  |  |  |  |  |  |  |  |
| Sichuan | Guang'an | 2 |  | 626 | 73 | N/A | 14 |  | 0.357 |  |  |  |  |  |  |  |  |
| Sichuan | Meishan | 2 |  | 626 | 88 | N/A | 14 |  | 0.357 |  |  |  |  |  |  |  |  |
| Sichuan | Ya'an | 2 |  | 626 | 43 | N/A | 14 |  | 0.357 |  |  |  |  |  |  |  |  |
| Fujian | Nanping | 2 |  | 370 | 53 | N/A | 8 |  | 0.625 |  |  |  |  |  |  |  |  |
| Shaanxi | Shangluo | 2 |  | 356 | 180 | N/A | 8 |  | 0.625 |  |  |  |  |  |  |  |  |
| Guangxi | Chongzuo | 1 |  | 355 | 29 | N/A | 6 |  | 0.333 |  |  |  |  |  |  |  |  |
| Guangxi | Chongzuo | 1 |  | 355 | 29 | N/A | 6 |  | 0.333 |  |  |  |  |  |  |  |  |
| Hebei | Xingtai | 2 |  | 351 | 204 | N/A | 6 |  | 0.500 |  |  |  |  |  |  |  |  |
| Inner Mongolia | Wuhai | 1 |  | 260 | 28 | N/A | 3 |  | 0.667 |  |  |  |  |  |  |  |  |
| Yunnan | Lincang | 1 |  | 195 | 52 | N/A | 3 |  | 0.333 |  |  |  |  |  |  |  |  |
| Hainan | Danzhou | 1 |  | 171 | 19 | N/A | 3 |  | 0.667 |  |  |  |  |  |  |  |  |
| Hainan | Danzhou | 1 |  | 171 | 19 | N/A | 3 |  | 0.667 |  |  |  |  |  |  |  |  |
| Gansu | Zhangye | 1 |  | 169 | 45 | N/A | 8 |  | 0.500 |  |  |  |  |  |  |  |  |
| Jilin | Siping | 2 |  | 157 | 78 | N/A | 3 |  | 0.667 |  |  |  |  |  |  |  |  |
| Jilin | Baishan | 1 |  | 157 | 40 | N/A | 4 |  | 0.500 |  |  |  |  |  |  |  |  |
| Jilin | Baishan | 1 |  | 157 | 40 | N/A | 4 |  | 0.500 |  |  |  |  |  |  |  |  |
| Jilin | Liaoyuan | 1 |  | 157 | 33 | N/A | 4 |  | 0.500 |  |  |  |  |  |  |  |  |
| Guizhou | Tongren | 2 |  | 147 | 107 | N/A | 3 |  | 1.000 |  |  |  |  |  |  |  |  |
| Ningxia | Guyuan | 1 |  | 75 | 13 | N/A | 4 |  | 1.000 |  |  |  |  |  |  |  |  |
| Ningxia | Guyuan | 1 |  | 75 | 13 | N/A | 4 |  | 1.000 |  |  |  |  |  |  |  |  |
| Ningxia | Shizuishan | 1 |  | 75 | 36 | N/A | 4 |  | 1.000 |  |  |  |  |  |  |  |  |
| Ningxia | Shizuishan | 1 |  | 75 | 36 | N/A | 4 |  | 1.000 |  |  |  |  |  |  |  |  |
| Ningxia | Wuzhong | 1 |  | 75 | 50 | N/A | 4 |  | 1.000 |  |  |  |  |  |  |  |  |
| Ningxia | Wuzhong | 1 |  | 75 | 50 | N/A | 4 |  | 1.000 |  |  |  |  |  |  |  |  |
| Ningxia | Zhongwei | 1 |  | 75 | 32 | N/A | 4 |  | 1.000 |  |  |  |  |  |  |  |  |
| Ningxia | Zhongwei | 1 |  | 75 | 32 | N/A | 4 |  | 1.000 |  |  |  |  |  |  |  |  |
| Qinghai | Haibei | 1 |  | 18 | 0 | N/A | 1 |  | 1.000 |  |  |  |  |  |  |  |  |
| Qinghai | Haibei | 1 |  | 18 | 0 | N/A | 1 |  | 1.000 |  |  |  |  |  |  |  |  |
| Qinghai | Haidong | 1 |  | 18 | 57 | N/A | 1 |  | 1.000 |  |  |  |  |  |  |  |  |
| Qinghai | Haidong | 1 |  | 18 | 57 | N/A | 1 |  | 1.000 |  |  |  |  |  |  |  |  |
| Qinghai | Hainan | 1 |  | 18 | 0 | N/A | 1 |  | 1.000 |  |  |  |  |  |  |  |  |
| Qinghai | Hainan | 1 |  | 18 | 0 | N/A | 1 |  | 1.000 |  |  |  |  |  |  |  |  |
| Qinghai | Haixi | 1 |  | 18 | 0 | N/A | 1 |  | 1.000 |  |  |  |  |  |  |  |  |
| Qinghai | Yushu | 2 |  | 18 |  | N/A | 1 |  | 1.000 |  |  |  |  |  |  |  |  |
| Qinghai | Yushu | 2 |  | 18 |  | N/A | 1 |  | 1.000 |  |  |  |  |  |  |  |  |
| Tibet | Qamdo | 1 |  | 1 | 29 | N/A | 6 |  | 1.000 |  |  |  |  |  |  |  |  |
| Tibet | Qamdo | 1 |  | 1 | 29 | N/A | 6 |  | 1.000 |  |  |  |  |  |  |  |  |
| Tibet | Linzhi | 1 |  | 1 | 17 | N/A | 6 |  | 1.000 |  |  |  |  |  |  |  |  |
| Tibet | Linzhi | 1 |  | 1 | 17 | N/A | 6 |  | 1.000 |  |  |  |  |  |  |  |  |
| Tibet | Naqu | 1 |  | 1 | 28 | N/A | 6 |  | 1.000 |  |  |  |  |  |  |  |  |
| Tibet | Xigaze | 1 |  | 1 | 29 | N/A | 6 |  | 1.000 |  |  |  |  |  |  |  |  |
| Tibet | Xigaze | 1 |  | 1 | 29 | N/A | 6 |  | 1.000 |  |  |  |  |  |  |  |  |
| Tibet | Shannan | 1 |  | 1 | 18 | N/A | 6 |  | 1.000 |  |  |  |  |  |  |  |  |
| Tibet | Shannan | 1 |  | 1 | 18 | N/A | 6 |  | 1.000 |  |  |  |  |  |  |  |  |

# Supplement 7: Questionnaire design

**Gender:**

Male, Female

**Birthday:** Month, Year

**Marital status:**

Married, Unmarried

**Nationality**:

**Workplace:**

Province/ Municipality directly under the Central Government

City (prefecture-level city)

Hospital

**Specialty:**

Gynecology, Obstetrics, Reproductive endocrinology, Family planning, Not divided

**The level of education:**

Below bachelor, Bachelor, Master, Doctor

**Working years in the hospital (including postgraduate education):**

1-5 years, 6-10 years, 11-15 years, 16-20 years, More than 2019 than 20 years

**Professional title:**

Chief doctor, Attending doctor, Resident doctor, Others

**Did you participate in the work related to COVID-19 pandemic in the first half of 2020? (multiple choices)**

Yes, involved in treating COVID-19 patients, as a supporter from other places

Yes, involved in treating COVID-19 patients, as a local doctor

Yes, emergency department or fever clinics

Yes, nucleic acid testing (local/ nonlocal)

Not directly involved in COVID-19 pandemic-related medical work

**The most stressful period of the pandemic you experienced was: (multiple choices)**

January, February, March, April, May, June

**During the most intense period of COVID-19 pandemic, how did you engage in the following medical activities compared to 2019?**

If you choose "Irrelevant", please skip "after July 1, 2020".

| **Outpatient visits** | Irrelevant | All stopped | Reduction by >50% | Reduction by 25-50% | Reduction by <25% | No change |
| --- | --- | --- | --- | --- | --- | --- |
| **After July 1, 2020** | Same as 2019 | Still less 2019 | More than 2019 | | | |
| **Surgical volumes** | Irrelevant | All stopped | Reduction by >50% | Reduction by 25-50% | Reduction by <25% | No change |
| **After July 1, 2020** | Same as 2019 | Still less 2019 | More than 2019 | | | |
| **Consultation request** | Irrelevant | All stopped | Reduction by >50% | Reduction by 25-50% | Reduction by <25% | No change |
| **After July 1, 2020** | Same as 2019 | Still less 2019 | More than 2019 |  |  |  |
| **Outpatient appointment** | Irrelevant | All stopped | Reduction by >50% | Reduction by 25-50% | Reduction by <25% | No change |
| **After July 1, 2020** | Same as 2019 | Still less 2019 | More than 2019 |  |  |  |
| **Admission arrangement** | Irrelevant | All stopped | Reduction by >50% | Reduction by 25-50% | Reduction by <25% | No change |
| **After July 1, 2020** | Same as 2019 | Still less 2019 | More than 2019 |  |  |  |
| **Emergency visits** | Irrelevant | All stopped | Reduction by >50% | Reduction by 25-50% | Reduction by <25% | No change |
| **After July 1, 2020** | Same as 2019 | Still less 2019 | More than 2019 |  |  |  |
| **Online consultation** | Irrelevant | Reduction by >50% | Reduction by <50% | No change | Increased by <50% | Increased >50% |
| **After July 1, 2020** | Same as 2019 | Still less 2019 | More than 2019 |  |  |  |

**Based on your experience, do you think the pandemic has any impact on the following problems?**

| **Preconception counseling** | Irrelevant | All stopped | Reduction by >50% | Reduction by 25-50% | Reduction by <25% | No change |
| --- | --- | --- | --- | --- | --- | --- |
| **Prenatal examinations** | Irrelevant | All stopped | Reduction by >50% | Reduction by 25-50% | Reduction by <25% | No change |
| **Prenatal diagnosis** | Irrelevant | All stopped | Reduction by >50% | Reduction by 25-50% | Reduction by <25% | No change |
| **Instructions for contraception and miscarriage** | Irrelevant | All stopped | Reduction by >50% | Reduction by 25-50% | Reduction by <25% | No change |
| **Assisted reproduction** | Irrelevant | All stopped | Reduction by >50% | Reduction by 25-50% | Reduction by <25% | No change |
| **Outpatient surgeries and procedures** | Irrelevant | All stopped | Reduction by >50% | Reduction by 25-50% | Reduction by <25% | No change |
| **Emergent obstetrical surgeries** | Irrelevant | All stopped | Reduction by >50% | Reduction by 25-50% | Reduction by <25% | No change |
| **Emergent gynecological surgeries** | Irrelevant | All stopped | Reduction by >50% | Reduction by 25-50% | Reduction by <25% | No change |
| **Cervical cancer screening** | Irrelevant | All stopped | Reduction by >50% | Reduction by 25-50% | Reduction by <25% | No change |
| **Treatment for benign neoplasm** | Irrelevant | All stopped | Reduction by >50% | Reduction by 25-50% | Reduction by <25% | No change |
| **Treatment for malignancies** | Irrelevant | All stopped | Reduction by >50% | Reduction by 25-50% | Reduction by <25% | No change |
| **Follow-up for malignancies** | Irrelevant | All stopped | Reduction by >50% | Reduction by 25-50% | Reduction by <25% | No change |
| **Treatment for pelvic floor dysfunctions** | Irrelevant | All stopped | Reduction by >50% | Reduction by 25-50% | Reduction by <25% | No change |

# Supplement 8: Hospital codes in this study

| **Codes** | **Hospital** |
| --- | --- |
| 1 | Anqing Third People's Hospital (Anqing Red Cross Hospital) |
| 2 | Anqing first people's Hospital |
| 3 | Anqing textile hospital |
| 4 | Anqing Municipal Hospital (Anqing hospital) |
| 5 | Taihu County People's Hospital |
| 6 | Chizhou Second People's Hospital |
| 7 | Chizhou people's Hospital |
| 8 | Chizhou Hospital of traditional Chinese Medicine |
| 9 | Dongzhi Hospital of traditional Chinese Medicine |
| 10 | Jiuhuashan people's Hospital |
| 11 | Chuzhou first people's Hospital |
| 12 | People's Hospital of Langya District, Chuzhou City |
| 13 | Laian County Hospital of traditional Chinese Medicine |
| 14 | Funan County Hospital |
| 15 | Fuyang people's Hospital |
| 16 | Fuyang Hospital of traditional Chinese Medicine |
| 17 | Linquan people's Hospital |
| 18 | Fengtai people's Hospital |
| 19 | Fengtai Hospital of traditional Chinese Medicine |
| 20 | Huainan Second People's Hospital |
| 21 | Huainan first people's Hospital |
| 22 | Huainan Hospital of traditional Chinese Medicine |
| 23 | Huainan Xinhua Hospital |
| 24 | Dangtu people's Hospital |
| 25 | Ma'anshan maternal and child health care hospital |
| 26 | Beijing Chaoyang Guanghua hospital |
| 27 | Beijing Fengtai Hospital |
| 28 | Beijing Jishuitan Hospital (Fourth Affiliated Hospital of Peking University, Jishuitan Hospital, Fourth Clinical Medical College of Peking University) |
| 29 | Beijing Jingmei Group General Hospital |
| 30 | Beijing Changping District Hospital |
| 31 | Beijing Chuiyangliu hospital (Beijing minimally invasive hospital) |
| 32 | Beijing Sixth Hospital |
| 33 | Beijing Haidian Hospital |
| 34 | Beijing Hui people's Hospital |
| 35 | Beijing Puren hospital (Beijing Fourth Hospital) |
| 36 | Tongzhou District Second Hospital of Beijing |
| 37 | Beijing integrated traditional Chinese and Western Medicine Hospital |
| 38 | Peking Union Medical College Hospital |
| 39 | Beijing Cancer Hospital (School of oncology, Peking University, Beijing Institute of cancer prevention and treatment) |
| 40 | Miyun maternal and child health care hospital |
| 41 | Yuquan Hospital of Tsinghua University (the Second Affiliated Hospital of Tsinghua University) |
| 42 | Fuxing Hospital Affiliated to Capital Medical University |
| 43 | Xuanwu Hospital Affiliated to Capital Medical University |
| 44 | Yanqing County Hospital |
| 45 | China Japan Friendship Hospital |
| 46 | Nanping First Hospital |
| 47 | Nanping Hospital of traditional Chinese Medicine |
| 48 | Songxi maternal and child health care hospital |
| 49 | Wuyishan Municipal Hospital |
| 50 | Ningde Mindong hospital |
| 51 | Putian people's Hospital |
| 52 | Putian Chengxiang District Hospital |
| 53 | Putian First Hospital |
| 54 | Affiliated Hospital of Putian University |
| 55 | Xianyou maternal and child health care hospital |
| 56 | Xianyou County Hospital |
| 57 | Mingxi County Hospital |
| 58 | Sanming Second Hospital |
| 59 | Sanming First Hospital |
| 60 | Sanming Hospital of traditional Chinese Medicine |
| 61 | Shaxian maternal and child health care hospital |
| 62 | Yongan maternal and child health care hospital |
| 63 | Zhangzhou Hospital of Fujian Province |
| 64 | Zhangzhou Longwen hospital |
| 65 | Zhangzhou Hospital of traditional Chinese Medicine |
| 66 | Jingtai Hospital of traditional Chinese Medicine |
| 67 | General Hospital of Jingyuan Coal Industry Co., Ltd |
| 68 | Jingyuan Hospital of traditional Chinese Medicine |
| 69 | Gansu Provincial People's Hospital |
| 70 | Lanzhou first people's Hospital |
| 71 | Yuzhong maternal and child health care hospital |
| 72 | Qingyang maternal and child health care hospital |
| 73 | Qingyang people's Hospital |
| 74 | Qingyang Hospital of traditional Chinese Medicine |
| 75 | Xifeng District People's Hospital |
| 76 | Zhenyuan Second People's Hospital |
| 77 | Tianshui Second People's Hospital |
| 78 | Tianshui Fourth People's Hospital |
| 79 | Tianshui maternal and child health care hospital |
| 80 | Tianshui Railway Hospital |
| 81 | Minqin people's Hospital |
| 82 | Tianzhu people's Hospital |
| 83 | Wuwei people's Hospital |
| 84 | Wuwei Cancer Hospital |
| 85 | Gaotai people's Hospital |
| 86 | Shandan people's Hospital |
| 87 | Zhangye maternal and Child Health Hospital |
| 88 | Zhangye people's Hospital |
| 89 | Guangdong maternal and child health care hospital |
| 90 | Xinhua Hospital, Huadu District, Guangzhou |
| 91 | Shahe people's Hospital of Tianhe District, Guangzhou |
| 92 | The First Affiliated Hospital of Guangzhou Medical College |
| 93 | JieXi people's Hospital |
| 94 | Lihu Central Hospital of Puning City, Jieyang City |
| 95 | Puning maternal and child health care hospital |
| 96 | Puning people's Hospital |
| 97 | Lechang Hospital of traditional Chinese Medicine |
| 98 | Qujiang maternal and child health care hospital |
| 99 | Shaoguan first people's Hospital |
| 100 | Shaoguan Yuebei people's Hospital |
| 101 | Shaoguan Hospital of traditional Chinese Medicine |
| 102 | Yangdong maternal and child health care hospital |
| 103 | Yangjiang people's Hospital |
| 104 | Zhaoqing first people's Hospital |
| 105 | Zhuhai maternal and child health care hospital |
| 106 | Zhuhai People's Hospital |
| 107 | Zhuhai Sanzao people's Hospital |
| 108 | Baise maternal and child health care hospital |
| 109 | Baise people's Hospital |
| 110 | Tiandong Hospital of traditional Chinese Medicine |
| 111 | Tianlin Hospital of traditional Chinese Medicine |
| 112 | Affiliated Hospital of Youjiang Medical College for Nationalities |
| 113 | Beihai Second People's Hospital |
| 114 | Beihai maternal and child health care hospital |
| 115 | Beihai people's Hospital |
| 116 | Beihai Hospital of traditional Chinese Medicine |
| 117 | Chongzuo people's Hospital |
| 118 | Chongzuo Hospital of traditional Chinese Medicine |
| 119 | Fushui maternal and child health care hospital |
| 120 | Liuzhou people's Hospital of Guangxi Zhuang Autonomous Region |
| 121 | Cenxi maternal and child health care hospital |
| 122 | Mengshan maternal and child health care hospital |
| 123 | Wuzhou Red Cross Hospital |
| 124 | Wuzhou people's Hospital |
| 125 | Bobai people's Hospital |
| 126 | Yulin first people's Hospital |
| 127 | Anshun maternal and child health care hospital |
| 128 | Anshun people's Hospital |
| 129 | Pingba Hospital of traditional Chinese Medicine |
| 130 | Zhenning people's Hospital |
| 131 | Bijie district hospital |
| 132 | Bijie Hospital of traditional Chinese Medicine |
| 133 | Dafang people's Hospital |
| 134 | Qianxinan people's Hospital |
| 135 | Qianxi people's Hospital |
| 136 | Weining people's Hospital |
| 137 | Guiyang first people's Hospital |
| 138 | Guiyang Yunyan District People's Hospital |
| 139 | The First Affiliated Hospital of Guiyang University of traditional Chinese Medicine |
| 140 | Guizhou electric power staff hospital |
| 141 | Guizhou Wanjiang hospital |
| 142 | Qingzhen Hospital of traditional Chinese Medicine |
| 143 | Liupanshui people's Hospital |
| 144 | Liuzhi Mining Bureau General Hospital |
| 145 | Shuicheng Mining Group General Hospital |
| 146 | Shuicheng people's Hospital |
| 147 | General Hospital of shuikuang group |
| 148 | Zhongshan People's Hospital |
| 149 | Dejiang people's Hospital |
| 150 | Tongren people's Hospital |
| 151 | Tongren Hospital of traditional Chinese Medicine |
| 152 | Wanshan Hospital of the people's special zone |
| 153 | Yuping people's Hospital |
| 154 | Chishui people's Hospital |
| 155 | Zunyi fire hospital |
| 156 | Zunyi Hospital of traditional Chinese Medicine |
| 157 | Zunyi Xiangjiang hospital |
| 158 | Affiliated Hospital of Zunyi Medical College |
| 159 | Danzhou first people's Hospital |
| 160 | Hainan state owned Xipei Farm Hospital |
| 161 | Haikou maternal and child health care hospital |
| 162 | Hainan Provincial People's Hospital |
| 163 | Hainan Hospital of traditional Chinese Medicine |
| 164 | The third people's Hospital of Hainan Province |
| 165 | Sanya Hospital of Hainan agricultural reclamation |
| 166 | Sanya maternal and child health care hospital |
| 167 | Sanya people's Hospital |
| 168 | Sanya Hospital of traditional Chinese Medicine |
| 169 | Baoding construction hospital |
| 170 | Baoding First Hospital |
| 171 | Baoding first Central Hospital |
| 172 | Baoding maternal and child health care hospital |
| 173 | Quyang County Hospital |
| 174 | Tang County Maternal and child health care hospital |
| 175 | Cangzhou people's Hospital |
| 176 | Cangzhou Central Hospital |
| 177 | Cangzhou Hospital of traditional Chinese Medicine |
| 178 | Nanpi people's Hospital |
| 179 | Qingxian maternal and child health care hospital |
| 180 | Chengde maternal and child health care hospital |
| 181 | Chengde Central Hospital |
| 182 | Chengde Hospital of traditional Chinese Medicine |
| 183 | Affiliated Hospital of Chengde Medical College |
| 184 | Fengning maternal and child health care hospital |
| 185 | Longhua maternal and child health care hospital |
| 186 | Cixian maternal and child health care hospital |
| 187 | Handan fourth hospital |
| 188 | Handan First Hospital |
| 189 | Handan Central Hospital |
| 190 | Wu'an hospital |
| 191 | Changli maternal and child health care hospital |
| 192 | Changli people's Hospital |
| 193 | Lulong maternal and child health care hospital |
| 194 | Qinhuangdao First Hospital |
| 195 | Qinhuangdao maternal and child health care hospital |
| 196 | Qinhuangdao railway hospital |
| 197 | Julu County Hospital |
| 198 | Renxian Hospital of traditional Chinese Medicine |
| 199 | Shahe hospital |
| 200 | Xinhe Hospital of traditional Chinese Medicine |
| 201 | Xingtai people's Hospital |
| 202 | Anyang Third People's Hospital |
| 203 | Anyang maternal and child health care hospital |
| 204 | Anyang Hospital of traditional Chinese Medicine |
| 205 | Huaxian Central Hospital |
| 206 | Neihuang Hospital of traditional Chinese Medicine |
| 207 | Jiaozuo Second People's Hospital |
| 208 | Jiaozuo Fifth People's Hospital |
| 209 | Jiaozuo Hospital of traditional Chinese Medicine |
| 210 | Mengzhou maternal and child health care hospital |
| 211 | Shanyang maternal and child health care hospital |
| 212 | Luoyang Second People's Hospital |
| 213 | Luoyang Second Hospital of traditional Chinese Medicine |
| 214 | Luoyang Third People's Hospital (Luoyang Railway Central Hospital) |
| 215 | Luoyang first people's Hospital |
| 216 | Yichuan Hospital of traditional Chinese Medicine |
| 217 | Linying people's Hospital |
| 218 | Luohe Third People's Hospital (Luohe maternal and child health care hospital) |
| 219 | Luohe first people's Hospital |
| 220 | Luohe Central Hospital |
| 221 | Luohe Hospital of traditional Chinese Medicine |
| 222 | Wuyang people's Hospital |
| 223 | Lushi County Hospital of traditional Chinese Medicine |
| 224 | Mianchi County Hospital of traditional Chinese Medicine |
| 225 | Sanmenxia people's Hospital |
| 226 | Sanmenxia Central Hospital |
| 227 | Fengqiu maternal and child health care hospital |
| 228 | Xinxiang first people's Hospital |
| 229 | Xinxiang maternal and child health care hospital |
| 230 | Xinxiang Railway Hospital |
| 231 | The First Affiliated Hospital of Xinxiang Medical College |
| 232 | Yuanyang Hospital of traditional Chinese Medicine |
| 233 | Daqing fourth hospital |
| 234 | Daqing people's Hospital |
| 235 | Daqing Railway Hospital |
| 236 | Daqing Oilfield General Hospital |
| 237 | Zhaoyuan maternal and child health care hospital |
| 238 | Fangzheng maternal and child health care hospital |
| 239 | Harbin Pingfang District People's Hospital |
| 240 | Cancer Hospital Affiliated to Harbin Medical University |
| 241 | Heilongjiang Electric Power Hospital |
| 242 | Heilongjiang Red Cross Hospital |
| 243 | Hulan maternal and child health care hospital |
| 244 | Fuyuan maternal and child health care hospital |
| 245 | Fujin maternal and child health care hospital |
| 246 | Huanan people's Hospital |
| 247 | jiamusi central hospital |
| 248 | Jiamusi Hospital of traditional Chinese Medicine |
| 249 | Baoqing people's Hospital |
| 250 | Jixian Hospital of traditional Chinese Medicine |
| 251 | Raohe maternal and child health care hospital |
| 252 | Raohe people's Hospital |
| 253 | People's Hospital of Qing'an County |
| 254 | Suihua Second Hospital |
| 255 | Suihua First Hospital |
| 256 | Wangkui County Hospital |
| 257 | The second Forestry hospital of Heilongjiang Province |
| 258 | Staff hospital of Hongxing Forestry Bureau |
| 259 | Yichun forestry Central Hospital |
| 260 | People's Hospital of Xilin District, Yichun City |
| 261 | Staff hospital of Echeng Iron and steel plant |
| 262 | Staff hospital of E Steel Group Company |
| 263 | Ezhou Second Hospital |
| 264 | Ezhou maternal and child health care hospital |
| 265 | Ezhou Hospital of traditional Chinese Medicine |
| 266 | Huangshi Second Hospital |
| 267 | Huangshi Tieshan people's Hospital |
| 268 | Huangshi Central Hospital |
| 269 | Huangshi Hospital of traditional Chinese Medicine |
| 270 | Yangxin maternal and child health care hospital |
| 271 | Yangxin Hospital of traditional Chinese Medicine |
| 272 | Jingmen construction hospital |
| 273 | Jingmen Second People's Hospital |
| 274 | Jingmen first people's Hospital |
| 275 | Jingmen Hospital of traditional Chinese Medicine |
| 276 | Shayang Farm General Hospital |
| 277 | Baiyun Hospital of Zengdu District |
| 278 | Guangshui first people's Hospital |
| 279 | Suizhou Second People's Hospital |
| 280 | Suizhou Central Hospital |
| 281 | Suizhou Hospital of traditional Chinese Medicine |
| 282 | Dongfeng Xiangfan hospital |
| 283 | Laohekou maternal and child health care hospital |
| 284 | Xiangfan Fourth People's Hospital |
| 285 | Xiangfan first people's Hospital |
| 286 | Xiangfan Central Hospital |
| 287 | Yicheng maternal and child health care hospital |
| 288 | Xiaochang maternal and child health care hospital |
| 289 | Xiaogan Xiaonan maternal and child health care hospital |
| 290 | Xiaogan Central Hospital |
| 291 | Xiaogan Hospital of traditional Chinese Medicine |
| 292 | Yingcheng people's Hospital |
| 293 | Chenzhou first people's Hospital |
| 294 | Chenzhou Hospital of traditional Chinese Medicine |
| 295 | Chenzhou Railway Hospital |
| 296 | Guiyang maternal and child health care hospital |
| 297 | Rucheng maternal and child health care hospital |
| 298 | Affiliated Hospital of Xiangnan University |
| 299 | Hengyang Central Hospital |
| 300 | Hengyang Hospital of traditional Chinese Medicine |
| 301 | Hengyang Railway Hospital |
| 302 | The First Affiliated Hospital of Nanhua University |
| 303 | Nanyue maternal and child health care hospital |
| 304 | Nanyue District People's Hospital |
| 305 | Anhua maternal and child health care hospital |
| 306 | Yiyang Third People's Hospital (Yiyang County People's Hospital) |
| 307 | Yiyang first people's Hospital |
| 308 | Yiyang people's Hospital |
| 309 | Yiyang Central Hospital |
| 310 | Cili maternal and child health care hospital |
| 311 | Zhangjiajie people's Hospital |
| 312 | Zhangjiajie Wulingyuan people's Hospital |
| 313 | Zhangjiajie Hospital of traditional Chinese Medicine |
| 314 | Hunan hospital of traditional Chinese Medicine |
| 315 | Hunan Provincial Tumor Hospital |
| 316 | Liuyang Hospital of traditional Chinese Medicine |
| 317 | Changsha Yuelu District People's Hospital |
| 318 | Changsha hospital of traditional Chinese Medicine |
| 319 | Changsha Red Cross Hospital |
| 320 | Chaling maternal and child health care hospital |
| 321 | Liling maternal and child health care hospital |
| 322 | Zhuzhou First Hospital |
| 323 | Zhuzhou people's Hospital |
| 324 | Zhuzhou Hospital of traditional Chinese Medicine |
| 325 | Zhuzhou Railway Hospital |
| 326 | Baishan Hospital of traditional Chinese Medicine |
| 327 | Jiangyuan integrated traditional Chinese and Western Medicine Hospital |
| 328 | Jingyu people's Hospital |
| 329 | Linjiang hospital |
| 330 | Dongfeng maternal and child health care hospital |
| 331 | Dongliao Hospital of traditional Chinese Medicine |
| 332 | Liaoyuan maternal and infant hospital |
| 333 | Liaoyuan Central Hospital |
| 334 | Liaoyuan Hospital of traditional Chinese Medicine |
| 335 | Gongzhuling Hospital of traditional Chinese Medicine |
| 336 | Shuangliao maternal and child health care hospital |
| 337 | Siping first people's Hospital |
| 338 | Siping Central People's Hospital |
| 339 | Tiexi District Women and infant hospital |
| 340 | Ji'an maternal and child health care hospital |
| 341 | Liuhe maternal and child health station |
| 342 | Meihekou Hospital of traditional Chinese Medicine |
| 343 | General Hospital of Tonghua Mining Bureau |
| 344 | Tonghua people's Hospital |
| 345 | Tonghua Hospital of traditional Chinese Medicine |
| 346 | Headquarters of China Japan Friendship Hospital of Jilin University |
| 347 | Nanguan maternal and Child Health Hospital |
| 348 | FAW General Hospital |
| 349 | Changchun National Hospital |
| 350 | Changchun Friendship Hospital |
| 351 | Changchun Railway Hospital |
| 352 | Donghai Hospital of traditional Chinese Medicine |
| 353 | Ganyu people's Hospital |
| 354 | Lianyungang Harbor Hospital |
| 355 | Hospital of Lianyungang Development Zone |
| 356 | Lianyungang first people's Hospital |
| 357 | Lianyungang Hospital of traditional Chinese Medicine |
| 358 | Jiangsu Provincial People's Hospital |
| 359 | Liuhe people's Hospital |
| 360 | Nanjing Maternal and child health care hospital |
| 361 | Nanjing Jianye hospital |
| 362 | Nanjing Jiangning District Hospital of traditional Chinese Medicine |
| 363 | Qixia maternal and child health care hospital |
| 364 | Jiangyan people's Hospital |
| 365 | Jingjiang Fourth People's Hospital |
| 366 | Taizhou Baima people's Hospital |
| 367 | Taizhou People's Hospital |
| 368 | Taizhou Hospital of traditional Chinese Medicine |
| 369 | Xinghua people's Hospital |
| 370 | Wuxi Binhu District Hospital of traditional Chinese Medicine |
| 371 | Wuxi maternal and child health care hospital |
| 372 | Wuxi People's Hospital |
| 373 | Wuxi Xishan District People's Hospital |
| 374 | Yixing People's hospital |
| 375 | Yixing Zhangzhu people's Hospital |
| 376 | Shuyang people's Hospital |
| 377 | Siyang Hospital of traditional Chinese Medicine |
| 378 | Suqian people's Hospital |
| 379 | Jingtou hospital, Suyu District, Suqian City |
| 380 | Suqian Hospital of traditional Chinese Medicine |
| 381 | Suyu workers' Hospital |
| 382 | People's Hospital of Binhai County |
| 383 | Funing people's Hospital |
| 384 | Xiangshui Hospital of traditional Chinese Medicine |
| 385 | Yancheng first people's Hospital |
| 386 | Yancheng Red Cross Hospital |
| 387 | Yandu Second People's Hospital |
| 388 | Duchang maternal and child health care hospital |
| 389 | Jiangxi Gongqingcheng people's Hospital |
| 390 | Jiujiang first people's Hospital |
| 391 | Jiujiang maternal and child health care hospital |
| 392 | Jiujiang Hospital of traditional Chinese Medicine |
| 393 | Wuning maternal and child health care hospital |
| 394 | Jiangxi Provincial People's Hospital |
| 395 | The First Affiliated Hospital of Nanchang University |
| 396 | Nanchang eighth hospital (Nanchang Donghu hospital) |
| 397 | Nanchang Xihu District Hospital |
| 398 | Yingtan Hospital of Nanchang Railway Bureau |
| 399 | Nanchang people's Hospital |
| 400 | Luxi people's Hospital |
| 401 | General Hospital of Pingxiang Mining Group Co., Ltd |
| 402 | Pingxiang Anyuan District People's Hospital |
| 403 | Pingxiang people's Hospital |
| 404 | Shangli Hospital of traditional Chinese Medicine |
| 405 | Fenyi people's Hospital |
| 406 | Tiekeng Iron Mine Staff Hospital |
| 407 | Xinyu Sixth Hospital |
| 408 | Xinyu maternal and child health care hospital |
| 409 | Xinyu people's Hospital |
| 410 | Xinyu Hospital of traditional Chinese Medicine |
| 411 | Guixi silver mine staff hospital |
| 412 | Yingtan people's Hospital |
| 413 | Yingtan Yuehu maternal and child health care hospital |
| 414 | Chaoyang Second Hospital |
| 415 | Chaoyang communications hospital |
| 416 | Chaoyang Central Hospital |
| 417 | Staff hospital of Chaoyang Heavy Machinery Factory |
| 418 | Jianping County Hospital |
| 419 | Lingyuan first people's Hospital |
| 420 | Affiliated Zhongshan Hospital Dalian University |
| 421 | Dalian Economic and Technological Development Zone Hospital |
| 422 | Dalian Jinzhou maternal and child health care hospital |
| 423 | Dalian Lushunkou District Shedao hospital |
| 424 | Dalian Lushunkou traditional Chinese medicine hospital |
| 425 | Dalian Friendship Hospital |
| 426 | General Hospital of Fuxin Mining Bureau |
| 427 | Fuxin maternal and child health care hospital |
| 428 | Fuxin construction engineering hospital |
| 429 | Fuxin Xinqiu maternal and child health care hospital |
| 430 | fuxin central hospital |
| 431 | Zhangwu Hospital of traditional Chinese Medicine |
| 432 | Central Hospital of Yingkou Economic and Technological Development Zone |
| 433 | Yingkou Third People's Hospital |
| 434 | Yingkou maternal and child health care hospital |
| 435 | Yingkou staff hospital |
| 436 | Yingkou integrated traditional Chinese and Western Medicine Hospital |
| 437 | Yingkou Hospital of traditional Chinese Medicine |
| 438 | Bayannur League hospital |
| 439 | Bayannur League Hospital of traditional Chinese Medicine |
| 440 | Bayannur Third Hospital |
| 441 | Linhe people's Hospital |
| 442 | Wuyuan maternal and child health station |
| 443 | Baotou Donghe District Hospital |
| 444 | Qingshan District Hospital of Baotou City |
| 445 | Baotou Railway Hospital |
| 446 | Baotou Central Hospital |
| 447 | The First Affiliated Hospital of Baotou Medical College |
| 448 | Chifeng hospital |
| 449 | The first hospital of Yuanbaoshan District, Chifeng City |
| 450 | Linxi County Hospital |
| 451 | Ningcheng Hospital of traditional Chinese Medicine |
| 452 | Hohhot New District Maternal and child health care hospital |
| 453 | Hohhot China Mongolia Hospital |
| 454 | The First Affiliated Hospital of Inner Mongolia Medical College |
| 455 | Inner Mongolia Hospital |
| 456 | Kailu maternal and child health station |
| 457 | Affiliated Hospital of Inner Mongolia University for Nationalities |
| 458 | Tongliao Second People's Hospital |
| 459 | Tongliao hospital |
| 460 | Tongliao Hospital of traditional Chinese Medicine |
| 461 | Laoshidan Coal Mine Hospital of Haibowan Mining Bureau |
| 462 | Wuda Mining Bureau General Hospital |
| 463 | Wuhai people's Hospital |
| 464 | Guyuan people's Hospital |
| 465 | People's Hospital of Yuanzhou District, Guyuan City |
| 466 | Jingyuan County Hospital |
| 467 | Pengyang maternal and child health care hospital |
| 468 | Ningxia Coal General Hospital |
| 469 | Pingluo people's Hospital |
| 470 | Shizuishan Hospital of traditional Chinese Medicine |
| 471 | Taole maternal and child health care hospital |
| 472 | Qingtongxia people's Hospital |
| 473 | Wuzhong Hospital of traditional Chinese Medicine |
| 474 | Yanchi people's Hospital |
| 475 | Helan maternal and child health care center |
| 476 | Ningxia people's Hospital |
| 477 | Affiliated Hospital of Ningxia Medical University |
| 478 | Yinchuan railway staff hospital |
| 479 | Yinchuan rubber factory hospital |
| 480 | Yongning people's Hospital |
| 481 | Zhongwei maternal and child health care hospital |
| 482 | Zhongwei people's Hospital |
| 483 | Zhongwei Hospital of traditional Chinese Medicine |
| 484 | Zhongwei people's Hospital |
| 485 | The second people's Hospital of Haibei Tibetan Autonomous Prefecture |
| 486 | People's Hospital of Qilian County |
| 487 | Haidong people's Hospital |
| 488 | Ledu people's Hospital |
| 489 | Minhe Hospital of traditional Chinese Medicine |
| 490 | Ping'an maternal and child health care hospital |
| 491 | Guide county people's Hospital |
| 492 | Hainan Tibetan Autonomous Prefecture hospital |
| 493 | Tongde County Hospital |
| 494 | Xinghai hospital |
| 495 | Dulan County Hospital |
| 496 | Golmud maternal and child health care hospital |
| 497 | People's Hospital of Haixi Autonomous Prefecture |
| 498 | Wulanhaote integrated traditional Chinese and Western Medicine Hospital |
| 499 | Qinghai women's and children's Hospital |
| 500 | Qinghai Red Cross Hospital |
| 501 | Qinghai communications hospital |
| 502 | Qinghai Provincial People's Hospital |
| 503 | Qinghai Provincial Hospital of traditional Chinese Medicine |
| 504 | Xichuan Central People's Hospital |
| 505 | Xining first people's Hospital |
| 506 | Maternal and child health care hospital of Yushu Tibetan Autonomous Prefecture |
| 507 | People's Hospital of Yushu Tibetan Autonomous Prefecture |
| 508 | Zaduo County Hospital |
| 509 | Zhiduo County People's Hospital |
| 510 | Jinan Iron and Steel General Hospital |
| 511 | Jinan Fourth People's Hospital |
| 512 | Jinan National Hospital |
| 513 | Shandong Provincial Hospital |
| 514 | Zhangqiu maternal and child health care hospital |
| 515 | Donga people's Hospital |
| 516 | Guanxian Chengguan hospital |
| 517 | Liaocheng Dongchangfu people's Hospital |
| 518 | Liaocheng people's Hospital |
| 519 | Liaocheng Hospital of traditional Chinese Medicine |
| 520 | Jimo people's Hospital |
| 521 | Jiaonan maternal and child health care hospital |
| 522 | Jiaonan people's Hospital |
| 523 | Laixi maternal and child health care hospital |
| 524 | Qingdao Central Hospital |
| 525 | Dongping maternal and child health care hospital |
| 526 | Feicheng Hospital of traditional Chinese Medicine |
| 527 | Taishan maternal and Child Health Hospital |
| 528 | Tai'an Central Hospital |
| 529 | Tai'an Hospital of traditional Chinese Medicine |
| 530 | Tengzhou maternal and child health care hospital |
| 531 | Central Hospital of Zaozhuang Mining Group Company |
| 532 | Zaozhuang Municipal Hospital of traditional Chinese Medicine |
| 533 | People's Hospital of Shizhong District of Zaozhuang City |
| 534 | Zaozhuang Taierzhuang maternal and child health care hospital |
| 535 | Huantai people's Hospital |
| 536 | Central Hospital of Zibo Mining Bureau |
| 537 | Zibo Central Hospital |
| 538 | Zichuan District Hospital of Zibo City |
| 539 | Zichuan maternal and child health care hospital |
| 540 | General Hospital of Datong Coal Mine Group Company |
| 541 | Datong Second People's Hospital |
| 542 | Datong first people's Hospital |
| 543 | Datong Railway Central Hospital |
| 544 | Datong people's Hospital |
| 545 | Jincheng Coal Industry Group General Hospital |
| 546 | Jincheng maternal and Child Health Hospital |
| 547 | Jincheng people's Hospital |
| 548 | Qinshui maternal and child health station |
| 549 | Fushan people's Hospital |
| 550 | Huozhou people's Hospital |
| 551 | Linfen Fourth People's Hospital |
| 552 | Linfen people's Hospital |
| 553 | Linfen Railway Hospital |
| 554 | Quwo Hospital of traditional Chinese Medicine |
| 555 | Linxian people's Hospital |
| 556 | Luliang people's Hospital |
| 557 | Shanxi Fenyang hospital |
| 558 | Shilou people's Hospital |
| 559 | Wenshui people's Hospital |
| 560 | Zhongyang maternal and child health care hospital |
| 561 | Baode maternal and child health care hospital |
| 562 | Hequ Hospital of traditional Chinese Medicine |
| 563 | Ningwu maternal and child health care hospital |
| 564 | Xinzhou people's Hospital |
| 565 | Yuanping first people's Hospital |
| 566 | Yuanping Railway Hospital |
| 567 | Pingding people's Hospital |
| 568 | Hospital of Yangquan iron and Steel Company |
| 569 | Yangquan Coal Industry Group General Hospital |
| 570 | Yangquan first people's Hospital |
| 571 | Yangquan Hospital of traditional Chinese Medicine |
| 572 | Yuxian Hospital of traditional Chinese Medicine |
| 573 | Ankang first people's Hospital |
| 574 | Ankang maternal and child health care hospital |
| 575 | Ankang Central Hospital |
| 576 | Ankang Hospital of traditional Chinese Medicine |
| 577 | Shiquan maternal and child health care hospital |
| 578 | Luonan hospital |
| 579 | Shangluo Central Hospital |
| 580 | Shangnan Hospital of traditional Chinese Medicine |
| 581 | Zhenan maternal and child health station |
| 582 | Baishui people's Hospital |
| 583 | Tongguan Hospital of traditional Chinese Medicine |
| 584 | Weinan maternal and child health care hospital |
| 585 | Weinan Linwei maternal and child health care hospital |
| 586 | Weinan Central Hospital |
| 587 | Xijing Hospital of the Fourth Military Medical University |
| 588 | Shaanxi Provincial People's Hospital |
| 589 | Xi'an Chang'an maternal and child health care hospital |
| 590 | Ganquan people's Hospital |
| 591 | Huangling maternal and child health station |
| 592 | Luochuan maternal and child health care hospital |
| 593 | Affiliated Hospital of Medical College of Yan'an University |
| 594 | People's Hospital of Baota District, Yan'an City |
| 595 | Yan'an people's Hospital |
| 596 | Yulin Second Hospital |
| 597 | Yulin Xisha hospital |
| 598 | Yulin Yuyang maternal and child health care hospital |
| 599 | Yulin Hospital of traditional Chinese Medicine |
| 600 | Jinshan Hospital Affiliated to Fudan University |
| 601 | Zhongshan Hospital Affiliated to Fudan University |
| 602 | Huangpu District Central Hospital |
| 603 | Lujiazui Community Health Service Center |
| 604 | Lianyang District Hospital of Pudong New Area |
| 605 | Baoshan Branch of Shanghai First People's Hospital |
| 606 | The Sixth People's Hospital Affiliated to Shanghai Jiaotong University |
| 607 | The Ninth People's Hospital Affiliated to medical school of Shanghai Jiaotong University |
| 608 | Xinhua Hospital Affiliated to Medical College of Shanghai Jiaotong University |
| 609 | Shanghai Seventh People's Hospital |
| 610 | Shanghai Fifth People's Hospital |
| 611 | Shanghai Hongkou District Jiaxing district hospital |
| 612 | Zhujing District Hospital of Jinshan District, Shanghai |
| 613 | Shanghai Minhang District Central Hospital |
| 614 | Shanghai Putuo District Central Hospital |
| 615 | Shanghai Tongji Hospital |
| 616 | Shanghai Xuhui District Central Hospital |
| 617 | Shanghai Yangpu District Jiangpu district hospital |
| 618 | Shanghai Integrated Traditional Chinese and Western Medicine Hospital |
| 619 | Shanghai Hospital of traditional Chinese Medicine |
| 620 | Dinghai District Hospital of Yangpu District, Shanghai |
| 621 | Shuguang Hospital Affiliated to Shanghai University of traditional Chinese Medicine |
| 622 | Zhabei District Central Hospital |
| 623 | Changning maternal and Child Health Hospital |
| 624 | Changning Central Hospital |
| 625 | Dujiangyan people's Hospital |
| 626 | Pengzhou maternal and child health care hospital |
| 627 | Shuangliu first people's Hospital |
| 628 | West China Second Hospital of Sichuan University |
| 629 | Deyang maternal and child health care hospital |
| 630 | Deyang people's Hospital |
| 631 | Guanghan people's Hospital |
| 632 | Mianzhu maternal and child health care hospital |
| 633 | Zhongjiang maternal and child health care hospital |
| 634 | Guang'an people's Hospital |
| 635 | Linshui maternal and child health station |
| 636 | Wusheng maternal and child health care hospital |
| 637 | Wusheng County Hospital of traditional Chinese Medicine |
| 638 | Yuechi people's Hospital |
| 639 | Meishan Dongpo maternal and child health care hospital |
| 640 | Meishan people's Hospital |
| 641 | Meishan Hospital of traditional Chinese Medicine |
| 642 | Renshou maternal and Child Health Hospital |
| 643 | Renshou people's Hospital |
| 644 | Anxian maternal and child health care hospital |
| 645 | Jiangyou people's Hospital |
| 646 | Mianyang maternal and child health care hospital |
| 647 | Mianyang Central Hospital |
| 648 | Mianyang Hospital of traditional Chinese Medicine |
| 649 | Hanyuan people's Hospital |
| 650 | Shimian maternal and Child Health Hospital |
| 651 | Ya'an people's Hospital |
| 652 | Ya'an Hospital of traditional Chinese Medicine |
| 653 | Dagang Oilfield General Hospital |
| 654 | Jixian maternal and Child Health Hospital |
| 655 | Jinghai hospital |
| 656 | Tianjin Fifth Central Hospital |
| 657 | Tianjin Public Security Hospital |
| 658 | Tianjin Heping District Hospital of traditional Chinese Medicine |
| 659 | Tianjin Nankai hospital |
| 660 | Tianjin People's Hospital |
| 661 | Tianjin Tanggu hospital |
| 662 | Tianjin Xiqing hospital |
| 663 | Tianjin Medical University General Hospital |
| 664 | The First Affiliated Hospital of Tianjin University of traditional Chinese Medicine |
| 665 | The First Affiliated Hospital of Tianjin University of traditional Chinese Medicine |
| 666 | People's Hospital of Basu County |
| 667 | Changdu maternal and child health care hospital |
| 668 | Changdu people's Hospital |
| 669 | Leiwuqi people's Hospital |
| 670 | Lhasa maternal and child health care hospital |
| 671 | Lhasa People's Hospital |
| 672 | Tibetan Hospital of Tibet Autonomous Region |
| 673 | Gongbujiangda people's Hospital |
| 674 | Linzhi maternal and child health care hospital |
| 675 | People's Hospital of Milin County |
| 676 | Motuo people's Hospital |
| 677 | For example, the county people's hospital |
| 678 | Naqu maternal and child health care hospital |
| 679 | People's Hospital of Jiangzi County |
| 680 | Xigaze people's Hospital |
| 681 | Shannan Tibetan Hospital |
| 682 | Shannan people's Hospital |
| 683 | Altay people's Hospital |
| 684 | Buerjin maternal and child health care hospital |
| 685 | Fuyun people's Hospital |
| 686 | Qinghe maternal and child health station |
| 687 | People's Hospital of Barkol County |
| 688 | Hami maternal and child health care center |
| 689 | Hami people's Hospital |
| 690 | Hami Railway Hospital |
| 691 | Karamay Central Hospital |
| 692 | Karamay people's Hospital |
| 693 | Xinjiang Dushanzi Petrochemical hospital |
| 694 | The 14th regiment Hospital of the eighth Division |
| 695 | Staff hospital of the 14th and 3rd regiment of the eighth Division |
| 696 | The First Affiliated Hospital of Medical College of Shihezi University |
| 697 | Shihezi oasis hospital |
| 698 | Shihezi people's Hospital |
| 699 | Shanshan maternal and child health station |
| 700 | Shanshan people's Hospital |
| 701 | Turpan maternal and child health care hospital |
| 702 | Turpan people's Hospital |
| 703 | Urumqi Xishan Hospital |
| 704 | Urumqi Friendship Hospital |
| 705 | West Railway Station Hospital of Urumqi Railway Bureau |
| 706 | General Hospital of Xinjiang production and Construction Corps |
| 707 | Kunming Panlong District Maternal and child health care center |
| 708 | Kunming Hospital of traditional Chinese Medicine |
| 709 | Songming maternal and child health station |
| 710 | Yunnan hospital of traditional Chinese Medicine |
| 711 | Cangyuan maternal and child health care hospital |
| 712 | Gengma maternal and child health care hospital |
| 713 | Yongde people's Hospital |
| 714 | Lincang people's Hospital of Yunnan Province |
| 715 | People's Hospital of Pu'er County |
| 716 | Simao people's Hospital of Yunnan Province |
| 717 | Luliang maternal and child health care hospital |
| 718 | People's Hospital of Luoping County |
| 719 | Shizong maternal and child health care hospital |
| 720 | Tonghai maternal and child health care hospital |
| 721 | Yuxi people's Hospital |
| 722 | Yuxi Hospital of traditional Chinese Medicine |
| 723 | Daguan people's Hospital |
| 724 | Shuifu people's Hospital |
| 725 | Weixin maternal and child health station |
| 726 | Yongshan people's Hospital |
| 727 | Zhaotong people's Hospital |
| 728 | Zhaotong Hospital of traditional Chinese Medicine |
| 729 | Haining people's Hospital |
| 730 | Haiyan maternal and child health care hospital |
| 731 | Jiaxing Second Hospital |
| 732 | Jiaxing Xincheng people's Hospital |
| 733 | Jiaxing Hospital of traditional Chinese Medicine |
| 734 | Pinghu Hospital of traditional Chinese Medicine |
| 735 | Jinhua people's Hospital |
| 736 | Jinhua Central Hospital |
| 737 | Pan'an maternal and child health care hospital |
| 738 | Wuyi maternal and child health care hospital |
| 739 | Yiwu Hospital of traditional Chinese Medicine |
| 740 | Yongkang Hospital of traditional Chinese Medicine |
| 741 | Lishui people's Hospital |
| 742 | Lishui Central Hospital |
| 743 | Lishui Hospital of traditional Chinese Medicine |
| 744 | Qingtian people's Hospital |
| 745 | Qingyuan maternal and child health care center |
| 746 | Yunhe maternal and child health center |
| 747 | Changshan people's Hospital |
| 748 | Jiangshan people's Hospital |
| 749 | Kaihua maternal and child health care hospital |
| 750 | Longyou maternal and child health care hospital |
| 751 | Quzhou people's Hospital |
| 752 | Quzhou Hospital of traditional Chinese Medicine |
| 753 | Cangnan people's Hospital |
| 754 | Yishan Hospital of Cangnan County |
| 755 | Yueqing maternal and child health care hospital |
| 756 | Wenzhou Third People's Hospital |
| 757 | Wenzhou integrated traditional Chinese and Western Medicine Hospital |
| 758 | Yongjia people's Hospital |
| 759 | Daishan maternal and child health care center |
| 760 | Shengsi people's Hospital |
| 761 | Zhoushan people's Hospital of Zhejiang Province |
| 762 | Zhoushan maternal and Child Health Hospital |
| 763 | Zhoushan Putuo District People's Hospital |
| 764 | Zhoushan Puxi hospital |
| 765 | Bishan maternal and child health care hospital |
| 766 | Dianjiang maternal and child health care hospital |
| 767 | Southwest Hospital |
| 768 | Yongchuan maternal and child health care hospital |
| 769 | Chongqing Three Gorges Central Hospital |
| 770 | Chongqing Daping Hospital |
| 771 | Chongqing maternal and child health care hospital |
| 772 | Chongqing Jialing hospital |
| 773 | Chongqing Qianjiang Central Hospital |
| 774 | Chongqing Shapingba District People's Hospital |
| 775 | Chongqing Wansheng maternal and child health care hospital |
| 776 | Chongqing Wanzhou maternal and child health care hospital |
| 777 | Chongqing Wanzhou District People's Hospital |
| 778 | Chongqing Cancer Hospital |
| 779 | The First Affiliated Hospital of Chongqing Medical University |

# References

1. Website of National Health Commission of the People's Republic of China. http://www.nhc.gov.cn/xcs/yqfkdt/202008/ac1fb9f4a3a94e91ae86aaec7a6b44d6.shtml.
2. National Health Commission. Yearbook of Health Statistics of China—2019. Beijing: Peking Union Medical College Press; 2019.
3. Division of Urban Social and Economic Survey, National Bureau of Statistics. China City Statistical Yearbook一2019. Beijing: China Statistics Press; 2020.
4. https://www.ibm.com/support/knowledgecenter/SSLVMB_sub/statistics_mainhelp_ddita/spss/complex_samples/idh_idd_anal_wiz_design.html
5. https://www.publichealth.columbia.edu/research/population-health-methods/construction-complex-survey-weights

# Legend of supplementary tables

Table S1

Changes in clinical activities according to city categories. The categories of cities were based on the total number of hospital beds. In categories 1, 2 and 3 cities, the total numbers of beds were fewer than 10,000; 10,000 to 30,000; and more than 30,000, respectively. 95% CI, 95% confidence interval

Table S2

Changes in clinical activities according to hospital levels. 95% CI, 95% confidence interval.

Table S3

Changes in clinical activities according to hospital natures. 95% CI, 95% confidence interval.

Table S4

Changes in clinical activities according to provinces. 95% CI, 95% confidence interval.

Table S5

Changes in the management of specific diseases or conditions according to city categories. The categories of cities were based on the total number of hospital beds. In categories 1, 2 and 3 cities, the total numbers of beds were fewer than 10,000; 10,000 to 30,000; and more than 30,000, respectively. 95% CI, 95% confidence interval.

Table S6

Changes in the management of specific diseases or conditions according to hospital levels. 95% CI, 95% confidence interval.

Table S7

Changes in the management of specific diseases or conditions according to hospital natures. 95% CI, 95% confidence interval.

Table S8

Changes in the management of specific diseases or conditions according to provinces. 95% CI, 95% confidence interval.

# Table S1

Changes in clinical activities according to city categories. The categories of cities were based on the total number of hospital beds. In categories 1, 2 and 3 cities, the total numbers of beds were fewer than 10,000; 10,000 to 30,000; and more than 30,000, respectively. 95% CI, 95% confidence interval

|  | No. (%) of participants | During the imminent months of the pandemics (% [95% CI]) | | | *p* values |  | After July 1, 2020 (% [95% CI]) | | | *p* values |
| --- | --- | --- | --- | --- | --- | --- | --- | --- | --- | --- |
|  |  | Complete shutdown or >50% reduction | Reduction by 25%-50% | Reduction <25% or no change |  |  | Less than 2019 | Same as 2019 | More than 2019 than 2019 |  |
| Outpatient visits | |  |  |  | 0.046 |  |  |  |  | 0.003 |
| Category 1 | 1747 (83.0%) | 39.9 (35.8-44.1) | 32.8 (29.9-35.9) | 27.3 (23.4-31.6) |  |  | 43.8 (39.1-48.7) | 43.5 (39.7-47.3) | 12.7 (10.8-14.8) |  |
| Category 2 | 4970 (82.2%) | 44.7 (42.1-47.3) | 32.6 (30.6-34.8) | 22.7 (20.4-25.1) |  |  | 48.0 (45.4-50.7) | 42.9 (40.5-45.4) | 9.1 (8.0-10.3) |  |
| Category 3 | 2956 (80.9%) | 46.8 (43.6-50.1) | 32.6 (30.2-35.1) | 20.6 (18.2-23.2) |  |  | 51.4 (46.9-55.9) | 42.1 (38.0-46.3) | 6.5 (5.3-7.9) |  |
| Outpatient appointments | |  |  |  | 0.010 |  |  |  |  | 0.006 |
| Category 1 | 1412 (67.0%) | 21.1 (16.9-25.9) | 26.4 (23.5-29.4) | 52.6 (47.2-57.9) |  |  | 40.3 (34.9-45.9) | 47.3 (42.8-52.0) | 12.4 (10.7-14.3) |  |
| Category 2 | 3855 (63.7%) | 26.8 (24.5-29.2) | 25.3 (23.3-27.4) | 47.9 (44.8-51.0) |  |  | 43.6 (41.1-46.2) | 48.0 (45.5-50.6) | 8.4 (7.4-9.5) |  |
| Category 3 | 2252 (61.7%) | 29.8 (26.3-33.4) | 27.0 (25.0-29.1) | 43.2 (39.9-46.6) |  |  | 46.5 (42.5-50.6) | 46.9 (43.5-50.4) | 6.5 (5.4-8.0) |  |
| Surgical volumes | |  |  |  | 0.006 |  |  |  |  | 0.053 |
| Category 1 | 1651 (78.4%) | 25.3 (20.3-31.0) | 28.3 (24.3-32.8) | 46.4 (38.6-54.3) |  |  | 46.5 (41.4-51.7) | 42.6 (38.9-46.3) | 10.9 (8.7-13.6) |  |
| Category 2 | 4906 (81.1%) | 27.8 (25.5-30.1) | 31.6 (29.3-33.9) | 40.7 (37.7-43.7) |  |  | 47.6 (44.7-50.5) | 43.9 (41.3-46.5) | 8.5 (7.6-9.6) |  |
| Category 3 | 2841 (77.8%) | 33.9 (30.2-37.8) | 30.1 (27.5-32.8) | 36.1 (33.5-38.7) |  |  | 51.3 (47.1-55.6) | 42.1 (37.9-46.4) | 6.6 (5.3-8.2) |  |
| Consultation request | |  |  |  | 0.001 |  |  |  |  | 0.037 |
| Category 1 | 1511 (71.7%) | 16.8 (13.6-20.6) | 19.6 (16.4-23.4) | 63.6 (57.7-69.0) |  |  | 38.4 (33.4-43.7) | 51.1 (46.8-55.4) | 10.5 (8.8-12.3) |  |
| Category 2 | 4168 (68.9%) | 20.7 (18.5-23.1) | 17.2 (15.6-19.0) | 62.1 (59.2-64.9) |  |  | 38.6 (35.6-41.6) | 54.5 (51.4-57.5) | 7.0 (6.0-8.1) |  |
| Category 3 | 2148 (58.8%) | 25.8 (22.5-29.3) | 19.1 (16.8-21.5) | 55.2 (52.0-58.3) |  |  | 41.3 (36.4-46.3) | 53.7 (48.3-59.0) | 5.1 (3.8-6.7) |  |
| Admission arrangements | |  |  |  | 0.002 |  |  |  |  | 0.005 |
| Category 1 | 1675 (79.5%) | 21.2 (17.2-25.8) | 28.3 (23.9-33.3) | 50.5 (43.5-57.4) |  |  | 43.9 (38.7-49.2) | 43.1 (39.8-46.6) | 13.0 (10.4-16.2) |  |
| Category 2 | 4788 (79.2%) | 24.8 (22.4-27.4) | 30.3 (28.5-32.1) | 44.9 (41.9-47.9) |  |  | 47.7 (44.9-50.4) | 43.8 (41.4-46.3) | 8.5 (7.3-9.8) |  |
| Category 3 | 2717 (74.4%) | 31.6 (27.6-35.9) | 28.1 (24.8-31.7) | 40.3 (37.2-43.4) |  |  | 49.9 (45.4-54.4) | 43.8 (39.7-48.0) | 6.2 (4.9-7.9) |  |
| Emergency visits | |  |  |  | 0.204 |  |  |  |  | 0.100 |
| Category 1 | 1261 (59.9%) | 17.8 (14.1-22.1) | 24.7 (21.2-28.7) | 57.5 (52.1-62.7) |  |  | 41.2 (36.4-46.1) | 47.7 (43.6-51.7) | 11.2 (9.4-13.2) |  |
| Category 2 | 3514 (58.1%) | 19.6 (17.7-21.8) | 24.9 (22.7-27.4) | 55.4 (52.4-58.3) |  |  | 42.2 (39.4-45.0) | 48.4 (45.6-51.3) | 9.4 (8.1-10.8) |  |
| Category 3 | 1988 (54.4%) | 22.7 (19.2-26.6) | 26.1 (22.2-30.5) | 51.1 (46.7-55.6) |  |  | 42.5 (37.2-48.1) | 50.8 (45.6-56.1) | 6.6 (5.3-8.3) |  |
| Online consultations^*^ | |  |  |  | 0.003 |  |  |  |  | 0.294 |
| Category 1 | 943 (44.8%) | 15.8 (13.1-18.9) | 39.9 (36.0-43.9) | 44.3 (40.0-48.7) |  |  | 30.7 (26.3-35.5) | 47.2 (43.1-51.3) | 22.1 (19.3-25.3) |  |
| Category 2 | 2771 (45.8%) | 16.6 (14.9-18.5) | 31.7 (29.1-34.4) | 51.7 (48.7-54.6) |  |  | 29.9 (27.6-32.4) | 49.6 (46.1-53.1) | 20.5 (18.4-22.8) |  |
| Category 3 | 1517 (41.5%) | 19.4 (16.4-22.9) | 27.2 (24.2-30.4) | 53.4 (48.3-58.3) |  |  | 29.7 (26.5-33.1) | 46.2 (42.1-50.3) | 24.1 (21.1-27.3) |  |

^*^ The three percentage values during the imminent months of the pandemics denote reduction, no change and increasing.

# Table S2

Changes in clinical activities according to hospital levels. 95% CI, 95% confidence interval.

|  | No. (%) of participants | During the imminent months of the pandemics (% [95% CI]) | | | *p* values |  | After July 1, 2020 (% [95% CI]) | | | *p* values |
| --- | --- | --- | --- | --- | --- | --- | --- | --- | --- | --- |
|  |  | Complete shutdown or >50% reduction | Reduction by 25%-50% | Reduction <25% or no change |  |  | Less than 2019 | Same as 2019 | More than 2019 than 2019 |  |
| Outpatient visits | |  |  |  | <0.001 |  |  |  |  | 0.184 |
| Primary | 1580 (82.4%) | 38.5 (34.7-42.4) | 35.6 (32.6-38.7) | 25.9 (22.6-29.5) |  |  | 50.0 (44.6-55.3) | 41.3 (36.1-46.6) | 8.7 (7.2-10.6) |  |
| Secondary | 3008 (82.1%) | 43.7 (40.9-46.5) | 33.4 (30.8-36.1) | 22.9 (20.4-25.6) |  |  | 50.2 (46.5-53.9) | 40.5 (36.9-44.1) | 9.4 (8.0-11.0) |  |
| Tertiary | 5085 (81.7%) | 51.1 (48.1-54.1) | 29.8 (28.3-31.3) | 19.1 (16.9-21.6) |  |  | 47.3 (42.9-51.8) | 45.7 (41.9-49.5) | 7.0 (6.0-8.2) |  |
| Outpatient appointments | |  |  |  | <0.001 |  |  |  |  | 0.018 |
| Primary | 1228 (64.0%) | 21.2 (18.0-24.9) | 26.6 (23.2-30.3) | 52.2 (48.0-56.5) |  |  | 46.7 (41.7-51.7) | 46.4 (41.0-51.9) | 6.9 (5.5-8.6) |  |
| Secondary | 2327 (63.5%) | 25.2 (22.3-28.4) | 27.0 (24.6-29.4) | 47.8 (44.6-51.1) |  |  | 46.3 (42.4-50.3) | 44.0 (40.5-47.5) | 9.7 (8.5-11.1) |  |
| Tertiary | 3964 (63.7%) | 33.8 (30.7-37.1) | 25.2 (23.3-27.2) | 41.0 (38.6-43.5) |  |  | 41.3 (37.5-45.2) | 51.5 (48.3-54.6) | 7.2 (6.2-8.4) |  |
| Surgical volumes | |  |  |  | <0.001 |  |  |  |  | 0.091 |
| Primary | 1481 (77.2%) | 22.9 (19.9-26.3) | 31.0 (28.1-33.9) | 46.1 (42.6-49.7) |  |  | 51.4 (44.4-58.2) | 41.5 (35.0-48.3) | 7.2 (5.9-8.6) |  |
| Secondary | 2867 (78.2%) | 28.6 (25.7-31.7) | 32.8 (30.1-35.5) | 38.6 (35.0-42.4) |  |  | 50.5 (46.6-54.4) | 40.1 (36.7-43.6) | 9.4 (7.7-11.5) |  |
| Tertiary | 5050 (81.2%) | 36.5 (32.6-40.6) | 28.2 (25.6-31.0) | 35.3 (32.6-38.1) |  |  | 46.4 (43.1-49.7) | 46.6 (43.5-49.7) | 7.1 (6.2-8.0) |  |
| Consultation requests | |  |  |  | <0.001 |  |  |  |  | 0.429 |
| Primary | 1284 (66.9%) | 15.4 (12.8-18.3) | 15.4 (13.4-17.7) | 69.2 (66.0-72.3) |  |  | 41.8 (36.0-47.9) | 52.1 (46.0-58.2) | 6.1 (4.8-7.6) |  |
| Secondary | 2527 (68.9%) | 21.1 (18.8-23.8) | 19.6 (17.3-22.1) | 59.2 (56.0-62.4) |  |  | 39.9 (36.2-43.7) | 52.7 (48.7-56.7) | 7.4 (6.1-8.9) |  |
| Tertiary | 4016 (64.5%) | 28.8 (25.9-31.9) | 19.0 (17.4-20.8) | 52.2 (49.1-55.3) |  |  | 37.7 (33.2-42.5) | 56.1 (52.0-60.1) | 6.2 (5.2-7.3) |  |
| Admission arrangements | |  |  |  | <0.001 |  |  |  |  | 0.119 |
| Primary | 1418 (73.9%) | 19.7 (16.7-23.1) | 30.2 (25.8-34.9) | 50.1 (45.2-55.0) |  |  | 49.4 (42.1-56.7) | 43.1 (36.1-50.3) | 7.6 (6.1-9.4) |  |
| Secondary | 2861 (78.0%) | 25.3 (22.1-28.9) | 30.7 (27.7-33.7) | 44.0 (40.6-47.5) |  |  | 50.0 (46.3-53.8) | 40.5 (37.7-43.5) | 9.4 (7.7-11.6) |  |
| Tertiary | 4901 (78.8%) | 34.3 (30.8-38.0) | 26.9 (24.8-29.2) | 38.8 (36.1-41.5) |  |  | 45.7 (42.7-48.8) | 47.3 (44.7-49.9) | 7.0 (5.9-8.3) |  |
| Emergency visits | |  |  |  | 0.070 |  |  |  |  | 0.012 |
| Primary | 1056 (55.1%) | 17.3 (14.7-20.3) | 25.7 (21.8-30.0) | 57.0 (52.1-61.8) |  |  | 46.6 (40.8-52.4) | 45.5 (39.4-51.6) | 8.0 (6.2-10.3) |  |
| Secondary | 2121 (57.9%) | 20.6 (17.1-24.6) | 24.7 (22.3-27.2) | 54.7 (51.0-58.3) |  |  | 42.7 (38.3-47.2) | 47.4 (43.3-51.6) | 9.9 (8.2-11.8) |  |
| Tertiary | 3586 (57.6%) | 23.3 (20.4-26.4) | 26.0 (23.3-28.9) | 50.7 (47.6-53.8) |  |  | 38.9 (35.8-42.0) | 53.9 (51.1-56.8) | 7.2 (6.0-8.5) |  |
| Online consultations^*^ | |  |  |  | 0.340 |  |  |  |  | 0.005 |
| Primary | 887 (46.2%) | 17.5 (14.4-21.2) | 32.3 (28.1-36.8) | 50.2 (44.6-55.8) |  |  | 35.3 (30.1-40.8) | 46.2 (39.7-52.9) | 18.5 (15.4-22.0) |  |
| Secondary | 1585 (43.2%) | 17.7 (15.5-20.1) | 32.1 (29.3-35.1) | 50.2 (46.7-53.7) |  |  | 28.2 (25.5-31.1) | 45.7 (41.6-49.9) | 26.1 (23.0-29.4) |  |
| Tertiary | 2759 (44.3%) | 18.0 (15.6-20.6) | 28.1 (25.5-30.9) | 53.9 (50.6-57.2) |  |  | 27.7 (25.4-30.2) | 51.0 (48.2-53.9) | 21.2 (18.8-23.9) |  |

^*^ The three percentage values during the imminent months of the pandemics denote reduction, no change and increasing.

# Table S3

Changes in clinical activities according to hospital natures. 95% CI, 95% confidence interval.

|  | No. (%) of participants | During the imminent months of the pandemics (% [95% CI]) | | | *p* values |  | After July 1, 2020 (% [95% CI]) | | | *p* values |
| --- | --- | --- | --- | --- | --- | --- | --- | --- | --- | --- |
|  |  | Complete shutdown or >50% reduction | Reduction by 25%-50% | Reduction <25% or no change |  |  | Less than 2019 | Same as 2019 | More than 2019 than 2019 |  |
| Outpatient visits | |  |  |  | 0.002 |  |  |  |  | 0.046 |
| General | 7607 (82.2%) | 47.8 (45.8-49.8) | 32.3 (30.6-34.0) | 19.9 (18.3-21.7) |  |  | 48.1 (45.9-50.3) | 43.4 (41.3-45.5) | 8.5 (7.7-9.4) |  |
| Specialized | 2066 (80.8%) | 38.4 (32.6-44.6) | 33.5 (30.7-36.5) | 28.1 (23.5-33.1) |  |  | 51.5 (45.5-57.4) | 40.7 (35.6-46.0) | 7.9 (6.1-10.1) |  |
| Outpatient appointments | |  |  |  | 0.002 |  |  |  |  | 0.712 |
| General | 5939 (64.2%) | 29.8 (27.8-31.8) | 27.0 (25.4-28.7) | 43.2 (41.1-45.4) |  |  | 44.4 (42.0-46.8) | 47.3 (45.1-49.5) | 8.3 (7.5-9.2) |  |
| Specialized | 1580 (61.8%) | 21.6 (16.5-27.9) | 24.1 (21.7-26.6) | 54.3 (48.5-60.0) |  |  | 44.8 (39.7-50.0) | 47.8 (42.8-52.9) | 7.4 (6.0-9.1) |  |
| Surgical volumes | |  |  |  | <0.001 |  |  |  |  | 0.604 |
| General | 7485 (80.9%) | 33.8 (31.4-36.3) | 31.4 (29.5-33.4) | 34.7 (32.8-36.7) |  |  | 48.5 (46.2-50.8) | 43.6 (41.6-45.7) | 7.9 (6.9-9.0) |  |
| Specialized | 1913 (74.8%) | 21.1 (16.3-26.8) | 28.3 (25.7-31.0) | 50.7 (45.0-56.3) |  |  | 50.7 (44.2-57.2) | 41.2 (35.4-47.3) | 8.1 (6.7-9.8) |  |
| Consultation requests | |  |  |  | <0.001 |  |  |  |  | 0.357 |
| General | 6250 (67.6%) | 24.9 (23.0-26.9) | 19.4 (17.9-20.9) | 55.7 (53.6-57.9) |  |  | 38.6 (36.4-40.8) | 54.7 (52.4-56.9) | 6.7 (5.9-7.7) |  |
| Specialized | 1577 (61.7%) | 15.6 (11.5-20.8) | 15.4 (13.4-17.7) | 69.0 (63.5-74.0) |  |  | 42.4 (35.8-49.4) | 51.3 (44.6-58.0) | 6.2 (4.8-8.0) |  |
| Admission arrangements | |  |  |  | 0.001 |  |  |  |  | 0.886 |
| General | 7316 (79.1%) | 30.3 (28.1-32.7) | 29.7 (28.0-31.5) | 39.9 (37.9-42.0) |  |  | 48.2 (46.0-50.5) | 43.6 (41.7-45.5) | 8.2 (7.2-9.3) |  |
| Specialized | 1864 (72.9%) | 19.5 (14.0-26.6) | 27.5 (23.7-31.7) | 52.9 (46.6-59.1) |  |  | 48.3 (41.2-55.4) | 44.1 (37.9-50.6) | 7.6 (5.8-9.9) |  |
| Emergency visits | |  |  |  | <0.001 |  |  |  |  | 0.808 |
| General | 5423 (58.6%) | 22.9 (20.6-25.5) | 26.1 (24.0-28.5) | 50.9 (48.2-53.6) |  |  | 41.9 (39.2-44.6) | 49.5 (47.0-52.0) | 8.6 (7.5-9.8) |  |
| Specialized | 1340 (52.4%) | 14.9 (12.3-18.0) | 23.6 (20.3-27.2) | 61.5 (56.8-66.0) |  |  | 43.1 (37.6-48.8) | 49.0 (43.3-54.8) | 7.8 (6.0-10.1) |  |
| Online consultations^*^ | |  |  |  | 0.249 |  |  |  |  | 0.572 |
| General | 4147 (44.8%) | 18.7 (16.8-20.8) | 30.6 (28.6-32.7) | 50.7 (47.9-53.4) |  |  | 29.4 (27.6-31.4) | 47.7 (45.2-50.2) | 22.8 (20.8-25.0) |  |
| Specialized | 1084 (42.4%) | 15.4 (12.1-19.4) | 30.7 (26.8-35.0) | 53.9 (49.0-58.7) |  |  | 31.1 (26.4-36.3) | 48.2 (42.2-54.2) | 20.6 (17.7-23.9) |  |

^*^ The three percentage values during the imminent months of the pandemics denote reduction, no change and increasing.

# Table S4

Changes in clinical activities according to provinces. 95% CI, 95% confidence interval.

|  | No. (%) of participants | During the imminent months of the pandemics (% [95% CI]) | | | *p* values |  | After July 1, 2020 (% [95% CI]) | | | *p* values |
| --- | --- | --- | --- | --- | --- | --- | --- | --- | --- | --- |
|  |  | Complete shutdown or >50% reduction | Reduction by 25%-50% | Reduction <25% or no change |  |  | Less than 2019 | Same as 2019 | More than 2019 than 2019 |  |
| Outpatient visits | |  |  |  | 0.003 |  |  |  |  | 0.247 |
| Hubei | 302 (80.3%) | 60.8 (50.1-70.5) | 18.2 (12.6-25.4) | 21.1 (13.3-31.6) |  |  | 54.6 (45.6-63.4) | 36.4 (28.4-45.2) | 8.9 (6.3-12.5) |  |
| Others | 9371 (82.0%) | 44.4 (42.5-46.4) | 33.2 (31.7-34.8) | 22.3 (20.7-24.0) |  |  | 48.8 (46.2-51.4) | 42.9 (40.6-45.2) | 8.3 (7.5-9.2) |  |
| Outpatient appointments | |  |  |  | 0.236 |  |  |  |  | 0.259 |
| Hubei | 245 (65.2%) | 35.1 (26.4-44.8) | 20.5 (13.4-30.1) | 44.4 (31.8-57.7) |  |  | 52.1 (40.1-63.9) | 40.1 (29.8-51.3) | 7.8 (4.9-12.3) |  |
| Others | 7274 (63.6%) | 27.1 (25.1-29.2) | 26.4 (25.1-27.8) | 46.5 (44.4-48.6) |  |  | 44.2 (41.9-46.5) | 47.8 (45.7-49.8) | 8.1 (7.3-8.9) |  |
| Surgical volumes | |  |  |  | 0.506 |  |  |  |  | 0.762 |
| Hubei | 337 (89.6%) | 34.1 (26.6-42.6) | 25.9 (18.6-34.9) | 40.0 (27.0-54.5) |  |  | 52.3 (40.4-64.0) | 39.8 (29.9-50.7) | 7.9 (4.4-13.7) |  |
| Others | 9061 (79.3%) | 30.0 (28.0-32.1) | 30.8 (29.1-32.5) | 39.2 (37.3-41.2) |  |  | 49.0 (46.5-51.5) | 43.1 (40.8-45.5) | 7.9 (7.1-8.8) |  |
| Consultation requests | |  |  |  | 0.160 |  |  |  |  | 0.298 |
| Hubei | 295 (78.5%) | 28.4 (23.0-34.4) | 16.7 (11.7-23.2) | 55.0 (46.1-63.5) |  |  | 46.2 (35.2-57.6) | 46.4 (34.7-58.6) | 7.4 (4.6-11.6) |  |
| Others | 7532 (65.9%) | 22.0 (20.2-23.9) | 18.4 (17.0-19.8) | 59.7 (57.6-61.7) |  |  | 39.3 (36.6-42.1) | 54.1 (51.3-56.9) | 6.6 (5.8-7.4) |  |
| Admission arrangements | |  |  |  | 0.790 |  |  |  |  | 0.160 |
| Hubei | 331 (88.0%) | 27.1 (18.8-37.4) | 27.2 (22.8-32.1) | 45.6 (35.2-56.5) |  |  | 54.7 (45.7-63.4) | 36.1 (27.4-45.7) | 9.2 (6.3-13.2) |  |
| Others | 8849 (77.4%) | 27.4 (25.1-29.8) | 29.2 (27.3-31.2) | 43.4 (41.2-45.6) |  |  | 47.9 (45.4-50.4) | 44.1 (41.9-46.4) | 8.0 (7.0-9.0) |  |
| Emergency visits | |  |  |  | 0.537 |  |  |  |  | 0.385 |
| Hubei | 236 (62.8%) | 25.0 (18.5-32.8) | 24.2 (16.8-33.4) | 50.8 (39.5-62.1) |  |  | 48.1 (38.6-57.6) | 43.7 (34.5-53.3) | 8.3 (4.8-13.7) |  |
| Others | 6527 (57.1%) | 20.6 (18.7-22.6) | 25.5 (23.3-27.9) | 53.9 (51.3-56.5) |  |  | 41.9 (39.1-44.8) | 49.7 (46.9-52.5) | 8.4 (7.4-9.4) |  |
| Online consultations^*^ | |  |  |  | 0.015 |  |  |  |  | 0.129 |
| Hubei | 188 (50.0%) | 25.7 (19.3-33.4) | 23.7 (17.9-30.6) | 50.6 (47.2-54.0) |  |  | 39.1 (31.9-46.8) | 42.1 (31.0-54.1) | 18.8 (12.6-27.1) |  |
| Others | 5043 (44.1%) | 17.4 (15.7-19.2) | 31.0 (29.0-33.1) | 51.6 (48.8-54.5) |  |  | 29.5 (27.6-31.5) | 48.1 (45.6-50.7) | 22.4 (20.7-24.2) |  |

^*^ The three percentage values during the imminent months of the pandemics denote reduction, no change and increasing.

# Table S5

Changes in the management of specific diseases or conditions according to city categories. The categories of cities were based on the total number of hospital beds. In categories 1, 2 and 3 cities, the total numbers of beds were fewer than 10,000; 10,000 to 30,000; and more than 30,000, respectively. 95% CI, 95% confidence interval.

|  | No. (%) of participants | Complete shutdown or >50% reduction, (% [95% CI]) | Reduction by 25%-50%, (% [95% CI]) | Reduction <25% or no change, (% [95% CI]) | P value for χ^2^ test |
| --- | --- | --- | --- | --- | --- |
| Preconception counseling | |  |  |  | 0.012 |
| Category 1 | 1593 (75.6%) | 30.6 (27.2-34.3) | 29.7 (27.0-32.6) | 39.6 (36.4-43.0) |  |
| Category 2 | 4432 (73.3%) | 36.1 (33.8-38.5) | 32.3 (30.5-34.2) | 31.5 (29.6-33.6) |  |
| Category 3 | 2216 (60.7%) | 33.3 (30.3-36.5) | 31.4 (28.7-34.3) | 35.2 (32.4-38.1) |  |
| Prenatal examinations | |  |  |  | 0.005 |
| Category 1 | 1588 (75.4%) | 26.1 (23.2-29.3) | 30.7 (28.1-33.4) | 43.2 (40.0-46.5) |  |
| Category 2 | 4411 (72.9%) | 30.0 (27.4-32.7) | 32.3 (30.4-34.2) | 37.8 (35.3-40.3) |  |
| Category 3 | 2219 (60.8%) | 23.6 (20.2-27.3) | 32.6 (29.3-36.2) | 43.8 (41.3-46.3) |  |
| Prenatal diagnosis |  |  |  |  | 0.001 |
| Category 1 | 1373 (65.2%) | 24.7 (21.7-27.9) | 26.2 (23.7-28.8) | 49.2 (46.0-52.3) |  |
| Category 2 | 3753 (62.1%) | 30.7 (28.0-33.4) | 27.9 (25.9-30.1) | 41.4 (38.5-44.4) |  |
| Category 3 | 1793 (49.1%) | 24.6 (21.7-27.7) | 28.6 (26.2-31.2) | 46.8 (43.8-49.7) |  |
| Instructions for contraception and miscarriage | |  |  |  | 0.193 |
| Category 1 | 1617 (76.8%) | 25.2 (22.3-28.5) | 24.6 (21.3-28.3) | 50.2 (46.2-54.1) |  |
| Category 2 | 4342 (71.8%) | 28.9 (26.6-31.3) | 24.4 (22.6-26.2) | 46.7 (44.2-49.3) |  |
| Category 3 | 2190 (60.0%) | 30.3 (27.0-33.8) | 21.9 (19.6-24.3) | 47.8 (45.0-50.6) |  |
| Assistant reproduction | |  |  |  | 0.014 |
| Category 1 | 824 (39.1%) | 30.6 (25.7-36.0) | 21.1 (18.0-24.6) | 48.3 (43.1-53.5) |  |
| Category 2 | 2120 (35.1%) | 41.3 (37.9-44.7) | 20.3 (18.0-22.8) | 38.4 (34.9-42.0) |  |
| Category 3 | 927 (25.4%) | 36.3 (31.8-41.2) | 23.3 (19.6-27.6) | 40.3 (36.7-44.0) |  |
| Outpatient surgeries and procedures | |  |  |  | 0.099 |
| Category 1 | 1469 (69.8%) | 29.3 (25.6-33.4) | 27.1 (22.5-32.3) | 43.6 (37.6-49.7) |  |
| Category 2 | 3770 (62.3%) | 35.7 (33.2-38.2) | 26.8 (24.8-29.0) | 37.5 (34.9-40.2) |  |
| Category 3 | 1957 (53.6%) | 37.2 (33.6-40.9) | 26.3 (23.6-29.2) | 36.5 (33.6-39.5) |  |
| Emergent obstetrical surgeries | |  |  |  | 0.835 |
| Category 1 | 1450 (68.9%) | 16.3 (13.3-19.9) | 19.0 (16.2-22.1) | 64.7 (59.8-69.2) |  |
| Category 2 | 4051 (67.0%) | 16.0 (13.8-18.4) | 21.5 (19.6-23.5) | 62.5 (59.4-65.6) |  |
| Category 3 | 2041 (55.9%) | 15.5 (11.7-20.2) | 20.3 (17.5-23.5) | 64.2 (59.9-68.2) |  |
| Emergent gynecological surgeries | |  |  |  | 0.851 |
| Category 1 | 1475 (70.0%) | 20.7 (16.9-25.1) | 19.6 (16.0-23.7) | 59.7 (53.2-65.9) |  |
| Category 2 | 3982 (65.8%) | 21.9 (19.8-24.2) | 20.1 (18.3-22.1) | 57.9 (55.2-60.6) |  |
| Category 3 | 2118 (58.0%) | 20.0 (15.8-25.0) | 20.6 (17.6-23.9) | 59.4 (55.9-62.8) |  |
| Cervical cancer screening | |  |  |  | 0.017 |
| Category 1 | 1439 (68.3%) | 30.7 (27.9-33.7) | 20.5 (17.2-24.3) | 48.8 (44.3-53.3) |  |
| Category 2 | 3914 (64.7%) | 38.3 (36.1-40.5) | 21.5 (20.0-23.2) | 40.2 (37.8-42.5) |  |
| Category 3 | 2081 (57.0%) | 38.2 (34.2-42.3) | 22.8 (20.2-25.7) | 39.0 (35.9-42.2) |  |
| Treatment for benign neoplasm | |  |  |  | 0.019 |
| Category 1 | 1388 (65.9%) | 29.7 (25.7-34.0) | 24.2 (20.4-28.5) | 46.1 (40.0-52.3) |  |
| Category 2 | 3818 (63.1%) | 36.1 (33.7-38.6) | 23.2 (21.2-25.2) | 40.7 (38.0-43.5) |  |
| Category 3 | 2072 (56.7%) | 36.9 (32.9-41.0) | 26.5 (23.5-29.8) | 36.6 (34.2-39.1) |  |
| Treatment for malignancies | |  |  |  | 0.873 |
| Category 1 | 1246 (59.2%) | 23.9 (20.1-28.0) | 21.1 (18.5-24.0) | 55.0 (51.2-58.8) |  |
| Category 2 | 3499 (57.9%) | 26.2 (23.8-28.8) | 19.8 (18.1-21.7) | 54.0 (51.4-56.5) |  |
| Category 3 | 1880 (51.5%) | 26.0 (21.7-30.7) | 20.2 (18.1-22.4) | 53.8 (49.4-58.3) |  |
| Follow-up for malignancies | |  |  |  | 0.706 |
| Category 1 | 1240 (58.9%) | 24.4 (20.6-28.6) | 20.1 (17.7-22.8) | 55.5 (50.8-60.1) |  |
| Category 2 | 3427 (56.7%) | 27.0 (24.7-29.4) | 18.8 (17.3-20.4) | 54.2 (51.6-56.8) |  |
| Category 3 | 1825 (50.0%) | 26.7 (22.9-30.9) | 20.3 (18.0-22.8) | 53.0 (49.3-56.7) |  |
| Treatment for pelvic floor dysfunctions | |  |  |  | 0.018 |
| Category 1 | 1308 (62.1%) | 33.4 (30.7-36.3) | 23.1 (20.9-25.5) | 43.5 (40.2-46.8) |  |
| Category 2 | 3525 (58.3%) | 39.2 (36.8-41.5) | 21.1 (19.4-22.9) | 39.8 (37.4-42.2) |  |
| Category 3 | 1718 (47.0%) | 41.8 (38.9-44.7) | 21.9 (19.2-24.8) | 36.3 (33.0-39.8) |  |

# Table S6

Changes in the management of specific diseases or conditions according to hospital levels. 95% CI, 95% confidence interval.

|  | No. (%) of participants | Complete shutdown or >50% reduction, (% [95% CI]) | Reduction by 25%-50%, (% [95% CI]) | Reduction <25% or no change, (% [95% CI]) | P value for χ^2^ test |
| --- | --- | --- | --- | --- | --- |
| Preconception counseling | |  |  |  | 0.116 |
| Primary | 1477 (77.0%) | 36.4 (32.4-40.6) | 29.4 (26.7-32.3) | 34.2 (30.7-37.8) |  |
| Secondary | 2649 (72.3%) | 31.6 (28.7-34.7) | 34.1 (31.2-37.2) | 34.2 (32.0-36.6) |  |
| Tertiary | 4115 (66.1%) | 35.6 (32.8-38.5) | 30.8 (28.7-32.9) | 33.6 (31.2-36.1) |  |
| Prenatal examinations | |  |  |  | 0.830 |
| Primary | 1484 (77.4%) | 27.7 (24.1-31.5) | 30.9 (27.6-34.5) | 41.4 (37.9-45.0) |  |
| Secondary | 2654 (72.4%) | 26.0 (23.2-29.1) | 33.2 (30.1-36.5) | 40.7 (38.2-43.3) |  |
| Tertiary | 4080 (65.6%) | 27.4 (24.0-31.0) | 32.2 (30.3-34.1) | 40.4 (37.8-43.1) |  |
| Prenatal diagnosis |  |  |  |  | 0.804 |
| Primary | 1245 (64.9%) | 27.6 (24.1-31.3) | 26.7 (23.7-29.9) | 45.7 (41.4-50.1) |  |
| Secondary | 2247 (61.3%) | 27.0 (24.2-30.0) | 28.5 (26.1-31.1) | 44.5 (41.7-47.3) |  |
| Tertiary | 3427 (55.1%) | 28.3 (25.3-31.5) | 28.4 (26.6-30.3) | 43.3 (40.7-45.9) |  |
| Instructions for contraception and miscarriage | |  |  |  | <0.001 |
| Primary | 1461 (76.2%) | 25.6 (22.4-29.0) | 22.9 (20.6-25.3) | 51.6 (47.8-55.4) |  |
| Secondary | 2634 (71.8%) | 27.1 (24.2-30.3) | 23.4 (21.0-25.9) | 49.5 (46.9-52.2) |  |
| Tertiary | 4054 (65.2%) | 33.9 (31.3-36.6) | 23.9 (22.2-25.6) | 42.2 (39.8-44.7) |  |
| Assistant reproduction | |  |  |  | 0.044 |
| Primary | 679 (35.4%) | 37.4 (33.0-41.9) | 20.0 (16.6-24.0) | 42.6 (37.6-47.7) |  |
| Secondary | 1154 (31.5%) | 35.3 (31.7-39.2) | 21.0 (18.3-23.9) | 43.7 (39.2-48.3) |  |
| Tertiary | 2038 (32.8%) | 40.9 (37.1-44.9) | 23.1 (19.7-27.0) | 35.9 (33.4-38.6) |  |
| Outpatient surgeries and procedures | |  |  |  | <0.001 |
| Primary | 1247 (65.0%) | 31.8 (28.0-35.8) | 23.9 (21.0-27.2) | 44.3 (39.4-49.3) |  |
| Secondary | 2282 (62.2%) | 31.6 (28.2-35.1) | 28.9 (25.6-32.5) | 39.5 (36.4-42.7) |  |
| Tertiary | 3667 (58.9%) | 42.1 (39.0-45.3) | 26.4 (24.6-28.2) | 31.5 (29.4-33.7) |  |
| Emergent obstetrical surgeries | |  |  |  | 0.477 |
| Primary | 1308 (68.2%) | 18.0 (15.0-21.5) | 19.1 (16.8-21.7) | 62.9 (58.5-67.0) |  |
| Secondary | 2490 (67.9%) | 14.9 (11.9-18.6) | 21.7 (19.1-24.6) | 63.3 (60.2-66.4) |  |
| Tertiary | 3744 (60.2%) | 15.1 (11.8-19.1) | 20.9 (18.9-23.1) | 64.0 (59.5-68.3) |  |
| Emergent gynecological surgeries | |  |  |  | 0.792 |
| Primary | 1235 (64.4%) | 22.5 (19.3-25.9) | 20.0 (17.6-22.7) | 57.5 (53.0-61.9) |  |
| Secondary | 2452 (66.9%) | 19.8 (16.1-24.2) | 20.5 (17.6-23.7) | 59.7 (56.5-62.9) |  |
| Tertiary | 3888 (62.5%) | 21.3 (17.3-25.8) | 20.2 (18.0-22.5) | 58.6 (55.9-61.2) |  |
| Cervical cancer screening | |  |  |  | <0.001 |
| Primary | 1267 (66.1%) | 39.5 (35.9-43.1) | 16.7 (14.2-19.5) | 43.8 (39.8-47.9) |  |
| Secondary | 2345 (64.0%) | 33.2 (30.0-36.4) | 24.4 (21.7-27.3) | 42.5 (39.4-45.6) |  |
| Tertiary | 3822 (61.4%) | 39.9 (36.2-43.6) | 23.3 (21.0-25.8) | 36.8 (34.5-39.3) |  |
| Treatment for benign neoplasm | |  |  |  | <0.001 |
| Primary | 1153 (60.1%) | 34.5 (31.0-38.2) | 21.1 (18.4-24.0) | 44.4 (40.7-48.1) |  |
| Secondary | 2313 (63.1%) | 31.6 (28.4-35.1) | 27.0 (24.0-30.2) | 41.4 (38.3-44.6) |  |
| Tertiary | 3812 (61.3%) | 40.3 (36.6-44.1) | 24.8 (22.0-28.0) | 34.8 (32.6-37.2) |  |
| Treatment for malignancies | |  |  |  | 0.109 |
| Primary | 937 (48.9%) | 27.2 (23.9-30.9) | 17.2 (14.5-20.3) | 55.6 (51.4-59.6) |  |
| Secondary | 2040 (55.6%) | 24.6 (20.7-29.1) | 19.4 (17.3-21.6) | 56.0 (51.6-60.3) |  |
| Tertiary | 3648 (58.6%) | 26.2 (23.0-29.6) | 22.4 (20.9-24.1) | 51.4 (48.6-54.2) |  |
| Follow-up for malignancies | |  |  |  | 0.003 |
| Primary | 923 (48.1%) | 26.5 (22.9-30.3) | 16.3 (13.9-19.1) | 57.2 (52.8-61.5) |  |
| Secondary | 1996 (54.4%) | 24.3 (20.7-28.3) | 18.8 (16.8-20.9) | 57.0 (53.3-60.6) |  |
| Tertiary | 3573 (57.4%) | 28.7 (25.8-31.7) | 22.2 (20.2-24.3) | 49.1 (46.6-51.6) |  |
| Treatment for pelvic floor dysfunctions | |  |  |  | <0.001 |
| Primary | 1106 (57.7%) | 36.3 (33.3-39.4) | 20.3 (18.0-22.8) | 43.4 (40.1-46.8) |  |
| Secondary | 2131 (58.1%) | 37.7 (35.1-40.3) | 22.5 (20.2-24.9) | 39.8 (36.7-43.1) |  |
| Tertiary | 3314 (53.3%) | 43.8 (41.1-46.4) | 21.8 (19.5-24.4) | 34.4 (31.9-37.0) |  |

# Table S7

Changes in the management of specific diseases or conditions according to hospital natures. 95% CI, 95% confidence interval.

|  | No. (%) of participants | Complete shutdown or >50% reduction, (% [95% CI]) | Reduction by 25%-50%, (% [95% CI]) | Reduction <25% or no change, (% [95% CI]) | P value for χ^2^ test |
| --- | --- | --- | --- | --- | --- |
| Preconception counseling | |  |  |  | 0.051 |
| General hospital | 6484 (70.1%) | 36.1 (33.6-38.6) | 31.7 (29.8-33.8) | 32.2 (30.3-34.1) |  |
| Specialized hospital | 1757 (68.7%) | 30.1 (25.4-35.4) | 31.5 (28.4-34.7) | 38.4 (34.9-42.0) |  |
| Prenatal examinations |  |  |  |  | <0.001 |
| General hospital | 6468 (69.9%) | 29.8 (27.5-32.3) | 31.9 (30.0-33.9) | 38.3 (36.3-40.3) |  |
| Specialized hospital | 1750 (68.5%) | 19.9 (17.0-23.2) | 33.0 (29.9-36.2) | 47.1 (44.0-50.2) |  |
| Prenatal diagnosis |  |  |  |  | <0.001 |
| General hospital | 5471 (59.1%) | 30.6 (28.4-32.9) | 27.9 (26.3-29.6) | 41.5 (39.3-43.7) |  |
| Specialized hospital | 1448 (56.7%) | 19.8 (16.7-23.3) | 28.1 (25.4-30.9) | 52.1 (48.1-56.2) |  |
| Instructions for contraception and miscarriage | |  |  |  | 0.003 |
| General hospital | 6480 (70.1%) | 31.5 (29.3-33.7) | 24.3 (22.7-25.9) | 44.3 (42.1-46.5) |  |
| Specialized hospital | 1669 (65.3%) | 22.8 (17.6-28.9) | 21.2 (19.3-23.2) | 56.0 (50.7-61.2) |  |
| Assistant reproduction |  |  |  |  | <0.001 |
| General hospital | 3083 (33.3%) | 41.1 (38.0-44.3) | 20.4 (18.4-22.6) | 38.5 (35.8-41.3) |  |
| Specialized hospital | 788 (30.8%) | 30.0 (26.7-33.4) | 24.4 (21.3-27.9) | 45.6 (40.8-50.5) |  |
| Outpatient surgeries and procedures | |  |  |  | <0.001 |
| General hospital | 5779 (62.5%) | 39.0 (36.7-41.5) | 26.9 (25.0-28.9) | 34.1 (32.1-36.1) |  |
| Specialized hospital | 1417 (55.4%) | 26.1 (21.4-31.5) | 26.1 (23.2-29.2) | 47.7 (41.7-53.9) |  |
| Emergent obstetrical surgeries | |  |  |  | <0.001 |
| General hospital | 6004 (64.9%) | 18.0 (15.7-20.5) | 22.2 (20.4-24.2) | 59.8 (57.5-62.1) |  |
| Specialized hospital | 1538 (60.2%) | 10.1 (8.0-12.6) | 16.7 (14.6-19.0) | 73.2 (69.5-76.6) |  |
| Emergent gynecological surgeries | |  |  |  | <0.001 |
| General hospital | 6102 (66.0%) | 23.1 (20.6-25.8) | 20.5 (18.7-22.5) | 56.4 (54.2-58.6) |  |
| Specialized hospital | 1473 (57.6%) | 15.2 (12.9-17.9) | 19.5 (16.8-22.5) | 65.3 (60.9-69.4) |  |
| Cervical cancer screening | |  |  |  | 0.004 |
| General hospital | 5979 (64.6%) | 39.1 (36.8-41.5) | 22.6 (21.0-24.2) | 38.3 (36.1-40.5) |  |
| Specialized hospital | 1455 (56.9%) | 32.5 (29.1-36.0) | 20.3 (16.9-24.3) | 47.2 (42.3-52.2) |  |
| Treatment for benign neoplasm | |  |  |  | 0.002 |
| General hospital | 5923 (64.0%) | 38.0 (35.6-40.5) | 24.8 (23.0-26.6) | 37.2 (35.2-39.3) |  |
| Specialized hospital | 1355 (53.0%) | 28.9 (24.7-33.4) | 24.5 (20.7-28.8) | 46.6 (41.0-52.3) |  |
| Treatment for malignancies | |  |  |  | 0.019 |
| General hospital | 5473 (59.2%) | 27.4 (24.8-30.2) | 19.8 (18.5-21.2) | 52.8 (49.9-55.7) |  |
| Specialized hospital | 1152 (45.1%) | 21.1 (18.0-24.5) | 21.0 (17.8-24.6) | 57.9 (53.7-62.0) |  |
| Follow-up for malignancies | |  |  |  | 0.056 |
| General hospital | 5366 (58.0%) | 28.0 (25.5-30.5) | 19.6 (18.4-21.0) | 52.4 (49.9-54.9) |  |
| Specialized hospital | 1126 (44.1%) | 22.1 (18.6-26.2) | 19.5 (16.2-23.2) | 58.4 (52.6-63.9) |  |
| Treatment for pelvic floor dysfunctions | |  |  |  | 0.013 |
| General hospital | 5290 (57.2%) | 41.9 (39.9-44.0) | 20.9 (19.2-22.7) | 37.2 (35.0-39.5) |  |
| Specialized hospital | 1261 (49.3%) | 32.8 (27.9-38.2) | 23.8 (21.5-26.3) | 43.4 (37.6-49.4) |  |

# Table S8

Changes in the management of specific diseases or conditions according to provinces. 95% CI, 95% confidence interval.

|  | No. (%) of participants | Complete shutdown or >50% reduction, (% [95% CI]) | Reduction by 25%-50%, (% [95% CI]) | Reduction <25% or no change, (% [95% CI]) | P value for χ^2^ test |
| --- | --- | --- | --- | --- | --- |
| Preconception counseling | |  |  |  | <0.001 |
| Hubei | 302 (80.3%) | 54.9 (46.2-63.3) | 24.1 (18.1-31.3) | 21.0 (14.0-30.3) |  |
| Others | 7939 (69.5%) | 33.3 (31.6-35.0) | 32.0 (30.6-33.6) | 34.7 (33.1-36.3) |  |
| Prenatal examinations | |  |  |  | <0.001 |
| Hubei | 305 (81.1%) | 47.8 (38.5-57.4) | 25.5 (19.1-33.2) | 26.7 (20.2-34.3) |  |
| Others | 7913 (69.2%) | 25.9 (24.0-27.9) | 32.6 (30.8-34.4) | 41.6 (39.9-43.2) |  |
| Prenatal diagnosis |  |  |  |  | <0.001 |
| Hubei | 275 (73.1%) | 51.6 (43.9-59.3) | 20.4 (14.5-27.8) | 28.0 (20.1-37.5) |  |
| Others | 6644 (58.1%) | 26.3 (24.5-28.2) | 28.4 (26.9-29.9) | 45.3 (43.4-47.3) |  |
| Instructions for contraception and miscarriage | |  |  |  | 0.025 |
| Hubei | 319 (84.8%) | 37.8 (34.3-41.4) | 23.0 (17.2-30.1) | 39.2 (31.6-47.3) |  |
| Others | 7830 (68.5%) | 28.5 (26.7-30.5) | 23.4 (22.1-24.8) | 48.1 (46.3-49.8) |  |
| Assistant reproduction | |  |  |  | 0.018 |
| Hubei | 154 (41.0%) | 57.6 (48.8-66.0) | 16.9 (8.8-29.9) | 25.5 (14.6-40.7) |  |
| Others | 3717 (32.5%) | 37.0 (34.3-39.7) | 21.8 (19.9-23.8) | 41.3 (38.9-43.7) |  |
| Outpatient surgeries and procedures | |  |  |  | 0.002 |
| Hubei | 277 (73.7%) | 49.7 (42.5-56.9) | 18.9 (15.0-23.4) | 31.5 (22.9-41.4) |  |
| Others | 6919 (60.5%) | 34.7 (32.8-36.7) | 27.1 (25.4-28.8) | 38.2 (36.3-40.1) |  |
| Emergent obstetrical surgeries | |  |  |  | 0.002 |
| Hubei | 292 (77.7%) | 25.6 (20.9-31.0) | 20.0 (15.1-26.1) | 54.4 (45.8-62.6) |  |
| Others | 7250 (63.4%) | 15.3 (13.3-17.5) | 20.8 (19.2-22.5) | 63.9 (61.5-66.3) |  |
| Emergent gynecological surgeries | |  |  |  | 0.001 |
| Hubei | 284 (75.5%) | 34.7 (27.2-43.0) | 20.2 (15.7-25.6) | 45.1 (35.0-55.6) |  |
| Others | 7291 (63.8%) | 20.3 (18.1-22.6) | 20.3 (18.6-22.0) | 59.5 (57.4-61.6) |  |
| Cervical cancer screening | |  |  |  | <0.001 |
| Hubei | 269 (71.5%) | 57.7 (49.9-65.2) | 17.6 (13.1-23.3) | 24.7 (16.9-34.5) |  |
| Others | 7165 (62.7%) | 36.3 (34.3-38.3) | 22.2 (20.7-23.7) | 41.5 (39.8-43.3) |  |
| Treatment for benign neoplasm | |  |  |  | 0.001 |
| Hubei | 268 (71.3%) | 55.2 (45.4-64.7) | 21.6 (14.8-30.3) | 23.2 (14.0-35.9) |  |
| Others | 7010 (61.3%) | 34.7 (32.6-36.8) | 24.9 (23.1-26.7) | 40.5 (38.7-42.3) |  |
| Treatment for malignancies | |  |  |  | <0.001 |
| Hubei | 254 (67.6%) | 49.6 (39.1-60.2) | 22.4 (16.5-29.7) | 28.0 (18.9-39.2) |  |
| Others | 6371 (55.7%) | 24.6 (22.4-27.0) | 20.0 (18.7-21.3) | 55.4 (53.0-57.7) |  |
| Follow-up for malignancies | |  |  |  | <0.001 |
| Hubei | 246 (65.4%) | 50.2 (39.0-61.3) | 17.6 (12.9-23.4) | 32.2 (23.1-42.9) |  |
| Others | 6246 (54.6%) | 25.3 (23.4-27.4) | 19.7 (18.4-21.1) | 55.0 (52.8-57.1) |  |
| Treatment for pelvic floor dysfunctions | |  |  |  | <0.001 |
| Hubei | 260 (69.1%) | 59.0 (52.0-65.6) | 12.8 (10.1-16.1) | 28.2 (20.9-36.9) |  |
| Others | 6291 (55.0%) | 38.4 (36.7-40.1) | 22.1 (20.7-23.7) | 39.5 (37.6-41.4) |  |
